# Gene expression profile in peripheral blood mononuclear cells of postpartum depression patients

**DOI:** 10.1038/s41598-018-28509-4

**Published:** 2018-07-04

**Authors:** Danqing Pan, Yuemei Xu, Lei Zhang, Qizhu Su, Manman Chen, Bing Li, Qian Xiao, Qi Gao, Xiuhua Peng, Binfei Jiang, Yilu Gu, Yuling Du, Pengfei Gao

**Affiliations:** 1 Department of TCM, Jinshan Hospital of Fudan University, Shanghai, China; 2 Department of Central Laboratory, Jinshan Hospital of Fudan University, Shanghai, China; 3 Department of Laboratory, Jinshan Hospital of Fudan University, Shanghai, China; 4 Department of Animal Experiments, Shanghai Public Health Clinical Center, Shanghai, China; 5 Department of Gynecology, Jinshan Hospital of Fudan University, Shanghai, China

## Abstract

Postpartum depression (PPD) is a common mental health problem that causes maternal suffering and various negative consequences for offspring. The pathogenesis of PPD and the causes of consequences for offspring remain largely unknown. Here, we applied RNA sequencing to sequence the whole transcriptomes of peripheral blood mononuclear cells (PBMCs) from PPD patients (Edinburgh Postnatal Depression Scale [EPDS] score ≥13) and control subjects (EPDS = 0). We found that PPD was positively correlated with multiple genes involved in energy metabolism, neurodegenerative diseases and immune response, while negatively correlated with multiple genes in mismatch repair and cancer-related pathways. Remarkably, genes associated with appetite regulation and nutrient response were differentially expressed between PPD and control subjects. Then, we employed a postnatal growth retardation model by repeated immobilization stress (IS) stimulation to maternal mice. The expression of appetite regulation and nutrient response-related genes in the PBMCs of IS mice and in the hypothalamus of their offspring were also affected. In conclusion, this study provides a comprehensive characterization of the PBMCs transcriptome in PPD and suggests that maternal stress may affect appetite regulation and nutrient response in the hypothalamus of offspring mice.

## Introduction

Postpartum depression (PPD) is one of the most prominent mood disorders that affects 10% to 15% of parturient women^1^. While the causes of PPD are not well understood, potential causes, including hormonal changes, genetics, and major life events, have been proposed. The symptom of PPD includes negative emotions (e.g., sadness, anxiety, worthlessness, and/or hopelessness), low energy and social withdrawal etc.^2^. PPD may lead mothers to be inconsistent with childcare and impair normal maternal-infant bonding^3^. Studies have revealed the effects of PPD on the behavioral, cognitive, and social impairments of infants^4,5^, as well as on infant physical health including poorer child cardiovascular functioning, higher rates of gastrointestinal infections and lower respiratory tract infections, and less weight gain^6–10^. Previous studies have reported a link between PPD symptoms and single nucleotide polymorphisms (SNPs) at several genes, such as 5-HTT, Catechol-O-methyl transferase (COMT), Monoamine Oxidase (MAO)^11^, Brain-Derived Neurotrophic Factor (BDNF)^12^ and Cytochrome P450 Family 2 Subfamily D Member 6 (CYP2D6)^13^. However, the pathogenesis of PPD and the causes of child consequences of PPD remain largely obscure.

Recently, gene expression profiling techniques provide powerful tools for identifying biomarkers and understanding the pathophysiology of mental disorders from the molecular level^14–17^. Brain tissue is rarely available for study. Previous studies have shown that peripheral blood cells share more than 80% of the transcriptome with brain tissues^18^. Comparable expression levels of many classes of biological processes have been observed in whole blood and prefrontal cortex^19^. Therefore, peripheral blood can be as a target tissue to explore mRNA expression profiling in PPD. Segman *et al*. found distinct gene expression signatures in patients with PPD by DNA microarrays^20^. However, array-based analysis is complicated by inconsistencies, limited sensitivity and hybridization artifacts^21^. In the current study, we applied next-generation RNA sequencing (RNA-Seq) technology to analyze mRNA expression profiling of peripheral blood mononuclear cells (PBMCs) from PPD patients and normal controls. The mRNA expression profiling was also studied in an immobilization stress-induced mice model. Further investigation indicated that several genes associated with appetite regulation and nutrient response may be related with the less weight gain of offspring.

## Results

### Analysis of gene expression by RNA-Seq in human subjects

Fifty-six women (age, 28 ± 4 years; BMI, 23 ± 6.1 kg/m^2^) with PPD (Edinburgh Postnatal Depression Scale [EPDS] ≥ 10) and 27 control subjects (age, 29 ± 4 years; body mass index [BMI], 23 ± 4.4 kg/m^2^) were enrolled in this study. Although there was no significant difference in the birth weight of the children between PPD and control subjects (Control: 3.26 ± 0.38 kg, PPD: 3.28 ± 0.47 kg), the weight gain of the children of mothers with PPD was significantly lower than those of control subjects (5.17 ± 0.84 kg vs. 5.75 ± 0.66 kg, *P* < 0.01) within 6 months. Of the 56 PPD patients, 28 patients with relative lower EPDS score (EPDS < 13) and 28 patients with relative higher EPDS score (EPDS ≥ 13). We performed RNA-seq on PBMCs from randomly selected PPD patients with higher EPDS (n = 10) and controls (n = 10) using the Illumina platform. Two PPD samples did not meet quality control measures and were removed from further analysis. Finally, based on the following criteria: *P* < 0.05 and fold change > 1.2, we identified a total of 2,164 significantly differentially expressed genes (DEGs) with 724 up-regulations (Supplementary Table 1) and 1,440 down-regulations (Supplementary Table 2) in PPD PBMCs, when compared with control PBMCs. Of these DEGs, 621 genes (172 up-regulations and 449 down-regulations) were with Fold change > 1.5.

### Multiple pathways were altered in PPD samples

Gene Set Enrichment Analysis (GSEA) was then performed to investigate functional associations of gene expression changes in the PBMCs samples (PPD and normal control). We generated a gene list with greatest changes using RNA-seq data, and the enrichment of Kyoto Encyclopedia of Genes and Genomes (KEGG) biological pathways was evaluated by GSEA. GSEA analysis indicated that 41 and 11 pathways were significantly altered in PPD and control samples, respectively, with *P* value less than 0.05 and FDR less than 0.25 (Table 1). Remarkably, PPD was positively correlated with multiple genes in energy metabolism (oxidative phosphorylation, pyruvate metabolism, glycolysis/gluconeogenesis, sphingolipid metabolism, galactose metabolism, ether lipid metabolism, tryptophan metabolism, citrate cycle/TCA cycle and adipocytokine signaling pathway), neurodegenerative diseases (Parkinson’s, Huntington’s, Alzheimer’s and Prion disease) and immune response (antigen processing and presentation, chemokine signaling pathway, cytokine/cytokine receptor interaction, Nod like receptor signaling pathway and Toll like Receptor signaling pathway), while negatively correlated with multiple genes in mismatch repair and cancer-related pathways.

**Table 1:** Enriched regulated (KEGG) biological pathways.

	NAME	SIZE	NES	NOM p value	FDR q value
**PPD**	OXIDATIVE_PHOSPHORYLATION	106	2.6082	0	0
	LYSOSOME	115	2.4018	0	0
	PARKINSONS_DISEASE	105	2.2859	0	0
	RIBOSOME	86	2.2179	0	0.0005
	HUNTINGTONS_DISEASE	161	2.1521	0	0.0004
	OTHER_GLYCAN_DEGRADATION	15	2.0702	0	0.0017
	PYRUVATE_METABOLISM	35	2.0367	0	0.0028
	PRION_DISEASES	28	1.9124	0.005618	0.0096
	CYTOSOLIC_DNA_SENSING_PATHWAY	42	1.8756	0	0.0124
	GLUTATHIONE_METABOLISM	43	1.8645	0	0.0127
	GLYCOSAMINOGLYCAN_DEGRADATION	19	1.8599	0	0.0119
	ALZHEIMERS_DISEASE	144	1.8592	0	0.0109
	FC_GAMMA_R_MEDIATED_PHAGOCYTOSIS	93	1.8459	0	0.0125
	CITRATE_CYCLE_TCA_CYCLE	28	1.8318	0.0031	0.0131
	GLYCOLYSIS_GLUCONEOGENESIS	47	1.8274	0.0034	0.0124
	SPHINGOLIPID_METABOLISM	38	1.8216	0	0.0120
	ANTIGEN_PROCESSING_AND_PRESENTATION	68	1.7736	0	0.0165
	GALACTOSE_METABOLISM	23	1.7530	0.0053	0.0202
	INTESTINAL_IMMUNE_NETWORK_FOR_IGA_PRODUCTION	44	1.7420	0	0.0213
	LEISHMANIA_INFECTION	70	1.7215	0	0.0238
	ETHER_LIPID_METABOLISM	26	1.7177	0.0158	0.0227
	PROTEASOME	43	1.6890	0.0088	0.0277
	GAP_JUNCTION	72	1.6688	0	0.0307
	AMINO_SUGAR_AND_NUCLEOTIDE_SUGAR_METABOLISM	42	1.6630	0.0094	0.0305
	SYSTEMIC_LUPUS_ERYTHEMATOSUS	99	1.6554	0.0038	0.0317
	CHEMOKINE_SIGNALING_PATHWAY	170	1.6361	0	0.0350
	GRAFT_VERSUS_HOST_DISEASE	37	1.6323	0.0129	0.0345
	TYPE_I_DIABETES_MELLITUS	38	1.6252	0.0058	0.0358
	NOD_LIKE_RECEPTOR_SIGNALING_PATHWAY	57	1.5896	0.0034	0.0467
	HEMATOPOIETIC_CELL_LINEAGE	80	1.5799	0.0118	0.0490
	TRYPTOPHAN_METABOLISM	33	1.5199	0.0177	0.0719
	CARDIAC_MUSCLE_CONTRACTION	55	1.5167	0.0174	0.0711
	GLYCOSAMINOGLYCAN_BIOSYNTHESIS_CHONDROITIN_SULFATE	21	1.5104	0.0478	0.0719
	CYTOKINE_CYTOKINE_RECEPTOR_INTERACTION	215	1.4982	0	0.0773
	ADIPOCYTOKINE_SIGNALING_PATHWAY	58	1.4974	0.0131	0.0757
	AUTOIMMUNE_THYROID_DISEASE	37	1.4612	0.0483	0.0966
	TOLL_LIKE_RECEPTOR_SIGNALING_PATHWAY	88	1.4398	0.0156	0.1078
	EPITHELIAL_CELL_SIGNALING_IN_HELICOBACTER_PYLORI_INFECTION	61	1.4138	0.0344	0.1242
	CELL_ADHESION_MOLECULES_CAMS	113	1.3490	0.0135	0.1705
	AXON_GUIDANCE	106	1.3444	0.0435	0.1717
	LEUKOCYTE_TRANSENDOTHELIAL_MIGRATION	96	1.3262	0.0377	0.1888
**Control**	ABC_TRANSPORTERS	36	−1.9567	0	0.0140
	MELANOMA	57	−1.8737	0	0.0262
	PHENYLALANINE_METABOLISM	16	−1.7663	0.00974	0.0574
	BLADDER_CANCER	39	−1.7562	0.00152	0.0494
	HOMOLOGOUS_RECOMBINATION	28	−1.6878	0.003135	0.0783
	PANCREATIC_CANCER	68	−1.6683	0.007082	0.0797
	MISMATCH_REPAIR	23	−1.6092	0.022187	0.1112
	ENDOMETRIAL_CANCER	50	−1.5847	0.022422	0.1204
	THYROID_CANCER	28	−1.5713	0.026114	0.1194
	PATHWAYS_IN_CANCER	292	−1.4819	0.001145	0.2108
	NON_SMALL_CELL_LUNG_CANCER	52	−1.4626	0.041018	0.2002

### Confirmation of expression measurements with real-time PCR and enzyme-linked immunosorbent assay (ELISA) analyses

**Figure 1:**
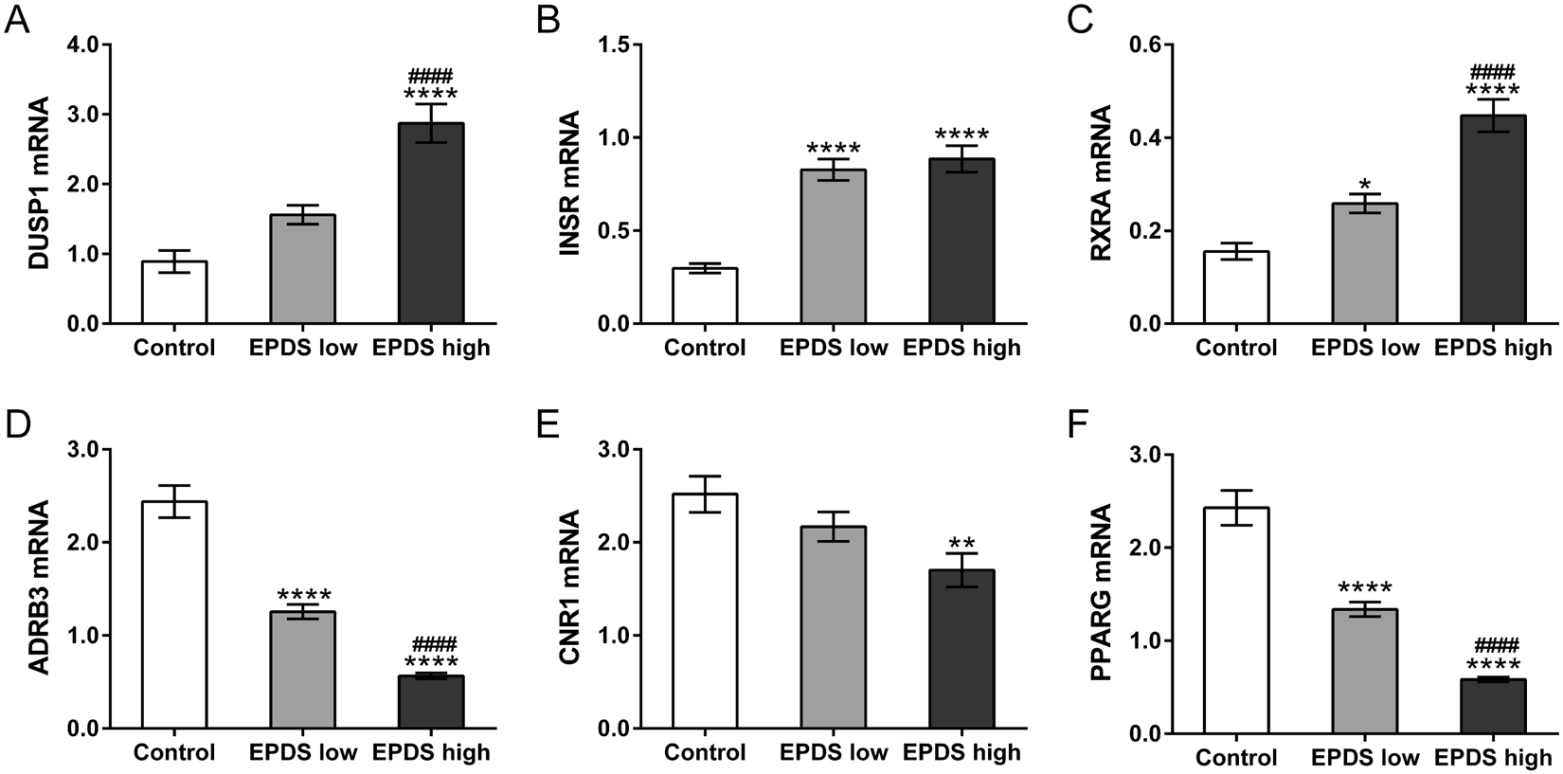
Validation of RNA-seq data by qRT-PCR. PBMCs were isolated from whole blood of control (n = 27), PPD patients with lower EPDS score (n = 28) and PPD patients with higher EPDS score (n = 28) for RNA extraction. mRNA levels of DUSP1 (**A**), INSR (**B**), RXRA (**C**), ADRB3 (**D**), CNR1 (**E**) and PPARG (**F**) were evaluated by qRT-PCR. *P < 0.05, **P < 0.01, ****P < 0.0001 vs. control; ^####^*P* < 0.0001 vs. EPDS low.

Eight genes associated with appetite regulation and nutrient response, Interleukin 1 Beta (IL1B), Dual Specificity Phosphatase 1 (DUSP1), Retinoid X Receptor Alpha (RXRA), Insulin Receptor (INSR), Adrenoceptor Beta 3 (ADRB3), Cannabinoid Receptor 1 (CNR1), Cyclin D1 (CCND1) and Peroxisome Proliferator Activated Receptor Gamma (PPARG), were identified as DEGs in this study (Table 2). To confirm the results of RNA-seq, real-time PCR was performed to detect the mRNA expression of DUSP1, INSR, RXRA, ADRB3, CNR1 and PPARG in PBMCs from control (n = 27), PPD patients with lower EPDS score (n = 28) and PPD patients with higher EPDS score (n = 28). The variation trend was consistent with RNA-seq results. As shown in Fig. 1, mRNA levels of DUSP1 and CNR1 had significant difference between higher-EPDS-score group and control group, while comparable mRNA levels were observed in lower-EPDS-score group comparing with control group. INSR mRNA levels were higher in PPD groups compared to control group, and no significant difference was observed between higher-EPDS-score group and lower-EPDS-score group. RXRA mRNA levels were progressively increased, and mRNA levels of ADRB3 and PPARG were gradually decreased with the increasing of EPDS score. No obvious difference was observed in the p-values between ADRB3 (RNA-seq FDR = 1.61E-23) and other genes (RNA-seq FDR > 0.2) (Table 3).

**Table 2:** DEGs associated with appetite regulation and nutrition level.

	Gene	log2 Fold Change	*P* value	FDR
**Up-regulated**	IL1B	0.7158	0.0080	0.1602
	DUSP1	0.4469	0.0369	0.3282
	INSR	0.4307	0.0134	0.2005
	RXRA	0.2851	0.0459	0.3570
**Down-regulated**	ADRB3	−2.8836	1.26E-26	1.61E-23
	CNR1	−0.4962	0.0357	0.3233
	PPARG	−0.4593	0.0487	0.3661
	CCND1	−0.4318	0.0197	0.2419

**Table 3:** p-values of qRT-PCR and RNA-sequencing.

	Gene	*P* value	
		RNA-sequencing	qRT-PCR
**Up-regulated**	DUSP1	0.0369	3.4559E-09
	INSR	0.0134	2.6645E-11
	RXRA	0.0459	3.2669E-11
**Down-regulated**	ADRB3	1.26E-26	1.1102E-16
	CNR1	0.0357	0.0069
	PPARG	0.0487	1.1102E-16

**Figure 2:**
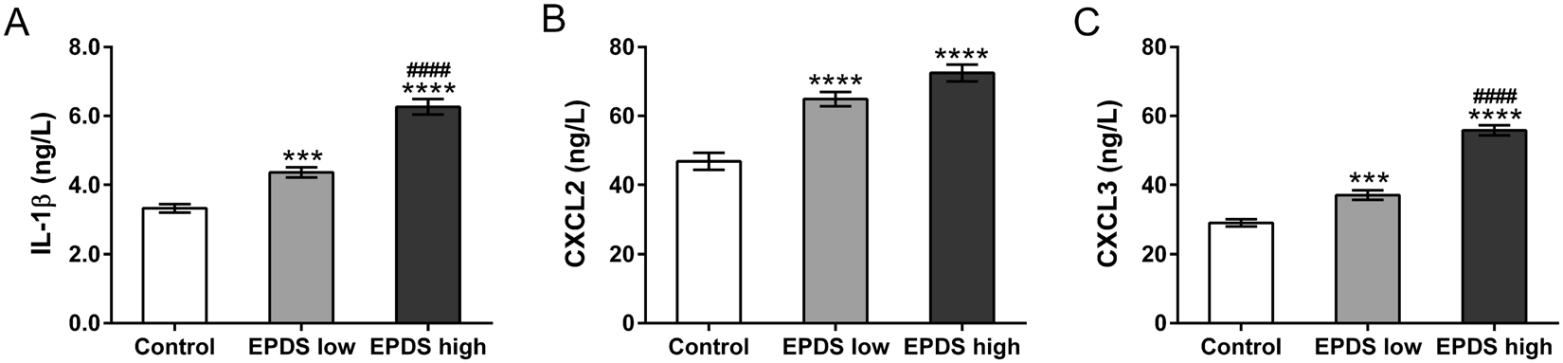
The protein levels of IL-1β (**A**), CXCL2 (**B**) and CXCL3 (**C**) in the sera were evaluated by ELISA assay. ***P < 0.001, ****P < 0.0001 vs. control; ^####^*P* < 0.0001 vs. EPDS low.

Genes encoded chemokines and cytokines, including IL1B, C-X-C Motif Chemokine Ligand 2/3/16 (CXCL2/3/16) and C-C Motif Chemokine Ligand 3/24/28 (CCL3/24/28) were identified up-regulated genes by RNA-seq (Table 4). The altered levels of cytokines/chemokines in the serum have been reported in various human diseases, such as hear failure^22^, cancers^23^ and systemic lupus erythematosus^24^. We then performed ELISA assays to detect the protein levels of IL1β, CXCL2 and CXCL3 in the serum form control (n = 27), PPD patients with lower EPDS score (n = 28) and PPD patients with higher EPDS score (n = 28). Figure 2 revealed the serum concentrations of IL1B, CXCL2 and CXCL3 were significantly increased in both PPD groups compared to control group.

**Table 4:** DEGs associated with immune response.

	Gene	log2 Fold Change	*P* value	FDR
Up-regulated	IL1B	0.7158	0.0080	0.1602
	CXCL2	0.8516	0.0021	0.0818
	CXCL3	0.5978	0.0149	0.2119
	CXCL16	0.3540	0.0398	0.3392
	CCL3	0.7593	0.0059	0.1384
	CCL24	0.4951	0.0115	NA
Down-regulated	IL13	−1.6424	8.31E-10	4.32E-07
	IL22	−0.6187	0.0239	NA
	IL4	−0.5131	0.0325	0.3093
	CCL28	−0.3019	0.0220	0.2558

### RNA sequencing analysis on a mouse model

**Figure 3:**
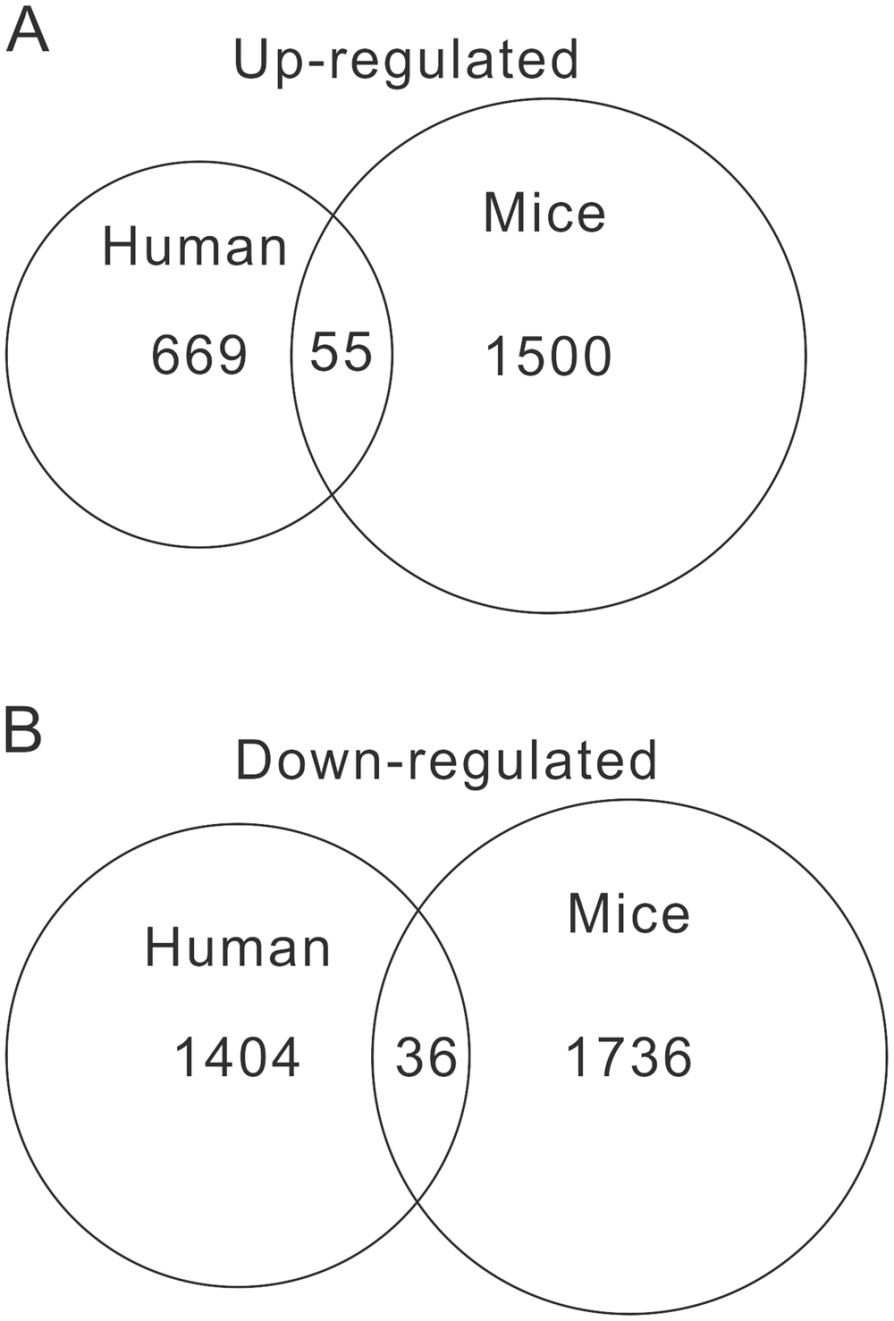
Venn-analysis of DEGs. (**A**) Overlap of up-regulated DEGs (human PPD VS control, mouse IS VS control). (**B**) Overlap of down-regulated DEGs (human PPD VS control, mouse IS VS control).

To further explore how PPD affects weight gain of children, we established a postnatal growth retardation model by repeated immobilization stress (IS) stimulation to maternal mice. At 1 week and 3 weeks after birth, the offspring mice were weighed. Maternal IS significantly decreased the body weight of their offspring (1 week old: Control, 4.94 ± 0.64 g; IS, 3.90 ± 0.66 g; 3 weeks old: Control, 14.12 ± 2.82 g; IS, 10.44 ± 1.90 g, *P* < 0.0001) within 3 weeks. We then performed RNA-seq on PMBCs from three pairs of control mice and IS mice. A total of 3,327 significantly DEGs with 1,555 up-regulations (Supplementary Table 3) and 1,772 down-regulations (Supplementary Table 4) were identified in IS mice, when compared with control mice (*P* < 0.05 and fold change > 1.2). Of these DEGs, 1817 genes (840 up-regulations and 977 down-regulations) were with Fold change > 1.5. We found that 55 DEGs were up-regulated (including appetite regulation and nutrient response-related genes, IL1B, DUSP1 and RXRA), while 36 DEGs (including CCND1) were down-regulated in PBMCs samples both from PPD patients and IS mice (Fig. 3).

### Maternal IS altered gene expression levels in hypothalamus of offspring mice

**Figure 4:**
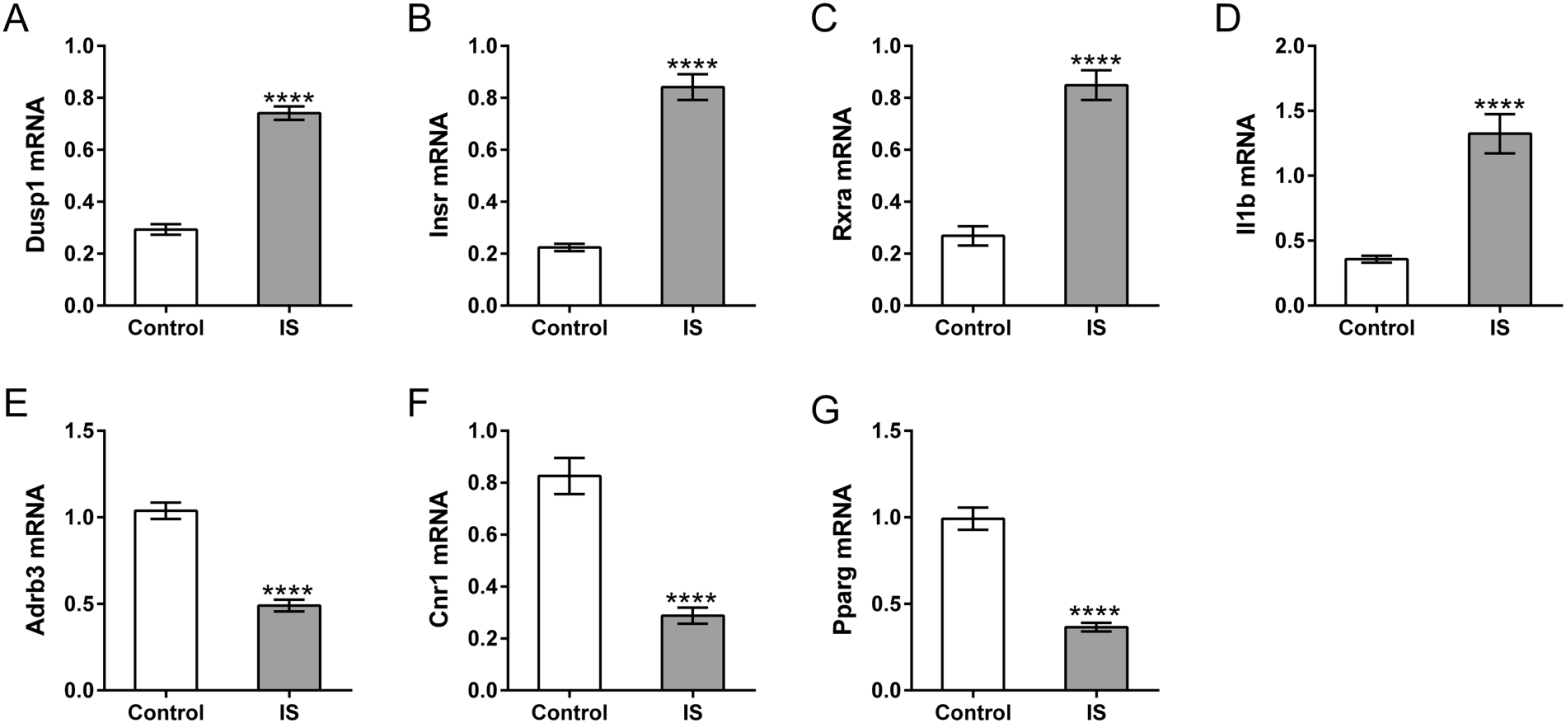
mRNA expression in the hypothalamus of offspring mice by qRT-PCR. The Hypothalamus was collected from the offspring mice of IS and control group for RNA extraction (n = 6). mRNA levels of Dusp1 (**A**), Insr (**B**), Rxra (**C**), Il1b (**D**), Adrb3 (**E**), Cnr1 (**F**) and Pparg (**G**) were evaluated by qRT-PCR. ****P < 0.0001 vs. control.

The appetite-satiety centers in the hypothalamus are also influenced by stress^25^. We hypothesized that the expression of appetite regulation and nutrient response-related genes in the hypothalamus of offspring mice were also affected by maternal IS. As shown Fig. 4, Dusp1, Insr, Rxra and Il1b significantly increased in the IS group, while Adrb3, Cnr1 and Pgarg notably decreased in IS group as compared to the Control group. The change trends were consistent with the findings in the PPD patients.

## Discussion

Maternal postpartum depression has become a significant public concern because of its increasing prevalence, high severity and great impact on both children and mothers. Although there are numerous studies examining the prevalence and correlates of PPD, the pathophysiology and the causes of child consequences of PPD remains largely obscure. In this study, we conducted the transcriptome sequencing for PBMCs from PPD patients and controls. We analyzed the expression difference at gene levels between PPD and controls and identified 2,164 DEGs. Some of the DEGs were identified as differentially expressed in a previous report such as Cyclin B1 (CCNB1), Early Growth Response 2 (EGR2), Polo Like Kinase 1 (PLK1) and Spermatogenesis Associated 20 (SPATA20)^20^, while most were novel genes. Functional analyses indicated that 41 and 11 KEGG pathways were significantly altered in PPD and control samples, respectively. Remarkably, PPD was positively correlated with multiple genes in energy metabolism, neurodegenerative diseases and immune response, while negatively correlated with multiple genes in mismatch repair and cancer-related pathways. A previous microarray study has found a signature of 73 genes in PBMCs (fold change > 1.5) between PPD patients and controls^20^, and found differences in transcription and immune activation. Here, we have identified more DEGs (621 with fold change > 1.5) and more pathways associated with PPD, suggesting RNA-seq is a powerful and sensitive tool for expression profiling.

We revealed the effects of maternal PPD on the weight gain of infants, which were in line with previous reports^6–8^. Stressful life events strongly correlated with the onset and progression of PPD^2^. Repeated immobilization stress (IS) stimulation has been widely used to study major depression^26,27^. Here, repeated maternal IS stimulation^28^ significantly decreased the body weight of their offspring within 3 weeks. RNA-seq was then performed on PBMCs from maternal mice samples. A total of 55 up-regulated genes and 36 down-regulated genes were identified in both samples from PPD patients and IS mice. Ray S *et al*. had reported the gene expression changes in the maternal brain during pregnancy and the postpartum period by using RNA-seq.^29^. They identified differential expression genes during pregnancy and the postpartum period implicated in PPD and depressive disorder. Among the DEGs identified in our present study, 2 DEGs implicated in PPD and 28 DEGs implicated in depressive disorder have been reported by Ray S *et al*.^29^ (Table S6). The appetite-satiety centers in the hypothalamus are influenced by stress^25^. Glucocorticoid treatment lowered the gain in body weight of rats^30^ and Dusp1 is a glucocorticoid target gene in rat hypothalamus^31^. Mice with a neuron-specific disruption of Insr gene had increased body fat^32^. Il1b is an anorectic gene and Cnr1 is an orexigenic gene in the hypothalamus^33^. PPARγ activation in the brain results in increased food intake and weight gain^34^. Here, qRT-PCR results showed that maternal IS increased the expression of Dusp1, Insr and Il1b, but reduced the expression of Cnr1 and Pgarg in the hypothalamus of offspring mice. These findings suggested that maternal stress may affect the weight of offspring by regulating the appetite. Adrb3 is an energy expenditure gene in the hypothalamus^33^. Rxra, an important adipogenesis regulator, has been found expressed in the central nervous system of mice^35^ and its function in the brain is unclear. Here, maternal IS treatment led to decreased Adrb3 expression and increased Rxra expression in the hypothalamus of offspring, which also suggested the regulatory role of maternal stress on energy balance and nutrient response of their offspring. Further investigation are needed to explore the detailed mechanisms how maternal stress affected the expression of these genes.

In summary, RNA-based approach provides a comprehensive gene expression profiling in PMBCs of PPD. Gene-set enrichment analysis further identified differences in biological pathways relating to energy metabolism, neurodegenerative diseases, immune response, mismatch repair and cancer-related pathways between the PPD and control groups. The IS mouse model experiments suggest that maternal stress may regulate the energy balance and nutrient response of their offspring, thus reducing the body weight.

## Materials and Methods

### Patients and sample collection

The study protocol was approved by the Institutional Review Board of Jinshan Hospital of Fudan University, and written informed consent was obtained. The study was carried out according to the relevant guidelines. Depressive symptom scores were evaluated using the Edinburgh Postnatal Depression Scale (EPDS) instrument^36^ on six weeks after delivery. Mothers with EPDS score ≥ 10 (n = 56) were included in this study. Mothers with EPDS score = 0 (n = 27) after delivery and showing no depressive symptoms were set as control subjects. The control subjects were matched for age, sex, and BMI with the PPD subjects. Subjects were not included if they had a history of past or present psychiatric diagnoses, had medical or neurological illness, or had used antidepressants or other psychotropic medications. Peripheral blood samples were collected by venipuncture from study subjects after delivery. Ten paired samples were randomly selected for RNA-sequencing, and the remaining samples were subjected to real-time PCR and ELISA analyses.

### Animal experiments

The animal study was approved by Animal Care Institutional Review Board of Fudan University and performed in accordance with the relevant guidelines and regulations. ICR mice (CLEA Japan, Inc., Tokyo, Japan) were kept at 21 ± 1 °C under a 12/12 h light-dark cycle. After 1 week of acclimatization, one female was housed with two males in each cage for 4 days until a copulation plug was found. On postpartum day 1, immobilization stress (IS) was performed by transferring mice from their home cage to a small meshed cylinder (3 × 6 cm). IS was applied to the animals for 3 hours every day for 3 weeks. The mice were sacrificed by cervical dislocation, blood was collected from the right ventricle, and the hypothalamus was dissected out as previously described^37^ and kept at −80 °C until use.

### RNA extraction, sequencing and data analysis

PBMCs were separated from collected whole blood by centrifugation at 300 g for 30 minutes with mononuclear/polynuclear cell-resolving medium (Flow Laboratories, Rockville, MD, USA). Total RNA was isolated with the Trizol reagent following the manufacturer’s instructions (Invitrogen, Carlsbad, CA, USA). The RNA yields were quantified by NanoDrop ND1000 (Thermo-Fisher Scientific, Waltham, MA, USA). cDNA sequencing libraries were prepared from 10 pairs of human samples and 3 pairs of animal samples using Illumina’s TruSeq Sample Preparation Kit (San Diego, CA, USA), which captured polyA-containing mRNAs, with 2 μg of total RNA. RNA single-end sequencing was performed using Illumina Genome Analyzer II using the standard protocol. Sequenced reads were base-called by using the Illumina standard pipeline.

The cleaning reads were mapped to the human genome (hg19) using TopHat v2.0.11 with the default options with a TopHat transcript index built from Ensembl GRCh37. Count files of the aligned sequencing reads were gene rated by the htseq-count script from the Python package HTSeq with union mode, using the GTF annotation file. The read counts from each sequenced sample were combined into a count file, which was subsequently used for the differential expression analysis. Differential analyses were performed to the count files using DESeq. 2 packages, following standard normalization procedures^38^. Genes with less than 5 total counts in both conditions were removed from further analysis.

### Pathway analysis

We identified DEGs between PPD and control samples based on the following criteria: *P* < 0.05 and fold change > 1.2. Gene Set Enrichment Analysis (GSEA) software was used to determine whether a total of 178 gene sets from KEGG showed statistically significant, concordant differences between PPD patients (n = 8) and normal controls (n = 10) as describe previously^39^. Differentially expressed gene sets/pathways were identified with threshold false discovery rate (FDR) less than 0.5 and *P* less than 0.05.

### Quantitative real time polymerase chain reaction (RT-PCR)

PBMC RNA was extracted from 27 control subjects, 28 PPD patients with lower EPDS score (10 ≤ EPDS < 13) and 28 PPD patients with higher EPDS score (≥13) and reverse transcribed with RevertAid First Strand cDNA Synthesis Kit (Thermo-Fischer Scientific). Gene expression was conducted on Applied Biosystems 7300 instrument (Applied Biosystems, Foster City, CA, USA) using SYBR-green PCR Master Mix (Thermo-Fischer Scientific). Gene expression values were calculated with the ΔΔ Ct method^40^ and GAPDH was served as reference gene. ANOVA analysis was used to calculate the statistical significance of difference among groups. Primers were listed in Supplementary Table 5.

### ELISA analysis

Serum was separated from collected whole blood by centrifugation at 2,500 rpm for 15 min at 4 °C. Serum concentrations of IL-1β, CXCL2 and CXCL3 were determined with ELISA assay (Bio-Swamp life science, Shanghai, China) following the instructions of the manufacturer. Absorbance was read at 450 nm using a microplate reader (Bio-Rad Laboratories Inc., Hercules, CA, USA). ANOVA analysis was performed to calculate the statistical significance of difference among groups.

## Electronic supplementary material

Supplementary File
