# Supplementary material for: Gene expression profile in peripheral blood mononuclear cells of postpartum depression patients
Source: Sci Rep. 2018 Jul 4;8:10139. doi: 10.1038/s41598-018-28509-4 (PMC6031634; doi:10.1038/s41598-018-28509-4)
Supplement: Supplementary file 1 — Supplementary File [file 41598_2018_28509_MOESM1_ESM.doc]

**Gene expression profile in peripheral blood mononuclear cells of postpartum depression patients**

Danqing Pan*^1^, Yuemei Xu*^1^, Lei Zhang*^1^, Qizhu Su*^2^, Manman Chen^1^, Bing Li^3^, Qian Xiao^1^, Qi Gao^1^, Xiuhua Peng^4^, Binfei Jiang^1^, Yilu Gu^5^, Yuling Du^1^, Pengfei Gao^1^

^1^Department of TCM, Jinshan Hospital of Fudan University, Shanghai, China.

^2^Department of Central Laboratory, Jinshan Hospital of Fudan University, Shanghai, China.

^3^Department of Laboratory, Jinshan Hospital of Fudan University, Shanghai, China.

^4^Department of Animal Experiments, Shanghai Public Health Clinical Center, China.

^5^Department of Gynecology, Jinshan Hospital of Fudan University, Shanghai, China.

*Equal contributors.

**Corresponding author:** Pengfei Gao, Department of TCM, Jinshan Hospital of Fudan University, Shanghai, China; Telephone: 86-21-57039600, Fax: 86-21-67226910; Email: gaopengfeibm@163.com.

**Supplementary Table 1. Upregulated genes in PBMCs from PPD patients.**

|  | **Gene** | **log2 Fold Change** | **p value** |
| --- | --- | --- | --- |
| 1 | SDSL | 1.2479 | 5.16E-11 |
| 2 | CTA-331P3.1 | 1.1491 | 3.24E-05 |
| 3 | RP11-281P23.1 | 1.0449 | 0.0001 |
| 4 | UPK3A | 1.0163 | 2.36E-05 |
| 5 | RP11-365O16.6 | 0.9936 | 0.0002 |
| 6 | PPFIA4 | 0.9765 | 4.43E-05 |
| 7 | OR2A20P | 0.9233 | 0.0009 |
| 8 | RP11-572C15.5 | 0.9156 | 0.0010 |
| 9 | IGLV7-46 | 0.9084 | 0.0003 |
| 10 | RP11-745O10.2 | 0.9068 | 0.0009 |
| 11 | CTF1 | 0.9005 | 0.0006 |
| 12 | DMRT2 | 0.8998 | 0.0010 |
| 13 | SPSB2 | 0.8951 | 0.0004 |
| 14 | COL25A1 | 0.8858 | 0.0010 |
| 15 | TRAV12-2 | 0.8823 | 0.0001 |
| 16 | RP11-187C18.4 | 0.8792 | 0.0009 |
| 17 | AREG | 0.8619 | 0.0016 |
| 18 | LINC00936 | 0.8545 | 0.0001 |
| 19 | CXCL2 | 0.8516 | 0.0021 |
| 20 | TDRD15 | 0.8451 | 0.0017 |
| 21 | RNU4ATAC | 0.8188 | 0.0033 |
| 22 | AC011515.2 | 0.8068 | 0.0038 |
| 23 | FFAR3 | 0.8035 | 0.0027 |
| 24 | RN7SL832P | 0.7971 | 0.0027 |
| 25 | EGR2 | 0.7953 | 0.0043 |
| 26 | NCBP2L | 0.7911 | 0.0045 |
| 27 | AL592284.1 | 0.7871 | 0.0036 |
| 28 | NUTM2E | 0.7807 | 0.0051 |
| 29 | RYR2 | 0.7797 | 0.0031 |
| 30 | ZDHHC1 | 0.7781 | 0.0001 |
| 31 | RP11-286H14.6 | 0.7774 | 0.0042 |
| 32 | SNORD3B-2 | 0.7736 | 0.0019 |
| 33 | MAFB | 0.7732 | 0.0003 |
| 34 | CTD-2006K23.2 | 0.7689 | 0.0041 |
| 35 | KCNE1L | 0.7639 | 0.0062 |
| 36 | RASD1 | 0.7615 | 0.0058 |
| 37 | CCL3 | 0.7593 | 0.0059 |
| 38 | AC096579.13 | 0.7586 | 0.0063 |
| 39 | RP11-138I18.2 | 0.7544 | 0.0038 |
| 40 | RHOB | 0.7538 | 0.0000 |
| 41 | RP11-712B9.5 | 0.7510 | 0.0052 |
| 42 | KAZALD1 | 0.7507 | 0.0067 |
| 43 | RP11-498C9.12 | 0.7505 | 0.0025 |
| 44 | FAM166A | 0.7435 | 0.0073 |
| 45 | RP11-343H5.4 | 0.7394 | 0.0066 |
| 46 | CTB-113D17.1 | 0.7355 | 0.0039 |
| 47 | RP11-190A12.8 | 0.7329 | 0.0006 |
| 48 | HBEGF | 0.7311 | 0.0082 |
| 49 | STK19P | 0.7309 | 0.0087 |
| 50 | RGPD2 | 0.7299 | 0.0032 |
| 51 | RP11-293M10.2 | 0.7282 | 0.0033 |
| 52 | SGK1 | 0.7242 | 0.0015 |
| 53 | ZNF503 | 0.7231 | 0.0061 |
| 54 | IL5RA | 0.7225 | 0.0017 |
| 55 | FOSB | 0.7212 | 0.0071 |
| 56 | CHRNA2 | 0.7197 | 0.0036 |
| 57 | EFNA5 | 0.7187 | 0.0043 |
| 58 | VDAC1P8 | 0.7165 | 0.0092 |
| 59 | IL1B | 0.7158 | 0.0080 |
| 60 | MUC20 | 0.7155 | 0.0041 |
| 61 | TSKS | 0.7011 | 0.0010 |
| 62 | KLF4 | 0.6991 | 0.0074 |
| 63 | RP11-139K1.2 | 0.6977 | 0.0120 |
| 64 | RP11-277A4.4 | 0.6963 | 0.0119 |
| 65 | NR4A1 | 0.6952 | 0.0115 |
| 66 | CES1 | 0.6951 | 0.0103 |
| 67 | LINC01023 | 0.6887 | 0.0030 |
| 68 | RP11-474L23.3 | 0.6884 | 0.0107 |
| 69 | LAMC1 | 0.6871 | 0.0011 |
| 70 | SNX7 | 0.6868 | 0.0110 |
| 71 | FAM212B-AS1 | 0.6857 | 0.0129 |
| 72 | IGHV2-70 | 0.6851 | 0.0100 |
| 73 | RP11-17M16.1 | 0.6807 | 0.0141 |
| 74 | WI2-89031B12.1 | 0.6796 | 0.0125 |
| 75 | FOS | 0.6787 | 0.0098 |
| 76 | ABHD17AP6 | 0.6776 | 0.0114 |
| 77 | CPSF1P1 | 0.6759 | 0.0030 |
| 78 | CTD-2587H24.1 | 0.6742 | 0.0089 |
| 79 | MRS2P2 | 0.6725 | 0.0140 |
| 80 | EGR1 | 0.6691 | 0.0144 |
| 81 | SDC3 | 0.6681 | 0.0020 |
| 82 | PHLDA2 | 0.6672 | 0.0159 |
| 83 | CHST8 | 0.6655 | 0.0154 |
| 84 | CTB-193M12.3 | 0.6653 | 0.0145 |
| 85 | TRIM40 | 0.6642 | 0.0090 |
| 86 | RP11-225H22.4 | 0.6632 | 0.0174 |
| 87 | GRHL1 | 0.6626 | 0.0070 |
| 88 | RNF182 | 0.6604 | 0.0121 |
| 89 | RP4-665N4.4 | 0.6600 | 0.0067 |
| 90 | TEX29 | 0.6591 | 0.0077 |
| 91 | AC084117.3 | 0.6589 | 0.0177 |
| 92 | KB-1572G7.3 | 0.6565 | 0.0079 |
| 93 | TPPP3 | 0.6544 | 0.0046 |
| 94 | RP11-218F4.1 | 0.6500 | 0.0162 |
| 95 | SLC10A1 | 0.6500 | 0.0140 |
| 96 | XIRP2 | 0.6498 | 0.0177 |
| 97 | HERC2P3 | 0.6471 | 0.0188 |
| 98 | AL662800.1 | 0.6463 | 0.0081 |
| 99 | RP11-308N19.1 | 0.6448 | 0.0099 |
| 100 | GPR1 | 0.6440 | 0.0208 |
| 101 | SCUBE1 | 0.6425 | 0.0178 |
| 102 | TMEM99 | 0.6413 | 0.0001 |
| 103 | IGHVIII-67-4 | 0.6412 | 0.0062 |
| 104 | RP5-1154L15.2 | 0.6407 | 0.0098 |
| 105 | CTA-85E5.10 | 0.6400 | 0.0216 |
| 106 | RP13-467H17.1 | 0.6398 | 0.0168 |
| 107 | RP11-757G1.6 | 0.6397 | 0.0081 |
| 108 | CCNB1 | 0.6358 | 0.0007 |
| 109 | RPS23P9 | 0.6356 | 0.0156 |
| 110 | HLA-DPB1 | 0.6354 | 0.0001 |
| 111 | CTD-2562J15.4 | 0.6352 | 0.0223 |
| 112 | TMEM191C | 0.6333 | 0.0031 |
| 113 | ANO2 | 0.6319 | 0.0199 |
| 114 | IGLL5 | 0.6319 | 0.0075 |
| 115 | HIST3H2BB | 0.6315 | 0.0233 |
| 116 | FPR3 | 0.6294 | 0.0166 |
| 117 | CHCHD4P2 | 0.6293 | 0.0060 |
| 118 | BCL2L2-PABPN1 | 0.6291 | 0.0241 |
| 119 | DUSP6 | 0.6287 | 0.0008 |
| 120 | SLC25A24P1 | 0.6264 | 0.0244 |
| 121 | PPAP2B | 0.6257 | 0.0099 |
| 122 | CDH20 | 0.6257 | 0.0159 |
| 123 | AC004895.4 | 0.6226 | 0.0223 |
| 124 | CLDN4 | 0.6172 | 0.0256 |
| 125 | RNASE2 | 0.6169 | 0.0043 |
| 126 | TPSP2 | 0.6145 | 0.0113 |
| 127 | MAEL | 0.6142 | 0.0268 |
| 128 | RP11-57H14.2 | 0.6137 | 0.0214 |
| 129 | KCNJ1 | 0.6121 | 0.0065 |
| 130 | LINC00514 | 0.6111 | 0.0280 |
| 131 | RPS23P10 | 0.6110 | 0.0224 |
| 132 | IGHD1-1 | 0.6105 | 0.0276 |
| 133 | RN7SL574P | 0.6096 | 0.0273 |
| 134 | RP3-375P9.2 | 0.6095 | 0.0002 |
| 135 | CTD-2561B21.4 | 0.6094 | 0.0152 |
| 136 | MIR4671 | 0.6089 | 0.0105 |
| 137 | C1QA | 0.6087 | 0.0043 |
| 138 | SLC2A3P4 | 0.6072 | 0.0252 |
| 139 | ZACN | 0.6066 | 0.0139 |
| 140 | JPH4 | 0.6061 | 0.0093 |
| 141 | CTD-3064M3.1 | 0.6059 | 0.0259 |
| 142 | AP001046.5 | 0.6057 | 0.0150 |
| 143 | CDKN1A | 0.6043 | 0.0089 |
| 144 | RHEBP2 | 0.6040 | 0.0272 |
| 145 | TUBB6 | 0.6035 | 0.0059 |
| 146 | RP11-533E19.5 | 0.6033 | 0.0305 |
| 147 | IGLC2 | 0.6029 | 0.0158 |
| 148 | ACOX2 | 0.6026 | 0.0305 |
| 149 | BRWD1-IT2 | 0.5999 | 0.0315 |
| 150 | TRIB1 | 0.5993 | 0.0019 |
| 151 | RP3-462E2.3 | 0.5993 | 0.0185 |
| 152 | GS1-421I3.2 | 0.5987 | 0.0263 |
| 153 | AC027307.3 | 0.5986 | 0.0228 |
| 154 | MYO7B | 0.5984 | 0.0048 |
| 155 | RP11-536K7.5 | 0.5979 | 0.0321 |
| 156 | CXCL3 | 0.5978 | 0.0149 |
| 157 | C9orf43 | 0.5975 | 0.0168 |
| 158 | LL22NC03-86G7.1 | 0.5952 | 0.0005 |
| 159 | RNU4-2 | 0.5951 | 0.0120 |
| 160 | CTA-384D8.35 | 0.5930 | 0.0199 |
| 161 | DRP2 | 0.5920 | 0.0333 |
| 162 | RP1-224A6.3 | 0.5920 | 0.0297 |
| 163 | RP11-343B5.1 | 0.5917 | 0.0309 |
| 164 | HES4 | 0.5912 | 0.0090 |
| 165 | CSMD1 | 0.5888 | 0.0277 |
| 166 | COLCA1 | 0.5885 | 0.0303 |
| 167 | ATF3 | 0.5885 | 0.0211 |
| 168 | C19orf67 | 0.5872 | 0.0272 |
| 169 | GPNMB | 0.5871 | 0.0103 |
| 170 | ATP6AP1L | 0.5860 | 0.0014 |
| 171 | ZCWPW2 | 0.5859 | 0.0222 |
| 172 | C15orf53 | 0.5855 | 0.0351 |
| 173 | RP11-326C3.4 | 0.5829 | 0.0328 |
| 174 | AP003041.2 | 0.5828 | 0.0361 |
| 175 | UNC5B | 0.5799 | 0.0268 |
| 176 | GFPT2 | 0.5789 | 0.0141 |
| 177 | FXYD6 | 0.5785 | 0.0094 |
| 178 | RPL7AP60 | 0.5784 | 0.0249 |
| 179 | CTD-2589M5.5 | 0.5779 | 0.0269 |
| 180 | DNER | 0.5774 | 0.0361 |
| 181 | PEG10 | 0.5764 | 0.0152 |
| 182 | CFD | 0.5754 | 0.0097 |
| 183 | SH3PXD2B | 0.5748 | 0.0116 |
| 184 | LINC01088 | 0.5746 | 0.0356 |
| 185 | AC034229.1 | 0.5740 | 0.0394 |
| 186 | SNORD3D | 0.5726 | 0.0359 |
| 187 | AC083949.1 | 0.5723 | 0.0117 |
| 188 | OLFML2B | 0.5696 | 0.0131 |
| 189 | RP11-284F21.10 | 0.5690 | 0.0286 |
| 190 | MIR221 | 0.5685 | 0.0404 |
| 191 | RP11-548H18.2 | 0.5683 | 0.0409 |
| 192 | RP11-10C24.2 | 0.5681 | 0.0378 |
| 193 | COL15A1 | 0.5679 | 0.0418 |
| 194 | BTF3P6 | 0.5666 | 0.0206 |
| 195 | AP000692.10 | 0.5665 | 0.0382 |
| 196 | GIPC2 | 0.5635 | 0.0294 |
| 197 | RP11-169K16.8 | 0.5631 | 0.0279 |
| 198 | LINC00877 | 0.5623 | 0.0028 |
| 199 | FBXO40 | 0.5622 | 0.0139 |
| 200 | CLEC4F | 0.5608 | 0.0353 |
| 201 | GPR182 | 0.5595 | 0.0291 |
| 202 | 5S_rRNA | 0.5595 | 0.0427 |
| 203 | MIR4530 | 0.5584 | 0.0270 |
| 204 | RP11-575A19.2 | 0.5582 | 0.0439 |
| 205 | NBPF4 | 0.5576 | 0.0347 |
| 206 | RP11-494O16.3 | 0.5564 | 0.0457 |
| 207 | LDLRAD3 | 0.5559 | 0.0083 |
| 208 | SPACA6P | 0.5549 | 0.0075 |
| 209 | CTD-2562J15.6 | 0.5545 | 0.0467 |
| 210 | CTD-3049M7.1 | 0.5544 | 0.0438 |
| 211 | RP11-133O22.6 | 0.5544 | 0.0367 |
| 212 | ADCY6 | 0.5540 | 0.0197 |
| 213 | RP11-330O11.3 | 0.5539 | 0.0142 |
| 214 | RP11-156E8.1 | 0.5538 | 0.0098 |
| 215 | AL807752.1 | 0.5528 | 0.0440 |
| 216 | CLEC1A | 0.5524 | 0.0048 |
| 217 | RP11-129K12.1 | 0.5519 | 0.0307 |
| 218 | ANKUB1 | 0.5518 | 0.0423 |
| 219 | OAF | 0.5513 | 0.0023 |
| 220 | PEBP4 | 0.5497 | 0.0117 |
| 221 | RP11-69L16.4 | 0.5488 | 0.0427 |
| 222 | POU5F1P3 | 0.5488 | 0.0029 |
| 223 | RP11-414J4.2 | 0.5483 | 0.0494 |
| 224 | RP1-7G5.6 | 0.5462 | 0.0451 |
| 225 | PMP22 | 0.5458 | 0.0137 |
| 226 | CTD-2134A5.4 | 0.5453 | 0.0402 |
| 227 | HLA-DRB6 | 0.5449 | 0.0260 |
| 228 | CEBPD | 0.5448 | 0.0017 |
| 229 | DBNDD2 | 0.5441 | 0.0162 |
| 230 | CCDC135 | 0.5434 | 0.0357 |
| 231 | ZFP36 | 0.5427 | 0.0021 |
| 232 | BLVRA | 0.5425 | 0.0000 |
| 233 | RN7SL566P | 0.5424 | 0.0418 |
| 234 | DNAH2 | 0.5418 | 0.0369 |
| 235 | C1orf100 | 0.5413 | 0.0313 |
| 236 | RNU6-1280P | 0.5412 | 0.0489 |
| 237 | RP11-343N15.1 | 0.5410 | 0.0192 |
| 238 | RP11-800A3.4 | 0.5409 | 0.0024 |
| 239 | NEURL3 | 0.5408 | 0.0314 |
| 240 | UBE2Q1-AS1 | 0.5408 | 0.0304 |
| 241 | UBE2FP1 | 0.5397 | 0.0141 |
| 242 | MFI2 | 0.5392 | 0.0056 |
| 243 | AC008984.2 | 0.5391 | 0.0316 |
| 244 | RP11-511I2.2 | 0.5391 | 0.0076 |
| 245 | RP11-382A20.4 | 0.5385 | 0.0283 |
| 246 | RP11-298J23.5 | 0.5379 | 0.0371 |
| 247 | RIBC2 | 0.5368 | 0.0187 |
| 248 | RP11-6N17.2 | 0.5362 | 0.0362 |
| 249 | RP11-51F16.5 | 0.5360 | 0.0303 |
| 250 | TMEM45A | 0.5357 | 0.0354 |
| 251 | HSPB3 | 0.5355 | 0.0223 |
| 252 | SNORD46 | 0.5355 | 0.0443 |
| 253 | RP11-390P2.4 | 0.5352 | 0.0292 |
| 254 | RP11-225H22.7 | 0.5350 | 0.0347 |
| 255 | RP11-363E6.3 | 0.5337 | 0.0347 |
| 256 | BAALC | 0.5337 | 0.0381 |
| 257 | RP11-497H16.4 | 0.5335 | 0.0454 |
| 258 | IFI30 | 0.5331 | 0.0244 |
| 259 | SPRY1 | 0.5330 | 0.0119 |
| 260 | GCSHP4 | 0.5322 | 0.0366 |
| 261 | C21orf128 | 0.5322 | 0.0380 |
| 262 | RP11-818O24.3 | 0.5318 | 0.0431 |
| 263 | SPDEF | 0.5317 | 0.0431 |
| 264 | AC018470.4 | 0.5314 | 0.0404 |
| 265 | RP11-271M24.2 | 0.5310 | 0.0157 |
| 266 | ADORA3 | 0.5308 | 0.0114 |
| 267 | CD1A | 0.5294 | 0.0097 |
| 268 | LGALS9 | 0.5293 | 0.0001 |
| 269 | SLC12A5 | 0.5278 | 0.0390 |
| 270 | RGL1 | 0.5278 | 0.0008 |
| 271 | APOF | 0.5276 | 0.0276 |
| 272 | LINC00622 | 0.5276 | 0.0279 |
| 273 | CYP2T2P | 0.5263 | 0.0191 |
| 274 | AXDND1 | 0.5261 | 0.0298 |
| 275 | AC011747.4 | 0.5249 | 0.0499 |
| 276 | SAPCD1 | 0.5248 | 0.0054 |
| 277 | CTA-357J21.1 | 0.5246 | 0.0454 |
| 278 | RP5-1022P6.3 | 0.5238 | 0.0125 |
| 279 | HLA-DPA1 | 0.5238 | 0.0152 |
| 280 | AC012065.7 | 0.5230 | 0.0186 |
| 281 | ZNF385A | 0.5229 | 0.0014 |
| 282 | TXNDC5 | 0.5222 | 0.0416 |
| 283 | FAM83A | 0.5220 | 0.0485 |
| 284 | XCR1 | 0.5208 | 0.0237 |
| 285 | CRHBP | 0.5206 | 0.0272 |
| 286 | SLC6A13 | 0.5206 | 0.0475 |
| 287 | ZMYND15 | 0.5197 | 0.0090 |
| 288 | CYP4F11 | 0.5158 | 0.0324 |
| 289 | LOXL3 | 0.5157 | 0.0001 |
| 290 | RP11-265P11.2 | 0.5147 | 0.0260 |
| 291 | CXXC4 | 0.5124 | 0.0284 |
| 292 | AL645728.1 | 0.5123 | 0.0345 |
| 293 | AP003733.1 | 0.5112 | 0.0454 |
| 294 | RP11-432I5.8 | 0.5094 | 0.0349 |
| 295 | SNORD3B-1 | 0.5091 | 0.0358 |
| 296 | ICAM5 | 0.5089 | 0.0158 |
| 297 | DPRXP4 | 0.5088 | 0.0272 |
| 298 | CDC20 | 0.5088 | 0.0475 |
| 299 | CSF1R | 0.5077 | 0.0111 |
| 300 | FAM110C | 0.5069 | 0.0280 |
| 301 | LGALS2 | 0.5050 | 0.0144 |
| 302 | MS4A4A | 0.5046 | 0.0264 |
| 303 | RNU5E-4P | 0.5045 | 0.0396 |
| 304 | CST3 | 0.5044 | 0.0036 |
| 305 | RP11-113K21.4 | 0.5039 | 0.0482 |
| 306 | ZNF703 | 0.5021 | 0.0063 |
| 307 | TGFBI | 0.5013 | 0.0085 |
| 308 | AIFM3 | 0.5008 | 0.0135 |
| 309 | SDHDP2 | 0.5004 | 0.0438 |
| 310 | EMR1 | 0.4996 | 0.0007 |
| 311 | CYBRD1 | 0.4970 | 0.0051 |
| 312 | CCL24 | 0.4951 | 0.0115 |
| 313 | TMPRSS4-AS1 | 0.4949 | 0.0205 |
| 314 | EGFL6 | 0.4949 | 0.0247 |
| 315 | FNDC5 | 0.4945 | 0.0421 |
| 316 | AC079767.4 | 0.4944 | 0.0359 |
| 317 | IGLVI-63 | 0.4927 | 0.0356 |
| 318 | RP11-61L19.2 | 0.4918 | 0.0404 |
| 319 | GPAT2 | 0.4916 | 0.0181 |
| 320 | RP11-487E1.2 | 0.4906 | 0.0320 |
| 321 | AC022532.1 | 0.4905 | 0.0433 |
| 322 | CTC-508F8.1 | 0.4889 | 0.0277 |
| 323 | NEK2 | 0.4885 | 0.0396 |
| 324 | PIP5K1B | 0.4883 | 0.0018 |
| 325 | CEACAM22P | 0.4871 | 0.0385 |
| 326 | RAB44 | 0.4869 | 0.0181 |
| 327 | RP5-1042I8.7 | 0.4865 | 0.0113 |
| 328 | TNS3 | 0.4863 | 0.0071 |
| 329 | SULT1A1 | 0.4862 | 0.0032 |
| 330 | RP4-752I6.1 | 0.4851 | 0.0330 |
| 331 | VSIG10L | 0.4846 | 0.0281 |
| 332 | ZEB2-AS1 | 0.4846 | 0.0321 |
| 333 | CD300LF | 0.4844 | 0.0006 |
| 334 | AC016753.7 | 0.4837 | 0.0335 |
| 335 | RP11-680H20.2 | 0.4834 | 0.0400 |
| 336 | RP11-536K7.3 | 0.4832 | 0.0041 |
| 337 | MROH6 | 0.4824 | 0.0134 |
| 338 | AC016734.2 | 0.4816 | 0.0016 |
| 339 | MYBPHL | 0.4815 | 0.0472 |
| 340 | RPL26P30 | 0.4809 | 0.0014 |
| 341 | IQCD | 0.4802 | 0.0496 |
| 342 | HMOX1 | 0.4796 | 0.0040 |
| 343 | SCARB1 | 0.4789 | 0.0010 |
| 344 | RP6-33F8.1 | 0.4786 | 0.0374 |
| 345 | RP11-380I10.4 | 0.4786 | 0.0314 |
| 346 | AP001007.1 | 0.4774 | 0.0089 |
| 347 | TEKT1 | 0.4772 | 0.0450 |
| 348 | GPR124 | 0.4768 | 0.0497 |
| 349 | PEBP1P3 | 0.4767 | 0.0158 |
| 350 | RNU6-878P | 0.4767 | 0.0486 |
| 351 | CD14 | 0.4766 | 0.0170 |
| 352 | TNS1 | 0.4763 | 0.0202 |
| 353 | LINC00222 | 0.4758 | 0.0481 |
| 354 | RNU5A-1 | 0.4745 | 0.0353 |
| 355 | PLK2 | 0.4731 | 0.0420 |
| 356 | RDH16 | 0.4728 | 0.0061 |
| 357 | PGM5 | 0.4727 | 0.0433 |
| 358 | LARGE | 0.4718 | 0.0279 |
| 359 | RP11-672A2.4 | 0.4692 | 0.0292 |
| 360 | P2RY2 | 0.4689 | 0.0188 |
| 361 | TMEM144 | 0.4675 | 0.0299 |
| 362 | SIGLEC18P | 0.4672 | 0.0452 |
| 363 | PODN | 0.4671 | 0.0281 |
| 364 | RP11-280G9.1 | 0.4660 | 0.0362 |
| 365 | SETD9 | 0.4635 | 0.0160 |
| 366 | MRPS18CP4 | 0.4635 | 0.0347 |
| 367 | LILRA5 | 0.4624 | 0.0048 |
| 368 | KCNMB1 | 0.4622 | 0.0039 |
| 369 | SLC31A2 | 0.4596 | 0.0151 |
| 370 | SYT17 | 0.4594 | 0.0224 |
| 371 | CTD-2186M15.3 | 0.4588 | 0.0179 |
| 372 | RP11-356N1.2 | 0.4554 | 0.0392 |
| 373 | PPP1R26 | 0.4554 | 0.0007 |
| 374 | JUNB | 0.4544 | 0.0405 |
| 375 | CA8 | 0.4532 | 0.0346 |
| 376 | TCTEX1D4 | 0.4531 | 0.0409 |
| 377 | CTIF | 0.4522 | 0.0007 |
| 378 | NRP2 | 0.4509 | 0.0401 |
| 379 | TRIM16L | 0.4505 | 0.0080 |
| 380 | RPL39L | 0.4501 | 0.0418 |
| 381 | ABHD12B | 0.4501 | 0.0196 |
| 382 | PADI2 | 0.4485 | 0.0477 |
| 383 | BATF3 | 0.4484 | 0.0458 |
| 384 | SLC24A4 | 0.4471 | 0.0405 |
| 385 | DUSP1 | 0.4469 | 0.0369 |
| 386 | DCLRE1CP1 | 0.4460 | 0.0427 |
| 387 | LRP1 | 0.4453 | 0.0315 |
| 388 | HMGB3 | 0.4447 | 0.0041 |
| 389 | SLC29A1 | 0.4445 | 0.0166 |
| 390 | NPM1P25 | 0.4443 | 0.0219 |
| 391 | RP11-8L8.2 | 0.4438 | 0.0203 |
| 392 | FAM72B | 0.4417 | 0.0202 |
| 393 | NEUROD2 | 0.4416 | 0.0412 |
| 394 | SUOX | 0.4411 | 0.0014 |
| 395 | CLDN7 | 0.4410 | 0.0040 |
| 396 | FAM26F | 0.4403 | 0.0367 |
| 397 | RP11-109G23.3 | 0.4393 | 0.0422 |
| 398 | PVR | 0.4391 | 0.0120 |
| 399 | CARD9 | 0.4390 | 0.0153 |
| 400 | RPP25 | 0.4372 | 0.0094 |
| 401 | KLF11 | 0.4370 | 0.0192 |
| 402 | ARL4D | 0.4357 | 0.0493 |
| 403 | OPHN1 | 0.4354 | 0.0263 |
| 404 | GLTPD2 | 0.4340 | 0.0184 |
| 405 | FBP1 | 0.4320 | 0.0041 |
| 406 | LILRB4 | 0.4313 | 0.0228 |
| 407 | INSR | 0.4307 | 0.0134 |
| 408 | ARHGAP22 | 0.4302 | 0.0394 |
| 409 | IGFBP7 | 0.4297 | 0.0027 |
| 410 | EIF4EBP1 | 0.4295 | 0.0061 |
| 411 | RP11-568K15.1 | 0.4285 | 0.0097 |
| 412 | AP001257.1 | 0.4274 | 0.0459 |
| 413 | GATA1 | 0.4269 | 0.0496 |
| 414 | ASAP2 | 0.4262 | 0.0191 |
| 415 | CIRBP-AS1 | 0.4257 | 0.0408 |
| 416 | SIGLEC15 | 0.4237 | 0.0457 |
| 417 | HSPA7 | 0.4237 | 0.0484 |
| 418 | PLXND1 | 0.4235 | 0.0012 |
| 419 | SAMD4A | 0.4231 | 0.0455 |
| 420 | MYADM | 0.4226 | 0.0043 |
| 421 | CD1D | 0.4219 | 0.0299 |
| 422 | FLJ27365 | 0.4218 | 0.0243 |
| 423 | CD300C | 0.4217 | 0.0132 |
| 424 | SRC | 0.4204 | 0.0003 |
| 425 | UBQLN4 | 0.4203 | 0.0143 |
| 426 | EAF2 | 0.4201 | 0.0026 |
| 427 | OXTR | 0.4201 | 0.0342 |
| 428 | SATL1 | 0.4196 | 0.0037 |
| 429 | CKS1B | 0.4191 | 0.0002 |
| 430 | TTYH3 | 0.4188 | 0.0106 |
| 431 | TBC1D8 | 0.4188 | 0.0197 |
| 432 | HERC2P9 | 0.4174 | 0.0033 |
| 433 | ALG14 | 0.4172 | 0.0009 |
| 434 | CPVL | 0.4169 | 0.0295 |
| 435 | RP11-597D13.9 | 0.4164 | 0.0476 |
| 436 | KIF23 | 0.4163 | 0.0252 |
| 437 | PIK3R6 | 0.4159 | 0.0047 |
| 438 | SLC7A7 | 0.4154 | 0.0158 |
| 439 | ARSD | 0.4150 | 0.0003 |
| 440 | MMP14 | 0.4150 | 0.0305 |
| 441 | RP11-173B14.4 | 0.4148 | 0.0340 |
| 442 | VDR | 0.4146 | 0.0136 |
| 443 | ARHGEF40 | 0.4144 | 0.0256 |
| 444 | RPL7AP11 | 0.4127 | 0.0160 |
| 445 | ASAP3 | 0.4120 | 0.0330 |
| 446 | KCNC3 | 0.4119 | 0.0357 |
| 447 | PNOC | 0.4119 | 0.0373 |
| 448 | C4orf48 | 0.4115 | 0.0177 |
| 449 | MRAS | 0.4115 | 0.0246 |
| 450 | NRROS | 0.4114 | 0.0017 |
| 451 | FLVCR2 | 0.4106 | 0.0088 |
| 452 | SSPO | 0.4095 | 0.0182 |
| 453 | ANXA2P2 | 0.4092 | 0.0027 |
| 454 | NPIPB5 | 0.4086 | 0.0250 |
| 455 | RP5-827C21.4 | 0.4079 | 0.0201 |
| 456 | RP11-61J19.4 | 0.4076 | 0.0443 |
| 457 | TNFSF13 | 0.4072 | 0.0440 |
| 458 | C20orf27 | 0.4070 | 0.0065 |
| 459 | HGF | 0.4068 | 0.0318 |
| 460 | C10orf25 | 0.4064 | 0.0304 |
| 461 | MRPS18A | 0.4061 | 0.0035 |
| 462 | RP11-96D1.10 | 0.4056 | 0.0483 |
| 463 | TPPP | 0.4056 | 0.0090 |
| 464 | HIST1H2BD | 0.4047 | 0.0456 |
| 465 | CADM1 | 0.4046 | 0.0354 |
| 466 | GOLM1 | 0.4041 | 0.0059 |
| 467 | CD300E | 0.4041 | 0.0221 |
| 468 | FZD1 | 0.4028 | 0.0282 |
| 469 | RP11-686D22.8 | 0.4010 | 0.0279 |
| 470 | LILRA1 | 0.4010 | 0.0399 |
| 471 | RNF112 | 0.4003 | 0.0245 |
| 472 | AC008132.12 | 0.4000 | 0.0469 |
| 473 | TUBA1C | 0.3988 | 0.0166 |
| 474 | DAPK1 | 0.3987 | 0.0271 |
| 475 | GRN | 0.3968 | 0.0298 |
| 476 | PDIA5 | 0.3966 | 0.0016 |
| 477 | ALDH3B1 | 0.3958 | 0.0223 |
| 478 | CCDC149 | 0.3950 | 0.0275 |
| 479 | SH3RF1 | 0.3950 | 0.0235 |
| 480 | NLRP3 | 0.3950 | 0.0260 |
| 481 | LAMB2 | 0.3922 | 0.0368 |
| 482 | ATP9A | 0.3920 | 0.0482 |
| 483 | RP11-293M10.6 | 0.3916 | 0.0411 |
| 484 | ASGR2 | 0.3902 | 0.0488 |
| 485 | NBPF10 | 0.3902 | 0.0013 |
| 486 | APLP2 | 0.3901 | 0.0137 |
| 487 | JUP | 0.3901 | 0.0335 |
| 488 | MFSD7 | 0.3897 | 0.0179 |
| 489 | ETS2 | 0.3894 | 0.0102 |
| 490 | RP11-145M9.4 | 0.3854 | 0.0323 |
| 491 | MGST1 | 0.3850 | 0.0323 |
| 492 | TMEM14C | 0.3849 | 0.0015 |
| 493 | LMNA | 0.3838 | 0.0476 |
| 494 | SMPDL3A | 0.3826 | 0.0345 |
| 495 | CAPG | 0.3825 | 0.0160 |
| 496 | RAPGEF3 | 0.3824 | 0.0431 |
| 497 | ARG2 | 0.3816 | 0.0433 |
| 498 | IER5L | 0.3815 | 0.0323 |
| 499 | ARHGEF10L | 0.3805 | 0.0490 |
| 500 | FGL1 | 0.3799 | 0.0393 |
| 501 | NAAA | 0.3797 | 0.0001 |
| 502 | SERAC1 | 0.3796 | 0.0026 |
| 503 | DAB2 | 0.3791 | 0.0120 |
| 504 | ADAP2 | 0.3784 | 0.0187 |
| 505 | WARS | 0.3774 | 0.0079 |
| 506 | CD1C | 0.3769 | 0.0256 |
| 507 | NCEH1 | 0.3763 | 0.0179 |
| 508 | EMILIN2 | 0.3751 | 0.0348 |
| 509 | SLC2A6 | 0.3743 | 0.0448 |
| 510 | PPIB | 0.3740 | 0.0002 |
| 511 | RPL7AP30 | 0.3739 | 0.0376 |
| 512 | METRNL | 0.3728 | 0.0151 |
| 513 | SMCO4 | 0.3721 | 0.0216 |
| 514 | SIDT2 | 0.3717 | 0.0124 |
| 515 | C10orf105 | 0.3710 | 0.0441 |
| 516 | TGM2 | 0.3710 | 0.0406 |
| 517 | BLVRB | 0.3694 | 0.0277 |
| 518 | PROCR | 0.3685 | 0.0328 |
| 519 | RP13-104F24.3 | 0.3682 | 0.0021 |
| 520 | HPSE | 0.3680 | 0.0248 |
| 521 | IQSEC2 | 0.3673 | 0.0294 |
| 522 | TP53I3 | 0.3672 | 0.0064 |
| 523 | MARCKS | 0.3669 | 0.0159 |
| 524 | CFP | 0.3655 | 0.0214 |
| 525 | SLC2A5 | 0.3642 | 0.0297 |
| 526 | CITED2 | 0.3641 | 0.0010 |
| 527 | TNNI2 | 0.3636 | 0.0366 |
| 528 | SLC15A3 | 0.3636 | 0.0161 |
| 529 | DPYSL2 | 0.3633 | 0.0087 |
| 530 | ODF3B | 0.3631 | 0.0407 |
| 531 | RASL11A | 0.3629 | 0.0129 |
| 532 | STARD8 | 0.3624 | 0.0334 |
| 533 | RP11-78O7.2 | 0.3613 | 0.0125 |
| 534 | RP11-587D21.1 | 0.3608 | 0.0401 |
| 535 | FTL | 0.3605 | 0.0033 |
| 536 | EEF1A1P13 | 0.3605 | 0.0257 |
| 537 | TCF7L2 | 0.3592 | 0.0259 |
| 538 | PSAP | 0.3577 | 0.0248 |
| 539 | UNC93B1 | 0.3575 | 0.0105 |
| 540 | UQCRQ | 0.3568 | 0.0190 |
| 541 | MICALCL | 0.3556 | 0.0459 |
| 542 | CECR1 | 0.3554 | 0.0010 |
| 543 | FSTL3 | 0.3554 | 0.0070 |
| 544 | OSCAR | 0.3551 | 0.0206 |
| 545 | CXCL16 | 0.3540 | 0.0398 |
| 546 | CSTA | 0.3536 | 0.0270 |
| 547 | RP11-65J3.1 | 0.3536 | 0.0484 |
| 548 | PSMD10P1 | 0.3536 | 0.0443 |
| 549 | ARRB1 | 0.3535 | 0.0127 |
| 550 | S100A10 | 0.3528 | 0.0059 |
| 551 | PCTP | 0.3523 | 0.0135 |
| 552 | SLC31A1 | 0.3518 | 0.0093 |
| 553 | SCO2 | 0.3516 | 0.0446 |
| 554 | ARSB | 0.3513 | 0.0294 |
| 555 | CDKN3 | 0.3509 | 0.0355 |
| 556 | TRBV5-1 | 0.3494 | 0.0420 |
| 557 | RASSF4 | 0.3491 | 0.0452 |
| 558 | RP11-162A12.2 | 0.3476 | 0.0289 |
| 559 | AFMID | 0.3465 | 0.0332 |
| 560 | MTMR11 | 0.3461 | 0.0440 |
| 561 | PRPS1P2 | 0.3461 | 0.0438 |
| 562 | CTSB | 0.3456 | 0.0073 |
| 563 | SLC43A3 | 0.3453 | 0.0059 |
| 564 | LGALS3 | 0.3451 | 0.0270 |
| 565 | LY6E | 0.3451 | 0.0468 |
| 566 | BFSP1 | 0.3450 | 0.0335 |
| 567 | RNF144B | 0.3442 | 0.0254 |
| 568 | PNKD | 0.3432 | 0.0092 |
| 569 | GNA15 | 0.3428 | 0.0282 |
| 570 | GAS7 | 0.3426 | 0.0238 |
| 571 | CD79B | 0.3424 | 0.0084 |
| 572 | AIF1 | 0.3423 | 0.0183 |
| 573 | C10orf128 | 0.3408 | 0.0369 |
| 574 | LINC00888 | 0.3406 | 0.0078 |
| 575 | YRDC | 0.3404 | 0.0403 |
| 576 | RP11-38P22.2 | 0.3401 | 0.0345 |
| 577 | ABHD11 | 0.3395 | 0.0008 |
| 578 | PC | 0.3392 | 0.0447 |
| 579 | SPINT1 | 0.3373 | 0.0345 |
| 580 | P2RX1 | 0.3371 | 0.0109 |
| 581 | NPHP4 | 0.3366 | 0.0051 |
| 582 | STAC3 | 0.3358 | 0.0373 |
| 583 | FAM129B | 0.3358 | 0.0479 |
| 584 | PPFIBP2 | 0.3357 | 0.0209 |
| 585 | PTPN2P1 | 0.3353 | 0.0470 |
| 586 | NAGA | 0.3348 | 0.0259 |
| 587 | ZDHHC12 | 0.3342 | 0.0031 |
| 588 | PLK1 | 0.3336 | 0.0458 |
| 589 | ADRBK2 | 0.3326 | 0.0331 |
| 590 | TOR2A | 0.3323 | 0.0013 |
| 591 | COTL1 | 0.3319 | 0.0045 |
| 592 | TREX1 | 0.3318 | 0.0071 |
| 593 | CSTB | 0.3318 | 0.0033 |
| 594 | NPC2 | 0.3314 | 0.0260 |
| 595 | ANXA2 | 0.3311 | 0.0128 |
| 596 | UBTD1 | 0.3311 | 0.0136 |
| 597 | C19orf54 | 0.3304 | 0.0110 |
| 598 | VAT1 | 0.3279 | 0.0015 |
| 599 | CHKA | 0.3277 | 0.0319 |
| 600 | RP11-680G24.4 | 0.3276 | 0.0499 |
| 601 | MOB3B | 0.3240 | 0.0262 |
| 602 | C7orf31 | 0.3239 | 0.0029 |
| 603 | TXNDC17 | 0.3230 | 0.0030 |
| 604 | PEX7 | 0.3227 | 0.0216 |
| 605 | HLA-DRA | 0.3214 | 0.0324 |
| 606 | IER5 | 0.3210 | 0.0441 |
| 607 | PRAM1 | 0.3202 | 0.0239 |
| 608 | AGAP1 | 0.3173 | 0.0459 |
| 609 | SLC2A8 | 0.3164 | 0.0062 |
| 610 | RP11-169K16.7 | 0.3163 | 0.0236 |
| 611 | BTBD3 | 0.3158 | 0.0378 |
| 612 | C19orf38 | 0.3155 | 0.0286 |
| 613 | IRF4 | 0.3155 | 0.0130 |
| 614 | RP11-622K12.1 | 0.3154 | 0.0132 |
| 615 | MTX1P1 | 0.3148 | 0.0417 |
| 616 | HLA-DMB | 0.3144 | 0.0139 |
| 617 | ATF5 | 0.3132 | 0.0261 |
| 618 | LHPP | 0.3126 | 0.0003 |
| 619 | GRINA | 0.3121 | 0.0248 |
| 620 | CLEC11A | 0.3114 | 0.0416 |
| 621 | SLC43A2 | 0.3099 | 0.0450 |
| 622 | FES | 0.3092 | 0.0454 |
| 623 | BTK | 0.3091 | 0.0189 |
| 624 | VIM | 0.3087 | 0.0244 |
| 625 | CREG1 | 0.3079 | 0.0427 |
| 626 | REEP4 | 0.3075 | 0.0083 |
| 627 | RP11-67L2.2 | 0.3073 | 0.0024 |
| 628 | ANXA5 | 0.3057 | 0.0104 |
| 629 | GAPDH | 0.3054 | 0.0100 |
| 630 | MPHOSPH6 | 0.3050 | 0.0377 |
| 631 | FGR | 0.3049 | 0.0086 |
| 632 | GLB1 | 0.3043 | 0.0139 |
| 633 | WASH6P | 0.3041 | 0.0217 |
| 634 | C19orf10 | 0.3028 | 0.0046 |
| 635 | KDM1B | 0.3019 | 0.0445 |
| 636 | SLC29A3 | 0.3015 | 0.0416 |
| 637 | RP11-4O1.2 | 0.3013 | 0.0299 |
| 638 | CDKN2C | 0.3008 | 0.0121 |
| 639 | CRIP1 | 0.3001 | 0.0320 |
| 640 | AC069282.6 | 0.2980 | 0.0331 |
| 641 | ADAM15 | 0.2973 | 0.0223 |
| 642 | PRELID1 | 0.2969 | 0.0032 |
| 643 | TIMM8B | 0.2964 | 0.0233 |
| 644 | SPHK1 | 0.2950 | 0.0197 |
| 645 | EIF4A1P2 | 0.2949 | 0.0151 |
| 646 | RAB24 | 0.2948 | 0.0382 |
| 647 | PRDX1 | 0.2937 | 0.0010 |
| 648 | TNFRSF1B | 0.2930 | 0.0044 |
| 649 | CORO1C | 0.2929 | 0.0239 |
| 650 | CTD-2545M3.8 | 0.2929 | 0.0341 |
| 651 | GABARAP | 0.2929 | 0.0281 |
| 652 | RNH1 | 0.2926 | 0.0062 |
| 653 | PLOD1 | 0.2914 | 0.0404 |
| 654 | PRCP | 0.2910 | 0.0142 |
| 655 | C9orf89 | 0.2906 | 0.0241 |
| 656 | HIST3H2A | 0.2904 | 0.0465 |
| 657 | CD4 | 0.2899 | 0.0294 |
| 658 | NR1H3 | 0.2897 | 0.0075 |
| 659 | SNAI3-AS1 | 0.2896 | 0.0315 |
| 660 | CCRL2 | 0.2894 | 0.0358 |
| 661 | KIAA0930 | 0.2892 | 0.0227 |
| 662 | KIAA0513 | 0.2891 | 0.0187 |
| 663 | SLC9A9 | 0.2887 | 0.0058 |
| 664 | TLR5 | 0.2878 | 0.0021 |
| 665 | RP11-36C20.1 | 0.2875 | 0.0108 |
| 666 | RNF135 | 0.2872 | 0.0115 |
| 667 | SRD5A3 | 0.2871 | 0.0442 |
| 668 | ARHGAP32 | 0.2868 | 0.0401 |
| 669 | SUMF1 | 0.2865 | 0.0120 |
| 670 | SLC38A7 | 0.2863 | 0.0117 |
| 671 | AGTRAP | 0.2860 | 0.0078 |
| 672 | RXRA | 0.2851 | 0.0459 |
| 673 | PTK2 | 0.2851 | 0.0500 |
| 674 | SRGAP2 | 0.2850 | 0.0031 |
| 675 | ZMIZ1 | 0.2849 | 0.0277 |
| 676 | IMP3 | 0.2847 | 0.0111 |
| 677 | PIWIL4 | 0.2847 | 0.0301 |
| 678 | CTNND1 | 0.2845 | 0.0271 |
| 679 | TMEM80 | 0.2841 | 0.0117 |
| 680 | TMEM180 | 0.2833 | 0.0160 |
| 681 | BTF3L4P2 | 0.2830 | 0.0166 |
| 682 | SEMA4A | 0.2830 | 0.0415 |
| 683 | ZDHHC7 | 0.2822 | 0.0099 |
| 684 | TKT | 0.2819 | 0.0343 |
| 685 | CTA-217C2.1 | 0.2818 | 0.0210 |
| 686 | HSD17B10 | 0.2810 | 0.0132 |
| 687 | DENND6B | 0.2810 | 0.0359 |
| 688 | MTSS1 | 0.2806 | 0.0134 |
| 689 | CUEDC1 | 0.2800 | 0.0486 |
| 690 | UBXN11 | 0.2793 | 0.0249 |
| 691 | SYNGR2 | 0.2779 | 0.0076 |
| 692 | CMTM7 | 0.2775 | 0.0265 |
| 693 | PDE4A | 0.2773 | 0.0247 |
| 694 | PLBD2 | 0.2771 | 0.0401 |
| 695 | LYL1 | 0.2758 | 0.0411 |
| 696 | C1orf162 | 0.2748 | 0.0224 |
| 697 | NDUFS4 | 0.2745 | 0.0125 |
| 698 | ACSS2 | 0.2745 | 0.0351 |
| 699 | RAD51C | 0.2737 | 0.0473 |
| 700 | MPDU1 | 0.2735 | 0.0074 |
| 701 | TM9SF4 | 0.2732 | 0.0065 |
| 702 | LINC00341 | 0.2726 | 0.0142 |
| 703 | TUBB4B | 0.2718 | 0.0359 |
| 704 | SLC3A2 | 0.2712 | 0.0048 |
| 705 | KCNQ1 | 0.2711 | 0.0217 |
| 706 | PPP1R32 | 0.2700 | 0.0312 |
| 707 | DNMBP | 0.2697 | 0.0049 |
| 708 | SOX4 | 0.2696 | 0.0424 |
| 709 | CLCF1 | 0.2683 | 0.0102 |
| 710 | LDHA | 0.2680 | 0.0007 |
| 711 | UQCRFS1P1 | 0.2676 | 0.0175 |
| 712 | PELI3 | 0.2660 | 0.0492 |
| 713 | ASAH1 | 0.2659 | 0.0287 |
| 714 | MFSD5 | 0.2658 | 0.0212 |
| 715 | FRMD4B | 0.2658 | 0.0468 |
| 716 | SNX21 | 0.2653 | 0.0430 |
| 717 | BCKDK | 0.2650 | 0.0358 |
| 718 | PLP2 | 0.2648 | 0.0342 |
| 719 | GSTZ1 | 0.2647 | 0.0459 |
| 720 | C15orf52 | 0.2644 | 0.0068 |
| 721 | GALK1 | 0.2638 | 0.0209 |
| 722 | FAM127A | 0.2635 | 0.0117 |
| 723 | TSPO | 0.2635 | 0.0462 |
| 724 | RP11-543P15.1 | 0.2633 | 0.0292 |

**Supplementary Table 2. Downregulated genes in PBMCs from PPD patients.**

|  | **Gene** | **log2 Fold Change** | **p value** |
| --- | --- | --- | --- |
| 1 | PAH | -4.8383 | 1.3711E-94 |
| 2 | EPCAM | -4.2202 | 2.6029E-70 |
| 3 | ABCC8 | -3.3612 | 2.2943E-37 |
| 4 | F11 | -3.3232 | 2.0493E-41 |
| 5 | CDH1 | -3.2424 | 3.3347E-72 |
| 6 | BRCA2 | -3.1903 | 1.1867E-123 |
| 7 | CHRNA3 | -3.1496 | 1.3541E-33 |
| 8 | MUC2 | -2.9889 | 1.2778E-28 |
| 9 | SLC26A4 | -2.9164 | 1.3857E-39 |
| 10 | ADRB3 | -2.8836 | 1.2592E-26 |
| 11 | EFS | -2.7568 | 4.6158E-24 |
| 12 | PRM1 | -2.7029 | 4.7228E-23 |
| 13 | OR11H7 | -2.6199 | 4.3399E-25 |
| 14 | ABCB11 | -2.5979 | 5.1697E-22 |
| 15 | LPA | -2.4888 | 2.0481E-19 |
| 16 | AP000695.4 | -2.4750 | 6.8708E-20 |
| 17 | TAS2R38 | -2.4300 | 1.7229E-18 |
| 18 | SLC30A8 | -2.2498 | 1.4697E-16 |
| 19 | TSPYL6 | -2.2400 | 4.7964E-17 |
| 20 | SLC45A2 | -2.2289 | 5.7184E-16 |
| 21 | FGF21 | -2.0792 | 8.1918E-14 |
| 22 | UQCRC2P1 | -2.0588 | 1.5595E-13 |
| 23 | ALDOB | -1.9506 | 2.7534E-15 |
| 24 | CASC10 | -1.8574 | 2.7869E-11 |
| 25 | CDKN2A | -1.7688 | 2.5224E-41 |
| 26 | HMGA2 | -1.7582 | 5.6623E-11 |
| 27 | BRCA1 | -1.6702 | 1.4343E-31 |
| 28 | HCG9 | -1.6687 | 2.9018E-10 |
| 29 | ABCG8 | -1.6521 | 2.9834E-09 |
| 30 | IL13 | -1.6424 | 8.3113E-10 |
| 31 | PALB2 | -1.4872 | 2.4976E-63 |
| 32 | FANCC | -1.4777 | 6.5276E-37 |
| 33 | COL6A4P1 | -1.4449 | 2.1456E-07 |
| 34 | CASC16 | -1.4402 | 1.6117E-07 |
| 35 | HCRTR2 | -1.4341 | 1.7788E-07 |
| 36 | IGF2-AS | -1.3905 | 3.8029E-07 |
| 37 | APOE | -1.3493 | 1.5933E-08 |
| 38 | ADH7 | -1.3177 | 1.1748E-06 |
| 39 | KCNJ11 | -1.2933 | 2.6032E-07 |
| 40 | MIR5692C2 | -1.2726 | 4.8170E-06 |
| 41 | RNA5SP187 | -1.2487 | 4.9782E-06 |
| 42 | RP11-701H24.3 | -1.2172 | 6.5531E-07 |
| 43 | AGGF1P2 | -1.1972 | 8.7280E-06 |
| 44 | KITLG | -1.1968 | 1.6077E-06 |
| 45 | ACTN3 | -1.1950 | 9.1065E-07 |
| 46 | RP11-292F22.5 | -1.1117 | 4.7910E-05 |
| 47 | PMS2 | -1.1108 | 1.5073E-35 |
| 48 | AL021917.1 | -1.0990 | 2.8843E-05 |
| 49 | TRAJ14 | -1.0932 | 0.0001 |
| 50 | SNRPGP4 | -1.0677 | 0.0001 |
| 51 | LGSN | -1.0541 | 0.0001 |
| 52 | ALG10 | -1.0522 | 8.1287E-08 |
| 53 | RP11-115D19.1 | -1.0488 | 0.0001 |
| 54 | LRRC37A9P | -1.0392 | 0.0001 |
| 55 | XIST | -1.0250 | 1.1960E-05 |
| 56 | MSMB | -1.0172 | 0.0001 |
| 57 | TRAJ21 | -1.0055 | 0.0003 |
| 58 | CICP4 | -0.9966 | 0.0003 |
| 59 | ZNF66 | -0.9952 | 5.0919E-06 |
| 60 | ZNF774 | -0.9840 | 0.0003 |
| 61 | MTND5P28 | -0.9835 | 0.0004 |
| 62 | RP4-545K15.5 | -0.9793 | 0.0003 |
| 63 | LL0XNC01-116E7.2 | -0.9768 | 1.0891E-05 |
| 64 | ASIC1 | -0.9560 | 0.0002 |
| 65 | GLIS3 | -0.9550 | 0.0001 |
| 66 | RP11-286E11.2 | -0.9546 | 0.0006 |
| 67 | RP11-553L6.2 | -0.9515 | 7.8427E-07 |
| 68 | RP6-206I17.4 | -0.9419 | 0.0007 |
| 69 | AL590452.1 | -0.9413 | 0.0006 |
| 70 | RP11-1085N6.2 | -0.9408 | 0.0007 |
| 71 | AC097713.4 | -0.9406 | 2.8344E-07 |
| 72 | AP001434.2 | -0.9345 | 0.0008 |
| 73 | RP11-1094M14.9 | -0.9202 | 1.0677E-07 |
| 74 | RP11-17P16.2 | -0.9194 | 0.0009 |
| 75 | RP11-1094M14.4 | -0.9187 | 4.8763E-06 |
| 76 | TRAJ12 | -0.9173 | 0.0005 |
| 77 | RN7SL333P | -0.9157 | 0.0008 |
| 78 | AF131215.5 | -0.9122 | 0.0009 |
| 79 | TRAJ13 | -0.9120 | 0.0011 |
| 80 | AC034220.3 | -0.9096 | 4.0645E-07 |
| 81 | APOBEC2 | -0.9054 | 0.0001 |
| 82 | RP11-777F6.3 | -0.9024 | 0.0002 |
| 83 | RP11-359J14.2 | -0.9005 | 0.0002 |
| 84 | TCEB1P19 | -0.8997 | 0.0002 |
| 85 | SNORD64 | -0.8931 | 0.0008 |
| 86 | CTC-339F2.2 | -0.8884 | 0.0013 |
| 87 | CCDC40 | -0.8874 | 0.0006 |
| 88 | TRAJ10 | -0.8852 | 0.0012 |
| 89 | SLC16A7 | -0.8840 | 0.0001 |
| 90 | ANKRD36C | -0.8835 | 1.4099E-05 |
| 91 | RN7SL19P | -0.8808 | 0.0016 |
| 92 | EPHA1-AS1 | -0.8746 | 4.2143E-05 |
| 93 | RP11-1191J2.2 | -0.8633 | 0.0014 |
| 94 | RP11-230C9.2 | -0.8618 | 0.0013 |
| 95 | ALPK2 | -0.8598 | 0.0013 |
| 96 | FAM153C | -0.8582 | 0.0010 |
| 97 | RP11-383I23.2 | -0.8537 | 0.0015 |
| 98 | AC092580.1 | -0.8507 | 0.0022 |
| 99 | TRAJ7 | -0.8479 | 0.0007 |
| 100 | AC009299.2 | -0.8460 | 0.0005 |
| 101 | CTD-3138B18.6 | -0.8456 | 0.0024 |
| 102 | TRAJ11 | -0.8403 | 0.0023 |
| 103 | RP11-214K3.21 | -0.8401 | 0.0024 |
| 104 | TAS2R3 | -0.8393 | 0.0022 |
| 105 | RP11-452D12.1 | -0.8369 | 0.0004 |
| 106 | TRAJ6 | -0.8351 | 0.0007 |
| 107 | RN7SKP74 | -0.8338 | 0.0028 |
| 108 | RP11-797H7.1 | -0.8333 | 7.9323E-08 |
| 109 | RNU6-915P | -0.8331 | 0.0025 |
| 110 | MIR421 | -0.8331 | 0.0016 |
| 111 | RP11-214K3.22 | -0.8290 | 0.0029 |
| 112 | EDN1 | -0.8274 | 2.7210E-06 |
| 113 | RP5-888M10.2 | -0.8235 | 0.0027 |
| 114 | RIMKLBP2 | -0.8235 | 0.0001 |
| 115 | HMGB3P4 | -0.8214 | 0.0011 |
| 116 | KLHL7-AS1 | -0.8201 | 0.0033 |
| 117 | TRPA1 | -0.8196 | 0.0029 |
| 118 | AF131215.3 | -0.8186 | 0.0024 |
| 119 | C4orf50 | -0.8165 | 0.0003 |
| 120 | RP11-263K4.1 | -0.8150 | 0.0014 |
| 121 | RP11-436I9.5 | -0.8143 | 0.0004 |
| 122 | CTC-448F2.4 | -0.8141 | 0.0034 |
| 123 | CAPN8 | -0.8129 | 0.0013 |
| 124 | ZNF891 | -0.8108 | 0.0001 |
| 125 | OR10AC1P | -0.8058 | 0.0021 |
| 126 | ZNF573 | -0.8054 | 1.2037E-05 |
| 127 | RP11-214K3.19 | -0.8007 | 0.0037 |
| 128 | RP11-53B2.3 | -0.8000 | 0.0009 |
| 129 | RP11-380G5.3 | -0.7991 | 0.0022 |
| 130 | RP11-114M5.1 | -0.7978 | 0.0017 |
| 131 | RP11-325P15.1 | -0.7976 | 0.0042 |
| 132 | OR52V1P | -0.7972 | 0.0028 |
| 133 | TRDJ3 | -0.7936 | 0.0028 |
| 134 | GS1-184P14.2 | -0.7935 | 0.0037 |
| 135 | RP11-386G11.5 | -0.7902 | 0.0032 |
| 136 | Z85986.1 | -0.7889 | 0.0044 |
| 137 | DLG2 | -0.7883 | 0.0004 |
| 138 | AC024940.1 | -0.7868 | 0.0042 |
| 139 | ZNF208 | -0.7810 | 0.0036 |
| 140 | KLRC3 | -0.7777 | 0.0017 |
| 141 | RAB19 | -0.7762 | 0.0054 |
| 142 | RP11-77C3.3 | -0.7757 | 0.0003 |
| 143 | RP11-420K14.6 | -0.7756 | 0.0037 |
| 144 | VN1R83P | -0.7715 | 0.0001 |
| 145 | RP11-53B2.1 | -0.7707 | 0.0052 |
| 146 | RP1-164L12.1 | -0.7706 | 0.0039 |
| 147 | ANTXRLP1 | -0.7705 | 0.0003 |
| 148 | RP11-275I4.1 | -0.7690 | 0.0054 |
| 149 | RP11-402J7.2 | -0.7686 | 2.2244E-06 |
| 150 | RP11-797A18.3 | -0.7683 | 0.0006 |
| 151 | ALG10B | -0.7678 | 9.1433E-06 |
| 152 | ZNF460 | -0.7662 | 0.0004 |
| 153 | PHOSPHO2 | -0.7652 | 0.0008 |
| 154 | ZBED6 | -0.7629 | 0.0049 |
| 155 | RNU6-652P | -0.7629 | 0.0060 |
| 156 | SEPP1 | -0.7624 | 0.0006 |
| 157 | MIR374B | -0.7612 | 0.0063 |
| 158 | RP3-408N23.4 | -0.7595 | 0.0057 |
| 159 | SPATA20P1 | -0.7585 | 0.0066 |
| 160 | AC015987.1 | -0.7583 | 0.0026 |
| 161 | TATDN2P2 | -0.7580 | 0.0002 |
| 162 | TRAJ25 | -0.7551 | 0.0064 |
| 163 | C10orf12 | -0.7535 | 0.0003 |
| 164 | TRBV1 | -0.7523 | 0.0021 |
| 165 | MME | -0.7501 | 0.0019 |
| 166 | NBEAL1 | -0.7493 | 0.0004 |
| 167 | RNU6ATAC24P | -0.7477 | 0.0004 |
| 168 | RP11-413E1.2 | -0.7447 | 0.0071 |
| 169 | TRAJ33 | -0.7447 | 0.0066 |
| 170 | RP11-667M19.9 | -0.7445 | 0.0019 |
| 171 | C17orf50 | -0.7444 | 0.0074 |
| 172 | KRT8P33 | -0.7436 | 9.4675E-06 |
| 173 | AAK1 | -0.7433 | 2.8475E-08 |
| 174 | FAM226B | -0.7408 | 0.0050 |
| 175 | KRT18P57 | -0.7401 | 0.0072 |
| 176 | AC007556.3 | -0.7395 | 0.0080 |
| 177 | C4orf47 | -0.7394 | 0.0040 |
| 178 | RP11-638I2.2 | -0.7390 | 0.0061 |
| 179 | EBF3 | -0.7381 | 0.0077 |
| 180 | SNORA12 | -0.7349 | 0.0001 |
| 181 | RP11-697E2.9 | -0.7325 | 0.0075 |
| 182 | AC005943.5 | -0.7316 | 0.0029 |
| 183 | MCOLN3 | -0.7277 | 0.0015 |
| 184 | RNA5SP317 | -0.7267 | 0.0045 |
| 185 | RP11-632K21.6 | -0.7263 | 0.0079 |
| 186 | STXBP4 | -0.7262 | 0.0001 |
| 187 | AL049542.1 | -0.7232 | 0.0095 |
| 188 | RPS2P52 | -0.7224 | 0.0075 |
| 189 | AASS | -0.7214 | 0.0028 |
| 190 | RP1-153P14.8 | -0.7203 | 0.0023 |
| 191 | IGKV1D-16 | -0.7199 | 0.0091 |
| 192 | REL | -0.7196 | 0.0001 |
| 193 | RP11-509J21.1 | -0.7173 | 0.0008 |
| 194 | CTD-3220F14.2 | -0.7171 | 0.0052 |
| 195 | MROH5 | -0.7167 | 0.0083 |
| 196 | CTC-559E9.12 | -0.7152 | 0.0090 |
| 197 | RP11-258C19.4 | -0.7142 | 0.0002 |
| 198 | LL0XNC01-237H1.2 | -0.7140 | 0.0093 |
| 199 | RP11-210N13.1 | -0.7139 | 0.0105 |
| 200 | RP11-22P6.2 | -0.7125 | 0.0040 |
| 201 | TRGV1 | -0.7120 | 0.0077 |
| 202 | MIR580 | -0.7119 | 0.0061 |
| 203 | TRAJ27 | -0.7105 | 0.0086 |
| 204 | DNM1P47 | -0.7099 | 0.0024 |
| 205 | RP11-613C6.2 | -0.7086 | 0.0092 |
| 206 | RP11-414H23.3 | -0.7085 | 0.0111 |
| 207 | EVPL | -0.7068 | 0.0083 |
| 208 | CTD-2140B24.6 | -0.7061 | 0.0094 |
| 209 | YWHAQP6 | -0.7054 | 0.0086 |
| 210 | GRK6P1 | -0.7041 | 0.0102 |
| 211 | SIDT1-AS1 | -0.7035 | 0.0080 |
| 212 | RNU6-1215P | -0.7033 | 0.0048 |
| 213 | PHACTR2P1 | -0.7027 | 0.0113 |
| 214 | CTD-2313N18.7 | -0.7023 | 0.0108 |
| 215 | DBH | -0.7020 | 0.0003 |
| 216 | SLC9B1 | -0.7010 | 0.0002 |
| 217 | GPR150 | -0.7009 | 0.0011 |
| 218 | RP4-657D16.6 | -0.7006 | 0.0029 |
| 219 | CTD-2283N19.1 | -0.6998 | 0.0099 |
| 220 | RP11-553L6.3 | -0.6982 | 0.0010 |
| 221 | AC092338.5 | -0.6979 | 0.0022 |
| 222 | GVINP1 | -0.6977 | 0.0002 |
| 223 | AF131215.4 | -0.6972 | 0.0113 |
| 224 | RP11-677I18.3 | -0.6971 | 0.0069 |
| 225 | RP11-309L24.4 | -0.6966 | 0.0115 |
| 226 | RP11-22B10.3 | -0.6957 | 0.0061 |
| 227 | MTX3 | -0.6946 | 8.6462E-06 |
| 228 | RP11-530C5.2 | -0.6945 | 0.0127 |
| 229 | RP11-902B17.1 | -0.6943 | 1.9615E-05 |
| 230 | NDUFA3P1 | -0.6942 | 0.0121 |
| 231 | RNU6-925P | -0.6941 | 0.0107 |
| 232 | TRDJ4 | -0.6920 | 0.0122 |
| 233 | MIR663A | -0.6906 | 0.0100 |
| 234 | RP11-690I21.2 | -0.6899 | 0.0112 |
| 235 | C6orf164 | -0.6888 | 0.0029 |
| 236 | ABCC11 | -0.6877 | 0.0125 |
| 237 | RP11-702H23.2 | -0.6876 | 0.0132 |
| 238 | CTA-363E19.2 | -0.6870 | 0.0010 |
| 239 | RN7SL172P | -0.6865 | 0.0075 |
| 240 | TMEM220 | -0.6858 | 0.0001 |
| 241 | TRAJ9 | -0.6855 | 0.0086 |
| 242 | CELF2-AS1 | -0.6853 | 0.0116 |
| 243 | RP11-111E14.1 | -0.6817 | 0.0145 |
| 244 | RNU7-40P | -0.6816 | 0.0137 |
| 245 | RP3-395M20.2 | -0.6806 | 0.0119 |
| 246 | RP11-478C6.1 | -0.6787 | 0.0122 |
| 247 | TMEM178B | -0.6776 | 0.0130 |
| 248 | RP11-613F22.5 | -0.6775 | 0.0140 |
| 249 | RP11-330M2.4 | -0.6754 | 0.0091 |
| 250 | COL5A3 | -0.6751 | 0.0086 |
| 251 | RP3-368A4.6 | -0.6746 | 0.0001 |
| 252 | A2M | -0.6743 | 0.0044 |
| 253 | RNU6-100P | -0.6737 | 0.0151 |
| 254 | RP11-383G6.3 | -0.6720 | 0.0030 |
| 255 | AC079781.8 | -0.6720 | 0.0157 |
| 256 | PRR15L | -0.6706 | 0.0147 |
| 257 | GOLGA8O | -0.6705 | 0.0022 |
| 258 | SORBS2 | -0.6704 | 0.0163 |
| 259 | SIRPB3P | -0.6701 | 0.0034 |
| 260 | RP11-382A20.7 | -0.6701 | 0.0157 |
| 261 | ANKRD36 | -0.6700 | 1.7584E-05 |
| 262 | TRAJ31 | -0.6677 | 0.0129 |
| 263 | AC007690.1 | -0.6668 | 0.0029 |
| 264 | KCNA3 | -0.6667 | 0.0001 |
| 265 | RP11-197P3.4 | -0.6661 | 0.0167 |
| 266 | SDK2 | -0.6658 | 0.0001 |
| 267 | RP11-493E12.2 | -0.6658 | 0.0076 |
| 268 | PFN1P3 | -0.6634 | 0.0161 |
| 269 | RP11-535A5.1 | -0.6621 | 0.0028 |
| 270 | RP11-862L9.3 | -0.6615 | 0.0175 |
| 271 | RP11-713N11.3 | -0.6607 | 0.0171 |
| 272 | CTC-336P14.1 | -0.6604 | 0.0145 |
| 273 | SNORA77 | -0.6591 | 0.0180 |
| 274 | IQSEC3 | -0.6590 | 0.0178 |
| 275 | RP13-735L24.1 | -0.6583 | 0.0055 |
| 276 | COX6CP1 | -0.6582 | 0.0105 |
| 277 | RP11-509J21.4 | -0.6580 | 0.0039 |
| 278 | RNU6-431P | -0.6576 | 0.0184 |
| 279 | TTN | -0.6574 | 1.2009E-07 |
| 280 | UPF3AP3 | -0.6572 | 0.0170 |
| 281 | GOLGA8N | -0.6571 | 0.0054 |
| 282 | RP11-384K6.4 | -0.6568 | 0.0145 |
| 283 | TRAJ22 | -0.6568 | 0.0177 |
| 284 | HMGN1P15 | -0.6557 | 0.0139 |
| 285 | RP11-795F19.5 | -0.6552 | 0.0184 |
| 286 | RP11-348F1.3 | -0.6551 | 0.0012 |
| 287 | RP11-526I2.1 | -0.6548 | 0.0155 |
| 288 | RP4-777D9.2 | -0.6544 | 0.0134 |
| 289 | WNT8B | -0.6543 | 0.0051 |
| 290 | AC096582.7 | -0.6539 | 0.0190 |
| 291 | RP3-323N1.2 | -0.6522 | 0.0194 |
| 292 | RP11-723O4.3 | -0.6520 | 0.0144 |
| 293 | RP11-69E11.8 | -0.6518 | 0.0083 |
| 294 | OR10G2 | -0.6516 | 0.0038 |
| 295 | RNU7-189P | -0.6515 | 0.0188 |
| 296 | RP5-894A10.6 | -0.6510 | 0.0003 |
| 297 | A2MP1 | -0.6510 | 0.0039 |
| 298 | RP11-433P17.3 | -0.6507 | 0.0189 |
| 299 | ZSCAN23 | -0.6506 | 0.0157 |
| 300 | RP11-815J21.1 | -0.6473 | 0.0190 |
| 301 | TTC3P1 | -0.6461 | 1.3558E-05 |
| 302 | LINC00943 | -0.6459 | 0.0005 |
| 303 | RP11-701H24.7 | -0.6456 | 0.0081 |
| 304 | RIMBP3B | -0.6446 | 0.0177 |
| 305 | DDX50P1 | -0.6443 | 0.0005 |
| 306 | RP11-35G22.1 | -0.6437 | 0.0184 |
| 307 | ZBED2 | -0.6436 | 0.0010 |
| 308 | RP11-973N13.4 | -0.6434 | 0.0002 |
| 309 | SNORD108 | -0.6434 | 0.0140 |
| 310 | TRAJ5 | -0.6422 | 0.0078 |
| 311 | RRH | -0.6417 | 0.0214 |
| 312 | CTB-180A7.3 | -0.6415 | 0.0195 |
| 313 | AC127904.2 | -0.6410 | 0.0104 |
| 314 | MYO5B | -0.6410 | 0.0110 |
| 315 | CCDC147-AS1 | -0.6405 | 0.0071 |
| 316 | SNORD109A | -0.6404 | 0.0164 |
| 317 | RP11-218L14.4 | -0.6401 | 0.0045 |
| 318 | RP11-674I16.2 | -0.6384 | 0.0052 |
| 319 | AP001610.5 | -0.6379 | 0.0222 |
| 320 | RP11-382J24.2 | -0.6374 | 0.0209 |
| 321 | SARDH | -0.6372 | 1.1744E-05 |
| 322 | U82671.8 | -0.6368 | 0.0156 |
| 323 | RP11-166O4.6 | -0.6364 | 0.0021 |
| 324 | CICP16 | -0.6363 | 0.0038 |
| 325 | AF131215.8 | -0.6359 | 0.0185 |
| 326 | MUTYH | -0.6345 | 3.3684E-15 |
| 327 | CTD-2165H16.3 | -0.6337 | 0.0214 |
| 328 | DSTNP1 | -0.6332 | 0.0004 |
| 329 | AC092835.2 | -0.6330 | 0.0064 |
| 330 | RP11-367J7.3 | -0.6318 | 0.0131 |
| 331 | NHLRC2 | -0.6314 | 2.7832E-05 |
| 332 | APC | -0.6310 | 2.3691E-22 |
| 333 | RNU6-1016P | -0.6305 | 0.0080 |
| 334 | BTBD8 | -0.6298 | 0.0206 |
| 335 | CTC-523E23.10 | -0.6296 | 0.0237 |
| 336 | SUGT1P3 | -0.6293 | 0.0094 |
| 337 | TRAJ8 | -0.6291 | 0.0144 |
| 338 | RP11-737O24.3 | -0.6285 | 0.0063 |
| 339 | CYP3A4 | -0.6280 | 0.0194 |
| 340 | AC092798.2 | -0.6280 | 0.0223 |
| 341 | IDSP1 | -0.6279 | 0.0232 |
| 342 | AP000692.9 | -0.6277 | 0.0143 |
| 343 | AP001350.1 | -0.6266 | 0.0200 |
| 344 | RP11-254B13.3 | -0.6264 | 0.0109 |
| 345 | ZNF888 | -0.6263 | 0.0243 |
| 346 | CTD-2561J22.6 | -0.6262 | 0.0243 |
| 347 | TENM1 | -0.6261 | 0.0005 |
| 348 | CYP2A7 | -0.6261 | 0.0171 |
| 349 | YBX2 | -0.6261 | 0.0247 |
| 350 | RNU6-322P | -0.6255 | 0.0082 |
| 351 | FAM217A | -0.6251 | 0.0250 |
| 352 | RP11-586D19.1 | -0.6251 | 0.0249 |
| 353 | RIPK4 | -0.6250 | 0.0235 |
| 354 | TRAJ18 | -0.6244 | 0.0122 |
| 355 | RP11-49K24.8 | -0.6243 | 0.0086 |
| 356 | CTD-2162K18.4 | -0.6237 | 0.0223 |
| 357 | RP4-740C4.9 | -0.6237 | 0.0093 |
| 358 | TAS2R4 | -0.6233 | 0.0045 |
| 359 | NADK2-AS1 | -0.6230 | 0.0084 |
| 360 | RP11-191G24.1 | -0.6222 | 0.0136 |
| 361 | PUS7L | -0.6216 | 0.0003 |
| 362 | AC094019.4 | -0.6216 | 0.0181 |
| 363 | RP11-102L12.2 | -0.6213 | 0.0059 |
| 364 | CDKL5 | -0.6206 | 0.0080 |
| 365 | MSH2 | -0.6205 | 3.8227E-12 |
| 366 | GLIPR1L2 | -0.6195 | 0.0128 |
| 367 | IL22 | -0.6187 | 0.0239 |
| 368 | ARHGEF35 | -0.6183 | 0.0084 |
| 369 | RP11-1252I4.2 | -0.6178 | 0.0218 |
| 370 | RP11-474D14.2 | -0.6173 | 0.0157 |
| 371 | RP3-395M20.3 | -0.6171 | 0.0207 |
| 372 | ROBO1 | -0.6169 | 0.0077 |
| 373 | RNU6-1153P | -0.6153 | 0.0274 |
| 374 | CTB-4E7.1 | -0.6146 | 0.0063 |
| 375 | RP4-646N3.1 | -0.6142 | 0.0269 |
| 376 | CTD-2010I16.1 | -0.6139 | 0.0001 |
| 377 | ADAMTSL2 | -0.6133 | 0.0278 |
| 378 | CTC-471F3.5 | -0.6131 | 0.0011 |
| 379 | MSH6 | -0.6127 | 7.3489E-15 |
| 380 | TRAJ20 | -0.6121 | 0.0282 |
| 381 | SLC26A7 | -0.6115 | 0.0164 |
| 382 | CMYA5 | -0.6101 | 0.0098 |
| 383 | AC087380.14 | -0.6099 | 0.0117 |
| 384 | RP11-403I13.4 | -0.6099 | 0.0071 |
| 385 | IGSF9B | -0.6097 | 0.0005 |
| 386 | RP13-131K19.2 | -0.6088 | 0.0285 |
| 387 | AC090571.1 | -0.6076 | 0.0293 |
| 388 | SLC2A12 | -0.6075 | 0.0295 |
| 389 | SLFN12L | -0.6073 | 6.4708E-06 |
| 390 | FTH1P24 | -0.6067 | 0.0248 |
| 391 | RP11-396C23.4 | -0.6063 | 0.0144 |
| 392 | FSIP2 | -0.6060 | 0.0076 |
| 393 | AC026150.8 | -0.6057 | 0.0255 |
| 394 | RP11-829H16.3 | -0.6055 | 0.0278 |
| 395 | RP11-505P4.7 | -0.6049 | 0.0123 |
| 396 | HOMER2P1 | -0.6049 | 0.0164 |
| 397 | NID2 | -0.6042 | 2.0217E-05 |
| 398 | RNU2-6P | -0.6040 | 0.0304 |
| 399 | PRKXP1 | -0.6040 | 0.0093 |
| 400 | RP11-5K23.5 | -0.6038 | 0.0211 |
| 401 | AC015849.16 | -0.6038 | 0.0113 |
| 402 | RP11-70P17.1 | -0.6036 | 0.0263 |
| 403 | RP11-973N13.2 | -0.6026 | 0.0290 |
| 404 | RNA5SP479 | -0.6015 | 0.0109 |
| 405 | RP11-661C3.2 | -0.6010 | 0.0310 |
| 406 | NT5C1B | -0.6008 | 0.0247 |
| 407 | RP11-62H7.3 | -0.6007 | 0.0250 |
| 408 | RP5-998N21.4 | -0.6006 | 0.0217 |
| 409 | SOS1-IT1 | -0.5995 | 0.0021 |
| 410 | RP11-6B6.3 | -0.5995 | 0.0217 |
| 411 | IQCA1 | -0.5993 | 0.0304 |
| 412 | TRGJ2 | -0.5988 | 0.0295 |
| 413 | TAF9BP1 | -0.5988 | 0.0152 |
| 414 | RNU6-531P | -0.5979 | 0.0321 |
| 415 | SDK1 | -0.5978 | 0.0248 |
| 416 | AE000661.37 | -0.5977 | 0.0026 |
| 417 | SRRM1P3 | -0.5975 | 0.0162 |
| 418 | RP11-207C16.4 | -0.5968 | 0.0324 |
| 419 | RP11-701P16.5 | -0.5963 | 0.0193 |
| 420 | RP11-598F7.5 | -0.5961 | 0.0104 |
| 421 | TPM2 | -0.5960 | 1.9796E-05 |
| 422 | TFAP2A | -0.5955 | 0.0254 |
| 423 | AC003104.1 | -0.5953 | 0.0077 |
| 424 | RP11-691H4.4 | -0.5950 | 0.0159 |
| 425 | PNPLA3 | -0.5937 | 0.0075 |
| 426 | RP11-345P4.7 | -0.5936 | 0.0200 |
| 427 | SMARCE1P6 | -0.5930 | 0.0330 |
| 428 | CTD-3236F5.1 | -0.5914 | 0.0276 |
| 429 | RP11-67L3.2 | -0.5910 | 0.0263 |
| 430 | CHL1 | -0.5896 | 0.0293 |
| 431 | MAGOH2 | -0.5891 | 0.0102 |
| 432 | RP11-415F23.3 | -0.5888 | 0.0009 |
| 433 | RC3H1-IT1 | -0.5888 | 0.0174 |
| 434 | ZNF484 | -0.5887 | 0.0002 |
| 435 | OR2A9P | -0.5883 | 0.0205 |
| 436 | CTD-2310F14.1 | -0.5880 | 0.0165 |
| 437 | OR7E7P | -0.5878 | 0.0004 |
| 438 | PLCXD2 | -0.5877 | 0.0001 |
| 439 | SAP30L-AS1 | -0.5876 | 0.0061 |
| 440 | RP11-407G23.4 | -0.5875 | 0.0278 |
| 441 | BCAN | -0.5875 | 0.0142 |
| 442 | SNORA79 | -0.5872 | 0.0312 |
| 443 | L29074.3 | -0.5871 | 0.0215 |
| 444 | CCDC144B | -0.5869 | 0.0259 |
| 445 | SGOL1 | -0.5869 | 0.0065 |
| 446 | RP11-10L12.2 | -0.5868 | 0.0205 |
| 447 | AC016910.1 | -0.5865 | 0.0323 |
| 448 | RASA4 | -0.5861 | 0.0082 |
| 449 | RP11-73B2.6 | -0.5853 | 0.0359 |
| 450 | RP11-1220K2.2 | -0.5837 | 0.0315 |
| 451 | RP1-91J24.3 | -0.5836 | 0.0183 |
| 452 | AC123768.3 | -0.5832 | 0.0310 |
| 453 | TUBB8P1 | -0.5823 | 0.0279 |
| 454 | OSMR | -0.5820 | 0.0210 |
| 455 | RN7SL262P | -0.5817 | 0.0371 |
| 456 | MYCBP2-AS1 | -0.5812 | 0.0294 |
| 457 | GPR52 | -0.5809 | 0.0330 |
| 458 | RHPN2 | -0.5808 | 0.0236 |
| 459 | MLANA | -0.5806 | 0.0010 |
| 460 | CTA-313A17.3 | -0.5805 | 0.0246 |
| 461 | NR2C2 | -0.5803 | 5.9942E-06 |
| 462 | DOCK9-AS2 | -0.5799 | 0.0117 |
| 463 | TRAJ45 | -0.5798 | 0.0377 |
| 464 | DDTP1 | -0.5797 | 0.0325 |
| 465 | AC009961.5 | -0.5794 | 0.0373 |
| 466 | RP11-74E22.6 | -0.5792 | 0.0352 |
| 467 | RP11-385D13.3 | -0.5789 | 0.0360 |
| 468 | RP11-431N15.2 | -0.5788 | 0.0340 |
| 469 | KB-318B8.7 | -0.5786 | 0.0019 |
| 470 | RP11-561P12.5 | -0.5783 | 0.0359 |
| 471 | KRTAP5-AS1 | -0.5781 | 0.0088 |
| 472 | ADAMTS16 | -0.5781 | 0.0383 |
| 473 | RP11-474P2.4 | -0.5780 | 0.0310 |
| 474 | ZNF154 | -0.5777 | 0.0010 |
| 475 | AC010525.7 | -0.5759 | 0.0333 |
| 476 | EYS | -0.5756 | 0.0049 |
| 477 | RP11-452J21.2 | -0.5752 | 0.0392 |
| 478 | ANK3 | -0.5752 | 0.0025 |
| 479 | RP11-573D15.8 | -0.5751 | 0.0256 |
| 480 | TRAJ40 | -0.5750 | 0.0291 |
| 481 | RP11-145M9.5 | -0.5747 | 0.0393 |
| 482 | MID2 | -0.5743 | 0.0023 |
| 483 | CTD-2162K18.3 | -0.5743 | 0.0253 |
| 484 | MED21 | -0.5738 | 1.5452E-06 |
| 485 | ATM | -0.5737 | 1.0057E-08 |
| 486 | RP11-192H23.7 | -0.5736 | 0.0318 |
| 487 | AC118278.1 | -0.5735 | 0.0008 |
| 488 | RBM12B | -0.5730 | 0.0039 |
| 489 | RP11-423P10.2 | -0.5729 | 0.0028 |
| 490 | TXNDC12-AS1 | -0.5727 | 0.0397 |
| 491 | RP11-196G18.3 | -0.5725 | 0.0396 |
| 492 | ZFX-AS1 | -0.5716 | 0.0394 |
| 493 | LEKR1 | -0.5715 | 0.0114 |
| 494 | PCDHB2 | -0.5711 | 0.0139 |
| 495 | CTD-2666L21.2 | -0.5706 | 0.0047 |
| 496 | RP11-1149O23.1 | -0.5706 | 0.0407 |
| 497 | AC097662.2 | -0.5704 | 0.0284 |
| 498 | RP11-91K8.2 | -0.5702 | 0.0343 |
| 499 | HOXA9 | -0.5697 | 0.0270 |
| 500 | SLC35G5 | -0.5690 | 0.0361 |
| 501 | LINC00563 | -0.5686 | 0.0308 |
| 502 | AP000640.2 | -0.5685 | 0.0094 |
| 503 | CTD-2651B20.3 | -0.5675 | 0.0205 |
| 504 | RP4-610C12.4 | -0.5671 | 0.0420 |
| 505 | RP11-826N14.2 | -0.5668 | 0.0393 |
| 506 | HOXA10 | -0.5664 | 0.0372 |
| 507 | KLRC4-KLRK1 | -0.5661 | 0.0113 |
| 508 | RP11-177C12.4 | -0.5660 | 0.0344 |
| 509 | DDX11L2 | -0.5658 | 0.0423 |
| 510 | HS3ST3A1 | -0.5657 | 0.0422 |
| 511 | AC066612.1 | -0.5652 | 0.0102 |
| 512 | RN7SL280P | -0.5644 | 0.0405 |
| 513 | SLC6A3 | -0.5639 | 0.0361 |
| 514 | RP11-43A14.1 | -0.5639 | 0.0420 |
| 515 | RP3-465N24.5 | -0.5638 | 0.0023 |
| 516 | Z95704.5 | -0.5636 | 0.0200 |
| 517 | EXTL3-AS1 | -0.5634 | 0.0080 |
| 518 | RP11-598F7.4 | -0.5629 | 0.0305 |
| 519 | ZNF81 | -0.5619 | 0.0001 |
| 520 | AC069213.1 | -0.5617 | 0.0438 |
| 521 | RP11-552C15.1 | -0.5613 | 0.0346 |
| 522 | TRAJ42 | -0.5613 | 0.0399 |
| 523 | RP11-761I4.3 | -0.5606 | 0.0431 |
| 524 | RP11-480D4.6 | -0.5589 | 0.0451 |
| 525 | RP11-259O18.4 | -0.5586 | 0.0444 |
| 526 | C9orf84 | -0.5584 | 0.0041 |
| 527 | RP11-506K6.4 | -0.5581 | 0.0454 |
| 528 | AC007038.7 | -0.5577 | 0.0010 |
| 529 | BRI3P1 | -0.5577 | 0.0419 |
| 530 | AC131971.1 | -0.5575 | 0.0076 |
| 531 | COL19A1 | -0.5573 | 0.0116 |
| 532 | EDA2R | -0.5572 | 0.0401 |
| 533 | COL4A6 | -0.5560 | 0.0420 |
| 534 | C11orf94 | -0.5560 | 0.0460 |
| 535 | RP11-277P12.20 | -0.5557 | 0.0022 |
| 536 | RP11-50B3.4 | -0.5551 | 0.0199 |
| 537 | RP11-381E24.1 | -0.5549 | 0.0154 |
| 538 | CATSPER2P1 | -0.5547 | 0.0128 |
| 539 | TRIM9 | -0.5545 | 0.0156 |
| 540 | HEATR1 | -0.5543 | 0.0001 |
| 541 | CTD-3099C6.9 | -0.5534 | 0.0017 |
| 542 | SYNE2 | -0.5532 | 1.0702E-05 |
| 543 | PLN | -0.5532 | 0.0340 |
| 544 | DTHD1 | -0.5530 | 0.0053 |
| 545 | NFAT5 | -0.5530 | 0.0001 |
| 546 | FAM96AP2 | -0.5527 | 0.0462 |
| 547 | RP11-513M16.7 | -0.5527 | 0.0114 |
| 548 | RP11-480N24.3 | -0.5526 | 0.0320 |
| 549 | ANKRD44 | -0.5525 | 6.4491E-06 |
| 550 | CTD-3110H11.1 | -0.5523 | 0.0112 |
| 551 | ZDBF2 | -0.5522 | 4.7718E-05 |
| 552 | ALOX12P2 | -0.5522 | 0.0291 |
| 553 | ENTPD3 | -0.5514 | 0.0479 |
| 554 | MIR3936 | -0.5509 | 0.0474 |
| 555 | AC018878.3 | -0.5507 | 0.0169 |
| 556 | RBMXP2 | -0.5501 | 0.0002 |
| 557 | RP11-885B4.2 | -0.5501 | 0.0486 |
| 558 | ZNF70 | -0.5497 | 0.0007 |
| 559 | RPL12P15 | -0.5494 | 0.0427 |
| 560 | RN7SL381P | -0.5493 | 0.0363 |
| 561 | RP11-511P7.4 | -0.5491 | 0.0466 |
| 562 | RP11-141C7.4 | -0.5490 | 0.0487 |
| 563 | PLEKHH2 | -0.5488 | 0.0160 |
| 564 | PHF2P2 | -0.5486 | 0.0461 |
| 565 | RP11-297C4.1 | -0.5485 | 0.0386 |
| 566 | RP11-713N11.4 | -0.5481 | 0.0430 |
| 567 | GOLGA8M | -0.5479 | 0.0428 |
| 568 | LRP6 | -0.5477 | 0.0011 |
| 569 | RP11-210K20.2 | -0.5471 | 0.0419 |
| 570 | OAZ3 | -0.5470 | 0.0131 |
| 571 | CTD-2012J19.1 | -0.5469 | 0.0105 |
| 572 | ACAP2-IT1 | -0.5466 | 0.0193 |
| 573 | RP11-797A18.4 | -0.5466 | 0.0118 |
| 574 | CCR12P | -0.5466 | 0.0195 |
| 575 | ADAM33 | -0.5466 | 0.0497 |
| 576 | SLC26A3 | -0.5465 | 0.0377 |
| 577 | RP11-1277A3.2 | -0.5456 | 0.0094 |
| 578 | ANKRD36B | -0.5451 | 0.0022 |
| 579 | RP11-686D22.10 | -0.5442 | 0.0001 |
| 580 | CTD-3148I10.15 | -0.5440 | 0.0346 |
| 581 | CAMK2N1 | -0.5437 | 0.0054 |
| 582 | EXPH5 | -0.5435 | 0.0008 |
| 583 | RP11-1094M14.7 | -0.5426 | 0.0270 |
| 584 | MUC16 | -0.5424 | 0.0449 |
| 585 | RP11-366M4.11 | -0.5423 | 0.0413 |
| 586 | AC104634.3 | -0.5420 | 0.0464 |
| 587 | RP11-147L13.8 | -0.5420 | 0.0002 |
| 588 | CTD-2245F17.3 | -0.5417 | 0.0239 |
| 589 | SMG1P1 | -0.5415 | 0.0296 |
| 590 | RP11-1094M14.10 | -0.5410 | 0.0470 |
| 591 | LINC00161 | -0.5389 | 0.0172 |
| 592 | LRP1B | -0.5386 | 0.0265 |
| 593 | TIGD7 | -0.5382 | 0.0099 |
| 594 | ZNF295-AS1 | -0.5382 | 0.0227 |
| 595 | TSIX | -0.5381 | 0.0317 |
| 596 | NKX3-1 | -0.5380 | 0.0211 |
| 597 | FMR1-AS1 | -0.5379 | 0.0402 |
| 598 | CTD-2196E14.9 | -0.5376 | 0.0070 |
| 599 | RP11-44F14.8 | -0.5376 | 0.0154 |
| 600 | AKAP6 | -0.5375 | 0.0209 |
| 601 | AP000654.4 | -0.5374 | 0.0281 |
| 602 | FAM90A2P | -0.5374 | 0.0233 |
| 603 | ZNF29P | -0.5370 | 0.0369 |
| 604 | GPR135 | -0.5362 | 0.0020 |
| 605 | RP11-104L21.3 | -0.5361 | 0.0269 |
| 606 | RP11-20D14.6 | -0.5359 | 0.0336 |
| 607 | RP11-204N11.2 | -0.5357 | 0.0368 |
| 608 | PTH2R | -0.5349 | 0.0400 |
| 609 | RP4-802A10.1 | -0.5345 | 0.0458 |
| 610 | RTKN2 | -0.5342 | 0.0035 |
| 611 | AF146191.4 | -0.5342 | 0.0371 |
| 612 | RP11-99A1.2 | -0.5338 | 0.0363 |
| 613 | RP11-767N6.7 | -0.5335 | 0.0124 |
| 614 | RP11-314A20.2 | -0.5320 | 0.0220 |
| 615 | MYOZ2 | -0.5320 | 0.0330 |
| 616 | RP11-484D2.3 | -0.5320 | 0.0362 |
| 617 | ALS2CR12 | -0.5319 | 0.0003 |
| 618 | KB-1047C11.2 | -0.5305 | 0.0442 |
| 619 | LUM | -0.5305 | 0.0488 |
| 620 | LINC01155 | -0.5304 | 0.0079 |
| 621 | NAP1L4P1 | -0.5296 | 0.0488 |
| 622 | LINC00612 | -0.5293 | 0.0067 |
| 623 | RP11-423O2.3 | -0.5288 | 0.0292 |
| 624 | CTD-2530N21.4 | -0.5283 | 0.0054 |
| 625 | HMGN1P2 | -0.5260 | 0.0139 |
| 626 | AC069363.1 | -0.5258 | 0.0351 |
| 627 | AC005523.3 | -0.5258 | 0.0321 |
| 628 | CCDC141 | -0.5257 | 0.0008 |
| 629 | LHX4 | -0.5255 | 0.0005 |
| 630 | PHC3 | -0.5251 | 3.4925E-05 |
| 631 | RP11-262H14.5 | -0.5247 | 0.0403 |
| 632 | DDHD1 | -0.5245 | 7.0761E-06 |
| 633 | HNRNPU-AS1 | -0.5245 | 0.0020 |
| 634 | GPM6B | -0.5241 | 0.0017 |
| 635 | RP11-114I8.4 | -0.5241 | 0.0146 |
| 636 | RP11-552M11.4 | -0.5235 | 0.0313 |
| 637 | MIR186 | -0.5235 | 0.0064 |
| 638 | DPY19L2P2 | -0.5232 | 0.0001 |
| 639 | DGKH | -0.5228 | 0.0001 |
| 640 | RP11-58E21.1 | -0.5228 | 0.0500 |
| 641 | RP11-834C11.7 | -0.5227 | 0.0047 |
| 642 | RP11-413E1.4 | -0.5226 | 0.0193 |
| 643 | TRBJ2-6 | -0.5220 | 0.0163 |
| 644 | RP11-299J3.6 | -0.5217 | 0.0428 |
| 645 | RP11-91A18.4 | -0.5214 | 0.0377 |
| 646 | RP11-295D4.1 | -0.5208 | 0.0492 |
| 647 | NAP1L2 | -0.5204 | 0.0084 |
| 648 | AL109947.2 | -0.5198 | 0.0431 |
| 649 | PZP | -0.5194 | 0.0402 |
| 650 | HELB | -0.5192 | 0.0003 |
| 651 | TNIK | -0.5191 | 0.0001 |
| 652 | RP3-423B22.5 | -0.5190 | 0.0375 |
| 653 | RP11-393I2.4 | -0.5189 | 0.0386 |
| 654 | C21orf140 | -0.5186 | 0.0174 |
| 655 | BRIP1 | -0.5182 | 0.0015 |
| 656 | NPM1P26 | -0.5180 | 0.0460 |
| 657 | RPS10P7 | -0.5180 | 0.0088 |
| 658 | RP11-175P13.3 | -0.5180 | 0.0145 |
| 659 | RP5-1042K10.13 | -0.5178 | 0.0265 |
| 660 | FBXL13 | -0.5176 | 0.0372 |
| 661 | C14orf105 | -0.5175 | 0.0238 |
| 662 | PABPC1P7 | -0.5170 | 0.0443 |
| 663 | MAPT | -0.5167 | 0.0139 |
| 664 | IKZF3 | -0.5167 | 0.0011 |
| 665 | RP1-315G1.1 | -0.5158 | 0.0466 |
| 666 | RAG1 | -0.5157 | 0.0097 |
| 667 | IGHD6-6 | -0.5150 | 0.0369 |
| 668 | BMX | -0.5148 | 0.0405 |
| 669 | RPL7AP10 | -0.5147 | 0.0097 |
| 670 | EFCAB13 | -0.5136 | 0.0151 |
| 671 | CTC-287O8.1 | -0.5136 | 0.0457 |
| 672 | KRR1P1 | -0.5134 | 0.0162 |
| 673 | IL4 | -0.5131 | 0.0325 |
| 674 | VPS13A | -0.5128 | 1.4285E-05 |
| 675 | DNAH6 | -0.5124 | 0.0171 |
| 676 | CTD-2574D22.4 | -0.5122 | 0.0033 |
| 677 | RP11-177C12.1 | -0.5115 | 0.0008 |
| 678 | RP3-508I15.10 | -0.5113 | 0.0434 |
| 679 | RORA | -0.5111 | 0.0005 |
| 680 | CTB-47B11.3 | -0.5110 | 0.0060 |
| 681 | RP11-255E6.5 | -0.5109 | 0.0427 |
| 682 | TRDV2 | -0.5108 | 0.0233 |
| 683 | TRBV5-5 | -0.5108 | 0.0483 |
| 684 | MKLN1 | -0.5103 | 0.0002 |
| 685 | ZNF100 | -0.5101 | 0.0016 |
| 686 | TMPRSS11D | -0.5100 | 0.0483 |
| 687 | C2CD4D | -0.5100 | 0.0294 |
| 688 | DSC1 | -0.5094 | 0.0398 |
| 689 | MYEF2 | -0.5093 | 0.0001 |
| 690 | SLC2A1-AS1 | -0.5084 | 0.0474 |
| 691 | CTD-2561J22.2 | -0.5083 | 0.0057 |
| 692 | RP11-144O23.8 | -0.5076 | 0.0367 |
| 693 | FRG1B | -0.5074 | 0.0062 |
| 694 | EFNA1 | -0.5072 | 0.0398 |
| 695 | ZNF221 | -0.5071 | 0.0276 |
| 696 | TRAJ3 | -0.5059 | 0.0170 |
| 697 | FAM86B1 | -0.5058 | 0.0442 |
| 698 | FAM66C | -0.5048 | 0.0027 |
| 699 | RP3-477O4.14 | -0.5048 | 0.0333 |
| 700 | RP11-440L14.4 | -0.5045 | 0.0203 |
| 701 | AF224669.3 | -0.5041 | 0.0411 |
| 702 | PLCE1 | -0.5033 | 0.0001 |
| 703 | CACNA1C-AS1 | -0.5031 | 0.0070 |
| 704 | RP11-685N10.1 | -0.5024 | 0.0331 |
| 705 | HSPE1P18 | -0.5021 | 0.0219 |
| 706 | AC024937.6 | -0.5020 | 0.0383 |
| 707 | AC008074.5 | -0.5014 | 0.0317 |
| 708 | RP11-96K19.4 | -0.5005 | 0.0359 |
| 709 | AC091729.9 | -0.5003 | 0.0075 |
| 710 | CHIAP1 | -0.4990 | 0.0390 |
| 711 | AC009120.3 | -0.4986 | 0.0338 |
| 712 | RP11-888D10.4 | -0.4984 | 0.0153 |
| 713 | RPL21P44 | -0.4983 | 0.0435 |
| 714 | RP11-338N10.1 | -0.4982 | 0.0377 |
| 715 | AHCTF1P1 | -0.4979 | 0.0087 |
| 716 | ZNF141 | -0.4964 | 0.0005 |
| 717 | ADAM22 | -0.4963 | 0.0002 |
| 718 | CNR1 | -0.4962 | 0.0357 |
| 719 | KLHDC8A | -0.4962 | 0.0378 |
| 720 | SNORA71D | -0.4962 | 0.0238 |
| 721 | SLC24A5 | -0.4958 | 0.0254 |
| 722 | C14orf64 | -0.4954 | 0.0003 |
| 723 | CLDN20 | -0.4953 | 0.0211 |
| 724 | RP11-225N10.3 | -0.4946 | 0.0393 |
| 725 | HMGB1P31 | -0.4944 | 0.0357 |
| 726 | RPL23AP7 | -0.4935 | 0.0315 |
| 727 | AL591025.1 | -0.4932 | 0.0243 |
| 728 | AP001877.1 | -0.4928 | 0.0030 |
| 729 | SOGA3 | -0.4926 | 0.0266 |
| 730 | AC007970.1 | -0.4920 | 0.0336 |
| 731 | ESR1 | -0.4913 | 0.0003 |
| 732 | TET1 | -0.4908 | 0.0018 |
| 733 | LINC00996 | -0.4904 | 0.0075 |
| 734 | SLC16A10 | -0.4903 | 0.0005 |
| 735 | ZNF718 | -0.4903 | 0.0009 |
| 736 | FOXO3B | -0.4895 | 0.0009 |
| 737 | TRAV6 | -0.4892 | 0.0317 |
| 738 | TRAJ48 | -0.4891 | 0.0488 |
| 739 | KAZN | -0.4883 | 0.0485 |
| 740 | GATM | -0.4878 | 0.0339 |
| 741 | C5orf42 | -0.4875 | 0.0002 |
| 742 | RP11-500C11.3 | -0.4872 | 0.0025 |
| 743 | ZNF391 | -0.4866 | 0.0180 |
| 744 | C6orf201 | -0.4859 | 0.0437 |
| 745 | TTC24 | -0.4859 | 0.0003 |
| 746 | RP11-111M22.2 | -0.4858 | 0.0143 |
| 747 | RP11-32B5.1 | -0.4858 | 0.0487 |
| 748 | RALGAPA1P | -0.4855 | 2.7846E-06 |
| 749 | TRGJP2 | -0.4851 | 0.0470 |
| 750 | SNORD12 | -0.4850 | 0.0327 |
| 751 | RP11-736I10.2 | -0.4847 | 0.0481 |
| 752 | MC1R | -0.4842 | 0.0079 |
| 753 | SDR42E1 | -0.4839 | 0.0095 |
| 754 | HKDC1 | -0.4834 | 0.0010 |
| 755 | CTB-58E17.3 | -0.4833 | 0.0354 |
| 756 | MRC2 | -0.4831 | 0.0011 |
| 757 | FLVCR1-AS1 | -0.4828 | 0.0246 |
| 758 | RP11-324O2.3 | -0.4827 | 0.0085 |
| 759 | ZNF354C | -0.4826 | 0.0004 |
| 760 | RP11-925D8.3 | -0.4826 | 0.0361 |
| 761 | ZNF677 | -0.4824 | 0.0012 |
| 762 | AC144521.1 | -0.4823 | 0.0003 |
| 763 | CYP3A5 | -0.4814 | 0.0031 |
| 764 | CD80 | -0.4812 | 0.0460 |
| 765 | RP11-79N23.1 | -0.4798 | 0.0401 |
| 766 | AC007278.2 | -0.4798 | 0.0127 |
| 767 | AC006026.9 | -0.4798 | 0.0469 |
| 768 | RP11-89M16.1 | -0.4796 | 0.0416 |
| 769 | ZNF805 | -0.4793 | 0.0028 |
| 770 | HOXA-AS3 | -0.4790 | 0.0306 |
| 771 | SOX6 | -0.4788 | 0.0186 |
| 772 | YPEL4 | -0.4782 | 0.0290 |
| 773 | CILP | -0.4780 | 0.0056 |
| 774 | NOTCH2NL | -0.4770 | 0.0095 |
| 775 | ZBTB37 | -0.4768 | 0.0002 |
| 776 | CAMK4 | -0.4767 | 4.5850E-06 |
| 777 | RP11-545I5.3 | -0.4761 | 0.0280 |
| 778 | RP5-1073O3.2 | -0.4761 | 0.0275 |
| 779 | MCM8 | -0.4756 | 0.0282 |
| 780 | CYP51A1P2 | -0.4752 | 0.0377 |
| 781 | RP11-56B16.5 | -0.4741 | 0.0413 |
| 782 | PVRIG | -0.4741 | 0.0392 |
| 783 | ATAD5 | -0.4735 | 0.0004 |
| 784 | RP11-317B3.2 | -0.4730 | 0.0416 |
| 785 | CTD-2611K5.5 | -0.4730 | 0.0497 |
| 786 | KRTAP5-8 | -0.4725 | 0.0479 |
| 787 | C5orf63 | -0.4723 | 0.0005 |
| 788 | FAM159A | -0.4722 | 0.0003 |
| 789 | HOXA4 | -0.4719 | 0.0321 |
| 790 | GPR174 | -0.4716 | 0.0008 |
| 791 | ZNF699 | -0.4707 | 0.0063 |
| 792 | SFRP5 | -0.4699 | 0.0363 |
| 793 | OXNAD1 | -0.4696 | 5.0986E-07 |
| 794 | PLEKHM3 | -0.4696 | 0.0006 |
| 795 | FTX | -0.4695 | 0.0012 |
| 796 | AC093627.9 | -0.4692 | 0.0116 |
| 797 | RP11-16E23.4 | -0.4688 | 0.0131 |
| 798 | ZNF681 | -0.4683 | 0.0041 |
| 799 | Z83001.1 | -0.4682 | 0.0429 |
| 800 | HAVCR1 | -0.4677 | 0.0077 |
| 801 | RP1-90L6.2 | -0.4673 | 0.0024 |
| 802 | RP11-10N23.2 | -0.4662 | 0.0379 |
| 803 | ALG13-AS1 | -0.4659 | 0.0072 |
| 804 | RP11-477J21.2 | -0.4657 | 0.0446 |
| 805 | AC122129.1 | -0.4656 | 0.0462 |
| 806 | TNIP3 | -0.4655 | 0.0185 |
| 807 | CARF | -0.4653 | 0.0002 |
| 808 | EFCAB5 | -0.4652 | 0.0390 |
| 809 | LINC00954 | -0.4650 | 0.0025 |
| 810 | TMEM161B-AS1 | -0.4650 | 0.0001 |
| 811 | MORN1 | -0.4632 | 0.0103 |
| 812 | EPPK1 | -0.4627 | 0.0335 |
| 813 | CTB-119C2.1 | -0.4626 | 0.0325 |
| 814 | HHLA2 | -0.4623 | 0.0205 |
| 815 | DNAH10 | -0.4605 | 0.0141 |
| 816 | ZNF35 | -0.4600 | 0.0120 |
| 817 | WASF5P | -0.4594 | 0.0328 |
| 818 | PPARG | -0.4593 | 0.0487 |
| 819 | RP11-73M18.6 | -0.4591 | 0.0104 |
| 820 | SHPRH | -0.4589 | 0.0002 |
| 821 | SLC14A1 | -0.4587 | 0.0014 |
| 822 | RP11-693N9.2 | -0.4581 | 0.0006 |
| 823 | MANEA-AS1 | -0.4580 | 0.0257 |
| 824 | GOLGA6L17P | -0.4576 | 0.0406 |
| 825 | AC006378.2 | -0.4568 | 0.0096 |
| 826 | AL137059.1 | -0.4566 | 0.0287 |
| 827 | INPP4B | -0.4562 | 0.0002 |
| 828 | EFCAB2 | -0.4562 | 0.0188 |
| 829 | RP11-327P2.5 | -0.4557 | 0.0179 |
| 830 | NKD1 | -0.4557 | 0.0303 |
| 831 | HIN1L | -0.4549 | 0.0050 |
| 832 | C12orf60 | -0.4547 | 0.0117 |
| 833 | HOOK1 | -0.4546 | 9.1657E-06 |
| 834 | KCNAB1 | -0.4542 | 0.0110 |
| 835 | GPRIN3 | -0.4542 | 0.0015 |
| 836 | RP11-421L21.2 | -0.4538 | 0.0446 |
| 837 | PTGDR | -0.4537 | 0.0137 |
| 838 | ZNF433 | -0.4525 | 0.0179 |
| 839 | C3orf52 | -0.4519 | 0.0155 |
| 840 | EBLN2 | -0.4516 | 0.0151 |
| 841 | MSL3P1 | -0.4512 | 0.0063 |
| 842 | ZFYVE9 | -0.4506 | 0.0106 |
| 843 | AC010761.13 | -0.4502 | 0.0211 |
| 844 | ZNF594 | -0.4494 | 0.0339 |
| 845 | IL12RB2 | -0.4493 | 0.0335 |
| 846 | FBXL20 | -0.4493 | 0.0006 |
| 847 | SV2A | -0.4468 | 0.0052 |
| 848 | RP3-462E2.5 | -0.4464 | 0.0151 |
| 849 | C21orf49 | -0.4458 | 0.0037 |
| 850 | SPATA32 | -0.4457 | 0.0425 |
| 851 | RP11-65J3.6 | -0.4453 | 0.0394 |
| 852 | HLA-U | -0.4449 | 0.0490 |
| 853 | CTD-2002H8.2 | -0.4448 | 0.0011 |
| 854 | RBM44 | -0.4448 | 0.0063 |
| 855 | RP4-631H13.6 | -0.4446 | 0.0240 |
| 856 | AC006946.12 | -0.4440 | 0.0345 |
| 857 | AC007382.1 | -0.4435 | 0.0188 |
| 858 | JAKMIP1 | -0.4433 | 0.0063 |
| 859 | ZNF483 | -0.4427 | 0.0470 |
| 860 | C10orf118 | -0.4420 | 0.0001 |
| 861 | ADAMTS17 | -0.4419 | 0.0139 |
| 862 | RP11-322D14.2 | -0.4416 | 0.0176 |
| 863 | RP11-52J3.2 | -0.4413 | 0.0439 |
| 864 | RP11-408A13.1 | -0.4413 | 0.0441 |
| 865 | IKZF2 | -0.4411 | 0.0046 |
| 866 | KCNC4 | -0.4408 | 0.0107 |
| 867 | RP1-68D18.4 | -0.4406 | 0.0372 |
| 868 | MTMR8 | -0.4404 | 0.0164 |
| 869 | CCDC152 | -0.4397 | 0.0391 |
| 870 | PLCL1 | -0.4392 | 0.0010 |
| 871 | LINC01160 | -0.4388 | 0.0403 |
| 872 | TUB | -0.4384 | 0.0020 |
| 873 | RP11-70L8.4 | -0.4384 | 0.0489 |
| 874 | ZNF382 | -0.4378 | 0.0033 |
| 875 | ANKRD22 | -0.4378 | 0.0351 |
| 876 | SYNE1 | -0.4372 | 3.6026E-05 |
| 877 | RP4-612B15.3 | -0.4370 | 0.0392 |
| 878 | RICTOR | -0.4369 | 0.0005 |
| 879 | RPS3P2 | -0.4366 | 0.0282 |
| 880 | CNOT6LP1 | -0.4360 | 0.0021 |
| 881 | EML5 | -0.4356 | 0.0009 |
| 882 | ZNF852 | -0.4356 | 0.0034 |
| 883 | REEP6 | -0.4352 | 0.0139 |
| 884 | SMG1 | -0.4349 | 0.0002 |
| 885 | RP11-57K17.1 | -0.4348 | 0.0344 |
| 886 | ZNF662 | -0.4346 | 0.0086 |
| 887 | ZEB1 | -0.4345 | 0.0001 |
| 888 | RP11-474B12.1 | -0.4334 | 0.0490 |
| 889 | AC010468.2 | -0.4332 | 0.0492 |
| 890 | RNA5SP372 | -0.4329 | 0.0463 |
| 891 | PDP2 | -0.4323 | 0.0158 |
| 892 | CCDC39 | -0.4320 | 0.0019 |
| 893 | CCND1 | -0.4318 | 0.0197 |
| 894 | RP5-902P8.10 | -0.4315 | 0.0385 |
| 895 | RP11-428G5.2 | -0.4312 | 0.0481 |
| 896 | CTB-152G17.6 | -0.4308 | 0.0187 |
| 897 | NAALAD2 | -0.4307 | 0.0012 |
| 898 | SPEG | -0.4305 | 0.0043 |
| 899 | PIGF | -0.4302 | 0.0027 |
| 900 | RP3-368A4.5 | -0.4300 | 0.0002 |
| 901 | GTF2IP1 | -0.4297 | 0.0208 |
| 902 | POMK | -0.4291 | 0.0343 |
| 903 | ANXA3 | -0.4288 | 0.0327 |
| 904 | CTC-523E23.1 | -0.4285 | 0.0109 |
| 905 | MICE | -0.4285 | 0.0028 |
| 906 | KLKB1 | -0.4284 | 0.0326 |
| 907 | CD84 | -0.4283 | 0.0022 |
| 908 | AL118506.1 | -0.4281 | 0.0426 |
| 909 | RP1-283E3.8 | -0.4277 | 0.0272 |
| 910 | AC020910.2 | -0.4276 | 0.0419 |
| 911 | MIR4477A | -0.4275 | 0.0121 |
| 912 | STAG3L1 | -0.4274 | 0.0297 |
| 913 | USP34 | -0.4273 | 0.0007 |
| 914 | ILK | -0.4272 | 0.0151 |
| 915 | RP11-617D20.1 | -0.4269 | 0.0115 |
| 916 | FAN1 | -0.4266 | 0.0002 |
| 917 | RP13-487P22.1 | -0.4263 | 0.0463 |
| 918 | EPS8L2 | -0.4261 | 0.0044 |
| 919 | CTD-2017D11.1 | -0.4249 | 0.0237 |
| 920 | ATF7IP | -0.4239 | 0.0002 |
| 921 | IPO7P2 | -0.4232 | 0.0179 |
| 922 | STARD4-AS1 | -0.4231 | 0.0111 |
| 923 | N4BP2 | -0.4227 | 0.0010 |
| 924 | RP11-531A24.5 | -0.4225 | 0.0452 |
| 925 | RP11-440L14.1 | -0.4223 | 0.0090 |
| 926 | ZNF770 | -0.4218 | 0.0059 |
| 927 | RP11-398K22.12 | -0.4213 | 0.0092 |
| 928 | AC084219.3 | -0.4209 | 0.0397 |
| 929 | RP3-395M20.9 | -0.4196 | 0.0262 |
| 930 | RP11-159N11.4 | -0.4192 | 0.0429 |
| 931 | AC093110.3 | -0.4189 | 0.0101 |
| 932 | PGAP1 | -0.4186 | 0.0005 |
| 933 | SLC25A53 | -0.4173 | 0.0005 |
| 934 | NCR3LG1 | -0.4171 | 0.0211 |
| 935 | C1RL-AS1 | -0.4166 | 0.0034 |
| 936 | MTRNR2L8 | -0.4162 | 0.0454 |
| 937 | UBASH3A | -0.4161 | 0.0010 |
| 938 | RBM41 | -0.4154 | 0.0002 |
| 939 | NBPF3 | -0.4148 | 0.0362 |
| 940 | AC073046.25 | -0.4147 | 0.0326 |
| 941 | XRN1 | -0.4147 | 0.0005 |
| 942 | KIAA1919 | -0.4136 | 0.0131 |
| 943 | BOD1L1 | -0.4134 | 0.0045 |
| 944 | DBF4P1 | -0.4134 | 0.0278 |
| 945 | TTC28 | -0.4128 | 0.0161 |
| 946 | CTD-2313F11.1 | -0.4124 | 0.0391 |
| 947 | TRIM66 | -0.4124 | 0.0055 |
| 948 | DBH-AS1 | -0.4119 | 0.0151 |
| 949 | TNK1 | -0.4115 | 0.0002 |
| 950 | RP11-337C18.8 | -0.4109 | 0.0123 |
| 951 | ZNF253 | -0.4108 | 0.0141 |
| 952 | MKL2 | -0.4106 | 0.0005 |
| 953 | CTD-2260A17.1 | -0.4106 | 0.0498 |
| 954 | INADL | -0.4103 | 0.0005 |
| 955 | RP11-480A16.1 | -0.4086 | 0.0308 |
| 956 | SYNJ2BP | -0.4084 | 0.0011 |
| 957 | BDP1 | -0.4076 | 0.0004 |
| 958 | KLHL25 | -0.4058 | 0.0371 |
| 959 | STX18-AS1 | -0.4054 | 0.0166 |
| 960 | HSF5 | -0.4054 | 0.0374 |
| 961 | RTTN | -0.4053 | 3.7109E-06 |
| 962 | RP1-151F17.2 | -0.4048 | 0.0081 |
| 963 | CTD-2575K13.6 | -0.4043 | 0.0221 |
| 964 | ZNF540 | -0.4034 | 0.0043 |
| 965 | HAR1A | -0.4031 | 0.0279 |
| 966 | CATSPER2 | -0.4029 | 0.0083 |
| 967 | RP11-20I20.4 | -0.4027 | 0.0184 |
| 968 | THEMIS | -0.4026 | 0.0003 |
| 969 | FAM86HP | -0.4020 | 0.0212 |
| 970 | RP11-53B2.2 | -0.4016 | 0.0154 |
| 971 | ZNF224 | -0.4012 | 0.0020 |
| 972 | FUBP1 | -0.4011 | 4.9327E-05 |
| 973 | BRWD1 | -0.4011 | 0.0003 |
| 974 | LINC00969 | -0.4007 | 0.0022 |
| 975 | RP1-179N16.6 | -0.4003 | 0.0368 |
| 976 | APBA2 | -0.4000 | 0.0057 |
| 977 | MYO9A | -0.3999 | 0.0031 |
| 978 | MCTP2 | -0.3993 | 0.0018 |
| 979 | ANKHD1 | -0.3979 | 0.0096 |
| 980 | KMT2A | -0.3974 | 7.0557E-06 |
| 981 | ZNF853 | -0.3967 | 0.0018 |
| 982 | AC015849.19 | -0.3963 | 0.0096 |
| 983 | ZNF91 | -0.3962 | 0.0002 |
| 984 | RP11-212P7.2 | -0.3961 | 0.0152 |
| 985 | MACF1 | -0.3957 | 0.0007 |
| 986 | RPL23AP64 | -0.3957 | 0.0166 |
| 987 | TSHZ2 | -0.3951 | 0.0128 |
| 988 | ZNF737 | -0.3945 | 0.0270 |
| 989 | SH3RF3 | -0.3937 | 0.0037 |
| 990 | DAPK2 | -0.3937 | 0.0178 |
| 991 | ZNF793 | -0.3930 | 0.0195 |
| 992 | ZNF543 | -0.3923 | 0.0483 |
| 993 | CCDC85C | -0.3916 | 0.0402 |
| 994 | NKTR | -0.3914 | 4.5342E-06 |
| 995 | ZNF107 | -0.3911 | 0.0005 |
| 996 | RP11-58E21.3 | -0.3903 | 0.0226 |
| 997 | RP11-541N10.3 | -0.3902 | 0.0043 |
| 998 | RP4-545C24.1 | -0.3901 | 0.0449 |
| 999 | CTD-2647L4.4 | -0.3901 | 0.0020 |
| 1000 | RNF157-AS1 | -0.3900 | 0.0059 |
| 1001 | DYNC2H1 | -0.3899 | 0.0080 |
| 1002 | AC074138.3 | -0.3898 | 0.0075 |
| 1003 | CRTAM | -0.3886 | 0.0193 |
| 1004 | LOH12CR1 | -0.3884 | 0.0062 |
| 1005 | THAP6 | -0.3883 | 0.0073 |
| 1006 | CYB5RL | -0.3879 | 0.0088 |
| 1007 | NONOP2 | -0.3874 | 0.0141 |
| 1008 | TRIM47 | -0.3872 | 0.0415 |
| 1009 | HCG18 | -0.3871 | 0.0001 |
| 1010 | STXBP1 | -0.3869 | 0.0182 |
| 1011 | MGA | -0.3868 | 1.9419E-05 |
| 1012 | GPR89B | -0.3864 | 0.0459 |
| 1013 | SLC5A3 | -0.3855 | 0.0013 |
| 1014 | CLUHP3 | -0.3854 | 0.0294 |
| 1015 | MPRIPP1 | -0.3839 | 0.0175 |
| 1016 | DIXDC1 | -0.3835 | 0.0138 |
| 1017 | MDN1 | -0.3834 | 0.0001 |
| 1018 | ARHGEF34P | -0.3832 | 0.0482 |
| 1019 | ASH1L | -0.3818 | 0.0001 |
| 1020 | MPHOSPH9 | -0.3816 | 4.4183E-06 |
| 1021 | ATP6V0E2-AS1 | -0.3814 | 0.0196 |
| 1022 | RP11-603J24.5 | -0.3809 | 0.0466 |
| 1023 | GPR155 | -0.3808 | 0.0008 |
| 1024 | ZNF417 | -0.3803 | 0.0021 |
| 1025 | ZNF708 | -0.3799 | 0.0013 |
| 1026 | RP11-156P1.3 | -0.3796 | 0.0028 |
| 1027 | C14orf182 | -0.3796 | 0.0050 |
| 1028 | RN7SL834P | -0.3792 | 0.0323 |
| 1029 | SCML4 | -0.3789 | 2.5559E-05 |
| 1030 | STAMBPL1 | -0.3785 | 0.0002 |
| 1031 | CACNA1I | -0.3785 | 0.0006 |
| 1032 | CTB-129O4.1 | -0.3775 | 0.0450 |
| 1033 | BHLHB9 | -0.3771 | 0.0216 |
| 1034 | RP11-264B17.2 | -0.3769 | 0.0181 |
| 1035 | PAG1 | -0.3763 | 0.0004 |
| 1036 | ANKRD12 | -0.3763 | 0.0005 |
| 1037 | RP11-767N6.2 | -0.3759 | 0.0400 |
| 1038 | ZIK1 | -0.3758 | 0.0445 |
| 1039 | TMPPE | -0.3757 | 0.0201 |
| 1040 | TDRKH | -0.3756 | 0.0010 |
| 1041 | TNRC6C | -0.3754 | 0.0016 |
| 1042 | PBLD | -0.3754 | 0.0048 |
| 1043 | SDHAP1 | -0.3739 | 0.0015 |
| 1044 | CEP44 | -0.3738 | 0.0005 |
| 1045 | GOLGA2P5 | -0.3733 | 0.0001 |
| 1046 | TLR3 | -0.3731 | 0.0365 |
| 1047 | CAPN7 | -0.3723 | 0.0003 |
| 1048 | PDK1 | -0.3720 | 8.1922E-07 |
| 1049 | ZSCAN12 | -0.3716 | 0.0044 |
| 1050 | RPAP2 | -0.3713 | 0.0001 |
| 1051 | ZNF397 | -0.3704 | 0.0381 |
| 1052 | LRRC69 | -0.3702 | 0.0080 |
| 1053 | TMEM106B | -0.3700 | 0.0007 |
| 1054 | LNPEP | -0.3700 | 0.0006 |
| 1055 | RP11-730K11.1 | -0.3699 | 0.0237 |
| 1056 | MRPS31P5 | -0.3698 | 0.0166 |
| 1057 | LSG1 | -0.3697 | 0.0114 |
| 1058 | RP11-85F14.5 | -0.3692 | 0.0470 |
| 1059 | GCC2 | -0.3691 | 3.6616E-05 |
| 1060 | RAB11FIP1P1 | -0.3688 | 0.0214 |
| 1061 | PDE3B | -0.3686 | 0.0082 |
| 1062 | KIAA1377 | -0.3684 | 0.0089 |
| 1063 | TRAV17 | -0.3684 | 0.0034 |
| 1064 | AGO2 | -0.3682 | 3.8721E-05 |
| 1065 | MEN1 | -0.3682 | 1.4276E-05 |
| 1066 | DET1 | -0.3681 | 0.0082 |
| 1067 | VPS13C | -0.3681 | 0.0013 |
| 1068 | RP11-683L23.1 | -0.3676 | 0.0483 |
| 1069 | NPHP3 | -0.3674 | 0.0075 |
| 1070 | GPR75 | -0.3673 | 0.0382 |
| 1071 | CCDC136 | -0.3668 | 0.0057 |
| 1072 | RP11-158H5.7 | -0.3667 | 0.0049 |
| 1073 | ZNF649 | -0.3661 | 0.0062 |
| 1074 | RP11-110I1.5 | -0.3656 | 0.0473 |
| 1075 | COL6A3 | -0.3653 | 0.0464 |
| 1076 | DENND4A | -0.3652 | 0.0001 |
| 1077 | LL22NC03-2H8.5 | -0.3651 | 0.0280 |
| 1078 | SLCO4C1 | -0.3649 | 0.0451 |
| 1079 | FAM208B | -0.3646 | 0.0001 |
| 1080 | GPR125 | -0.3643 | 0.0115 |
| 1081 | LEF1-AS1 | -0.3639 | 0.0279 |
| 1082 | ANKEF1 | -0.3636 | 0.0194 |
| 1083 | CHIC1 | -0.3626 | 0.0119 |
| 1084 | DGKE | -0.3615 | 4.7842E-05 |
| 1085 | TSGA10 | -0.3613 | 0.0284 |
| 1086 | SUV39H2 | -0.3610 | 0.0259 |
| 1087 | ABCD2 | -0.3606 | 0.0015 |
| 1088 | RP11-1023L17.1 | -0.3602 | 0.0059 |
| 1089 | ACE | -0.3597 | 0.0253 |
| 1090 | SLFN5 | -0.3597 | 0.0206 |
| 1091 | ERCC6L2 | -0.3597 | 0.0039 |
| 1092 | NPAT | -0.3592 | 0.0002 |
| 1093 | DNAJC24 | -0.3591 | 0.0049 |
| 1094 | TP53 | -0.3586 | 0.0018 |
| 1095 | RP11-430C7.4 | -0.3586 | 0.0487 |
| 1096 | SCAF11 | -0.3582 | 0.0004 |
| 1097 | PANK3 | -0.3581 | 0.0014 |
| 1098 | TTBK2 | -0.3580 | 0.0040 |
| 1099 | TRBV23-1 | -0.3574 | 0.0030 |
| 1100 | CEP85L | -0.3573 | 0.0007 |
| 1101 | TMSB15B | -0.3572 | 0.0341 |
| 1102 | LINC00861 | -0.3570 | 0.0006 |
| 1103 | CNPY2 | -0.3566 | 0.0224 |
| 1104 | PTAR1 | -0.3565 | 0.0014 |
| 1105 | ZC3H12B | -0.3564 | 0.0122 |
| 1106 | CTD-2358C21.4 | -0.3549 | 0.0179 |
| 1107 | NUDT3 | -0.3548 | 0.0026 |
| 1108 | FAM169A | -0.3541 | 0.0423 |
| 1109 | TPH1 | -0.3537 | 0.0327 |
| 1110 | ANKRD10-IT1 | -0.3526 | 0.0134 |
| 1111 | PPIE | -0.3525 | 0.0215 |
| 1112 | PPP2R3A | -0.3524 | 0.0332 |
| 1113 | ZXDA | -0.3519 | 0.0103 |
| 1114 | CRIPT | -0.3519 | 0.0060 |
| 1115 | ZMYND10 | -0.3518 | 0.0247 |
| 1116 | KIAA1147 | -0.3517 | 2.5665E-05 |
| 1117 | RPGRIP1L | -0.3517 | 0.0176 |
| 1118 | NDFIP2 | -0.3516 | 0.0277 |
| 1119 | GK5 | -0.3513 | 0.0036 |
| 1120 | SCML1 | -0.3512 | 0.0057 |
| 1121 | ZNF525 | -0.3510 | 0.0246 |
| 1122 | LCOR | -0.3510 | 0.0073 |
| 1123 | EPHX2 | -0.3505 | 0.0094 |
| 1124 | RP11-504P24.2 | -0.3498 | 0.0485 |
| 1125 | TRAV8-1 | -0.3498 | 0.0248 |
| 1126 | C1orf147 | -0.3491 | 0.0310 |
| 1127 | ACVR1C | -0.3489 | 0.0063 |
| 1128 | PSMD6-AS2 | -0.3488 | 0.0457 |
| 1129 | snoU13 | -0.3487 | 0.0015 |
| 1130 | MYSM1 | -0.3487 | 0.0004 |
| 1131 | KANSL1L | -0.3485 | 0.0015 |
| 1132 | USP37 | -0.3483 | 0.0152 |
| 1133 | RNPC3 | -0.3482 | 0.0068 |
| 1134 | ZKSCAN8 | -0.3480 | 4.1025E-06 |
| 1135 | EXOSC3 | -0.3479 | 0.0159 |
| 1136 | RP11-1114A5.4 | -0.3477 | 0.0316 |
| 1137 | PLCH2 | -0.3473 | 0.0008 |
| 1138 | TMEM65 | -0.3473 | 0.0034 |
| 1139 | CTD-2368P22.1 | -0.3472 | 0.0207 |
| 1140 | RBP5 | -0.3472 | 0.0055 |
| 1141 | ZNF33B | -0.3469 | 0.0002 |
| 1142 | ATRX | -0.3461 | 0.0004 |
| 1143 | RP11-159G9.5 | -0.3455 | 0.0466 |
| 1144 | MBD5 | -0.3452 | 0.0008 |
| 1145 | DENND4C | -0.3447 | 0.0010 |
| 1146 | MORF4L2-AS1 | -0.3444 | 0.0222 |
| 1147 | UTP20 | -0.3443 | 0.0001 |
| 1148 | FLT3LG | -0.3439 | 0.0122 |
| 1149 | C17orf67 | -0.3437 | 0.0003 |
| 1150 | TTC14 | -0.3427 | 0.0004 |
| 1151 | SPTBN1 | -0.3425 | 0.0002 |
| 1152 | FZD3 | -0.3420 | 0.0228 |
| 1153 | TRBV20-1 | -0.3412 | 0.0248 |
| 1154 | NAA35 | -0.3412 | 0.0019 |
| 1155 | ZNF493 | -0.3407 | 0.0028 |
| 1156 | RFX7 | -0.3396 | 0.0002 |
| 1157 | CEP350 | -0.3393 | 0.0019 |
| 1158 | PLAG1 | -0.3393 | 0.0025 |
| 1159 | PCNXL2 | -0.3384 | 0.0001 |
| 1160 | CD160 | -0.3381 | 0.0416 |
| 1161 | VPS13B | -0.3381 | 0.0008 |
| 1162 | DDX12P | -0.3374 | 0.0120 |
| 1163 | ACSL6 | -0.3371 | 0.0021 |
| 1164 | ZNF292 | -0.3371 | 0.0005 |
| 1165 | GS1-124K5.2 | -0.3369 | 0.0452 |
| 1166 | AGL | -0.3368 | 0.0002 |
| 1167 | BIRC3 | -0.3367 | 0.0029 |
| 1168 | MASP2 | -0.3367 | 0.0027 |
| 1169 | NABP1 | -0.3366 | 0.0375 |
| 1170 | KIAA1551 | -0.3365 | 0.0109 |
| 1171 | SOCS4 | -0.3354 | 0.0091 |
| 1172 | AC009495.2 | -0.3351 | 0.0187 |
| 1173 | ZEB1-AS1 | -0.3350 | 0.0237 |
| 1174 | ANKRD26 | -0.3350 | 0.0050 |
| 1175 | RP11-23N2.4 | -0.3347 | 0.0365 |
| 1176 | CLK4 | -0.3346 | 0.0048 |
| 1177 | GTPBP8 | -0.3345 | 0.0046 |
| 1178 | FAM161A | -0.3340 | 0.0380 |
| 1179 | KLF12 | -0.3333 | 0.0023 |
| 1180 | ZNF507 | -0.3326 | 0.0005 |
| 1181 | RPL32P3 | -0.3323 | 0.0128 |
| 1182 | ODF2L | -0.3322 | 0.0070 |
| 1183 | HELZ | -0.3321 | 0.0005 |
| 1184 | HIVEP2 | -0.3319 | 3.5409E-05 |
| 1185 | ZNRD1-AS1 | -0.3318 | 0.0465 |
| 1186 | ZBTB16 | -0.3316 | 0.0147 |
| 1187 | KIAA1671 | -0.3312 | 0.0277 |
| 1188 | OBSCN | -0.3311 | 0.0148 |
| 1189 | RP11-47I22.3 | -0.3307 | 0.0494 |
| 1190 | AKAP11 | -0.3307 | 0.0008 |
| 1191 | MTERF | -0.3305 | 0.0020 |
| 1192 | RP11-174G6.5 | -0.3304 | 0.0032 |
| 1193 | AP001258.4 | -0.3303 | 0.0371 |
| 1194 | SPRY3 | -0.3303 | 0.0333 |
| 1195 | FAM126B | -0.3301 | 0.0017 |
| 1196 | LINC00893 | -0.3301 | 0.0153 |
| 1197 | CD274 | -0.3294 | 0.0361 |
| 1198 | DUSP16 | -0.3293 | 0.0055 |
| 1199 | USP44 | -0.3292 | 0.0442 |
| 1200 | CHD7 | -0.3292 | 0.0001 |
| 1201 | DOCK9 | -0.3284 | 0.0001 |
| 1202 | CD28 | -0.3278 | 0.0021 |
| 1203 | ZNF138 | -0.3275 | 0.0021 |
| 1204 | RLIM | -0.3272 | 0.0012 |
| 1205 | RP4-657D16.3 | -0.3265 | 0.0481 |
| 1206 | ARHGAP5 | -0.3263 | 0.0025 |
| 1207 | RP1-59D14.5 | -0.3263 | 0.0300 |
| 1208 | SMC4 | -0.3261 | 0.0290 |
| 1209 | C8orf46 | -0.3254 | 0.0400 |
| 1210 | CLOCK | -0.3254 | 0.0035 |
| 1211 | RP11-295P9.3 | -0.3253 | 0.0001 |
| 1212 | NUFIP2 | -0.3249 | 0.0023 |
| 1213 | FAM63B | -0.3248 | 0.0027 |
| 1214 | RSF1 | -0.3243 | 0.0001 |
| 1215 | ATP7A | -0.3242 | 0.0117 |
| 1216 | NR3C2 | -0.3230 | 0.0065 |
| 1217 | CASD1 | -0.3227 | 0.0046 |
| 1218 | PHIP | -0.3226 | 0.0026 |
| 1219 | KDM5A | -0.3225 | 0.0001 |
| 1220 | RP11-85K15.2 | -0.3223 | 0.0346 |
| 1221 | CTD-2555O16.4 | -0.3222 | 0.0434 |
| 1222 | FBXO22 | -0.3220 | 0.0042 |
| 1223 | TMEM194B | -0.3217 | 0.0092 |
| 1224 | AC025171.1 | -0.3217 | 0.0474 |
| 1225 | FAM228B | -0.3214 | 0.0129 |
| 1226 | KATNAL1 | -0.3198 | 0.0173 |
| 1227 | ZSCAN30 | -0.3196 | 0.0003 |
| 1228 | FANCB | -0.3178 | 0.0443 |
| 1229 | CENPL | -0.3176 | 0.0117 |
| 1230 | ITPR2 | -0.3173 | 0.0027 |
| 1231 | MSMO1 | -0.3170 | 0.0113 |
| 1232 | LMBRD2 | -0.3166 | 0.0026 |
| 1233 | TRMT13 | -0.3166 | 0.0207 |
| 1234 | DYRK2 | -0.3166 | 0.0003 |
| 1235 | SENP7 | -0.3166 | 0.0030 |
| 1236 | TTC21B | -0.3165 | 0.0002 |
| 1237 | TRANK1 | -0.3162 | 0.0185 |
| 1238 | FKTN | -0.3162 | 0.0017 |
| 1239 | ZC3H6 | -0.3162 | 0.0002 |
| 1240 | MYCBP2 | -0.3159 | 0.0079 |
| 1241 | ZNF283 | -0.3153 | 0.0484 |
| 1242 | DOCK3 | -0.3152 | 0.0122 |
| 1243 | ARL10 | -0.3148 | 0.0284 |
| 1244 | RP11-894P9.1 | -0.3140 | 0.0338 |
| 1245 | REV3L | -0.3135 | 0.0105 |
| 1246 | CEP97 | -0.3135 | 0.0075 |
| 1247 | FAM92A1 | -0.3135 | 0.0280 |
| 1248 | KLHL11 | -0.3132 | 0.0302 |
| 1249 | WRN | -0.3132 | 0.0100 |
| 1250 | RP11-1407O15.2 | -0.3131 | 0.0135 |
| 1251 | PTPN4 | -0.3125 | 0.0061 |
| 1252 | GMDS-AS1 | -0.3123 | 0.0253 |
| 1253 | ZBTB46 | -0.3115 | 0.0022 |
| 1254 | STX17 | -0.3113 | 0.0011 |
| 1255 | WDR52 | -0.3110 | 0.0009 |
| 1256 | SMCHD1 | -0.3107 | 0.0115 |
| 1257 | ALMS1 | -0.3106 | 0.0129 |
| 1258 | FRA10AC1 | -0.3105 | 0.0029 |
| 1259 | BPTF | -0.3104 | 0.0006 |
| 1260 | ZNF605 | -0.3103 | 0.0006 |
| 1261 | BTAF1 | -0.3093 | 0.0036 |
| 1262 | ZNF12 | -0.3091 | 0.0026 |
| 1263 | C5orf56 | -0.3091 | 0.0007 |
| 1264 | ZNF431 | -0.3091 | 0.0055 |
| 1265 | ACO1 | -0.3079 | 0.0278 |
| 1266 | APOL6 | -0.3077 | 0.0032 |
| 1267 | TC2N | -0.3077 | 0.0145 |
| 1268 | KIAA0947 | -0.3065 | 0.0014 |
| 1269 | KMT2C | -0.3064 | 0.0063 |
| 1270 | CTC-228N24.3 | -0.3061 | 0.0009 |
| 1271 | LUZP1 | -0.3060 | 0.0065 |
| 1272 | CDC7 | -0.3058 | 0.0201 |
| 1273 | GPALPP1 | -0.3055 | 0.0074 |
| 1274 | ZDHHC21 | -0.3055 | 0.0019 |
| 1275 | ZNF506 | -0.3051 | 0.0088 |
| 1276 | DENND2D | -0.3051 | 0.0246 |
| 1277 | SACS | -0.3045 | 0.0082 |
| 1278 | ZCCHC11 | -0.3045 | 0.0001 |
| 1279 | ZNRF2 | -0.3042 | 0.0037 |
| 1280 | UBN2 | -0.3042 | 0.0011 |
| 1281 | TARBP1 | -0.3042 | 0.0012 |
| 1282 | BRWD3 | -0.3041 | 0.0032 |
| 1283 | ZBTB20 | -0.3039 | 0.0439 |
| 1284 | SLC7A6 | -0.3036 | 4.9777E-05 |
| 1285 | BICD1 | -0.3034 | 0.0052 |
| 1286 | KIF20B | -0.3028 | 0.0099 |
| 1287 | LIN54 | -0.3027 | 0.0006 |
| 1288 | PCNX | -0.3024 | 0.0007 |
| 1289 | CEP290 | -0.3021 | 0.0060 |
| 1290 | CCL28 | -0.3019 | 0.0220 |
| 1291 | LL0XNC01-7P3.1 | -0.3017 | 0.0348 |
| 1292 | EPG5 | -0.3016 | 0.0011 |
| 1293 | BAZ2B | -0.3016 | 0.0256 |
| 1294 | TFDP2 | -0.3013 | 0.0045 |
| 1295 | ZNF222 | -0.3013 | 0.0367 |
| 1296 | SLC35F5 | -0.3012 | 0.0235 |
| 1297 | LMO7 | -0.3008 | 0.0019 |
| 1298 | C1orf192 | -0.3005 | 0.0484 |
| 1299 | ATXN7 | -0.3004 | 0.0044 |
| 1300 | ANKRD32 | -0.3004 | 0.0103 |
| 1301 | NAPEPLD | -0.3001 | 0.0190 |
| 1302 | VPS13D | -0.2999 | 0.0001 |
| 1303 | RAD1 | -0.2996 | 0.0009 |
| 1304 | AC009403.2 | -0.2991 | 0.0320 |
| 1305 | NKIRAS1 | -0.2991 | 0.0431 |
| 1306 | NUFIP1 | -0.2990 | 0.0442 |
| 1307 | ZNF264 | -0.2983 | 0.0077 |
| 1308 | ZFC3H1 | -0.2983 | 0.0009 |
| 1309 | ST8SIA1 | -0.2981 | 0.0462 |
| 1310 | PREPL | -0.2979 | 0.0139 |
| 1311 | RNF157 | -0.2977 | 0.0135 |
| 1312 | AKAP9 | -0.2977 | 0.0037 |
| 1313 | LRRC37A4P | -0.2976 | 0.0209 |
| 1314 | LINC01125 | -0.2975 | 0.0244 |
| 1315 | MEMO1 | -0.2975 | 0.0282 |
| 1316 | GABPB1-AS1 | -0.2975 | 0.0163 |
| 1317 | ZCCHC6 | -0.2971 | 0.0195 |
| 1318 | TBCCD1 | -0.2971 | 0.0063 |
| 1319 | SPICE1 | -0.2971 | 0.0128 |
| 1320 | METTL15 | -0.2971 | 0.0148 |
| 1321 | RP11-288H12.3 | -0.2970 | 0.0342 |
| 1322 | NLE1 | -0.2964 | 0.0405 |
| 1323 | CASK | -0.2963 | 0.0110 |
| 1324 | TNFRSF25 | -0.2962 | 0.0083 |
| 1325 | JMJD1C | -0.2960 | 0.0117 |
| 1326 | RPE | -0.2955 | 0.0029 |
| 1327 | PHLDB3 | -0.2953 | 0.0313 |
| 1328 | TCP11L1 | -0.2953 | 0.0123 |
| 1329 | FAM86DP | -0.2947 | 0.0348 |
| 1330 | OGT | -0.2933 | 2.8367E-05 |
| 1331 | PRKDC | -0.2933 | 0.0012 |
| 1332 | RAD50 | -0.2926 | 0.0010 |
| 1333 | NF1 | -0.2923 | 0.0004 |
| 1334 | ZFYVE16 | -0.2923 | 0.0340 |
| 1335 | ZNF337 | -0.2922 | 0.0115 |
| 1336 | ITGA6 | -0.2922 | 0.0060 |
| 1337 | GUSBP1 | -0.2920 | 0.0408 |
| 1338 | HERC1 | -0.2915 | 0.0093 |
| 1339 | RALGAPA1 | -0.2912 | 0.0091 |
| 1340 | AC083843.1 | -0.2906 | 0.0159 |
| 1341 | DPP4 | -0.2903 | 0.0171 |
| 1342 | C8orf37 | -0.2901 | 0.0235 |
| 1343 | TRAF5 | -0.2899 | 0.0021 |
| 1344 | NEK1 | -0.2897 | 0.0079 |
| 1345 | LNX2 | -0.2892 | 0.0007 |
| 1346 | NOC3L | -0.2890 | 0.0124 |
| 1347 | KIF21A | -0.2886 | 0.0411 |
| 1348 | UBQLN2 | -0.2886 | 0.0039 |
| 1349 | DYNC1H1 | -0.2878 | 0.0147 |
| 1350 | ARID4B | -0.2877 | 0.0010 |
| 1351 | ZNF204P | -0.2875 | 0.0052 |
| 1352 | TBC1D32 | -0.2873 | 0.0456 |
| 1353 | CCDC14 | -0.2872 | 0.0034 |
| 1354 | KLHL3 | -0.2872 | 0.0035 |
| 1355 | KPNA5 | -0.2870 | 0.0268 |
| 1356 | RP5-1007M22.2 | -0.2865 | 0.0024 |
| 1357 | AC024560.3 | -0.2865 | 0.0170 |
| 1358 | CBX5 | -0.2861 | 0.0053 |
| 1359 | MYNN | -0.2861 | 0.0052 |
| 1360 | SP4 | -0.2857 | 0.0084 |
| 1361 | TMEM161B | -0.2856 | 0.0031 |
| 1362 | SH2D1A | -0.2854 | 0.0386 |
| 1363 | UBR5 | -0.2851 | 0.0020 |
| 1364 | BBX | -0.2851 | 0.0008 |
| 1365 | RNF144A | -0.2847 | 0.0112 |
| 1366 | ZNF546 | -0.2846 | 0.0197 |
| 1367 | KIAA1109 | -0.2840 | 0.0056 |
| 1368 | SLC4A7 | -0.2837 | 0.0215 |
| 1369 | LA16c-390H2.4 | -0.2831 | 0.0481 |
| 1370 | KIAA2026 | -0.2827 | 0.0031 |
| 1371 | ADAT1 | -0.2827 | 0.0002 |
| 1372 | CEP68 | -0.2827 | 0.0059 |
| 1373 | TRIM33 | -0.2826 | 0.0009 |
| 1374 | TRAT1 | -0.2822 | 0.0218 |
| 1375 | RALGPS1 | -0.2821 | 0.0037 |
| 1376 | AP001062.7 | -0.2820 | 0.0365 |
| 1377 | EDAR | -0.2820 | 0.0463 |
| 1378 | DOPEY1 | -0.2814 | 0.0187 |
| 1379 | BIRC6 | -0.2812 | 0.0043 |
| 1380 | FRYL | -0.2803 | 0.0056 |
| 1381 | ARAP2 | -0.2803 | 0.0363 |
| 1382 | TSEN2 | -0.2801 | 0.0096 |
| 1383 | USP45 | -0.2796 | 0.0094 |
| 1384 | PCM1 | -0.2796 | 0.0066 |
| 1385 | MAP9 | -0.2794 | 0.0122 |
| 1386 | TCF7 | -0.2790 | 0.0027 |
| 1387 | AC006129.2 | -0.2787 | 0.0123 |
| 1388 | BTLA | -0.2777 | 0.0331 |
| 1389 | SCAI | -0.2772 | 0.0017 |
| 1390 | OSBPL8 | -0.2771 | 0.0031 |
| 1391 | TRIM32 | -0.2768 | 0.0469 |
| 1392 | PTPDC1 | -0.2767 | 0.0183 |
| 1393 | ENOSF1 | -0.2765 | 0.0079 |
| 1394 | ARMCX2 | -0.2760 | 0.0347 |
| 1395 | DDX17 | -0.2755 | 7.7087E-06 |
| 1396 | CHD9 | -0.2749 | 0.0121 |
| 1397 | DPY19L4 | -0.2746 | 0.0086 |
| 1398 | OFD1 | -0.2744 | 0.0029 |
| 1399 | LYRM7 | -0.2743 | 0.0175 |
| 1400 | HAUS3 | -0.2742 | 0.0194 |
| 1401 | ZNF720 | -0.2741 | 0.0093 |
| 1402 | STXBP5 | -0.2737 | 0.0047 |
| 1403 | NIPBL | -0.2736 | 0.0027 |
| 1404 | GLS | -0.2736 | 0.0027 |
| 1405 | FAM8A1 | -0.2734 | 0.0017 |
| 1406 | ARID4A | -0.2734 | 0.0048 |
| 1407 | C16orf52 | -0.2732 | 0.0299 |
| 1408 | C1GALT1 | -0.2728 | 0.0050 |
| 1409 | RIC3 | -0.2727 | 0.0038 |
| 1410 | TRPM7 | -0.2721 | 0.0031 |
| 1411 | DLEU1 | -0.2715 | 0.0092 |
| 1412 | TRABD2A | -0.2711 | 0.0224 |
| 1413 | MCOLN2 | -0.2702 | 0.0404 |
| 1414 | EXOG | -0.2701 | 0.0041 |
| 1415 | SFXN4 | -0.2700 | 0.0309 |
| 1416 | PAXIP1-AS2 | -0.2698 | 0.0138 |
| 1417 | NUP43 | -0.2696 | 0.0387 |
| 1418 | SFMBT1 | -0.2689 | 0.0123 |
| 1419 | LYSMD3 | -0.2688 | 0.0146 |
| 1420 | SDCBP2-AS1 | -0.2686 | 0.0227 |
| 1421 | RIF1 | -0.2685 | 0.0041 |
| 1422 | GUF1 | -0.2682 | 0.0050 |
| 1423 | NAA16 | -0.2679 | 0.0014 |
| 1424 | TAOK1 | -0.2678 | 0.0226 |
| 1425 | TBC1D19 | -0.2678 | 0.0427 |
| 1426 | CEP192 | -0.2676 | 0.0112 |
| 1427 | ZNF841 | -0.2662 | 0.0499 |
| 1428 | DICER1 | -0.2662 | 0.0263 |
| 1429 | KIAA1107 | -0.2661 | 0.0499 |
| 1430 | ZNF84 | -0.2655 | 0.0231 |
| 1431 | ACP6 | -0.2654 | 0.0283 |
| 1432 | KRIT1 | -0.2654 | 0.0295 |
| 1433 | NNT-AS1 | -0.2652 | 0.0483 |
| 1434 | ZNF644 | -0.2650 | 0.0093 |
| 1435 | VWA8 | -0.2647 | 0.0032 |
| 1436 | MED13 | -0.2645 | 0.0018 |
| 1437 | ZNF846 | -0.2644 | 0.0494 |
| 1438 | TTC37 | -0.2643 | 0.0043 |
| 1439 | RANBP2 | -0.2638 | 0.0068 |
| 1440 | STK11 | -0.2632 | 3.3657E-05 |

**Supplementary Table 3**. Upregulated genes in PBMCs from IS mice.

|  | **Gene** | **log2 Fold Change** | **p value** |
| --- | --- | --- | --- |
| 1 | Asprv1 | 3.6166 | 1.55E-23 |
| 2 | Stfa2l1 | 3.5785 | 4.34E-30 |
| 3 | Mybph | 3.2585 | 2.53E-19 |
| 4 | Fpr1 | 3.1643 | 3.22E-21 |
| 5 | Irg1 | 3.1225 | 1.20E-17 |
| 6 | Lrg1 | 3.1203 | 1.54E-35 |
| 7 | Il1r2 | 3.0846 | 1.10E-26 |
| 8 | Gm5483 | 3.0409 | 2.14E-21 |
| 9 | Tarm1 | 2.9601 | 3.06E-21 |
| 10 | BC100530 | 2.9220 | 6.56E-19 |
| 11 | Stfa2 | 2.8917 | 5.55E-15 |
| 12 | Mrgpra2a | 2.7901 | 1.44E-14 |
| 13 | Bst1 | 2.7861 | 1.37E-30 |
| 14 | Rnd1 | 2.7672 | 1.37E-31 |
| 15 | Upp1 | 2.7226 | 8.04E-15 |
| 16 | Cxcr2 | 2.7215 | 4.74E-24 |
| 17 | Clec5a | 2.6592 | 1.79E-28 |
| 18 | Il1rn | 2.6032 | 5.62E-19 |
| 19 | Ceacam18 | 2.5699 | 6.22E-13 |
| 20 | Marcksl1 | 2.5636 | 4.74E-14 |
| 21 | Rhov | 2.5507 | 2.15E-12 |
| 22 | Siglece | 2.5479 | 3.46E-19 |
| 23 | Gm10872 | 2.5262 | 1.01E-15 |
| 24 | Steap4 | 2.5066 | 7.18E-13 |
| 25 | Gpr84 | 2.4858 | 1.61E-13 |
| 26 | Gm16712 | 2.4842 | 2.79E-18 |
| 27 | Il1bos | 2.4477 | 9.16E-17 |
| 28 | Hdc | 2.4409 | 1.37E-16 |
| 29 | Crispld2 | 2.4370 | 2.31E-19 |
| 30 | Mgam | 2.4186 | 8.20E-17 |
| 31 | Ccr1 | 2.3943 | 2.42E-14 |
| 32 | Sycp2 | 2.3851 | 7.18E-11 |
| 33 | Ifitm1 | 2.3622 | 4.70E-20 |
| 34 | Adora1 | 2.3521 | 4.95E-10 |
| 35 | Cd33 | 2.3469 | 3.65E-27 |
| 36 | Rdh12 | 2.3449 | 9.85E-19 |
| 37 | Plin5 | 2.3375 | 7.73E-10 |
| 38 | Psca | 2.3233 | 2.45E-09 |
| 39 | Slc2a6 | 2.3206 | 2.12E-14 |
| 40 | Dhrs9 | 2.3157 | 3.72E-12 |
| 41 | P2ry13 | 2.3099 | 2.89E-16 |
| 42 | Cpne2 | 2.3085 | 4.27E-27 |
| 43 | Fpr2 | 2.2850 | 1.30E-21 |
| 44 | Wfdc17 | 2.2833 | 5.15E-22 |
| 45 | S100a9 | 2.2746 | 1.79E-18 |
| 46 | Clec4e | 2.2663 | 3.56E-21 |
| 47 | Mmp8 | 2.2606 | 1.13E-21 |
| 48 | Mrgpra2b | 2.2488 | 6.03E-12 |
| 49 | Chil1 | 2.2352 | 1.85E-16 |
| 50 | Csf1 | 2.2302 | 1.78E-21 |
| 51 | BC117090 | 2.2082 | 1.47E-08 |
| 52 | Nos2 | 2.2037 | 1.21E-08 |
| 53 | 1100001G20Rik | 2.2011 | 5.59E-15 |
| 54 | Il1f9 | 2.1863 | 4.62E-09 |
| 55 | Nfkbiz | 2.1703 | 6.70E-15 |
| 56 | Mefv | 2.1603 | 1.03E-13 |
| 57 | Rufy4 | 2.1398 | 2.20E-09 |
| 58 | Dgat2 | 2.1383 | 9.53E-15 |
| 59 | Ifnlr1 | 2.1237 | 3.77E-11 |
| 60 | Ifitm5 | 2.1235 | 3.33E-08 |
| 61 | Col5a1 | 2.1215 | 1.31E-10 |
| 62 | Cd300lf | 2.1121 | 2.55E-23 |
| 63 | C5ar1 | 2.1017 | 2.15E-13 |
| 64 | Lrrc25 | 2.0949 | 4.99E-18 |
| 65 | Cd14 | 2.0944 | 5.23E-31 |
| 66 | Lcn2 | 2.0826 | 2.71E-13 |
| 67 | S100a8 | 2.0674 | 7.10E-15 |
| 68 | Nlrp3 | 2.0656 | 3.64E-10 |
| 69 | Kcnj2 | 2.0638 | 4.50E-12 |
| 70 | Hp | 2.0514 | 7.11E-17 |
| 71 | 4933431G14Rik | 2.0362 | 1.18E-07 |
| 72 | Il1b | 2.0356 | 3.27E-08 |
| 73 | Lipg | 2.0334 | 9.94E-08 |
| 74 | Csf3r | 2.0327 | 2.05E-11 |
| 75 | Dmxl2 | 2.0197 | 1.31E-12 |
| 76 | Slc6a14 | 2.0177 | 2.37E-07 |
| 77 | Cxcl2 | 2.0152 | 2.18E-10 |
| 78 | Plet1 | 2.0060 | 2.09E-07 |
| 79 | F630028O10Rik | 1.9995 | 5.11E-22 |
| 80 | Tnf | 1.9958 | 5.71E-09 |
| 81 | Slc40a1 | 1.9860 | 2.20E-11 |
| 82 | G0s2 | 1.9726 | 1.66E-08 |
| 83 | F3 | 1.9715 | 3.55E-08 |
| 84 | Mal | 1.9651 | 4.55E-07 |
| 85 | AA467197 | 1.9617 | 1.51E-08 |
| 86 | Trpm2 | 1.9617 | 4.80E-11 |
| 87 | Slfn4 | 1.9610 | 2.60E-12 |
| 88 | Mapk13 | 1.9559 | 5.24E-22 |
| 89 | 4931406B18Rik | 1.9468 | 4.44E-07 |
| 90 | Trem1 | 1.9360 | 6.66E-16 |
| 91 | Marcks | 1.9345 | 1.93E-37 |
| 92 | Txnl4b | 1.9289 | 4.91E-12 |
| 93 | 5430425K12Rik | 1.9258 | 1.47E-07 |
| 94 | Hcar2 | 1.9253 | 1.06E-07 |
| 95 | Gca | 1.9246 | 2.20E-16 |
| 96 | Il1rap | 1.9194 | 5.64E-16 |
| 97 | Cd101 | 1.9088 | 2.19E-07 |
| 98 | Mmp9 | 1.9081 | 6.63E-13 |
| 99 | Gdf15 | 1.8976 | 2.50E-07 |
| 100 | Lilrb4 | 1.8792 | 2.39E-08 |
| 101 | Chrm3 | 1.8716 | 2.45E-07 |
| 102 | Cxcl3 | 1.8688 | 3.60E-07 |
| 103 | Gp49a | 1.8605 | 2.98E-08 |
| 104 | Rab11fip1 | 1.8567 | 7.19E-12 |
| 105 | Sema6b | 1.8505 | 5.10E-13 |
| 106 | Itgb2l | 1.8376 | 1.12E-13 |
| 107 | Entpd3 | 1.8346 | 1.80E-06 |
| 108 | Cdc42bpg | 1.8282 | 2.72E-08 |
| 109 | Plekhs1 | 1.8261 | 2.93E-06 |
| 110 | Cdc42ep2 | 1.8254 | 3.65E-07 |
| 111 | Clec4d | 1.8171 | 1.72E-12 |
| 112 | Trim30b | 1.8127 | 2.46E-11 |
| 113 | Sgms2 | 1.8124 | 4.27E-14 |
| 114 | Gm16894 | 1.8119 | 1.11E-06 |
| 115 | Clca4 | 1.8024 | 3.85E-06 |
| 116 | Ptafr | 1.8008 | 3.60E-09 |
| 117 | Lbp | 1.7887 | 6.20E-09 |
| 118 | Arg2 | 1.7704 | 4.55E-11 |
| 119 | Ppp1r3d | 1.7698 | 7.39E-09 |
| 120 | Batf | 1.7690 | 1.93E-26 |
| 121 | Acta2 | 1.7624 | 1.79E-10 |
| 122 | Bmx | 1.7584 | 9.18E-15 |
| 123 | Rab20 | 1.7583 | 9.55E-12 |
| 124 | Samsn1 | 1.7507 | 2.41E-16 |
| 125 | Mtus1 | 1.7462 | 8.48E-16 |
| 126 | N4bp1 | 1.7395 | 4.93E-16 |
| 127 | Gbp2 | 1.7389 | 3.82E-18 |
| 128 | Anxa1 | 1.7382 | 7.20E-18 |
| 129 | Icam1 | 1.7240 | 2.51E-18 |
| 130 | Snai1 | 1.7225 | 1.68E-08 |
| 131 | Tnfaip2 | 1.7195 | 5.91E-13 |
| 132 | Pram1 | 1.7100 | 2.47E-11 |
| 133 | Krt86 | 1.7068 | 1.66E-07 |
| 134 | Sfxn5 | 1.7034 | 3.88E-10 |
| 135 | Glis3 | 1.6886 | 4.24E-06 |
| 136 | Hunk | 1.6826 | 1.30E-05 |
| 137 | Muc4 | 1.6793 | 1.89E-06 |
| 138 | Rnf149 | 1.6623 | 3.17E-17 |
| 139 | Krt13 | 1.6548 | 2.04E-05 |
| 140 | S100a11 | 1.6511 | 2.65E-14 |
| 141 | Slc15a3 | 1.6511 | 1.84E-13 |
| 142 | Ankrd22 | 1.6482 | 5.51E-06 |
| 143 | Prr33 | 1.6430 | 2.36E-12 |
| 144 | Bcl3 | 1.6427 | 2.85E-14 |
| 145 | Nlrp12 | 1.6341 | 1.25E-10 |
| 146 | Slc24a1 | 1.6248 | 6.63E-06 |
| 147 | Ccrl2 | 1.6208 | 3.24E-08 |
| 148 | Slc7a11 | 1.6173 | 6.80E-07 |
| 149 | Fbxl5 | 1.6104 | 1.16E-17 |
| 150 | Rasip1 | 1.6078 | 5.75E-07 |
| 151 | Ngp | 1.6057 | 9.47E-08 |
| 152 | Gm9573 | 1.6051 | 3.30E-05 |
| 153 | Adamtsl4 | 1.6034 | 5.12E-10 |
| 154 | Mreg | 1.5971 | 2.71E-07 |
| 155 | Il23a | 1.5943 | 4.19E-05 |
| 156 | Prss16 | 1.5857 | 4.59E-06 |
| 157 | Prss27 | 1.5838 | 4.16E-05 |
| 158 | Mxd1 | 1.5826 | 4.00E-14 |
| 159 | Casp4 | 1.5754 | 7.92E-13 |
| 160 | Lhx1 | 1.5734 | 5.43E-05 |
| 161 | Slfn2 | 1.5713 | 5.33E-17 |
| 162 | Tlr13 | 1.5647 | 7.00E-09 |
| 163 | Ltf | 1.5643 | 1.78E-08 |
| 164 | Inhba | 1.5642 | 6.17E-05 |
| 165 | Sh2b2 | 1.5639 | 1.73E-08 |
| 166 | Mcemp1 | 1.5628 | 2.12E-20 |
| 167 | Per2 | 1.5504 | 1.12E-12 |
| 168 | Gm6377 | 1.5460 | 2.31E-12 |
| 169 | Ambp | 1.5381 | 7.97E-05 |
| 170 | Lmnb1 | 1.5348 | 2.28E-15 |
| 171 | Prnp | 1.5290 | 4.89E-10 |
| 172 | Il1a | 1.5222 | 4.91E-05 |
| 173 | Ccm2l | 1.5148 | 8.75E-05 |
| 174 | Plscr1 | 1.5122 | 2.21E-14 |
| 175 | Fgl2 | 1.5114 | 6.23E-09 |
| 176 | Soat2 | 1.4986 | 5.41E-08 |
| 177 | Slc27a4 | 1.4982 | 1.21E-10 |
| 178 | Pglyrp1 | 1.4854 | 5.22E-12 |
| 179 | Tlr4 | 1.4818 | 2.93E-06 |
| 180 | Prok2 | 1.4811 | 0.0001459 |
| 181 | Slc36a3 | 1.4804 | 0.0001499 |
| 182 | Pfkfb3 | 1.4796 | 1.64E-17 |
| 183 | Itpkc | 1.4707 | 5.88E-24 |
| 184 | Themis2 | 1.4697 | 4.51E-12 |
| 185 | 4933416M07Rik | 1.4665 | 5.03E-05 |
| 186 | Tas2r143 | 1.4627 | 0.0001152 |
| 187 | Abca13 | 1.4583 | 4.99E-05 |
| 188 | Tnfrsf23 | 1.4581 | 4.78E-07 |
| 189 | Amer2 | 1.4452 | 0.0001599 |
| 190 | Sirpb1b | 1.4306 | 2.06E-06 |
| 191 | 1700047M11Rik | 1.4256 | 5.34E-05 |
| 192 | Igfbp6 | 1.4126 | 0.0002765 |
| 193 | Gbp5 | 1.4108 | 6.26E-12 |
| 194 | Cdk5r1 | 1.4065 | 8.17E-07 |
| 195 | Pygl | 1.4049 | 1.30E-10 |
| 196 | Lin28a | 1.4048 | 0.0003213 |
| 197 | Cpa3 | 1.4033 | 4.32E-06 |
| 198 | Trem3 | 1.4004 | 3.37E-08 |
| 199 | Slc9a3r2 | 1.4002 | 1.05E-05 |
| 200 | Orm1 | 1.3994 | 1.74E-05 |
| 201 | C1rl | 1.3968 | 2.37E-06 |
| 202 | Sod2 | 1.3920 | 1.57E-23 |
| 203 | Vcan | 1.3897 | 1.48E-05 |
| 204 | Mmp25 | 1.3859 | 1.68E-06 |
| 205 | Ncam1 | 1.3855 | 0.0001139 |
| 206 | Epha1 | 1.3823 | 7.88E-06 |
| 207 | Csf3 | 1.3750 | 0.0003761 |
| 208 | Tex15 | 1.3746 | 3.53E-05 |
| 209 | Gm20752 | 1.3730 | 0.0004297 |
| 210 | Prom1 | 1.3710 | 0.0003105 |
| 211 | Dfna5 | 1.3699 | 2.79E-09 |
| 212 | Fam110a | 1.3682 | 3.49E-06 |
| 213 | Oasl2 | 1.3671 | 1.87E-06 |
| 214 | Slc22a15 | 1.3669 | 2.10E-11 |
| 215 | Gbp11 | 1.3577 | 1.01E-12 |
| 216 | Ptgds | 1.3573 | 0.000464 |
| 217 | Stfa3 | 1.3543 | 0.0005253 |
| 218 | Gm14085 | 1.3505 | 9.02E-12 |
| 219 | Igf1r | 1.3500 | 7.15E-13 |
| 220 | St3gal6 | 1.3495 | 2.58E-07 |
| 221 | Lpcat2 | 1.3469 | 1.48E-08 |
| 222 | Padi4 | 1.3437 | 2.41E-12 |
| 223 | Ffar2 | 1.3436 | 0.0005569 |
| 224 | Scrg1 | 1.3431 | 0.0002958 |
| 225 | Slc28a2 | 1.3405 | 1.61E-18 |
| 226 | Apol10a | 1.3395 | 0.0004559 |
| 227 | Cyp3a13 | 1.3390 | 0.0006 |
| 228 | Dusp16 | 1.3374 | 2.78E-10 |
| 229 | Ankrd33b | 1.3362 | 2.83E-06 |
| 230 | Chil3 | 1.3233 | 1.12E-05 |
| 231 | Socs3 | 1.3232 | 3.94E-17 |
| 232 | Hc | 1.3183 | 0.0006388 |
| 233 | Alas1 | 1.3148 | 1.86E-07 |
| 234 | Epn3 | 1.3144 | 0.0005497 |
| 235 | Hif1a | 1.3132 | 6.57E-11 |
| 236 | Fcgr4 | 1.3115 | 5.69E-13 |
| 237 | Fas | 1.3105 | 1.41E-11 |
| 238 | Gadd45b | 1.2966 | 0.0001247 |
| 239 | Xkr8 | 1.2864 | 0.0001096 |
| 240 | Slc5a1 | 1.2846 | 0.0005944 |
| 241 | Sh2d3c | 1.2833 | 1.31E-07 |
| 242 | Camp | 1.2831 | 6.94E-06 |
| 243 | Gm5150 | 1.2806 | 1.20E-06 |
| 244 | Retnlg | 1.2794 | 5.35E-06 |
| 245 | Ptgs2 | 1.2789 | 0.0001069 |
| 246 | Bend4 | 1.2771 | 3.19E-09 |
| 247 | Gpr97 | 1.2770 | 2.92E-09 |
| 248 | Nod2 | 1.2728 | 3.37E-06 |
| 249 | Itga5 | 1.2684 | 3.30E-09 |
| 250 | Piwil2 | 1.2658 | 0.0006277 |
| 251 | Calml3 | 1.2622 | 0.0007969 |
| 252 | Tchh | 1.2596 | 0.0012207 |
| 253 | Neb | 1.2576 | 5.20E-05 |
| 254 | Ggt5 | 1.2539 | 6.03E-09 |
| 255 | Ptger2 | 1.2537 | 1.11E-05 |
| 256 | Adam8 | 1.2535 | 1.44E-08 |
| 257 | Dhrs7 | 1.2524 | 2.11E-12 |
| 258 | Lcp1 | 1.2507 | 6.68E-11 |
| 259 | Anxa3 | 1.2488 | 2.02E-11 |
| 260 | C5ar2 | 1.2445 | 6.61E-05 |
| 261 | Ifitm6 | 1.2400 | 3.52E-11 |
| 262 | Nfe2l2 | 1.2376 | 1.88E-06 |
| 263 | Hecw2 | 1.2359 | 0.0005858 |
| 264 | Tnfsf8 | 1.2325 | 1.18E-07 |
| 265 | Nfkbia | 1.2275 | 1.54E-06 |
| 266 | Plk2 | 1.2269 | 6.27E-07 |
| 267 | Agap1 | 1.2268 | 4.64E-07 |
| 268 | Igsf6 | 1.2247 | 3.71E-09 |
| 269 | Ncf1 | 1.2222 | 5.76E-09 |
| 270 | Saa3 | 1.2080 | 0.0015815 |
| 271 | Wfikkn2 | 1.2039 | 0.0016816 |
| 272 | Pirb | 1.2030 | 1.96E-11 |
| 273 | B430010I23Rik | 1.2021 | 0.0018927 |
| 274 | Iigp1 | 1.2018 | 4.01E-05 |
| 275 | Ccdc37 | 1.2004 | 0.000774 |
| 276 | Elf3 | 1.1955 | 0.0014124 |
| 277 | Olfr456 | 1.1951 | 0.0013367 |
| 278 | Pi16 | 1.1842 | 9.92E-09 |
| 279 | Hk3 | 1.1837 | 5.43E-11 |
| 280 | Basp1 | 1.1832 | 3.12E-05 |
| 281 | Tha1 | 1.1822 | 0.0003217 |
| 282 | Hnmt | 1.1818 | 0.0003118 |
| 283 | Rab32 | 1.1806 | 1.10E-08 |
| 284 | Hgsnat | 1.1801 | 8.02E-12 |
| 285 | Asns | 1.1791 | 0.0001067 |
| 286 | Fosl1 | 1.1785 | 0.0024448 |
| 287 | Rhou | 1.1771 | 1.49E-07 |
| 288 | Mir6386 | 1.1714 | 0.0025621 |
| 289 | Pilrb2 | 1.1714 | 4.70E-05 |
| 290 | Atp11a | 1.1692 | 1.37E-21 |
| 291 | Fmo2 | 1.1691 | 0.0023651 |
| 292 | Megf9 | 1.1664 | 1.01E-07 |
| 293 | Adpgk | 1.1593 | 6.47E-18 |
| 294 | Kcnip4 | 1.1592 | 0.0029631 |
| 295 | Fam114a1 | 1.1583 | 1.21E-06 |
| 296 | Card10 | 1.1568 | 1.82E-05 |
| 297 | Grhl3 | 1.1556 | 0.0023367 |
| 298 | Agrn | 1.1550 | 7.49E-10 |
| 299 | Slc16a3 | 1.1547 | 2.75E-05 |
| 300 | Bcl2l15 | 1.1531 | 0.0004502 |
| 301 | Ccnjl | 1.1526 | 0.0030092 |
| 302 | Galnt3 | 1.1520 | 0.0004719 |
| 303 | Tlr6 | 1.1489 | 8.36E-07 |
| 304 | Trib1 | 1.1475 | 8.27E-11 |
| 305 | Ifitm2 | 1.1456 | 1.49E-13 |
| 306 | Zswim4 | 1.1439 | 2.52E-07 |
| 307 | Cd163l1 | 1.1437 | 4.48E-06 |
| 308 | Spatc1 | 1.1390 | 0.0025499 |
| 309 | Il10 | 1.1377 | 0.0020388 |
| 310 | Edn1 | 1.1345 | 0.0008615 |
| 311 | BC048671 | 1.1327 | 0.0036176 |
| 312 | Cebpd | 1.1295 | 6.88E-08 |
| 313 | Chst11 | 1.1283 | 4.83E-07 |
| 314 | Pilra | 1.1282 | 7.76E-05 |
| 315 | Hmga1 | 1.1280 | 0.0022623 |
| 316 | Lilra6 | 1.1272 | 9.43E-09 |
| 317 | 1600014C10Rik | 1.1270 | 2.79E-12 |
| 318 | Gm5416 | 1.1256 | 0.0029862 |
| 319 | Cdc42ep4 | 1.1233 | 1.01E-09 |
| 320 | Snord89 | 1.1230 | 0.0002866 |
| 321 | Jdp2 | 1.1171 | 2.58E-05 |
| 322 | Sema4a | 1.1169 | 2.60E-10 |
| 323 | Erg | 1.1162 | 0.0015709 |
| 324 | S100a6 | 1.1160 | 3.40E-09 |
| 325 | Celsr3 | 1.1155 | 8.68E-08 |
| 326 | Rbm47 | 1.1150 | 4.86E-05 |
| 327 | Mocos | 1.1106 | 2.80E-05 |
| 328 | Ceacam10 | 1.1096 | 0.0006514 |
| 329 | S100a7a | 1.1096 | 0.0036606 |
| 330 | Zfp263 | 1.0967 | 3.03E-14 |
| 331 | Cpd | 1.0950 | 6.06E-06 |
| 332 | 2810474O19Rik | 1.0947 | 1.39E-20 |
| 333 | Adora2b | 1.0943 | 0.0010075 |
| 334 | Alox5 | 1.0933 | 7.16E-06 |
| 335 | Slfn1 | 1.0910 | 2.18E-06 |
| 336 | Bnip3 | 1.0908 | 0.0008058 |
| 337 | Entpd1 | 1.0906 | 2.35E-06 |
| 338 | Sbno2 | 1.0898 | 4.12E-10 |
| 339 | Mtmr6 | 1.0898 | 2.11E-12 |
| 340 | Hspa13 | 1.0880 | 6.08E-14 |
| 341 | Tspan1 | 1.0872 | 0.0043201 |
| 342 | Lhfpl2 | 1.0863 | 0.0022952 |
| 343 | Pim1 | 1.0842 | 1.28E-09 |
| 344 | 2310007B03Rik | 1.0842 | 0.0032459 |
| 345 | Amdhd2 | 1.0829 | 9.99E-07 |
| 346 | Mybpc3 | 1.0812 | 0.0001707 |
| 347 | Wdfy3 | 1.0794 | 1.08E-07 |
| 348 | Ccno | 1.0791 | 0.0011232 |
| 349 | Rab23 | 1.0790 | 0.0003329 |
| 350 | Lrp12 | 1.0741 | 0.000173 |
| 351 | Serpinb1a | 1.0719 | 1.86E-11 |
| 352 | Ckap4 | 1.0715 | 4.74E-12 |
| 353 | Il1r1 | 1.0710 | 0.0001839 |
| 354 | Clec4b2 | 1.0703 | 0.0061274 |
| 355 | Clec7a | 1.0659 | 2.74E-05 |
| 356 | Gpr110 | 1.0642 | 0.0036062 |
| 357 | Hist1h2bh | 1.0641 | 0.0025581 |
| 358 | Sell | 1.0634 | 6.20E-28 |
| 359 | Hdac4 | 1.0575 | 7.48E-13 |
| 360 | Rtp4 | 1.0552 | 1.61E-05 |
| 361 | Bcl2a1a | 1.0537 | 0.0002428 |
| 362 | Coq4 | 1.0531 | 0.0014301 |
| 363 | 1700071M16Rik | 1.0510 | 6.15E-06 |
| 364 | Alpl | 1.0507 | 0.0064852 |
| 365 | Atg3 | 1.0507 | 7.26E-10 |
| 366 | 4933428G20Rik | 1.0505 | 0.0041324 |
| 367 | Il18bp | 1.0502 | 2.23E-06 |
| 368 | Map3k8 | 1.0497 | 5.82E-06 |
| 369 | Tlr2 | 1.0459 | 0.0002648 |
| 370 | Rps6ka4 | 1.0446 | 2.51E-09 |
| 371 | Abtb2 | 1.0429 | 2.25E-18 |
| 372 | Aatk | 1.0402 | 2.96E-05 |
| 373 | Fam110c | 1.0379 | 0.0077547 |
| 374 | Krt7 | 1.0363 | 0.0049175 |
| 375 | Stab1 | 1.0314 | 0.0031255 |
| 376 | Anxa2 | 1.0310 | 1.51E-13 |
| 377 | Ppp1r42 | 1.0304 | 0.0083129 |
| 378 | Csf2rb2 | 1.0302 | 4.66E-13 |
| 379 | F730035M05Rik | 1.0247 | 0.004326 |
| 380 | Arhgef3 | 1.0236 | 3.25E-07 |
| 381 | Ctsg | 1.0222 | 0.008429 |
| 382 | Msrb1 | 1.0219 | 1.10E-05 |
| 383 | Map1b | 1.0211 | 0.0089258 |
| 384 | Trnp1 | 1.0200 | 0.0075427 |
| 385 | Ccrn4l | 1.0188 | 4.37E-07 |
| 386 | Txn1 | 1.0178 | 3.96E-08 |
| 387 | Capns2 | 1.0176 | 0.0052722 |
| 388 | Zfp281 | 1.0166 | 1.73E-09 |
| 389 | Trib2 | 1.0164 | 9.14E-07 |
| 390 | Fam84a | 1.0149 | 0.0093591 |
| 391 | Tmc4 | 1.0111 | 2.95E-07 |
| 392 | Cyp4f18 | 1.0106 | 5.15E-05 |
| 393 | Tas2r135 | 1.0093 | 0.0047367 |
| 394 | Ms4a4b | 1.0073 | 0.0003529 |
| 395 | Slpi | 1.0055 | 2.38E-10 |
| 396 | Cacfd1 | 1.0033 | 8.93E-06 |
| 397 | Slc16a14 | 1.0024 | 0.0055773 |
| 398 | Plin4 | 1.0022 | 0.0092305 |
| 399 | C3 | 0.9998 | 1.48E-08 |
| 400 | Khdc3 | 0.9993 | 0.0051981 |
| 401 | Klhl2 | 0.9987 | 8.27E-07 |
| 402 | 4930506M07Rik | 0.9971 | 7.60E-05 |
| 403 | Prss8 | 0.9961 | 0.0080904 |
| 404 | Cyp2s1 | 0.9955 | 0.0001336 |
| 405 | Ttc30a2 | 0.9952 | 0.0059811 |
| 406 | Dync2li1 | 0.9943 | 0.0082373 |
| 407 | Mcpt8 | 0.9887 | 0.0046475 |
| 408 | Myd88 | 0.9885 | 1.11E-07 |
| 409 | Zfp503 | 0.9881 | 0.0083624 |
| 410 | Il18rap | 0.9875 | 2.52E-07 |
| 411 | Clec4n | 0.9864 | 9.20E-07 |
| 412 | Ccdc146 | 0.9837 | 0.0045425 |
| 413 | Rnd3 | 0.9804 | 1.50E-06 |
| 414 | Gm19705 | 0.9790 | 0.0046973 |
| 415 | Gpr116 | 0.9785 | 0.0120659 |
| 416 | Ovgp1 | 0.9775 | 0.0001445 |
| 417 | Ikzf4 | 0.9758 | 0.0010316 |
| 418 | 5830428M24Rik | 0.9705 | 7.78E-07 |
| 419 | Retn | 0.9690 | 0.0130297 |
| 420 | Insl3 | 0.9668 | 0.0016985 |
| 421 | Miat | 0.9660 | 0.0095331 |
| 422 | Lyst | 0.9606 | 8.28E-05 |
| 423 | Cd177 | 0.9595 | 1.64E-07 |
| 424 | Mettl9 | 0.9568 | 1.77E-13 |
| 425 | Fgr | 0.9565 | 8.75E-06 |
| 426 | Pald1 | 0.9549 | 0.0007845 |
| 427 | Nrg1 | 0.9546 | 0.0069322 |
| 428 | Gbp7 | 0.9526 | 4.40E-08 |
| 429 | Fcgr3 | 0.9504 | 1.05E-07 |
| 430 | Csf2rb | 0.9491 | 1.01E-13 |
| 431 | Gys1 | 0.9485 | 2.92E-06 |
| 432 | Snx18 | 0.9478 | 1.03E-06 |
| 433 | Mthfs | 0.9466 | 9.80E-05 |
| 434 | Zfp719 | 0.9452 | 0.0001435 |
| 435 | Gm1604b | 0.9440 | 0.0143555 |
| 436 | Vsig10l | 0.9418 | 0.0032066 |
| 437 | Ascl4 | 0.9418 | 0.0158006 |
| 438 | Sntb2 | 0.9384 | 2.97E-05 |
| 439 | Ets2 | 0.9381 | 1.45E-09 |
| 440 | Mir6387 | 0.9378 | 0.0083893 |
| 441 | Phlda1 | 0.9377 | 0.0002764 |
| 442 | Ltb4r1 | 0.9339 | 1.30E-08 |
| 443 | 2010310C07Rik | 0.9335 | 0.0168455 |
| 444 | Mmp2 | 0.9335 | 0.0138214 |
| 445 | Gcnt2 | 0.9333 | 0.0006532 |
| 446 | Tpd52 | 0.9298 | 1.84E-10 |
| 447 | Aff2 | 0.9248 | 0.0151788 |
| 448 | Antxr2 | 0.9235 | 1.55E-08 |
| 449 | Alox8 | 0.9222 | 0.0141199 |
| 450 | Gzf1 | 0.9214 | 1.66E-12 |
| 451 | Gm10791 | 0.9205 | 0.0122618 |
| 452 | Pnpla1 | 0.9186 | 0.0185659 |
| 453 | Arl5a | 0.9167 | 2.81E-06 |
| 454 | 4921509O07Rik | 0.9153 | 0.0096263 |
| 455 | Prss34 | 0.9150 | 0.0102947 |
| 456 | Ehd1 | 0.9118 | 8.00E-06 |
| 457 | Adrb2 | 0.9105 | 8.86E-08 |
| 458 | Slc22a20 | 0.9100 | 0.0187103 |
| 459 | Ier3 | 0.9093 | 0.0002443 |
| 460 | Cask | 0.9084 | 4.49E-06 |
| 461 | Nfam1 | 0.9067 | 9.79E-06 |
| 462 | Tefm | 0.9065 | 0.0069575 |
| 463 | Adtrp | 0.9065 | 0.0154261 |
| 464 | Ms4a2 | 0.9041 | 0.0040008 |
| 465 | B430306N03Rik | 0.9036 | 3.41E-05 |
| 466 | Egr1 | 0.9026 | 0.0027723 |
| 467 | E130311K13Rik | 0.9006 | 0.0056461 |
| 468 | Col4a2 | 0.8995 | 0.0036261 |
| 469 | Boll | 0.8990 | 0.0213433 |
| 470 | 4930431P03Rik | 0.8989 | 0.0148994 |
| 471 | Lct | 0.8983 | 0.0213902 |
| 472 | Uaca | 0.8967 | 0.021685 |
| 473 | Bcl2a1b | 0.8966 | 7.21E-09 |
| 474 | Slc39a5 | 0.8963 | 0.011264 |
| 475 | Rab27a | 0.8962 | 2.06E-06 |
| 476 | Tirap | 0.8960 | 9.34E-08 |
| 477 | Ephb3 | 0.8935 | 0.0114291 |
| 478 | Frk | 0.8935 | 0.0114291 |
| 479 | Gcnt1 | 0.8923 | 5.48E-07 |
| 480 | Dock4 | 0.8921 | 4.47E-05 |
| 481 | Lyrm7 | 0.8920 | 0.0223301 |
| 482 | Mir6935 | 0.8905 | 0.0219597 |
| 483 | E530011L22Rik | 0.8867 | 0.0115727 |
| 484 | Drc1 | 0.8863 | 0.003559 |
| 485 | Mpo | 0.8833 | 0.0194151 |
| 486 | 1700029H14Rik | 0.8831 | 0.0178169 |
| 487 | Slc35a5 | 0.8827 | 6.66E-07 |
| 488 | Ampd3 | 0.8809 | 0.0003977 |
| 489 | Rab11fip4 | 0.8782 | 0.0001571 |
| 490 | 4930519G04Rik | 0.8781 | 0.0178655 |
| 491 | Rnf144a | 0.8768 | 0.0002137 |
| 492 | Zfp819 | 0.8742 | 0.0181842 |
| 493 | Arl11 | 0.8725 | 0.0008624 |
| 494 | Apoa2 | 0.8717 | 0.021887 |
| 495 | Dclre1c | 0.8710 | 2.41E-15 |
| 496 | Amotl2 | 0.8705 | 0.0116502 |
| 497 | Klf5 | 0.8701 | 0.0112423 |
| 498 | Gpr31b | 0.8699 | 0.0226531 |
| 499 | Pnkp | 0.8690 | 3.42E-07 |
| 500 | A530064D06Rik | 0.8685 | 0.0034626 |
| 501 | Cd274 | 0.8674 | 4.52E-09 |
| 502 | Tjp1 | 0.8672 | 0.017687 |
| 503 | Bhlhe40 | 0.8647 | 1.70E-05 |
| 504 | Xdh | 0.8646 | 1.12E-05 |
| 505 | Igfbp4 | 0.8641 | 8.50E-14 |
| 506 | Mgst1 | 0.8636 | 2.12E-05 |
| 507 | Btnl2 | 0.8627 | 0.0172333 |
| 508 | Glipr2 | 0.8616 | 2.29E-16 |
| 509 | Apobr | 0.8615 | 3.10E-06 |
| 510 | Prr5l | 0.8596 | 0.0003864 |
| 511 | Gm17455 | 0.8588 | 0.0277199 |
| 512 | Asb7 | 0.8563 | 1.37E-06 |
| 513 | 9130230L23Rik | 0.8556 | 0.0230057 |
| 514 | 3010026O09Rik | 0.8529 | 0.0081406 |
| 515 | Gabpb1 | 0.8515 | 6.26E-09 |
| 516 | 6530402F18Rik | 0.8512 | 6.84E-06 |
| 517 | F10 | 0.8499 | 5.78E-05 |
| 518 | Itgam | 0.8484 | 5.97E-09 |
| 519 | Ddx60 | 0.8471 | 0.0002578 |
| 520 | Tomm40l | 0.8467 | 0.0006796 |
| 521 | Gtf2ird2 | 0.8461 | 0.0029538 |
| 522 | Nfkbib | 0.8417 | 2.19E-06 |
| 523 | 5430427O19Rik | 0.8395 | 0.0011932 |
| 524 | Tmem132a | 0.8393 | 0.0083447 |
| 525 | Mvd | 0.8381 | 0.0001587 |
| 526 | Itih4 | 0.8375 | 0.0316605 |
| 527 | Mcm4 | 0.8362 | 2.96E-07 |
| 528 | Tnfsf13b | 0.8346 | 0.0008226 |
| 529 | Rhpn1 | 0.8343 | 0.0232494 |
| 530 | Slc28a3 | 0.8340 | 0.0162805 |
| 531 | Vldlr | 0.8332 | 0.0002392 |
| 532 | Dgkh | 0.8330 | 0.0047604 |
| 533 | Tmem110 | 0.8328 | 1.34E-07 |
| 534 | Dpep2 | 0.8326 | 0.0002441 |
| 535 | Mmp27 | 0.8306 | 0.0262904 |
| 536 | Cxcl10 | 0.8303 | 0.0331638 |
| 537 | P2ry2 | 0.8291 | 0.0037636 |
| 538 | Kcnc1 | 0.8289 | 0.0011125 |
| 539 | Ccdc152 | 0.8284 | 0.0292544 |
| 540 | Ncf4 | 0.8267 | 3.35E-06 |
| 541 | Slc31a2 | 0.8257 | 0.0019695 |
| 542 | Ogfrl1 | 0.8248 | 1.35E-15 |
| 543 | Acacb | 0.8247 | 0.0175188 |
| 544 | 5-Sep | 0.8244 | 0.0057517 |
| 545 | Nav2 | 0.8229 | 1.36E-05 |
| 546 | Sox13 | 0.8216 | 0.0043276 |
| 547 | Tnfrsf26 | 0.8212 | 0.0001304 |
| 548 | Ly75 | 0.8206 | 0.0004963 |
| 549 | Pde7b | 0.8202 | 0.0006999 |
| 550 | Cpne3 | 0.8195 | 3.39E-07 |
| 551 | Fignl2 | 0.8190 | 0.0345462 |
| 552 | Btbd19 | 0.8180 | 0.0004632 |
| 553 | Cyp11a1 | 0.8177 | 3.70E-05 |
| 554 | Nacad | 0.8141 | 1.43E-05 |
| 555 | Foxq1 | 0.8136 | 0.0281552 |
| 556 | Cd44 | 0.8124 | 1.74E-11 |
| 557 | Stx17 | 0.8119 | 6.72E-08 |
| 558 | Alyref2 | 0.8103 | 0.0118268 |
| 559 | Cep97 | 0.8097 | 2.68E-07 |
| 560 | Ptchd3 | 0.8092 | 0.0185343 |
| 561 | Osgin1 | 0.8085 | 0.0210328 |
| 562 | Unc13a | 0.8082 | 0.0309697 |
| 563 | Tpbg | 0.8077 | 0.031083 |
| 564 | Ndrg3 | 0.8076 | 6.54E-11 |
| 565 | Daam2 | 0.8059 | 0.0051965 |
| 566 | Lama5 | 0.8038 | 0.0068726 |
| 567 | Tctex1d2 | 0.8030 | 0.0027183 |
| 568 | Cnfn | 0.8023 | 0.0180606 |
| 569 | Capza1 | 0.8017 | 0.0023811 |
| 570 | Dtx3l | 0.8015 | 7.16E-14 |
| 571 | Arsg | 0.7992 | 0.0069075 |
| 572 | Ces2g | 0.7974 | 0.0105923 |
| 573 | Miip | 0.7966 | 3.44E-06 |
| 574 | Oas2 | 0.7932 | 4.87E-05 |
| 575 | Adamts17 | 0.7917 | 0.0209194 |
| 576 | Gm6289 | 0.7917 | 0.0209194 |
| 577 | 4933408B17Rik | 0.7912 | 0.0418203 |
| 578 | Tmco6 | 0.7892 | 2.22E-06 |
| 579 | Aldh3b1 | 0.7891 | 1.91E-05 |
| 580 | Ccdc88b | 0.7885 | 1.42E-05 |
| 581 | Gbp4 | 0.7881 | 1.49E-09 |
| 582 | Ebi3 | 0.7879 | 0.0034019 |
| 583 | Atp13a4 | 0.7862 | 0.0420614 |
| 584 | Mcl1 | 0.7860 | 1.49E-21 |
| 585 | Tgm1 | 0.7856 | 0.0053094 |
| 586 | Plekha4 | 0.7851 | 0.0442584 |
| 587 | Rundc3b | 0.7850 | 0.0152669 |
| 588 | Atxn10 | 0.7850 | 3.96E-15 |
| 589 | Fabp5 | 0.7848 | 0.0051926 |
| 590 | Cd46 | 0.7844 | 0.0116794 |
| 591 | Cpt1a | 0.7841 | 1.89E-09 |
| 592 | Bmp8a | 0.7837 | 0.0386178 |
| 593 | Pira2 | 0.7836 | 0.0420521 |
| 594 | Klra2 | 0.7834 | 0.0023764 |
| 595 | Ptges | 0.7823 | 0.0423511 |
| 596 | Vnn3 | 0.7802 | 0.0452455 |
| 597 | Casc1 | 0.7797 | 0.0437255 |
| 598 | Il17a | 0.7795 | 0.0455568 |
| 599 | Bmpr1a | 0.7778 | 0.0124056 |
| 600 | Tox2 | 0.7778 | 0.0427844 |
| 601 | Rdh1 | 0.7774 | 0.0331266 |
| 602 | Sqrdl | 0.7767 | 7.13E-08 |
| 603 | 2310001H17Rik | 0.7756 | 0.0306664 |
| 604 | Klra17 | 0.7755 | 0.0023737 |
| 605 | Rabgef1 | 0.7732 | 0.0009569 |
| 606 | Tnfsf9 | 0.7724 | 0.0453405 |
| 607 | Gpr82 | 0.7721 | 0.0231443 |
| 608 | Klrg1 | 0.7713 | 0.0077899 |
| 609 | Pde11a | 0.7707 | 0.0375225 |
| 610 | Amica1 | 0.7702 | 8.97E-06 |
| 611 | Cdh1 | 0.7699 | 0.0006813 |
| 612 | Fndc3b | 0.7692 | 2.64E-05 |
| 613 | Arntl2 | 0.7672 | 0.0080861 |
| 614 | Fam21 | 0.7657 | 2.27E-09 |
| 615 | Lrrc3 | 0.7643 | 0.0219646 |
| 616 | Tbc1d2 | 0.7641 | 0.0002306 |
| 617 | Iqgap1 | 0.7628 | 2.17E-07 |
| 618 | Ttc30a1 | 0.7623 | 0.0498123 |
| 619 | Cd8b1 | 0.7615 | 3.27E-06 |
| 620 | LOC100038947 | 0.7601 | 0.0028894 |
| 621 | Ccl3 | 0.7599 | 0.0012861 |
| 622 | Fes | 0.7597 | 3.49E-05 |
| 623 | Serpinb7 | 0.7584 | 0.0231364 |
| 624 | Myadm | 0.7580 | 3.31E-05 |
| 625 | Flot2 | 0.7576 | 1.13E-08 |
| 626 | Slc5a8 | 0.7567 | 0.0250037 |
| 627 | Gpr146 | 0.7558 | 0.0005434 |
| 628 | Gbp3 | 0.7556 | 4.69E-06 |
| 629 | Pilrb1 | 0.7555 | 0.0125013 |
| 630 | Sorl1 | 0.7548 | 0.0009932 |
| 631 | Selplg | 0.7520 | 1.52E-05 |
| 632 | Ntng2 | 0.7515 | 0.0021526 |
| 633 | Cldn1 | 0.7509 | 0.0112954 |
| 634 | Lincrna-cox2 | 0.7507 | 0.0473125 |
| 635 | 1700012B09Rik | 0.7500 | 0.0357978 |
| 636 | Clec4a2 | 0.7489 | 0.0002063 |
| 637 | Col18a1 | 0.7487 | 0.0135704 |
| 638 | Zmpste24 | 0.7483 | 1.31E-06 |
| 639 | Prss22 | 0.7480 | 0.0383793 |
| 640 | P4ha1 | 0.7467 | 9.35E-09 |
| 641 | Mfsd1 | 0.7453 | 3.35E-06 |
| 642 | Orm2 | 0.7447 | 0.0238443 |
| 643 | Kctd12 | 0.7437 | 6.43E-06 |
| 644 | Gm19395 | 0.7435 | 0.049088 |
| 645 | Slco4c1 | 0.7419 | 0.0072972 |
| 646 | Chsy3 | 0.7414 | 0.0474861 |
| 647 | Tal2 | 0.7413 | 0.0472443 |
| 648 | Slc12a5 | 0.7412 | 0.0240834 |
| 649 | Sepn1 | 0.7408 | 0.0012791 |
| 650 | Ppt1 | 0.7403 | 5.96E-13 |
| 651 | Aph1b | 0.7402 | 0.004989 |
| 652 | Slc32a1 | 0.7391 | 0.0415679 |
| 653 | Nfkbie | 0.7387 | 0.0014287 |
| 654 | Mospd4 | 0.7387 | 0.0393163 |
| 655 | Zfp14 | 0.7384 | 0.0198412 |
| 656 | Tmem194 | 0.7365 | 0.0006557 |
| 657 | Rnf19a | 0.7351 | 2.27E-06 |
| 658 | Furin | 0.7350 | 1.79E-08 |
| 659 | Sec22c | 0.7340 | 4.45E-05 |
| 660 | Il13ra1 | 0.7314 | 5.29E-06 |
| 661 | Ckap2l | 0.7307 | 0.0057789 |
| 662 | Upk3bl | 0.7293 | 0.0442959 |
| 663 | Skp2 | 0.7290 | 0.0431309 |
| 664 | Lrrc75b | 0.7289 | 0.0151859 |
| 665 | Pik3ap1 | 0.7277 | 0.0001257 |
| 666 | Ankrd6 | 0.7274 | 0.0400297 |
| 667 | Tet1 | 0.7262 | 0.0449446 |
| 668 | Rps6ka2 | 0.7249 | 0.0007912 |
| 669 | Krt6b | 0.7248 | 0.0286171 |
| 670 | Gm15446 | 0.7242 | 0.0330051 |
| 671 | Fam217b | 0.7219 | 0.0055222 |
| 672 | Ugt1a6b | 0.7218 | 0.028992 |
| 673 | Ms4a8a | 0.7214 | 0.0121554 |
| 674 | Plk3 | 0.7210 | 0.0042362 |
| 675 | Tagap | 0.7209 | 0.0009185 |
| 676 | 6330407A03Rik | 0.7202 | 0.0031857 |
| 677 | Trim30a | 0.7201 | 1.49E-07 |
| 678 | Ptplb | 0.7179 | 0.006237 |
| 679 | Klk10 | 0.7178 | 0.0311942 |
| 680 | Ggt1 | 0.7168 | 0.0003359 |
| 681 | Svil | 0.7160 | 2.31E-08 |
| 682 | Tcn2 | 0.7158 | 0.0001167 |
| 683 | Anxa11 | 0.7157 | 1.96E-06 |
| 684 | 6330416G13Rik | 0.7144 | 1.16E-05 |
| 685 | Tlr1 | 0.7134 | 5.77E-10 |
| 686 | Mpzl3 | 0.7128 | 0.0057038 |
| 687 | Socs1 | 0.7125 | 0.0012413 |
| 688 | Noxred1 | 0.7119 | 0.0484326 |
| 689 | Sri | 0.7101 | 5.71E-07 |
| 690 | Mctp2 | 0.7091 | 2.91E-06 |
| 691 | Ift88 | 0.7079 | 0.0085055 |
| 692 | BC049352 | 0.7062 | 0.0083514 |
| 693 | Ms4a6b | 0.7051 | 0.0012196 |
| 694 | Meig1 | 0.7050 | 0.0484452 |
| 695 | Olfm4 | 0.7024 | 0.0426327 |
| 696 | Cntd1 | 0.7022 | 0.0430575 |
| 697 | Cd53 | 0.7020 | 7.01E-13 |
| 698 | Pigb | 0.7019 | 0.0175649 |
| 699 | Ctnnbip1 | 0.7014 | 0.0017437 |
| 700 | Lrrc63 | 0.7010 | 0.0164314 |
| 701 | Cutc | 0.7009 | 0.006395 |
| 702 | Pla2g7 | 0.7005 | 3.67E-05 |
| 703 | Tcf7 | 0.7003 | 4.45E-09 |
| 704 | Osbpl9 | 0.6990 | 3.98E-12 |
| 705 | Wfdc18 | 0.6981 | 0.0334203 |
| 706 | Plin2 | 0.6974 | 0.0061267 |
| 707 | Pde1b | 0.6951 | 1.26E-09 |
| 708 | 2010111I01Rik | 0.6939 | 7.96E-05 |
| 709 | Spata13 | 0.6928 | 1.14E-08 |
| 710 | Snx20 | 0.6924 | 0.0009512 |
| 711 | Timp2 | 0.6924 | 0.0002744 |
| 712 | Rnpep | 0.6908 | 0.0027529 |
| 713 | Adrb1 | 0.6897 | 0.0191483 |
| 714 | Plin3 | 0.6891 | 0.0009911 |
| 715 | Nudt4 | 0.6873 | 7.04E-05 |
| 716 | Fbxo25 | 0.6871 | 0.0028604 |
| 717 | Cd200r3 | 0.6871 | 0.0410242 |
| 718 | Ptges2 | 0.6866 | 0.0029697 |
| 719 | Jak3 | 0.6866 | 1.06E-09 |
| 720 | Suco | 0.6865 | 3.02E-18 |
| 721 | Wtap | 0.6860 | 1.52E-11 |
| 722 | BC068281 | 0.6856 | 0.0173766 |
| 723 | B3gat2 | 0.6845 | 0.0187913 |
| 724 | Gm12216 | 0.6845 | 0.0312057 |
| 725 | Bphl | 0.6838 | 0.0149023 |
| 726 | Perp | 0.6836 | 0.0291558 |
| 727 | Galm | 0.6828 | 0.0088314 |
| 728 | Csta1 | 0.6825 | 0.0487057 |
| 729 | Sipa1l2 | 0.6821 | 0.0008449 |
| 730 | Zfhx3 | 0.6788 | 0.0002608 |
| 731 | Plp2 | 0.6771 | 0.0004339 |
| 732 | Sowahc | 0.6750 | 0.0046791 |
| 733 | Mcam | 0.6745 | 0.005311 |
| 734 | Pik3c2a | 0.6740 | 0.004402 |
| 735 | Abr | 0.6736 | 0.000109 |
| 736 | G630090E17Rik | 0.6727 | 0.0376735 |
| 737 | Vsig10 | 0.6720 | 0.0079841 |
| 738 | Ap4b1 | 0.6717 | 1.12E-06 |
| 739 | Dennd4a | 0.6714 | 1.07E-06 |
| 740 | Pdlim2 | 0.6706 | 0.001108 |
| 741 | Arap3 | 0.6688 | 0.0001782 |
| 742 | Irak2 | 0.6685 | 0.0031272 |
| 743 | Zc3h12a | 0.6677 | 0.0040459 |
| 744 | Slc35f2 | 0.6668 | 0.0008642 |
| 745 | Mir221 | 0.6665 | 0.0410538 |
| 746 | Zfp185 | 0.6659 | 0.0411333 |
| 747 | Nt5c2 | 0.6654 | 2.14E-08 |
| 748 | Tgm2 | 0.6627 | 0.0001815 |
| 749 | Zfp953 | 0.6609 | 0.0266426 |
| 750 | Xpc | 0.6591 | 0.0002071 |
| 751 | Slc30a4 | 0.6574 | 0.0015272 |
| 752 | Ano10 | 0.6571 | 0.0001436 |
| 753 | 9830107B12Rik | 0.6542 | 0.0068341 |
| 754 | Spryd3 | 0.6529 | 1.31E-06 |
| 755 | Irak3 | 0.6515 | 1.46E-05 |
| 756 | Gm1966 | 0.6506 | 0.0043223 |
| 757 | Gpi1 | 0.6499 | 1.06E-07 |
| 758 | Nadk | 0.6495 | 0.000808 |
| 759 | Atp6v1c2 | 0.6493 | 0.044512 |
| 760 | Olfr183 | 0.6493 | 0.044512 |
| 761 | Picalm | 0.6482 | 1.90E-08 |
| 762 | Kctd17 | 0.6468 | 0.0203154 |
| 763 | Mrps31 | 0.6462 | 0.0057734 |
| 764 | Rbm45 | 0.6455 | 3.37E-06 |
| 765 | Slc25a27 | 0.6450 | 0.0039435 |
| 766 | Slc25a20 | 0.6428 | 0.0004081 |
| 767 | Fuca2 | 0.6426 | 7.63E-05 |
| 768 | Blcap | 0.6423 | 0.0042948 |
| 769 | Flt1 | 0.6411 | 0.0165839 |
| 770 | Hs3st3b1 | 0.6400 | 0.0045919 |
| 771 | Pim2 | 0.6397 | 6.67E-07 |
| 772 | Naprt1 | 0.6393 | 0.0121492 |
| 773 | Prelid2 | 0.6378 | 0.0035437 |
| 774 | Pbx1 | 0.6370 | 0.0049153 |
| 775 | Atg9a | 0.6364 | 2.73E-05 |
| 776 | Clec2d | 0.6356 | 0.00071 |
| 777 | Ugt1a7c | 0.6348 | 0.0111149 |
| 778 | Fam122a | 0.6344 | 0.0069621 |
| 779 | Rab31 | 0.6343 | 2.39E-05 |
| 780 | Irf1 | 0.6330 | 0.000137 |
| 781 | Cnga4 | 0.6322 | 0.0458749 |
| 782 | Tnfsf14 | 0.6312 | 0.0135649 |
| 783 | Mov10 | 0.6299 | 0.0044475 |
| 784 | Rab3d | 0.6297 | 2.50E-06 |
| 785 | Icosl | 0.6297 | 0.0328186 |
| 786 | Gbp6 | 0.6295 | 0.0072955 |
| 787 | Tmprss11g | 0.6286 | 0.0464546 |
| 788 | Stk39 | 0.6280 | 0.0032063 |
| 789 | Atp6v0a1 | 0.6278 | 0.0004627 |
| 790 | Xylt1 | 0.6275 | 0.0106584 |
| 791 | Dapl1 | 0.6270 | 0.0019856 |
| 792 | Tmcc1 | 0.6256 | 0.0011896 |
| 793 | Iqsec1 | 0.6254 | 0.0021304 |
| 794 | Scnn1a | 0.6244 | 0.0079403 |
| 795 | Rasgrp4 | 0.6202 | 0.0005549 |
| 796 | Pmel | 0.6189 | 1.76E-06 |
| 797 | Fam129a | 0.6182 | 0.0009301 |
| 798 | Magi3 | 0.6175 | 0.0136406 |
| 799 | A330023F24Rik | 0.6165 | 0.0322554 |
| 800 | Rlf | 0.6126 | 8.63E-10 |
| 801 | Il10rb | 0.6115 | 2.19E-05 |
| 802 | Prickle3 | 0.6108 | 0.0019466 |
| 803 | Cep19 | 0.6106 | 0.0102671 |
| 804 | AW112010 | 0.6105 | 0.0141684 |
| 805 | Fnip2 | 0.6089 | 0.001163 |
| 806 | Igflr1 | 0.6088 | 0.001833 |
| 807 | Nfu1 | 0.6084 | 0.0122702 |
| 808 | Grm6 | 0.6061 | 0.0285427 |
| 809 | Cd80 | 0.6059 | 6.22E-05 |
| 810 | Cd300a | 0.6045 | 0.0058032 |
| 811 | Sos2 | 0.6038 | 3.33E-06 |
| 812 | Traf3ip1 | 0.6035 | 0.0057448 |
| 813 | Gm8615 | 0.6029 | 0.0005365 |
| 814 | Dram1 | 0.6028 | 0.0413958 |
| 815 | Cma1 | 0.6024 | 0.0122566 |
| 816 | Spi1 | 0.6019 | 0.0040684 |
| 817 | Ciapin1 | 0.5983 | 0.0001855 |
| 818 | Dennd3 | 0.5978 | 0.0125661 |
| 819 | Metrnl | 0.5972 | 0.000161 |
| 820 | Sptlc2 | 0.5953 | 6.41E-07 |
| 821 | Myo1d | 0.5951 | 0.0081059 |
| 822 | Mfsd6 | 0.5948 | 1.23E-07 |
| 823 | Shmt1 | 0.5944 | 0.0064868 |
| 824 | Tsen34 | 0.5938 | 0.0027474 |
| 825 | Bbs4 | 0.5928 | 0.0227038 |
| 826 | Capn1 | 0.5917 | 3.70E-06 |
| 827 | Etohi1 | 0.5916 | 0.0300769 |
| 828 | Tyro3 | 0.5914 | 0.0249621 |
| 829 | Tmem216 | 0.5907 | 0.031011 |
| 830 | Psd | 0.5902 | 0.0145457 |
| 831 | Trdmt1 | 0.5901 | 0.0264143 |
| 832 | Mettl20 | 0.5895 | 0.009616 |
| 833 | Alg1 | 0.5885 | 0.0056841 |
| 834 | Atp1b1 | 0.5873 | 1.47E-05 |
| 835 | Synj1 | 0.5860 | 4.53E-07 |
| 836 | Rxra | 0.5859 | 0.0013526 |
| 837 | Ksr1 | 0.5859 | 0.0002643 |
| 838 | Ero1l | 0.5857 | 6.78E-07 |
| 839 | Gyk | 0.5856 | 0.0001572 |
| 840 | Tagap1 | 0.5851 | 0.0023517 |
| 841 | Rgl2 | 0.5846 | 2.73E-09 |
| 842 | 9930012K11Rik | 0.5844 | 0.021089 |
| 843 | Pnpla7 | 0.5834 | 1.69E-05 |
| 844 | Phf21b | 0.5834 | 0.0134639 |
| 845 | Prdm1 | 0.5833 | 0.0001007 |
| 846 | Golim4 | 0.5826 | 6.62E-06 |
| 847 | Cry1 | 0.5824 | 4.82E-05 |
| 848 | Slc41a1 | 0.5821 | 0.0004623 |
| 849 | Fam160a2 | 0.5817 | 1.14E-05 |
| 850 | Prr7 | 0.5817 | 2.13E-05 |
| 851 | Osm | 0.5815 | 0.0053223 |
| 852 | Etfdh | 0.5803 | 6.73E-06 |
| 853 | Klhdc4 | 0.5796 | 1.85E-05 |
| 854 | Mocs1 | 0.5795 | 0.0012737 |
| 855 | Hmox1 | 0.5791 | 0.0006027 |
| 856 | Depdc1b | 0.5782 | 0.0095198 |
| 857 | Osbpl7 | 0.5779 | 0.0042194 |
| 858 | Lmo4 | 0.5776 | 2.74E-07 |
| 859 | Atp6v1b2 | 0.5775 | 7.94E-05 |
| 860 | A930013F10Rik | 0.5771 | 0.0028732 |
| 861 | Ccnh | 0.5768 | 0.02143 |
| 862 | Pfkfb4 | 0.5766 | 0.0017348 |
| 863 | 3110057O12Rik | 0.5765 | 0.0176167 |
| 864 | Notch1 | 0.5762 | 3.32E-05 |
| 865 | Naa38 | 0.5760 | 0.0204676 |
| 866 | Gpcpd1 | 0.5752 | 2.00E-12 |
| 867 | Cdc42ep3 | 0.5748 | 0.0047735 |
| 868 | Id2 | 0.5746 | 2.83E-05 |
| 869 | Arrdc4 | 0.5744 | 0.0026997 |
| 870 | Hck | 0.5739 | 0.0021018 |
| 871 | Trip4 | 0.5738 | 0.0004888 |
| 872 | Oas3 | 0.5736 | 0.0098002 |
| 873 | Map3k15 | 0.5736 | 0.0442658 |
| 874 | Rab44 | 0.5735 | 8.66E-05 |
| 875 | Hrh2 | 0.5720 | 0.0254047 |
| 876 | Rab8b | 0.5716 | 4.46E-07 |
| 877 | Grina | 0.5704 | 0.0169506 |
| 878 | Cd52 | 0.5694 | 9.27E-05 |
| 879 | Dars2 | 0.5691 | 0.046704 |
| 880 | Sez6l2 | 0.5687 | 0.0263714 |
| 881 | Ddx6 | 0.5679 | 1.55E-08 |
| 882 | Bcl2a1d | 0.5671 | 0.0158412 |
| 883 | Nxn | 0.5665 | 0.006041 |
| 884 | Reep3 | 0.5664 | 0.0003334 |
| 885 | Trex1 | 0.5649 | 0.0013132 |
| 886 | Tspan5 | 0.5646 | 0.003944 |
| 887 | Pbxip1 | 0.5643 | 1.41E-05 |
| 888 | Sh2d1a | 0.5643 | 0.0008089 |
| 889 | Arid5b | 0.5640 | 0.0005311 |
| 890 | Perm1 | 0.5623 | 0.0435328 |
| 891 | Snx14 | 0.5615 | 7.40E-05 |
| 892 | Btg1 | 0.5615 | 1.74E-06 |
| 893 | Rnf157 | 0.5614 | 0.0001991 |
| 894 | AB124611 | 0.5612 | 4.25E-05 |
| 895 | Esm1 | 0.5603 | 0.0254805 |
| 896 | Ngly1 | 0.5598 | 1.19E-06 |
| 897 | 2310015A10Rik | 0.5581 | 0.0018701 |
| 898 | Numb | 0.5575 | 5.83E-06 |
| 899 | Klf11 | 0.5566 | 0.005339 |
| 900 | Haus1 | 0.5549 | 0.0409699 |
| 901 | Nanos1 | 0.5536 | 0.0274348 |
| 902 | Klhl9 | 0.5531 | 0.0001754 |
| 903 | Card9 | 0.5530 | 0.0192472 |
| 904 | Plekhn1 | 0.5530 | 0.0049771 |
| 905 | Atp6v1d | 0.5528 | 1.52E-05 |
| 906 | Lsp1 | 0.5517 | 0.0003988 |
| 907 | Zkscan8 | 0.5501 | 0.0247201 |
| 908 | Mir22hg | 0.5486 | 0.0064409 |
| 909 | Tbc1d30 | 0.5486 | 0.0315215 |
| 910 | Tmem154 | 0.5483 | 0.0023891 |
| 911 | Rbms1 | 0.5476 | 2.10E-05 |
| 912 | Fxyd5 | 0.5463 | 0.0001069 |
| 913 | Tjp3 | 0.5462 | 0.0281442 |
| 914 | Hiatl1 | 0.5454 | 3.44E-05 |
| 915 | App | 0.5438 | 0.0003155 |
| 916 | Fhod1 | 0.5429 | 0.0007927 |
| 917 | Trim30d | 0.5426 | 0.0143783 |
| 918 | Athl1 | 0.5425 | 0.0213153 |
| 919 | Malsu1 | 0.5408 | 0.0073413 |
| 920 | Pgs1 | 0.5405 | 2.36E-05 |
| 921 | Gyg | 0.5386 | 0.0017225 |
| 922 | Mon1b | 0.5386 | 0.0001132 |
| 923 | Eif2b2 | 0.5383 | 4.62E-07 |
| 924 | Casp1 | 0.5380 | 0.0004321 |
| 925 | Crnkl1 | 0.5369 | 9.18E-05 |
| 926 | Fbxo31 | 0.5369 | 0.0084698 |
| 927 | Wdr12 | 0.5363 | 0.0086594 |
| 928 | Zxdc | 0.5359 | 0.0013133 |
| 929 | S1pr4 | 0.5348 | 9.35E-05 |
| 930 | Actb | 0.5342 | 6.44E-05 |
| 931 | Hsd17b11 | 0.5331 | 0.0056019 |
| 932 | Man2a1 | 0.5325 | 7.16E-05 |
| 933 | Neat1 | 0.5313 | 1.28E-07 |
| 934 | 5031414D18Rik | 0.5280 | 0.0025479 |
| 935 | Taf12 | 0.5270 | 0.0011768 |
| 936 | Ipcef1 | 0.5267 | 0.0003257 |
| 937 | Cited2 | 0.5264 | 0.0011732 |
| 938 | Birc3 | 0.5258 | 0.0193699 |
| 939 | Mrps15 | 0.5258 | 0.0354772 |
| 940 | Gcc1 | 0.5257 | 4.04E-05 |
| 941 | Ublcp1 | 0.5247 | 0.0412392 |
| 942 | Dhx32 | 0.5232 | 0.005941 |
| 943 | Tmed8 | 0.5210 | 0.0007437 |
| 944 | Suv39h2 | 0.5191 | 0.0364375 |
| 945 | Peli2 | 0.5190 | 0.0080689 |
| 946 | Cpt2 | 0.5185 | 0.0423921 |
| 947 | Braf | 0.5183 | 0.0001245 |
| 948 | Clec12a | 0.5183 | 0.0006304 |
| 949 | Acpp | 0.5180 | 2.17E-05 |
| 950 | Grin3a | 0.5156 | 0.0334584 |
| 951 | Ccdc53 | 0.5146 | 0.0099163 |
| 952 | Snx27 | 0.5118 | 0.0001599 |
| 953 | 1200014J11Rik | 0.5117 | 0.000102 |
| 954 | Ostf1 | 0.5115 | 2.10E-05 |
| 955 | Mgst2 | 0.5108 | 0.0349913 |
| 956 | Rbmx2 | 0.5105 | 0.046698 |
| 957 | Cdkn2d | 0.5105 | 0.0119269 |
| 958 | Ak2 | 0.5104 | 6.82E-05 |
| 959 | Ccdc43 | 0.5093 | 0.0172438 |
| 960 | Ndst1 | 0.5091 | 0.0010154 |
| 961 | Zhx2 | 0.5088 | 0.0020226 |
| 962 | Thap4 | 0.5084 | 0.0020216 |
| 963 | Ralgapa1 | 0.5082 | 0.0001163 |
| 964 | S100pbp | 0.5074 | 0.0106584 |
| 965 | Ager | 0.5071 | 0.0417038 |
| 966 | Slc35d2 | 0.5060 | 0.0049643 |
| 967 | Hsd11b1 | 0.5059 | 0.0199011 |
| 968 | Sgsh | 0.5057 | 0.0264757 |
| 969 | Stat6 | 0.5051 | 5.96E-05 |
| 970 | Slc11a1 | 0.5037 | 0.0295139 |
| 971 | Mrpl45 | 0.5021 | 0.0162856 |
| 972 | Themis | 0.5015 | 0.0197144 |
| 973 | Osbpl2 | 0.5011 | 0.0002644 |
| 974 | Triobp | 0.5009 | 0.000142 |
| 975 | Tbc1d24 | 0.5008 | 0.0336274 |
| 976 | Lfng | 0.5007 | 0.0007 |
| 977 | Cd244 | 0.5000 | 0.0150039 |
| 978 | Tfdp1 | 0.4995 | 0.0009551 |
| 979 | Pde4b | 0.4991 | 2.88E-05 |
| 980 | Adprm | 0.4990 | 0.013266 |
| 981 | Atp8b4 | 0.4987 | 0.0001979 |
| 982 | Fbxo10 | 0.4980 | 0.0288975 |
| 983 | Fam126b | 0.4980 | 0.0011366 |
| 984 | Asb13 | 0.4980 | 0.0028808 |
| 985 | St3gal4 | 0.4975 | 0.0003204 |
| 986 | Tmco3 | 0.4971 | 0.0273461 |
| 987 | Tpcn2 | 0.4964 | 0.0030243 |
| 988 | Klhdc2 | 0.4956 | 0.0067679 |
| 989 | Slc9a3r1 | 0.4948 | 0.0033107 |
| 990 | Ankzf1 | 0.4912 | 0.0075045 |
| 991 | Nfat5 | 0.4911 | 0.0009581 |
| 992 | Gab2 | 0.4904 | 0.0008484 |
| 993 | Pik3r6 | 0.4885 | 0.0057045 |
| 994 | Utp23 | 0.4882 | 0.0049509 |
| 995 | Ugdh | 0.4880 | 0.0005483 |
| 996 | Tbc1d10c | 0.4878 | 9.44E-06 |
| 997 | Adam19 | 0.4878 | 1.57E-07 |
| 998 | Tlr7 | 0.4877 | 0.0182172 |
| 999 | Pex6 | 0.4868 | 9.28E-05 |
| 1000 | Myo1f | 0.4868 | 0.008804 |
| 1001 | Pstpip1 | 0.4867 | 0.002755 |
| 1002 | Pdcl3 | 0.4859 | 0.0012297 |
| 1003 | Tbc1d14 | 0.4855 | 0.0026665 |
| 1004 | Usb1 | 0.4850 | 0.0012559 |
| 1005 | Gnai3 | 0.4845 | 7.69E-10 |
| 1006 | 5730508B09Rik | 0.4832 | 0.0005277 |
| 1007 | Poc5 | 0.4829 | 0.0329345 |
| 1008 | Ly6a | 0.4820 | 0.0010242 |
| 1009 | Rcbtb1 | 0.4819 | 0.0004859 |
| 1010 | Mbd6 | 0.4810 | 9.37E-05 |
| 1011 | Klhdc1 | 0.4807 | 0.020331 |
| 1012 | Rac2 | 0.4803 | 5.07E-05 |
| 1013 | Calcrl | 0.4799 | 0.0113741 |
| 1014 | Tmem5 | 0.4795 | 0.0214442 |
| 1015 | Rnf169 | 0.4794 | 0.0031186 |
| 1016 | Cd8a | 0.4790 | 0.0118817 |
| 1017 | Mrpl33 | 0.4789 | 0.012499 |
| 1018 | Zfp688 | 0.4786 | 0.0424614 |
| 1019 | Vps4b | 0.4783 | 0.0016589 |
| 1020 | Snrk | 0.4775 | 0.0055541 |
| 1021 | Taf1b | 0.4775 | 0.0043117 |
| 1022 | Dhdds | 0.4774 | 0.0058043 |
| 1023 | Izumo4 | 0.4768 | 0.0102151 |
| 1024 | Ankrd12 | 0.4760 | 0.0143834 |
| 1025 | Plod1 | 0.4758 | 0.00767 |
| 1026 | Agfg1 | 0.4733 | 0.0274583 |
| 1027 | Gbp10 | 0.4731 | 0.0092823 |
| 1028 | Gpc1 | 0.4721 | 0.0376189 |
| 1029 | Nktr | 0.4719 | 0.0001099 |
| 1030 | Ncf2 | 0.4718 | 0.0280229 |
| 1031 | Ntrk3 | 0.4714 | 0.033448 |
| 1032 | Klhdc10 | 0.4710 | 0.0012533 |
| 1033 | Peli1 | 0.4707 | 8.94E-06 |
| 1034 | Raf1 | 0.4704 | 0.0018879 |
| 1035 | Osgin2 | 0.4704 | 0.0209904 |
| 1036 | Nat9 | 0.4699 | 0.023471 |
| 1037 | Gadd45g | 0.4697 | 0.0293911 |
| 1038 | Rab11fip2 | 0.4696 | 0.0216467 |
| 1039 | Aptx | 0.4693 | 0.0366351 |
| 1040 | Tgm4 | 0.4691 | 0.0402773 |
| 1041 | Dusp1 | 0.4678 | 0.0229681 |
| 1042 | Psmb9 | 0.4671 | 0.0004007 |
| 1043 | Dusp6 | 0.4667 | 0.04832 |
| 1044 | Vmp1 | 0.4659 | 0.0029738 |
| 1045 | Supv3l1 | 0.4658 | 0.0018868 |
| 1046 | Rpain | 0.4653 | 0.0339806 |
| 1047 | Cog1 | 0.4652 | 0.0134978 |
| 1048 | Emb | 0.4645 | 2.85E-11 |
| 1049 | Tyrobp | 0.4640 | 0.0320327 |
| 1050 | Mapre3 | 0.4634 | 0.0346715 |
| 1051 | Acp2 | 0.4631 | 0.0196609 |
| 1052 | Cd3d | 0.4627 | 0.0013308 |
| 1053 | Tpr | 0.4623 | 0.0038413 |
| 1054 | Gmfg | 0.4622 | 0.0001329 |
| 1055 | Fam101b | 0.4619 | 0.0023401 |
| 1056 | Eif2b3 | 0.4617 | 0.0387459 |
| 1057 | 2310047M10Rik | 0.4615 | 0.0060027 |
| 1058 | Nt5dc1 | 0.4600 | 0.0021567 |
| 1059 | Cep104 | 0.4590 | 0.0028807 |
| 1060 | Zfyve26 | 0.4577 | 0.0019818 |
| 1061 | Lbr | 0.4563 | 3.55E-08 |
| 1062 | Fnip1 | 0.4561 | 0.0008537 |
| 1063 | Sec61a2 | 0.4559 | 0.0055698 |
| 1064 | Snx11 | 0.4557 | 0.0038491 |
| 1065 | Inpp1 | 0.4552 | 0.0111223 |
| 1066 | Glipr1 | 0.4548 | 0.011632 |
| 1067 | Clip1 | 0.4539 | 2.05E-05 |
| 1068 | Rbm7 | 0.4524 | 0.0006012 |
| 1069 | Serac1 | 0.4522 | 0.028084 |
| 1070 | 2610305D13Rik | 0.4517 | 0.0404848 |
| 1071 | Syt6 | 0.4515 | 0.0123392 |
| 1072 | Cdc73 | 0.4510 | 0.0076902 |
| 1073 | Stam2 | 0.4503 | 0.0102655 |
| 1074 | Srpk2 | 0.4502 | 4.44E-05 |
| 1075 | Cst7 | 0.4501 | 0.0419657 |
| 1076 | Gpr137b-ps | 0.4497 | 0.0210652 |
| 1077 | Tceanc | 0.4493 | 0.0243378 |
| 1078 | Pex3 | 0.4488 | 0.0242019 |
| 1079 | 4930581F22Rik | 0.4476 | 0.0051197 |
| 1080 | Vim | 0.4472 | 0.0004214 |
| 1081 | Ctu1 | 0.4468 | 0.0473596 |
| 1082 | Nfkb2 | 0.4467 | 0.0491541 |
| 1083 | Abcd2 | 0.4464 | 0.0343043 |
| 1084 | Smn1 | 0.4463 | 0.0210546 |
| 1085 | Mrpl39 | 0.4460 | 0.0178647 |
| 1086 | Plagl2 | 0.4454 | 0.036424 |
| 1087 | Uvssa | 0.4452 | 0.008191 |
| 1088 | Abcc10 | 0.4451 | 0.0037343 |
| 1089 | Mroh1 | 0.4451 | 0.0002396 |
| 1090 | Pwp1 | 0.4449 | 0.0038608 |
| 1091 | Heatr1 | 0.4441 | 0.0003955 |
| 1092 | Dab2ip | 0.4439 | 0.0320221 |
| 1093 | Pex2 | 0.4434 | 0.0190695 |
| 1094 | Txnip | 0.4429 | 0.0057117 |
| 1095 | Galnt4 | 0.4425 | 0.0135759 |
| 1096 | Dnajc18 | 0.4416 | 0.0254193 |
| 1097 | Nckap1l | 0.4412 | 0.0009553 |
| 1098 | 8430419L09Rik | 0.4408 | 0.0016023 |
| 1099 | Folr4 | 0.4405 | 0.0247533 |
| 1100 | Sh3bp5 | 0.4404 | 2.21E-07 |
| 1101 | Dnmbp | 0.4403 | 0.021225 |
| 1102 | Rgs11 | 0.4400 | 0.0349247 |
| 1103 | Stard5 | 0.4399 | 0.0452172 |
| 1104 | Phf1 | 0.4394 | 0.0001018 |
| 1105 | Pak2 | 0.4383 | 8.10E-06 |
| 1106 | Rnf34 | 0.4383 | 0.0058812 |
| 1107 | Ctnnb1 | 0.4382 | 0.0236929 |
| 1108 | Ppp1r7 | 0.4382 | 0.0074833 |
| 1109 | Anxa6 | 0.4367 | 0.0021188 |
| 1110 | Fam98b | 0.4366 | 0.0055599 |
| 1111 | Crlf2 | 0.4363 | 4.43E-05 |
| 1112 | Btbd6 | 0.4361 | 0.0343503 |
| 1113 | Zdhhc3 | 0.4361 | 0.0005574 |
| 1114 | Lcp2 | 0.4354 | 0.0013774 |
| 1115 | Mcm3ap | 0.4350 | 6.15E-06 |
| 1116 | Prdm4 | 0.4349 | 0.0015554 |
| 1117 | Foxn2 | 0.4348 | 0.0158286 |
| 1118 | Bhlhb9 | 0.4346 | 0.0311552 |
| 1119 | Larp4b | 0.4342 | 0.0003743 |
| 1120 | Ankrd39 | 0.4326 | 0.0253807 |
| 1121 | Sephs2 | 0.4313 | 0.0031743 |
| 1122 | Gnb4 | 0.4305 | 0.0367464 |
| 1123 | Mcm9 | 0.4304 | 0.0012339 |
| 1124 | Lamtor3 | 0.4297 | 0.008829 |
| 1125 | Fmnl1 | 0.4297 | 0.0138758 |
| 1126 | Edem2 | 0.4285 | 4.23E-05 |
| 1127 | Neu1 | 0.4285 | 0.0008408 |
| 1128 | Ptprc | 0.4284 | 0.0006994 |
| 1129 | Taf9b | 0.4279 | 0.0441419 |
| 1130 | Zbtb17 | 0.4278 | 0.0010929 |
| 1131 | Cpeb4 | 0.4272 | 5.12E-05 |
| 1132 | Nt5e | 0.4268 | 0.0092118 |
| 1133 | Agpat2 | 0.4261 | 0.0114014 |
| 1134 | Ufsp2 | 0.4254 | 0.0143429 |
| 1135 | Phyh | 0.4252 | 0.0278714 |
| 1136 | Carns1 | 0.4248 | 0.000396 |
| 1137 | G6pdx | 0.4243 | 0.0047191 |
| 1138 | Prps1 | 0.4239 | 0.0008664 |
| 1139 | Fam134b | 0.4236 | 0.0097273 |
| 1140 | Sgms1 | 0.4231 | 0.00019 |
| 1141 | Lta4h | 0.4217 | 0.0048907 |
| 1142 | Tbc1d15 | 0.4214 | 2.51E-05 |
| 1143 | Adi1 | 0.4207 | 0.0026382 |
| 1144 | Sft2d2 | 0.4203 | 0.0002325 |
| 1145 | Gmps | 0.4202 | 0.0009644 |
| 1146 | Tgfbi | 0.4201 | 0.0002304 |
| 1147 | Zfp53 | 0.4199 | 0.0105123 |
| 1148 | Scand1 | 0.4198 | 0.0046127 |
| 1149 | Mrpl52 | 0.4197 | 0.0195399 |
| 1150 | Faim | 0.4195 | 0.0146153 |
| 1151 | Map2k4 | 0.4191 | 0.0001884 |
| 1152 | Tgds | 0.4191 | 0.0396877 |
| 1153 | Zfp943 | 0.4189 | 0.0102266 |
| 1154 | Ripk1 | 0.4186 | 0.0034312 |
| 1155 | Fam65b | 0.4182 | 0.0001515 |
| 1156 | Ttc39b | 0.4176 | 0.0131043 |
| 1157 | Edrf1 | 0.4172 | 0.0006434 |
| 1158 | Pde8a | 0.4168 | 0.0043752 |
| 1159 | Ccng2 | 0.4160 | 0.0062202 |
| 1160 | Ppapdc2 | 0.4154 | 0.0257646 |
| 1161 | Msra | 0.4137 | 0.009162 |
| 1162 | Rwdd1 | 0.4127 | 0.0261469 |
| 1163 | Parp9 | 0.4125 | 0.0006048 |
| 1164 | Stard7 | 0.4124 | 0.000874 |
| 1165 | 8030462N17Rik | 0.4123 | 0.0059489 |
| 1166 | Cln5 | 0.4123 | 0.0070468 |
| 1167 | Mtg2 | 0.4110 | 0.0426497 |
| 1168 | Sco1 | 0.4108 | 0.0212288 |
| 1169 | Plcl2 | 0.4106 | 0.0018813 |
| 1170 | Pknox1 | 0.4103 | 0.0059275 |
| 1171 | Ttc7 | 0.4100 | 0.0178994 |
| 1172 | Pag1 | 0.4098 | 0.0082367 |
| 1173 | Cyp4f13 | 0.4097 | 0.0067994 |
| 1174 | Nvl | 0.4091 | 0.0276947 |
| 1175 | Cd1d1 | 0.4089 | 0.0222076 |
| 1176 | Wdr77 | 0.4088 | 0.0170179 |
| 1177 | Trpv2 | 0.4080 | 0.0035622 |
| 1178 | Setd7 | 0.4078 | 0.0001049 |
| 1179 | Eapp | 0.4076 | 0.0043656 |
| 1180 | Srp68 | 0.4075 | 0.0006803 |
| 1181 | Supt20 | 0.4063 | 0.0008169 |
| 1182 | Slc25a16 | 0.4063 | 0.0300794 |
| 1183 | Mical1 | 0.4063 | 0.0169309 |
| 1184 | Mios | 0.4057 | 0.0098113 |
| 1185 | Mul1 | 0.4055 | 0.0022343 |
| 1186 | Jmjd1c | 0.4043 | 0.0492113 |
| 1187 | Prr12 | 0.4042 | 0.0062316 |
| 1188 | Rab8a | 0.4026 | 0.0059513 |
| 1189 | Riok2 | 0.4013 | 0.0022409 |
| 1190 | Eaf1 | 0.4006 | 0.0003562 |
| 1191 | Tspan13 | 0.4003 | 1.07E-06 |
| 1192 | Dnajc10 | 0.3996 | 0.0006202 |
| 1193 | Rnf185 | 0.3992 | 0.0020477 |
| 1194 | Adprh | 0.3987 | 0.0108223 |
| 1195 | 2410131K14Rik | 0.3984 | 0.0414742 |
| 1196 | Cntrl | 0.3964 | 0.0040432 |
| 1197 | Cflar | 0.3959 | 0.0009066 |
| 1198 | Mbnl3 | 0.3958 | 0.0059635 |
| 1199 | Tes | 0.3944 | 0.0001734 |
| 1200 | Adh5 | 0.3944 | 0.013412 |
| 1201 | Pold2 | 0.3929 | 0.0170105 |
| 1202 | Exosc7 | 0.3926 | 0.0341608 |
| 1203 | Gde1 | 0.3916 | 0.0282978 |
| 1204 | Pten | 0.3913 | 0.0003209 |
| 1205 | Mvp | 0.3912 | 0.0055676 |
| 1206 | Dync1li1 | 0.3908 | 0.0029335 |
| 1207 | Cmah | 0.3903 | 0.0090491 |
| 1208 | Tbk1 | 0.3902 | 2.19E-05 |
| 1209 | Casp7 | 0.3889 | 0.0373143 |
| 1210 | Bin3 | 0.3888 | 0.0015376 |
| 1211 | Tbrg1 | 0.3887 | 0.0014176 |
| 1212 | Il16 | 0.3882 | 0.0474948 |
| 1213 | Cass4 | 0.3864 | 0.0265219 |
| 1214 | Tars2 | 0.3863 | 0.0002057 |
| 1215 | Zfp507 | 0.3844 | 0.0051046 |
| 1216 | Hmgb2 | 0.3842 | 0.0441448 |
| 1217 | Zc3h12d | 0.3840 | 0.0286395 |
| 1218 | Gpkow | 0.3840 | 1.78E-05 |
| 1219 | Fcer1g | 0.3839 | 0.0131786 |
| 1220 | Gtf3c3 | 0.3836 | 0.0157183 |
| 1221 | Lrwd1 | 0.3835 | 0.0078616 |
| 1222 | Pgrmc2 | 0.3835 | 0.0335732 |
| 1223 | Txnrd1 | 0.3829 | 0.0009988 |
| 1224 | Lasp1 | 0.3829 | 0.0010401 |
| 1225 | Sigirr | 0.3828 | 0.0050778 |
| 1226 | Epc2 | 0.3826 | 4.27E-05 |
| 1227 | Sema4f | 0.3824 | 0.023886 |
| 1228 | Armc8 | 0.3823 | 0.0023969 |
| 1229 | Ralgps1 | 0.3820 | 0.0217786 |
| 1230 | Rpap3 | 0.3818 | 0.0211858 |
| 1231 | Wdr36 | 0.3817 | 0.0485866 |
| 1232 | Pgm1 | 0.3815 | 0.0045686 |
| 1233 | Rcl1 | 0.3815 | 0.0211289 |
| 1234 | Znrf1 | 0.3811 | 0.0004077 |
| 1235 | Rela | 0.3809 | 0.0028848 |
| 1236 | Yme1l1 | 0.3808 | 0.0001432 |
| 1237 | Pigx | 0.3808 | 0.0425907 |
| 1238 | Ccdc71l | 0.3799 | 0.0391428 |
| 1239 | Tbc1d25 | 0.3798 | 0.0320402 |
| 1240 | Sp140 | 0.3797 | 0.0013433 |
| 1241 | Map3k9 | 0.3795 | 0.0049364 |
| 1242 | Iqgap2 | 0.3794 | 0.0044713 |
| 1243 | Ptpn6 | 0.3781 | 0.0351083 |
| 1244 | Ccpg1 | 0.3781 | 0.0077712 |
| 1245 | Atxn1 | 0.3778 | 0.018023 |
| 1246 | Rinl | 0.3776 | 0.0024737 |
| 1247 | Pde12 | 0.3766 | 0.005712 |
| 1248 | Nck1 | 0.3764 | 0.0336603 |
| 1249 | Golga5 | 0.3761 | 0.0020797 |
| 1250 | Sik2 | 0.3756 | 0.0131196 |
| 1251 | Fbxl3 | 0.3745 | 2.34E-05 |
| 1252 | Igf2r | 0.3744 | 0.024105 |
| 1253 | Arl1 | 0.3739 | 0.0028188 |
| 1254 | Sde2 | 0.3739 | 0.0061486 |
| 1255 | Cd3e | 0.3737 | 0.0003394 |
| 1256 | Gnl3l | 0.3736 | 0.0061847 |
| 1257 | Pdlim5 | 0.3731 | 0.0022911 |
| 1258 | Cd247 | 0.3728 | 0.0026013 |
| 1259 | Arhgef2 | 0.3728 | 0.0402208 |
| 1260 | Nbeal2 | 0.3722 | 0.0090655 |
| 1261 | Ssu72 | 0.3718 | 0.0174018 |
| 1262 | Ttc1 | 0.3715 | 0.0114874 |
| 1263 | Dnajc14 | 0.3709 | 0.0001955 |
| 1264 | Mmadhc | 0.3707 | 0.0028901 |
| 1265 | Prr14l | 0.3705 | 0.0182924 |
| 1266 | Cmip | 0.3701 | 0.0014677 |
| 1267 | Rffl | 0.3695 | 0.0156217 |
| 1268 | Zfyve1 | 0.3693 | 0.0164399 |
| 1269 | Pphln1 | 0.3691 | 0.0075888 |
| 1270 | Tmem170b | 0.3689 | 0.0210746 |
| 1271 | Map3k5 | 0.3688 | 0.0008666 |
| 1272 | Vps36 | 0.3684 | 0.0401552 |
| 1273 | Vasp | 0.3684 | 0.0031298 |
| 1274 | Fbxo18 | 0.3676 | 0.0218569 |
| 1275 | 4930402H24Rik | 0.3675 | 0.0338417 |
| 1276 | Egln1 | 0.3671 | 0.0207679 |
| 1277 | Jund | 0.3670 | 0.0130544 |
| 1278 | Lonp2 | 0.3669 | 0.0009619 |
| 1279 | Cenpl | 0.3669 | 0.0329438 |
| 1280 | Pskh1 | 0.3668 | 0.0074528 |
| 1281 | Tle3 | 0.3655 | 0.0064747 |
| 1282 | Prkaa1 | 0.3654 | 0.0104287 |
| 1283 | Nupl1 | 0.3652 | 0.0198639 |
| 1284 | 7-Mar | 0.3644 | 7.93E-05 |
| 1285 | Stim2 | 0.3639 | 0.0129702 |
| 1286 | Zfp182 | 0.3637 | 0.0307095 |
| 1287 | Rhog | 0.3636 | 0.0055704 |
| 1288 | Rgs19 | 0.3633 | 0.0017059 |
| 1289 | Cd3g | 0.3627 | 0.0028781 |
| 1290 | Parp10 | 0.3627 | 0.0302856 |
| 1291 | Polr3f | 0.3627 | 0.028839 |
| 1292 | Ap3m1 | 0.3625 | 0.0027927 |
| 1293 | Clpx | 0.3623 | 0.0189766 |
| 1294 | Impa1 | 0.3614 | 0.018868 |
| 1295 | Atp6v1h | 0.3611 | 0.0055711 |
| 1296 | Atp7a | 0.3610 | 0.029257 |
| 1297 | Sugt1 | 0.3606 | 0.008209 |
| 1298 | Cluap1 | 0.3605 | 0.0470599 |
| 1299 | Smarca2 | 0.3604 | 2.61E-05 |
| 1300 | Ccser2 | 0.3602 | 0.0170003 |
| 1301 | Zfp687 | 0.3592 | 0.0023581 |
| 1302 | Samhd1 | 0.3586 | 0.0017725 |
| 1303 | Ptpn2 | 0.3585 | 0.0007633 |
| 1304 | BC005624 | 0.3585 | 0.0048667 |
| 1305 | Ldha | 0.3581 | 0.000139 |
| 1306 | Prpf6 | 0.3578 | 0.0006178 |
| 1307 | Atp6v1a | 0.3574 | 0.0292145 |
| 1308 | Mysm1 | 0.3573 | 0.002919 |
| 1309 | Npepps | 0.3568 | 0.0271704 |
| 1310 | Dqx1 | 0.3561 | 0.0469538 |
| 1311 | Stk40 | 0.3561 | 0.0242008 |
| 1312 | Pacs1 | 0.3559 | 4.65E-05 |
| 1313 | Psip1 | 0.3554 | 0.011382 |
| 1314 | Fkbp15 | 0.3549 | 0.008247 |
| 1315 | Vrk1 | 0.3535 | 0.0102394 |
| 1316 | Nmi | 0.3532 | 0.0348548 |
| 1317 | N4bp2l1 | 0.3525 | 0.0226401 |
| 1318 | Sipa1l1 | 0.3516 | 0.0008525 |
| 1319 | Ints12 | 0.3513 | 0.0243297 |
| 1320 | Mboat7 | 0.3511 | 0.0400427 |
| 1321 | Mboat1 | 0.3507 | 0.0415481 |
| 1322 | 2210016L21Rik | 0.3505 | 0.0481553 |
| 1323 | Smchd1 | 0.3502 | 0.000268 |
| 1324 | Lpcat3 | 0.3501 | 0.005802 |
| 1325 | Bloc1s6 | 0.3501 | 0.0038307 |
| 1326 | Kif1b | 0.3498 | 0.0016576 |
| 1327 | Hectd3 | 0.3498 | 0.0018106 |
| 1328 | Cast | 0.3488 | 4.95E-05 |
| 1329 | Zfp276 | 0.3486 | 0.0218499 |
| 1330 | Tnrc6a | 0.3485 | 0.0128237 |
| 1331 | Nek9 | 0.3484 | 8.56E-05 |
| 1332 | Dgka | 0.3484 | 4.93E-05 |
| 1333 | Usp8 | 0.3483 | 5.89E-05 |
| 1334 | D19Bwg1357e | 0.3482 | 0.0057813 |
| 1335 | Kri1 | 0.3480 | 0.0164679 |
| 1336 | Kdm5b | 0.3480 | 0.0046836 |
| 1337 | Cyfip2 | 0.3478 | 0.0129855 |
| 1338 | Cd97 | 0.3473 | 0.0316272 |
| 1339 | Stat3 | 0.3469 | 0.0002923 |
| 1340 | Aldh3a2 | 0.3468 | 0.0090628 |
| 1341 | Arid4b | 0.3456 | 0.0040469 |
| 1342 | Cog8 | 0.3453 | 0.0187492 |
| 1343 | Cct6a | 0.3453 | 0.0001331 |
| 1344 | Kiz | 0.3451 | 0.0155722 |
| 1345 | Ate1 | 0.3450 | 0.0196963 |
| 1346 | Eefsec | 0.3444 | 0.0438118 |
| 1347 | Cers6 | 0.3443 | 0.028973 |
| 1348 | Ppp1r12a | 0.3441 | 0.0002193 |
| 1349 | Rpa2 | 0.3441 | 0.0110422 |
| 1350 | Ip6k1 | 0.3434 | 2.69E-05 |
| 1351 | Klf2 | 0.3431 | 0.0453313 |
| 1352 | Hectd1 | 0.3431 | 0.0322698 |
| 1353 | Rabgap1 | 0.3430 | 0.0083982 |
| 1354 | Sp2 | 0.3430 | 0.0200593 |
| 1355 | Lypla1 | 0.3429 | 0.008832 |
| 1356 | Xpr1 | 0.3429 | 0.0002268 |
| 1357 | Dnajc2 | 0.3428 | 0.032477 |
| 1358 | Zfp639 | 0.3426 | 0.017122 |
| 1359 | Terf1 | 0.3426 | 0.0407863 |
| 1360 | Slc25a30 | 0.3425 | 0.0494228 |
| 1361 | Ube4a | 0.3416 | 0.0250271 |
| 1362 | Ifngr1 | 0.3416 | 0.018341 |
| 1363 | Aqr | 0.3413 | 0.0086271 |
| 1364 | Ddx26b | 0.3411 | 0.0036687 |
| 1365 | Grk6 | 0.3409 | 1.09E-05 |
| 1366 | Ell | 0.3408 | 0.0301911 |
| 1367 | Dennd1c | 0.3407 | 0.0071044 |
| 1368 | Prpf18 | 0.3407 | 0.0104389 |
| 1369 | Exoc7 | 0.3406 | 0.0402936 |
| 1370 | Tnip1 | 0.3396 | 0.0078044 |
| 1371 | Slc20a1 | 0.3392 | 0.0035923 |
| 1372 | Rnf24 | 0.3390 | 0.0352005 |
| 1373 | Xpo6 | 0.3387 | 0.0024564 |
| 1374 | Rab7 | 0.3382 | 0.0011955 |
| 1375 | Irf3 | 0.3381 | 0.0060144 |
| 1376 | Rb1cc1 | 0.3379 | 0.0183099 |
| 1377 | Nedd4l | 0.3373 | 0.0022801 |
| 1378 | Rif1 | 0.3370 | 0.044858 |
| 1379 | Tcea1 | 0.3368 | 0.0223505 |
| 1380 | Flt3l | 0.3361 | 0.0139761 |
| 1381 | Plxnc1 | 0.3355 | 0.0176587 |
| 1382 | Mfsd7b | 0.3355 | 0.0383123 |
| 1383 | Acap2 | 0.3353 | 0.0009735 |
| 1384 | Usp16 | 0.3350 | 0.0013068 |
| 1385 | Slc39a1 | 0.3349 | 0.0113041 |
| 1386 | Chm | 0.3346 | 0.0077164 |
| 1387 | Pdzd8 | 0.3344 | 0.0314599 |
| 1388 | Preb | 0.3341 | 0.0073457 |
| 1389 | Eif1a | 0.3340 | 0.0138598 |
| 1390 | Cpne1 | 0.3337 | 0.0219139 |
| 1391 | Tnip2 | 0.3336 | 0.0069832 |
| 1392 | Acvrl1 | 0.3332 | 0.0490791 |
| 1393 | Ring1 | 0.3331 | 0.0190803 |
| 1394 | Sec23ip | 0.3328 | 0.0125821 |
| 1395 | Tmem127 | 0.3317 | 0.0108087 |
| 1396 | Gbp9 | 0.3310 | 3.39E-05 |
| 1397 | Gpd2 | 0.3300 | 0.0029651 |
| 1398 | Idh3a | 0.3296 | 0.0143738 |
| 1399 | Msl1 | 0.3292 | 0.0168974 |
| 1400 | Gimap3 | 0.3289 | 0.025516 |
| 1401 | Cwf19l2 | 0.3286 | 0.0441863 |
| 1402 | Zdhhc6 | 0.3285 | 0.0392704 |
| 1403 | Ablim1 | 0.3269 | 1.90E-05 |
| 1404 | Sik3 | 0.3255 | 0.0028291 |
| 1405 | Stk35 | 0.3253 | 0.0317841 |
| 1406 | Tmx4 | 0.3252 | 0.0050359 |
| 1407 | Phf20l1 | 0.3249 | 0.0146646 |
| 1408 | Flot1 | 0.3246 | 0.0198817 |
| 1409 | Cspp1 | 0.3246 | 0.0061799 |
| 1410 | Sbno1 | 0.3244 | 0.0055511 |
| 1411 | Agtrap | 0.3237 | 0.0130898 |
| 1412 | Kctd18 | 0.3234 | 0.006883 |
| 1413 | Cstf3 | 0.3225 | 0.0364396 |
| 1414 | Elac2 | 0.3225 | 0.0230379 |
| 1415 | Tnrc6b | 0.3217 | 0.040391 |
| 1416 | Sike1 | 0.3208 | 0.0324406 |
| 1417 | Smc1a | 0.3206 | 0.0091308 |
| 1418 | Usp3 | 0.3206 | 0.0001042 |
| 1419 | Tubb6 | 0.3206 | 0.0479715 |
| 1420 | Ccdc71 | 0.3205 | 0.0373501 |
| 1421 | Ncbp1 | 0.3199 | 0.0016515 |
| 1422 | Ppil4 | 0.3196 | 0.0189564 |
| 1423 | 2610001J05Rik | 0.3194 | 0.0239212 |
| 1424 | Ubxn4 | 0.3188 | 0.0007505 |
| 1425 | Crlf3 | 0.3188 | 0.032775 |
| 1426 | Sec24a | 0.3184 | 0.0079029 |
| 1427 | Aldh4a1 | 0.3182 | 0.0403585 |
| 1428 | Chtf8 | 0.3175 | 0.0065644 |
| 1429 | Cdc40 | 0.3169 | 0.0025044 |
| 1430 | Setd8 | 0.3168 | 0.0373938 |
| 1431 | Chfr | 0.3166 | 0.0050204 |
| 1432 | Got2 | 0.3153 | 0.0031351 |
| 1433 | Galnt7 | 0.3153 | 0.0194365 |
| 1434 | Hcls1 | 0.3152 | 0.0103262 |
| 1435 | Satb1 | 0.3147 | 0.0010619 |
| 1436 | Naa60 | 0.3127 | 0.0212013 |
| 1437 | Exoc3 | 0.3116 | 0.0065628 |
| 1438 | Zdhhc21 | 0.3115 | 0.0186182 |
| 1439 | Skiv2l | 0.3104 | 0.0050349 |
| 1440 | Il4ra | 0.3098 | 0.0053897 |
| 1441 | Smek2 | 0.3095 | 6.74E-05 |
| 1442 | Prkacb | 0.3093 | 0.0224511 |
| 1443 | R3hcc1l | 0.3088 | 0.0179848 |
| 1444 | Plaa | 0.3085 | 0.0186308 |
| 1445 | Nlrc5 | 0.3084 | 2.60E-05 |
| 1446 | Stx6 | 0.3078 | 0.0433406 |
| 1447 | Rc3h2 | 0.3077 | 0.0484595 |
| 1448 | Mier3 | 0.3074 | 0.0220791 |
| 1449 | Slmo2 | 0.3070 | 0.049228 |
| 1450 | Pik3cg | 0.3068 | 0.0276326 |
| 1451 | Leo1 | 0.3067 | 0.0399593 |
| 1452 | Ncoa6 | 0.3064 | 0.0122817 |
| 1453 | Samd4b | 0.3063 | 0.0079291 |
| 1454 | Idua | 0.3055 | 0.0458198 |
| 1455 | Slc25a11 | 0.3050 | 0.0405746 |
| 1456 | Ralb | 0.3045 | 0.0241168 |
| 1457 | D16Ertd472e | 0.3038 | 0.0036773 |
| 1458 | Stat1 | 0.3034 | 0.0023543 |
| 1459 | Sdha | 0.3032 | 0.0048745 |
| 1460 | Dnmt1 | 0.3016 | 0.0015659 |
| 1461 | Tmem164 | 0.3013 | 0.0274956 |
| 1462 | Cdc123 | 0.3007 | 0.0169559 |
| 1463 | Apaf1 | 0.3002 | 0.0257597 |
| 1464 | Atp11b | 0.2993 | 0.0158677 |
| 1465 | Tfe3 | 0.2987 | 0.0490221 |
| 1466 | Apeh | 0.2985 | 0.0091753 |
| 1467 | Rcsd1 | 0.2979 | 0.0008928 |
| 1468 | Fip1l1 | 0.2962 | 0.0100941 |
| 1469 | Exoc1 | 0.2959 | 0.0042346 |
| 1470 | Meis3 | 0.2959 | 0.0310986 |
| 1471 | Ap1m1 | 0.2954 | 0.0163113 |
| 1472 | Ppm1h | 0.2952 | 0.0271416 |
| 1473 | Brms1 | 0.2949 | 0.0067296 |
| 1474 | Myo9b | 0.2946 | 0.0156158 |
| 1475 | Cpsf4 | 0.2943 | 0.0234132 |
| 1476 | Bcl2l11 | 0.2940 | 0.008491 |
| 1477 | Parp4 | 0.2939 | 0.0017674 |
| 1478 | Scfd1 | 0.2939 | 0.029702 |
| 1479 | Crtc3 | 0.2938 | 0.0008207 |
| 1480 | Lrrfip1 | 0.2931 | 0.0335983 |
| 1481 | Dhx8 | 0.2918 | 0.0075129 |
| 1482 | Ccnk | 0.2917 | 0.0062102 |
| 1483 | Polr2a | 0.2917 | 0.0361557 |
| 1484 | Ctr9 | 0.2917 | 0.0237274 |
| 1485 | Kras | 0.2914 | 0.0303595 |
| 1486 | Pigo | 0.2906 | 0.0149221 |
| 1487 | Nisch | 0.2903 | 0.0177796 |
| 1488 | Zfp384 | 0.2900 | 0.014834 |
| 1489 | Pdcd10 | 0.2899 | 0.0409582 |
| 1490 | Tex264 | 0.2896 | 0.0383933 |
| 1491 | 2-Mar | 0.2896 | 0.0326148 |
| 1492 | Eif4ebp2 | 0.2894 | 0.0009829 |
| 1493 | Hnrnpul1 | 0.2890 | 0.0015839 |
| 1494 | Dpf2 | 0.2887 | 0.0432675 |
| 1495 | Tm9sf2 | 0.2887 | 0.0060439 |
| 1496 | Wbp1l | 0.2886 | 0.0016859 |
| 1497 | Sec23b | 0.2883 | 0.0302806 |
| 1498 | Aaas | 0.2875 | 0.0449722 |
| 1499 | Slc4a1ap | 0.2870 | 0.0331264 |
| 1500 | Akap11 | 0.2866 | 0.039292 |
| 1501 | Stt3b | 0.2866 | 0.0050344 |
| 1502 | Frs2 | 0.2866 | 0.0344659 |
| 1503 | Fbrs | 0.2864 | 0.0491185 |
| 1504 | Ap1g2 | 0.2864 | 0.0030424 |
| 1505 | Map4 | 0.2864 | 0.0017763 |
| 1506 | Traf6 | 0.2863 | 0.00178 |
| 1507 | Ap5z1 | 0.2862 | 0.0417926 |
| 1508 | Wdr20 | 0.2862 | 0.0297153 |
| 1509 | Orai2 | 0.2859 | 0.0016989 |
| 1510 | Rps6ka3 | 0.2847 | 0.0078117 |
| 1511 | Acsl5 | 0.2839 | 0.0377397 |
| 1512 | Kcnab2 | 0.2838 | 0.0192325 |
| 1513 | Slc6a6 | 0.2838 | 0.0429447 |
| 1514 | Ccdc55 | 0.2831 | 0.0225174 |
| 1515 | Oxsr1 | 0.2820 | 0.0059182 |
| 1516 | Tmem106b | 0.2818 | 0.0456131 |
| 1517 | Fbxw7 | 0.2815 | 0.0130896 |
| 1518 | Zscan26 | 0.2811 | 0.033064 |
| 1519 | Gabpa | 0.2808 | 0.0173064 |
| 1520 | Scyl2 | 0.2806 | 0.0097247 |
| 1521 | Psma6 | 0.2801 | 0.0097695 |
| 1522 | Cbx7 | 0.2799 | 0.0231514 |
| 1523 | Zranb2 | 0.2787 | 0.0314057 |
| 1524 | Uba2 | 0.2785 | 0.003876 |
| 1525 | Dr1 | 0.2783 | 0.014273 |
| 1526 | Zdhhc5 | 0.2775 | 0.0037125 |
| 1527 | Prkd3 | 0.2774 | 0.0036491 |
| 1528 | Nhlrc2 | 0.2773 | 0.0497834 |
| 1529 | Rap2c | 0.2763 | 0.013952 |
| 1530 | Tnfrsf18 | 0.2756 | 0.0488957 |
| 1531 | Cdadc1 | 0.2753 | 0.0292988 |
| 1532 | Mapkapk2 | 0.2749 | 0.012959 |
| 1533 | Cyba | 0.2728 | 0.0056282 |
| 1534 | Ip6k2 | 0.2725 | 0.0230331 |
| 1535 | Wdr1 | 0.2723 | 0.0156426 |
| 1536 | Coro1a | 0.2717 | 0.0454564 |
| 1537 | Actr3 | 0.2711 | 0.0009171 |
| 1538 | Gpr183 | 0.2711 | 0.042969 |
| 1539 | Gdap2 | 0.2707 | 0.028991 |
| 1540 | Fam134a | 0.2700 | 0.028304 |
| 1541 | Supt6 | 0.2696 | 0.0056947 |
| 1542 | Snrnp200 | 0.2688 | 0.0224032 |
| 1543 | Parg | 0.2684 | 0.0243583 |
| 1544 | Ufd1l | 0.2682 | 0.0319465 |
| 1545 | Pydc3 | 0.2673 | 0.0450849 |
| 1546 | Slamf6 | 0.2673 | 0.0293731 |
| 1547 | Il27ra | 0.2672 | 0.0231709 |
| 1548 | Copa | 0.2660 | 0.0045408 |
| 1549 | 4632428N05Rik | 0.2656 | 0.0398382 |
| 1550 | Txlng | 0.2656 | 0.0324488 |
| 1551 | Mtap | 0.2649 | 0.0308028 |
| 1552 | Prpf40a | 0.2646 | 0.0136653 |
| 1553 | 5830418K08Rik | 0.2644 | 0.0352706 |
| 1554 | Hdac2 | 0.2640 | 0.0461449 |
| 1555 | Twf2 | 0.2636 | 0.0161392 |

**Supplementary Table 4.** Downregulated genes in PBMCs from IS mice.

|  | Gene | log2 Fold Change | p value |
| --- | --- | --- | --- |
| 1 | Siglech | -3.2020 | 0.0000 |
| 2 | Cd209a | -3.0981 | 0.0000 |
| 3 | Wnt11 | -2.9125 | 0.0000 |
| 4 | H2-Ea-ps | -2.6432 | 0.0000 |
| 5 | Epha2 | -2.5717 | 0.0000 |
| 6 | Cd209d | -2.4734 | 0.0000 |
| 7 | Mybpc2 | -2.3985 | 0.0000 |
| 8 | Ccr3 | -2.3957 | 0.0000 |
| 9 | Mrc1 | -2.1400 | 0.0000 |
| 10 | Klk1 | -2.0505 | 0.0000 |
| 11 | Clec10a | -1.9730 | 0.0000 |
| 12 | Mkx | -1.9584 | 0.0000 |
| 13 | Cd209c | -1.8916 | 0.0000 |
| 14 | Arhgef10 | -1.7455 | 0.0000 |
| 15 | Pdxk | -1.7297 | 0.0000 |
| 16 | Alox15 | -1.6885 | 0.0000 |
| 17 | Siglec1 | -1.6564 | 0.0000 |
| 18 | Cd300c | -1.6407 | 0.0000 |
| 19 | 1810011H11Rik | -1.6238 | 0.0000 |
| 20 | Fam83d | -1.6220 | 0.0000 |
| 21 | Fgfr1 | -1.6095 | 0.0000 |
| 22 | Spib | -1.6071 | 0.0000 |
| 23 | Cadm1 | -1.5771 | 0.0000 |
| 24 | Lpl | -1.5713 | 0.0000 |
| 25 | Ifi44 | -1.5597 | 0.0000 |
| 26 | Ptprf | -1.5476 | 0.0000 |
| 27 | 5430437J10Rik | -1.5374 | 0.0000 |
| 28 | Hvcn1 | -1.4986 | 0.0000 |
| 29 | Ciart | -1.4975 | 0.0000 |
| 30 | Mthfr | -1.4948 | 0.0000 |
| 31 | Nr4a3 | -1.4943 | 0.0000 |
| 32 | Nr4a2 | -1.4828 | 0.0000 |
| 33 | Chdh | -1.4784 | 0.0000 |
| 34 | Chst3 | -1.4608 | 0.0000 |
| 35 | Soga1 | -1.4475 | 0.0000 |
| 36 | Pmvk | -1.4467 | 0.0000 |
| 37 | Rtn1 | -1.4441 | 0.0002 |
| 38 | Dos | -1.4438 | 0.0000 |
| 39 | Reps2 | -1.4398 | 0.0000 |
| 40 | Fcer2a | -1.4386 | 0.0000 |
| 41 | Map3k6 | -1.4378 | 0.0000 |
| 42 | Naaladl1 | -1.4249 | 0.0002 |
| 43 | Inpp5j | -1.4085 | 0.0000 |
| 44 | Sema4c | -1.3942 | 0.0000 |
| 45 | Irf4 | -1.3937 | 0.0000 |
| 46 | Aif1l | -1.3891 | 0.0002 |
| 47 | Rasgrp3 | -1.3874 | 0.0000 |
| 48 | Dnajb13 | -1.3820 | 0.0000 |
| 49 | Peg10 | -1.3780 | 0.0000 |
| 50 | Mx1 | -1.3766 | 0.0000 |
| 51 | Gpr157 | -1.3599 | 0.0000 |
| 52 | Ftsj2 | -1.3569 | 0.0003 |
| 53 | Epx | -1.3547 | 0.0003 |
| 54 | Upb1 | -1.3542 | 0.0003 |
| 55 | Gm12253 | -1.3524 | 0.0005 |
| 56 | Eps8 | -1.3508 | 0.0003 |
| 57 | Clec9a | -1.3455 | 0.0000 |
| 58 | Kcnip2 | -1.3421 | 0.0000 |
| 59 | Cds1 | -1.3403 | 0.0000 |
| 60 | Kmo | -1.3329 | 0.0001 |
| 61 | Angptl7 | -1.3321 | 0.0006 |
| 62 | Cyp51 | -1.3188 | 0.0000 |
| 63 | Ldlr | -1.3177 | 0.0000 |
| 64 | Clvs1 | -1.3150 | 0.0007 |
| 65 | Hes1 | -1.3109 | 0.0000 |
| 66 | Tnfrsf13c | -1.3009 | 0.0000 |
| 67 | Marco | -1.2938 | 0.0006 |
| 68 | Gmnn | -1.2898 | 0.0000 |
| 69 | Tob2 | -1.2876 | 0.0000 |
| 70 | H2-DMb1 | -1.2809 | 0.0001 |
| 71 | Cybrd1 | -1.2767 | 0.0000 |
| 72 | BC033916 | -1.2764 | 0.0004 |
| 73 | Zfp652os | -1.2739 | 0.0000 |
| 74 | Fndc4 | -1.2727 | 0.0010 |
| 75 | Plxdc1 | -1.2711 | 0.0001 |
| 76 | Sit1 | -1.2684 | 0.0000 |
| 77 | Havcr1 | -1.2676 | 0.0011 |
| 78 | Lrrc16a | -1.2616 | 0.0000 |
| 79 | Ifi44l | -1.2605 | 0.0000 |
| 80 | Smo | -1.2370 | 0.0009 |
| 81 | Nrarp | -1.2351 | 0.0002 |
| 82 | Notch3 | -1.2343 | 0.0015 |
| 83 | Bank1 | -1.2097 | 0.0001 |
| 84 | Smpd3 | -1.2096 | 0.0017 |
| 85 | Pde6h | -1.2077 | 0.0000 |
| 86 | Hist1h4a | -1.2070 | 0.0020 |
| 87 | Myh6 | -1.2063 | 0.0009 |
| 88 | Cd160 | -1.2043 | 0.0001 |
| 89 | Tmem176b | -1.2009 | 0.0000 |
| 90 | Phf11d | -1.1820 | 0.0001 |
| 91 | Arhgef25 | -1.1819 | 0.0025 |
| 92 | Smad7 | -1.1778 | 0.0000 |
| 93 | Stau2 | -1.1736 | 0.0001 |
| 94 | Bmf | -1.1663 | 0.0000 |
| 95 | Fads3 | -1.1641 | 0.0017 |
| 96 | Pla2g3 | -1.1581 | 0.0030 |
| 97 | Garnl3 | -1.1564 | 0.0018 |
| 98 | Cd163 | -1.1564 | 0.0001 |
| 99 | Kcnh4 | -1.1561 | 0.0031 |
| 100 | Xcl1 | -1.1536 | 0.0004 |
| 101 | Hist1h4h | -1.1481 | 0.0004 |
| 102 | Insig1 | -1.1458 | 0.0000 |
| 103 | Cacna1i | -1.1453 | 0.0002 |
| 104 | Serpina11 | -1.1379 | 0.0019 |
| 105 | Tmem140 | -1.1368 | 0.0003 |
| 106 | Sh2d2a | -1.1290 | 0.0000 |
| 107 | Slc26a10 | -1.1273 | 0.0000 |
| 108 | Fam71a | -1.1257 | 0.0028 |
| 109 | Ly6c2 | -1.1223 | 0.0000 |
| 110 | Hdac11 | -1.1159 | 0.0037 |
| 111 | Samd4 | -1.1134 | 0.0011 |
| 112 | Bcl11a | -1.1113 | 0.0003 |
| 113 | 4930486L24Rik | -1.1108 | 0.0001 |
| 114 | Il7 | -1.1044 | 0.0040 |
| 115 | Psd3 | -1.1025 | 0.0000 |
| 116 | Sstr4 | -1.1019 | 0.0012 |
| 117 | 2810055G20Rik | -1.1007 | 0.0045 |
| 118 | Rnf150 | -1.0990 | 0.0013 |
| 119 | Gm16853 | -1.0972 | 0.0042 |
| 120 | Atp2a1 | -1.0963 | 0.0000 |
| 121 | Sult2b1 | -1.0956 | 0.0007 |
| 122 | Mycl | -1.0929 | 0.0044 |
| 123 | Scd1 | -1.0877 | 0.0001 |
| 124 | Rasa4 | -1.0870 | 0.0000 |
| 125 | Sox4 | -1.0862 | 0.0032 |
| 126 | Sdc3 | -1.0814 | 0.0000 |
| 127 | C920021L13Rik | -1.0798 | 0.0020 |
| 128 | Lphn3 | -1.0745 | 0.0056 |
| 129 | Alpk2 | -1.0720 | 0.0022 |
| 130 | Cd83 | -1.0711 | 0.0003 |
| 131 | Serpinf1 | -1.0703 | 0.0060 |
| 132 | H2-Oa | -1.0701 | 0.0015 |
| 133 | Axl | -1.0678 | 0.0010 |
| 134 | A930024E05Rik | -1.0670 | 0.0000 |
| 135 | Thsd7b | -1.0656 | 0.0037 |
| 136 | Hivep3 | -1.0648 | 0.0002 |
| 137 | Mest | -1.0648 | 0.0000 |
| 138 | Slc1a4 | -1.0565 | 0.0005 |
| 139 | Blnk | -1.0561 | 0.0003 |
| 140 | Gpr3 | -1.0546 | 0.0007 |
| 141 | Aif1 | -1.0546 | 0.0002 |
| 142 | Sema7a | -1.0516 | 0.0000 |
| 143 | Enkur | -1.0487 | 0.0067 |
| 144 | Arpp21 | -1.0483 | 0.0056 |
| 145 | Rac3 | -1.0481 | 0.0073 |
| 146 | Sdcbp2 | -1.0471 | 0.0002 |
| 147 | Ctnnd2 | -1.0470 | 0.0072 |
| 148 | Renbp | -1.0448 | 0.0000 |
| 149 | Nyx | -1.0431 | 0.0012 |
| 150 | 4931429I11Rik | -1.0419 | 0.0000 |
| 151 | Yap1 | -1.0412 | 0.0000 |
| 152 | Snhg12 | -1.0402 | 0.0000 |
| 153 | 2310034O05Rik | -1.0395 | 0.0007 |
| 154 | Tspan18 | -1.0374 | 0.0001 |
| 155 | Hist1h2bc | -1.0362 | 0.0009 |
| 156 | Ciita | -1.0318 | 0.0004 |
| 157 | Sec61g | -1.0300 | 0.0003 |
| 158 | 1810058I24Rik | -1.0294 | 0.0000 |
| 159 | 2900026A02Rik | -1.0289 | 0.0000 |
| 160 | Hspa1b | -1.0288 | 0.0070 |
| 161 | Btbd8 | -1.0285 | 0.0008 |
| 162 | Pltp | -1.0251 | 0.0014 |
| 163 | Scd2 | -1.0168 | 0.0000 |
| 164 | Gria3 | -1.0164 | 0.0036 |
| 165 | Gchfr | -1.0160 | 0.0003 |
| 166 | Cat | -1.0131 | 0.0002 |
| 167 | St6galnac3 | -1.0099 | 0.0006 |
| 168 | Dhrs11 | -1.0096 | 0.0013 |
| 169 | Cadm3 | -1.0090 | 0.0097 |
| 170 | Zfp521 | -1.0072 | 0.0059 |
| 171 | Cxcr5 | -1.0058 | 0.0046 |
| 172 | Serpinh1 | -1.0047 | 0.0101 |
| 173 | Alkbh2 | -1.0043 | 0.0007 |
| 174 | Hpn | -1.0026 | 0.0019 |
| 175 | Ifi205 | -1.0009 | 0.0060 |
| 176 | Tuba3a | -1.0008 | 0.0075 |
| 177 | Mafb | -0.9992 | 0.0000 |
| 178 | Kcnc3 | -0.9991 | 0.0009 |
| 179 | Thbd | -0.9974 | 0.0000 |
| 180 | Rab30 | -0.9971 | 0.0016 |
| 181 | Il5ra | -0.9970 | 0.0019 |
| 182 | Mvk | -0.9955 | 0.0000 |
| 183 | Atf5 | -0.9938 | 0.0005 |
| 184 | Itgb5 | -0.9930 | 0.0000 |
| 185 | Gm11978 | -0.9917 | 0.0012 |
| 186 | Acer2 | -0.9914 | 0.0002 |
| 187 | Pcx | -0.9913 | 0.0001 |
| 188 | Fzd7 | -0.9905 | 0.0001 |
| 189 | Cd72 | -0.9897 | 0.0001 |
| 190 | Kcnb1 | -0.9893 | 0.0050 |
| 191 | Klk1b27 | -0.9864 | 0.0108 |
| 192 | Selm | -0.9843 | 0.0012 |
| 193 | Zfyve28 | -0.9840 | 0.0000 |
| 194 | C330013E15Rik | -0.9821 | 0.0057 |
| 195 | Tmem86a | -0.9817 | 0.0000 |
| 196 | 2810442I21Rik | -0.9817 | 0.0107 |
| 197 | Hspg2 | -0.9816 | 0.0096 |
| 198 | Gm4827 | -0.9805 | 0.0006 |
| 199 | Chad | -0.9797 | 0.0072 |
| 200 | Tspan17 | -0.9793 | 0.0002 |
| 201 | Sdc1 | -0.9789 | 0.0075 |
| 202 | Zfp667 | -0.9735 | 0.0001 |
| 203 | Mrps6 | -0.9726 | 0.0001 |
| 204 | Hist1h2ae | -0.9695 | 0.0119 |
| 205 | Dmpk | -0.9692 | 0.0131 |
| 206 | 1700124L16Rik | -0.9683 | 0.0099 |
| 207 | Gad1 | -0.9681 | 0.0009 |
| 208 | Ttc16 | -0.9678 | 0.0034 |
| 209 | Batf3 | -0.9667 | 0.0118 |
| 210 | 1110032A03Rik | -0.9665 | 0.0010 |
| 211 | Prkag2os1 | -0.9664 | 0.0133 |
| 212 | Cerk | -0.9606 | 0.0000 |
| 213 | Kynu | -0.9599 | 0.0021 |
| 214 | Kcnk5 | -0.9584 | 0.0026 |
| 215 | Col15a1 | -0.9567 | 0.0022 |
| 216 | Plod2 | -0.9561 | 0.0065 |
| 217 | Msmo1 | -0.9550 | 0.0002 |
| 218 | 1110019D14Rik | -0.9548 | 0.0144 |
| 219 | Hist2h2bb | -0.9516 | 0.0085 |
| 220 | Slx1b | -0.9511 | 0.0000 |
| 221 | Ube2l6 | -0.9501 | 0.0024 |
| 222 | A930003A15Rik | -0.9473 | 0.0097 |
| 223 | Specc1 | -0.9446 | 0.0007 |
| 224 | Atp8a2 | -0.9424 | 0.0062 |
| 225 | Siglec5 | -0.9407 | 0.0005 |
| 226 | F8 | -0.9401 | 0.0119 |
| 227 | 1700020I14Rik | -0.9391 | 0.0008 |
| 228 | Fkbp1a | -0.9388 | 0.0000 |
| 229 | Cdkl1 | -0.9378 | 0.0040 |
| 230 | 2810459M11Rik | -0.9377 | 0.0093 |
| 231 | Kcnn4 | -0.9371 | 0.0000 |
| 232 | Fcgr1 | -0.9362 | 0.0107 |
| 233 | Slc16a7 | -0.9358 | 0.0016 |
| 234 | Hbq1b | -0.9358 | 0.0041 |
| 235 | Fam213a | -0.9356 | 0.0048 |
| 236 | Nrgn | -0.9351 | 0.0005 |
| 237 | Hrh1 | -0.9343 | 0.0064 |
| 238 | Serpine1 | -0.9334 | 0.0158 |
| 239 | Vpreb3 | -0.9333 | 0.0114 |
| 240 | Sik1 | -0.9320 | 0.0000 |
| 241 | Tmem176a | -0.9318 | 0.0065 |
| 242 | Tnn | -0.9316 | 0.0007 |
| 243 | Ankrd45 | -0.9296 | 0.0146 |
| 244 | Ly6i | -0.9294 | 0.0144 |
| 245 | Tfdp2 | -0.9277 | 0.0019 |
| 246 | Ccdc122 | -0.9271 | 0.0064 |
| 247 | Flt3 | -0.9268 | 0.0006 |
| 248 | Col3a1 | -0.9265 | 0.0108 |
| 249 | Rab3a | -0.9262 | 0.0042 |
| 250 | Gm19557 | -0.9260 | 0.0000 |
| 251 | Nhlrc4 | -0.9248 | 0.0143 |
| 252 | BC031361 | -0.9237 | 0.0005 |
| 253 | Fam65c | -0.9237 | 0.0003 |
| 254 | Bpgm | -0.9234 | 0.0044 |
| 255 | Gpx1 | -0.9226 | 0.0006 |
| 256 | Fam46c | -0.9224 | 0.0019 |
| 257 | Prkaa2 | -0.9220 | 0.0007 |
| 258 | Mdga1 | -0.9216 | 0.0001 |
| 259 | Apoe | -0.9216 | 0.0000 |
| 260 | Npas4 | -0.9196 | 0.0175 |
| 261 | Lancl3 | -0.9187 | 0.0072 |
| 262 | Slc13a3 | -0.9179 | 0.0186 |
| 263 | Sds | -0.9176 | 0.0180 |
| 264 | AK129341 | -0.9170 | 0.0056 |
| 265 | Rap1gap | -0.9165 | 0.0175 |
| 266 | Siglecg | -0.9156 | 0.0004 |
| 267 | Hist1h2bn | -0.9154 | 0.0014 |
| 268 | Cd74 | -0.9152 | 0.0005 |
| 269 | Mmd | -0.9150 | 0.0005 |
| 270 | Myo1e | -0.9145 | 0.0008 |
| 271 | Lta | -0.9144 | 0.0026 |
| 272 | Plcl1 | -0.9140 | 0.0161 |
| 273 | Fzd4 | -0.9137 | 0.0000 |
| 274 | Pdgfrb | -0.9132 | 0.0061 |
| 275 | Rassf1 | -0.9129 | 0.0000 |
| 276 | Hepacam2 | -0.9085 | 0.0157 |
| 277 | Nr3c2 | -0.9071 | 0.0194 |
| 278 | Ccdc80 | -0.9070 | 0.0183 |
| 279 | Rnase6 | -0.9068 | 0.0002 |
| 280 | 1810020O05Rik | -0.9063 | 0.0109 |
| 281 | Tmem158 | -0.9059 | 0.0006 |
| 282 | Ebf1 | -0.9048 | 0.0021 |
| 283 | Glrx5 | -0.9029 | 0.0041 |
| 284 | Gpr162 | -0.9027 | 0.0208 |
| 285 | Slc9c1 | -0.9019 | 0.0202 |
| 286 | Cd22 | -0.9014 | 0.0035 |
| 287 | Gabarapl2 | -0.8999 | 0.0008 |
| 288 | Faim3 | -0.8983 | 0.0049 |
| 289 | Cxx1c | -0.8971 | 0.0007 |
| 290 | Snx29 | -0.8970 | 0.0000 |
| 291 | H19 | -0.8968 | 0.0078 |
| 292 | Srpk3 | -0.8968 | 0.0038 |
| 293 | Idi1 | -0.8954 | 0.0034 |
| 294 | Abcc2 | -0.8952 | 0.0055 |
| 295 | Ggn | -0.8948 | 0.0016 |
| 296 | Cyp27a1 | -0.8944 | 0.0000 |
| 297 | Ccr8 | -0.8941 | 0.0122 |
| 298 | Tspan9 | -0.8933 | 0.0001 |
| 299 | Upk3b | -0.8919 | 0.0079 |
| 300 | Prdx6 | -0.8915 | 0.0004 |
| 301 | Slc41a2 | -0.8911 | 0.0017 |
| 302 | Tspan2 | -0.8910 | 0.0006 |
| 303 | Gm10768 | -0.8908 | 0.0225 |
| 304 | Ahr | -0.8895 | 0.0000 |
| 305 | Ptpn5 | -0.8892 | 0.0214 |
| 306 | Rnf122 | -0.8878 | 0.0003 |
| 307 | Atpif1 | -0.8852 | 0.0011 |
| 308 | Akr1b8 | -0.8848 | 0.0135 |
| 309 | Rab39 | -0.8843 | 0.0235 |
| 310 | Slc3a2 | -0.8821 | 0.0000 |
| 311 | Spns3 | -0.8820 | 0.0177 |
| 312 | Vat1 | -0.8811 | 0.0000 |
| 313 | Gapt | -0.8808 | 0.0000 |
| 314 | Mkrn1 | -0.8804 | 0.0023 |
| 315 | Dcaf12 | -0.8804 | 0.0022 |
| 316 | Dap | -0.8803 | 0.0003 |
| 317 | Tgfb2 | -0.8799 | 0.0199 |
| 318 | Rnf10 | -0.8793 | 0.0057 |
| 319 | Pik3c2b | -0.8790 | 0.0000 |
| 320 | Stk3 | -0.8770 | 0.0012 |
| 321 | Mef2c | -0.8768 | 0.0000 |
| 322 | Tmem150c | -0.8765 | 0.0038 |
| 323 | Gm8801 | -0.8759 | 0.0094 |
| 324 | Cbfa2t3 | -0.8756 | 0.0000 |
| 325 | Ppp1r14a | -0.8753 | 0.0198 |
| 326 | Rab11a | -0.8752 | 0.0002 |
| 327 | Sel1l3 | -0.8752 | 0.0210 |
| 328 | Hpgds | -0.8749 | 0.0169 |
| 329 | Cx3cr1 | -0.8749 | 0.0000 |
| 330 | Actn2 | -0.8746 | 0.0037 |
| 331 | Gypa | -0.8738 | 0.0089 |
| 332 | Fech | -0.8725 | 0.0067 |
| 333 | Pla2g12a | -0.8723 | 0.0021 |
| 334 | Rsad2 | -0.8722 | 0.0078 |
| 335 | Hsf2bp | -0.8721 | 0.0210 |
| 336 | Klhl14 | -0.8719 | 0.0245 |
| 337 | H2-K1 | -0.8699 | 0.0003 |
| 338 | Fcrla | -0.8697 | 0.0093 |
| 339 | Ghitm | -0.8692 | 0.0008 |
| 340 | Bnip3l | -0.8681 | 0.0024 |
| 341 | Fert2 | -0.8676 | 0.0049 |
| 342 | Pgpep1 | -0.8666 | 0.0083 |
| 343 | Cpe | -0.8662 | 0.0123 |
| 344 | Rgn | -0.8650 | 0.0256 |
| 345 | Fam167a | -0.8640 | 0.0156 |
| 346 | Fam220a | -0.8636 | 0.0083 |
| 347 | Acmsd | -0.8634 | 0.0137 |
| 348 | Klra5 | -0.8630 | 0.0011 |
| 349 | Ptpn7 | -0.8605 | 0.0000 |
| 350 | Ramp1 | -0.8605 | 0.0001 |
| 351 | Clec3b | -0.8601 | 0.0005 |
| 352 | Paqr9 | -0.8599 | 0.0060 |
| 353 | Trim36 | -0.8597 | 0.0002 |
| 354 | Clic4 | -0.8576 | 0.0002 |
| 355 | Ipo11 | -0.8572 | 0.0001 |
| 356 | Hagh | -0.8572 | 0.0055 |
| 357 | Mfsd12 | -0.8565 | 0.0009 |
| 358 | Nrxn2 | -0.8564 | 0.0252 |
| 359 | Ppp1cb | -0.8559 | 0.0040 |
| 360 | Creg1 | -0.8552 | 0.0012 |
| 361 | Tnni1 | -0.8550 | 0.0285 |
| 362 | Ifi30 | -0.8532 | 0.0024 |
| 363 | Rassf8 | -0.8530 | 0.0234 |
| 364 | 1700119H24Rik | -0.8528 | 0.0281 |
| 365 | Isca1 | -0.8525 | 0.0015 |
| 366 | Syt9 | -0.8518 | 0.0292 |
| 367 | Vegfa | -0.8512 | 0.0049 |
| 368 | 2-Mar | -0.8509 | 0.0022 |
| 369 | 2410004P03Rik | -0.8477 | 0.0298 |
| 370 | 1700037H04Rik | -0.8473 | 0.0001 |
| 371 | Pisd-ps2 | -0.8455 | 0.0049 |
| 372 | Eif4ebp1 | -0.8454 | 0.0001 |
| 373 | Snrnp25 | -0.8438 | 0.0016 |
| 374 | Tmprss6 | -0.8438 | 0.0307 |
| 375 | Triqk | -0.8434 | 0.0020 |
| 376 | Itga8 | -0.8432 | 0.0306 |
| 377 | Krt18 | -0.8430 | 0.0309 |
| 378 | Plekho1 | -0.8430 | 0.0000 |
| 379 | Ptp4a3 | -0.8424 | 0.0058 |
| 380 | Sfxn1 | -0.8409 | 0.0000 |
| 381 | Glp1r | -0.8395 | 0.0239 |
| 382 | Tmem123 | -0.8393 | 0.0000 |
| 383 | Cd24a | -0.8389 | 0.0077 |
| 384 | Epb4.1 | -0.8387 | 0.0062 |
| 385 | Ramp3 | -0.8385 | 0.0001 |
| 386 | Pim3 | -0.8385 | 0.0000 |
| 387 | Uhrf1bp1 | -0.8359 | 0.0020 |
| 388 | Ldhc | -0.8359 | 0.0323 |
| 389 | Loxl1 | -0.8358 | 0.0323 |
| 390 | F830002L21Rik | -0.8346 | 0.0096 |
| 391 | 4932443I19Rik | -0.8343 | 0.0056 |
| 392 | Snn | -0.8342 | 0.0003 |
| 393 | Aldh1a1 | -0.8334 | 0.0025 |
| 394 | Kif5a | -0.8330 | 0.0220 |
| 395 | Gnat1 | -0.8326 | 0.0191 |
| 396 | Txnrd2 | -0.8325 | 0.0077 |
| 397 | Aldh3b2 | -0.8324 | 0.0325 |
| 398 | Evpl | -0.8318 | 0.0325 |
| 399 | Slc25a39 | -0.8316 | 0.0113 |
| 400 | Aldh7a1 | -0.8314 | 0.0162 |
| 401 | Cecr2 | -0.8312 | 0.0005 |
| 402 | 9830132P13Rik | -0.8303 | 0.0234 |
| 403 | Creb3l1 | -0.8295 | 0.0331 |
| 404 | Lipc | -0.8294 | 0.0023 |
| 405 | Tenm4 | -0.8293 | 0.0257 |
| 406 | Josd1 | -0.8291 | 0.0000 |
| 407 | Ptpn18 | -0.8289 | 0.0003 |
| 408 | Scpep1os | -0.8284 | 0.0335 |
| 409 | E230016K23Rik | -0.8284 | 0.0242 |
| 410 | Ormdl3 | -0.8262 | 0.0051 |
| 411 | H2-Ab1 | -0.8260 | 0.0009 |
| 412 | Jag2 | -0.8254 | 0.0190 |
| 413 | Zfp318 | -0.8247 | 0.0001 |
| 414 | 4930417O13Rik | -0.8247 | 0.0178 |
| 415 | Snord100 | -0.8247 | 0.0088 |
| 416 | Fam83f | -0.8245 | 0.0309 |
| 417 | Mgl2 | -0.8241 | 0.0320 |
| 418 | E2f2 | -0.8233 | 0.0036 |
| 419 | Acot13 | -0.8231 | 0.0060 |
| 420 | Ttc39a | -0.8220 | 0.0213 |
| 421 | Slc27a6 | -0.8220 | 0.0328 |
| 422 | Prr5 | -0.8213 | 0.0011 |
| 423 | Adam23 | -0.8208 | 0.0234 |
| 424 | Whrn | -0.8208 | 0.0008 |
| 425 | Cdk20 | -0.8198 | 0.0124 |
| 426 | Klf16 | -0.8192 | 0.0000 |
| 427 | Ucp2 | -0.8191 | 0.0110 |
| 428 | Slc44a1 | -0.8190 | 0.0005 |
| 429 | Ckb | -0.8184 | 0.0028 |
| 430 | Mir6913 | -0.8177 | 0.0206 |
| 431 | Gm4489 | -0.8175 | 0.0214 |
| 432 | Kctd14 | -0.8171 | 0.0290 |
| 433 | Cd9 | -0.8167 | 0.0036 |
| 434 | Ost4 | -0.8158 | 0.0015 |
| 435 | Bcar3 | -0.8156 | 0.0057 |
| 436 | Nppa | -0.8154 | 0.0360 |
| 437 | B330016D10Rik | -0.8145 | 0.0073 |
| 438 | C430049B03Rik | -0.8144 | 0.0107 |
| 439 | 4930426D05Rik | -0.8143 | 0.0104 |
| 440 | Ppef2 | -0.8137 | 0.0362 |
| 441 | Prdx2 | -0.8137 | 0.0070 |
| 442 | Zfp704 | -0.8136 | 0.0012 |
| 443 | Nynrin | -0.8135 | 0.0107 |
| 444 | Pax5 | -0.8134 | 0.0075 |
| 445 | B3gnt8 | -0.8132 | 0.0115 |
| 446 | Igip | -0.8129 | 0.0062 |
| 447 | Asb1 | -0.8126 | 0.0017 |
| 448 | Pptc7 | -0.8126 | 0.0000 |
| 449 | Tcf4 | -0.8123 | 0.0000 |
| 450 | Cd19 | -0.8111 | 0.0082 |
| 451 | Ptpro | -0.8108 | 0.0005 |
| 452 | Tifab | -0.8105 | 0.0070 |
| 453 | Spa17 | -0.8091 | 0.0181 |
| 454 | Dnah2 | -0.8082 | 0.0034 |
| 455 | Lima1 | -0.8081 | 0.0005 |
| 456 | Pnck | -0.8077 | 0.0371 |
| 457 | Pcsk9 | -0.8074 | 0.0381 |
| 458 | Zdhhc14 | -0.8072 | 0.0067 |
| 459 | 2410076I21Rik | -0.8070 | 0.0052 |
| 460 | Hbq1a | -0.8067 | 0.0286 |
| 461 | Dbn1 | -0.8064 | 0.0012 |
| 462 | Snca | -0.8051 | 0.0064 |
| 463 | Map1a | -0.8041 | 0.0021 |
| 464 | Snap23 | -0.8040 | 0.0005 |
| 465 | 1700029N11Rik | -0.8035 | 0.0036 |
| 466 | Pla2g16 | -0.8032 | 0.0029 |
| 467 | Cdr2 | -0.8027 | 0.0121 |
| 468 | Ccnd1 | -0.8026 | 0.0049 |
| 469 | Lsm12 | -0.8025 | 0.0015 |
| 470 | Shank3 | -0.8022 | 0.0371 |
| 471 | Grk5 | -0.8019 | 0.0003 |
| 472 | Aldh1b1 | -0.8019 | 0.0002 |
| 473 | Aqp1 | -0.8017 | 0.0028 |
| 474 | Efcc1 | -0.8015 | 0.0109 |
| 475 | Sytl4 | -0.8006 | 0.0043 |
| 476 | Gem | -0.7991 | 0.0000 |
| 477 | D030028A08Rik | -0.7989 | 0.0041 |
| 478 | Tmem163 | -0.7988 | 0.0138 |
| 479 | Tsc22d1 | -0.7966 | 0.0018 |
| 480 | Apitd1 | -0.7966 | 0.0291 |
| 481 | Pvrl1 | -0.7953 | 0.0004 |
| 482 | Mpp2 | -0.7942 | 0.0215 |
| 483 | Cnst | -0.7941 | 0.0002 |
| 484 | S100a1 | -0.7939 | 0.0024 |
| 485 | Nfic | -0.7928 | 0.0014 |
| 486 | Cd209e | -0.7905 | 0.0150 |
| 487 | Ctla2b | -0.7904 | 0.0010 |
| 488 | Sh3bgrl2 | -0.7900 | 0.0015 |
| 489 | Angptl4 | -0.7899 | 0.0234 |
| 490 | Galnt9 | -0.7894 | 0.0024 |
| 491 | Tigd3 | -0.7888 | 0.0146 |
| 492 | Utp14b | -0.7881 | 0.0000 |
| 493 | Pcp4l1 | -0.7874 | 0.0038 |
| 494 | Rab22a | -0.7871 | 0.0012 |
| 495 | Rgs10 | -0.7869 | 0.0003 |
| 496 | Alas2 | -0.7864 | 0.0161 |
| 497 | Bst2 | -0.7860 | 0.0000 |
| 498 | Sash1 | -0.7860 | 0.0145 |
| 499 | Apoc1 | -0.7859 | 0.0427 |
| 500 | Coa5 | -0.7859 | 0.0029 |
| 501 | Isg20 | -0.7858 | 0.0104 |
| 502 | Esd | -0.7858 | 0.0010 |
| 503 | Fbxl13 | -0.7850 | 0.0108 |
| 504 | Ppp1r10 | -0.7842 | 0.0029 |
| 505 | Ptpn14 | -0.7840 | 0.0019 |
| 506 | Ube2c | -0.7840 | 0.0084 |
| 507 | Hist1h4c | -0.7839 | 0.0279 |
| 508 | Dusp10 | -0.7829 | 0.0003 |
| 509 | Armcx4 | -0.7828 | 0.0113 |
| 510 | Rgs16 | -0.7827 | 0.0027 |
| 511 | Snx15 | -0.7819 | 0.0005 |
| 512 | Tmsb4x | -0.7818 | 0.0015 |
| 513 | Acp1 | -0.7812 | 0.0078 |
| 514 | Gtse1 | -0.7805 | 0.0265 |
| 515 | Zbtb10 | -0.7804 | 0.0005 |
| 516 | Slc38a5 | -0.7793 | 0.0302 |
| 517 | Plcb1 | -0.7788 | 0.0005 |
| 518 | Ephb2 | -0.7781 | 0.0087 |
| 519 | Phldb3 | -0.7777 | 0.0005 |
| 520 | Ctla2a | -0.7776 | 0.0033 |
| 521 | Slc30a10 | -0.7776 | 0.0378 |
| 522 | Bola3 | -0.7775 | 0.0109 |
| 523 | Ctxn1 | -0.7774 | 0.0053 |
| 524 | Naip1 | -0.7765 | 0.0460 |
| 525 | Gm6634 | -0.7761 | 0.0057 |
| 526 | Ubap1 | -0.7758 | 0.0007 |
| 527 | Slamf9 | -0.7757 | 0.0339 |
| 528 | Mylk | -0.7756 | 0.0020 |
| 529 | Rcor3 | -0.7755 | 0.0000 |
| 530 | Aamdc | -0.7753 | 0.0003 |
| 531 | Tmem150b | -0.7752 | 0.0351 |
| 532 | Clip2 | -0.7749 | 0.0000 |
| 533 | Ndufa4 | -0.7738 | 0.0006 |
| 534 | Armc2 | -0.7737 | 0.0279 |
| 535 | Maff | -0.7737 | 0.0000 |
| 536 | Oit3 | -0.7732 | 0.0043 |
| 537 | Cd79a | -0.7731 | 0.0133 |
| 538 | Scamp1 | -0.7730 | 0.0012 |
| 539 | Tmem14a | -0.7715 | 0.0138 |
| 540 | Pdp2 | -0.7691 | 0.0020 |
| 541 | Slc7a5 | -0.7691 | 0.0024 |
| 542 | 1700048O20Rik | -0.7687 | 0.0368 |
| 543 | Fth1 | -0.7676 | 0.0018 |
| 544 | Mx2 | -0.7662 | 0.0007 |
| 545 | Zeb2os | -0.7660 | 0.0033 |
| 546 | Psme3 | -0.7656 | 0.0078 |
| 547 | Ubac1 | -0.7656 | 0.0057 |
| 548 | Jmy | -0.7656 | 0.0000 |
| 549 | Lamp3 | -0.7642 | 0.0354 |
| 550 | Zcchc24 | -0.7639 | 0.0022 |
| 551 | Gpr21 | -0.7638 | 0.0487 |
| 552 | Acot12 | -0.7635 | 0.0414 |
| 553 | Ak8 | -0.7633 | 0.0467 |
| 554 | Rad54b | -0.7615 | 0.0239 |
| 555 | Lrp4 | -0.7607 | 0.0413 |
| 556 | Spta1 | -0.7603 | 0.0160 |
| 557 | Gng11 | -0.7599 | 0.0040 |
| 558 | Gtf2ird1 | -0.7595 | 0.0209 |
| 559 | Fam45a | -0.7592 | 0.0070 |
| 560 | Lcat | -0.7591 | 0.0283 |
| 561 | Rilp | -0.7590 | 0.0265 |
| 562 | Wnk4 | -0.7586 | 0.0131 |
| 563 | Vangl1 | -0.7583 | 0.0234 |
| 564 | Efnb2 | -0.7577 | 0.0470 |
| 565 | Acp5 | -0.7573 | 0.0001 |
| 566 | Ifit2 | -0.7558 | 0.0027 |
| 567 | Trerf1 | -0.7556 | 0.0013 |
| 568 | Cpq | -0.7555 | 0.0015 |
| 569 | H2-DMa | -0.7555 | 0.0006 |
| 570 | Sbf2 | -0.7553 | 0.0008 |
| 571 | Rgs9 | -0.7537 | 0.0157 |
| 572 | Slc48a1 | -0.7527 | 0.0217 |
| 573 | Rrad | -0.7526 | 0.0000 |
| 574 | Rhoj | -0.7522 | 0.0021 |
| 575 | Fhl1 | -0.7510 | 0.0035 |
| 576 | Map3k14 | -0.7508 | 0.0001 |
| 577 | Ccdc136 | -0.7504 | 0.0481 |
| 578 | Cd81 | -0.7499 | 0.0063 |
| 579 | Mslnl | -0.7495 | 0.0491 |
| 580 | Afp | -0.7494 | 0.0493 |
| 581 | Ccndbp1 | -0.7487 | 0.0049 |
| 582 | Nuak2 | -0.7481 | 0.0000 |
| 583 | Esam | -0.7469 | 0.0004 |
| 584 | B4galt1 | -0.7465 | 0.0000 |
| 585 | Ntan1 | -0.7455 | 0.0002 |
| 586 | Csgalnact1 | -0.7451 | 0.0030 |
| 587 | Ube2r2 | -0.7451 | 0.0055 |
| 588 | Ftl1 | -0.7444 | 0.0039 |
| 589 | Hrsp12 | -0.7442 | 0.0029 |
| 590 | Them7 | -0.7438 | 0.0467 |
| 591 | Srgap3 | -0.7433 | 0.0000 |
| 592 | Btbd3 | -0.7431 | 0.0210 |
| 593 | Chst7 | -0.7424 | 0.0384 |
| 594 | Ptpla | -0.7417 | 0.0053 |
| 595 | 1110046J04Rik | -0.7416 | 0.0387 |
| 596 | Irs2 | -0.7415 | 0.0008 |
| 597 | Cecr6 | -0.7411 | 0.0325 |
| 598 | Tnfrsf12a | -0.7410 | 0.0002 |
| 599 | Hist3h2a | -0.7410 | 0.0004 |
| 600 | Nmral1 | -0.7399 | 0.0088 |
| 601 | Nmnat2 | -0.7384 | 0.0398 |
| 602 | Car3 | -0.7383 | 0.0458 |
| 603 | Mxi1 | -0.7380 | 0.0022 |
| 604 | Cd300lg | -0.7378 | 0.0301 |
| 605 | Ppil3 | -0.7377 | 0.0011 |
| 606 | Trpc6 | -0.7372 | 0.0236 |
| 607 | Hscb | -0.7361 | 0.0095 |
| 608 | Cstad | -0.7359 | 0.0403 |
| 609 | Ybx3 | -0.7353 | 0.0110 |
| 610 | H2-Ob | -0.7352 | 0.0073 |
| 611 | 4930511A02Rik | -0.7349 | 0.0208 |
| 612 | Vmn2r26 | -0.7349 | 0.0208 |
| 613 | Slc27a2 | -0.7349 | 0.0492 |
| 614 | I730030J21Rik | -0.7347 | 0.0065 |
| 615 | Rit1 | -0.7346 | 0.0021 |
| 616 | Atp6v1f | -0.7345 | 0.0011 |
| 617 | Rgs2 | -0.7343 | 0.0000 |
| 618 | Pea15a | -0.7333 | 0.0015 |
| 619 | Cetn2 | -0.7327 | 0.0032 |
| 620 | Ngfr | -0.7324 | 0.0419 |
| 621 | Pla2g4a | -0.7321 | 0.0039 |
| 622 | Wap | -0.7319 | 0.0202 |
| 623 | Bcas3os1 | -0.7317 | 0.0331 |
| 624 | Crem | -0.7317 | 0.0000 |
| 625 | Olfr199 | -0.7310 | 0.0212 |
| 626 | Tuba3b | -0.7310 | 0.0212 |
| 627 | Got1 | -0.7303 | 0.0000 |
| 628 | Phtf2 | -0.7294 | 0.0067 |
| 629 | Xcr1 | -0.7293 | 0.0136 |
| 630 | Sh3d19 | -0.7291 | 0.0151 |
| 631 | Mctp1 | -0.7286 | 0.0038 |
| 632 | 4833439L19Rik | -0.7275 | 0.0003 |
| 633 | Hsbp1 | -0.7272 | 0.0010 |
| 634 | Taldo1 | -0.7270 | 0.0010 |
| 635 | P2rx1 | -0.7268 | 0.0032 |
| 636 | Rgcc | -0.7256 | 0.0000 |
| 637 | Redrum | -0.7251 | 0.0294 |
| 638 | Nadk2 | -0.7241 | 0.0036 |
| 639 | Cd226 | -0.7237 | 0.0044 |
| 640 | Trappc2 | -0.7236 | 0.0115 |
| 641 | Stac2 | -0.7234 | 0.0396 |
| 642 | Tspo2 | -0.7233 | 0.0288 |
| 643 | Mustn1 | -0.7231 | 0.0373 |
| 644 | Wbscr27 | -0.7211 | 0.0131 |
| 645 | Triap1 | -0.7205 | 0.0093 |
| 646 | Emr1 | -0.7196 | 0.0000 |
| 647 | Cyb5 | -0.7195 | 0.0023 |
| 648 | Chtf18 | -0.7187 | 0.0114 |
| 649 | Bin1 | -0.7185 | 0.0007 |
| 650 | Ascc1 | -0.7181 | 0.0078 |
| 651 | Mex3b | -0.7179 | 0.0002 |
| 652 | Slc4a11 | -0.7174 | 0.0470 |
| 653 | Tgfb1i1 | -0.7171 | 0.0038 |
| 654 | Foxo4 | -0.7167 | 0.0097 |
| 655 | Notum | -0.7153 | 0.0344 |
| 656 | Zbtb1 | -0.7152 | 0.0000 |
| 657 | Blk | -0.7146 | 0.0170 |
| 658 | Paip2 | -0.7142 | 0.0016 |
| 659 | Casz1 | -0.7141 | 0.0005 |
| 660 | Traf4 | -0.7138 | 0.0001 |
| 661 | Acsl3 | -0.7136 | 0.0048 |
| 662 | Rhbdf1 | -0.7124 | 0.0273 |
| 663 | Ift22 | -0.7120 | 0.0292 |
| 664 | Serf2 | -0.7118 | 0.0002 |
| 665 | Pf4 | -0.7117 | 0.0108 |
| 666 | Trim10 | -0.7109 | 0.0265 |
| 667 | Ell3 | -0.7092 | 0.0218 |
| 668 | Slc46a3 | -0.7090 | 0.0302 |
| 669 | Lim2 | -0.7075 | 0.0279 |
| 670 | Klrc2 | -0.7073 | 0.0424 |
| 671 | Tdrp | -0.7054 | 0.0000 |
| 672 | Ptprs | -0.7053 | 0.0012 |
| 673 | Tmem129 | -0.7049 | 0.0020 |
| 674 | Pdgfa | -0.7044 | 0.0141 |
| 675 | Eif2ak3 | -0.7040 | 0.0011 |
| 676 | Rnf11 | -0.7031 | 0.0051 |
| 677 | Ecm1 | -0.7029 | 0.0018 |
| 678 | Trim9 | -0.7020 | 0.0258 |
| 679 | Dmrta1 | -0.7014 | 0.0186 |
| 680 | Spc24 | -0.7013 | 0.0314 |
| 681 | Fam96b | -0.7012 | 0.0009 |
| 682 | Plxnd1 | -0.6992 | 0.0257 |
| 683 | Cyp4b1-ps2 | -0.6988 | 0.0319 |
| 684 | Cnn3 | -0.6982 | 0.0095 |
| 685 | Tubb2b | -0.6981 | 0.0461 |
| 686 | Nsg2 | -0.6977 | 0.0008 |
| 687 | Cd40 | -0.6959 | 0.0236 |
| 688 | Sh3bgrl3 | -0.6951 | 0.0082 |
| 689 | Ttll11 | -0.6944 | 0.0042 |
| 690 | Mfsd7a | -0.6938 | 0.0293 |
| 691 | Cd99l2 | -0.6937 | 0.0062 |
| 692 | Prkab2 | -0.6935 | 0.0079 |
| 693 | Srxn1 | -0.6935 | 0.0071 |
| 694 | Snx8 | -0.6933 | 0.0229 |
| 695 | Ubb | -0.6932 | 0.0007 |
| 696 | Polr3k | -0.6929 | 0.0012 |
| 697 | Isg15 | -0.6911 | 0.0416 |
| 698 | Itsn1 | -0.6908 | 0.0039 |
| 699 | Arhgef40 | -0.6907 | 0.0257 |
| 700 | Atpaf2 | -0.6907 | 0.0062 |
| 701 | Myl6 | -0.6903 | 0.0010 |
| 702 | P2ry14 | -0.6898 | 0.0146 |
| 703 | Cdc6 | -0.6886 | 0.0474 |
| 704 | Trpc1 | -0.6883 | 0.0213 |
| 705 | Ccdc150 | -0.6881 | 0.0331 |
| 706 | Coa3 | -0.6873 | 0.0097 |
| 707 | Maml3 | -0.6873 | 0.0000 |
| 708 | Snhg10 | -0.6872 | 0.0481 |
| 709 | Gm5431 | -0.6872 | 0.0404 |
| 710 | Cacna1e | -0.6860 | 0.0225 |
| 711 | Kcnj5 | -0.6860 | 0.0021 |
| 712 | Klri2 | -0.6856 | 0.0004 |
| 713 | 1600020E01Rik | -0.6855 | 0.0054 |
| 714 | Hpse | -0.6852 | 0.0037 |
| 715 | Fam174b | -0.6844 | 0.0021 |
| 716 | Dyx1c1 | -0.6841 | 0.0441 |
| 717 | Armcx6 | -0.6840 | 0.0072 |
| 718 | Tmcc3 | -0.6839 | 0.0001 |
| 719 | Dnajb2 | -0.6829 | 0.0198 |
| 720 | Pdlim1 | -0.6823 | 0.0034 |
| 721 | Tmem11 | -0.6814 | 0.0000 |
| 722 | Lmo2 | -0.6811 | 0.0027 |
| 723 | Ap2m1 | -0.6807 | 0.0074 |
| 724 | Pink1 | -0.6804 | 0.0012 |
| 725 | Hmga2-ps1 | -0.6804 | 0.0072 |
| 726 | Ankrd35 | -0.6803 | 0.0344 |
| 727 | Ppp1r16b | -0.6801 | 0.0019 |
| 728 | Myct1 | -0.6799 | 0.0036 |
| 729 | P2ry12 | -0.6799 | 0.0012 |
| 730 | Cox7a2 | -0.6797 | 0.0009 |
| 731 | Yaf2 | -0.6796 | 0.0003 |
| 732 | Srp14 | -0.6787 | 0.0054 |
| 733 | Ccr9 | -0.6786 | 0.0006 |
| 734 | Pygm | -0.6780 | 0.0001 |
| 735 | Mrap2 | -0.6779 | 0.0451 |
| 736 | Gpr68 | -0.6769 | 0.0009 |
| 737 | Atf4 | -0.6765 | 0.0000 |
| 738 | Spata2l | -0.6742 | 0.0041 |
| 739 | Sdc4 | -0.6740 | 0.0295 |
| 740 | Cnr2 | -0.6733 | 0.0003 |
| 741 | Zfp367 | -0.6723 | 0.0058 |
| 742 | Dnajc17 | -0.6719 | 0.0056 |
| 743 | Etv5 | -0.6715 | 0.0088 |
| 744 | 8-Mar | -0.6708 | 0.0060 |
| 745 | Cpa2 | -0.6705 | 0.0484 |
| 746 | Tmem246 | -0.6695 | 0.0345 |
| 747 | Zfyve21 | -0.6687 | 0.0173 |
| 748 | Adcy5 | -0.6686 | 0.0131 |
| 749 | Nptn | -0.6685 | 0.0087 |
| 750 | Pid1 | -0.6684 | 0.0004 |
| 751 | Ndrg1 | -0.6675 | 0.0059 |
| 752 | Med9 | -0.6674 | 0.0114 |
| 753 | Gm15706 | -0.6670 | 0.0170 |
| 754 | Sesn1 | -0.6670 | 0.0012 |
| 755 | Fgd2 | -0.6660 | 0.0125 |
| 756 | Setbp1 | -0.6660 | 0.0055 |
| 757 | Kifc1 | -0.6650 | 0.0081 |
| 758 | Ccl24 | -0.6650 | 0.0375 |
| 759 | Myom1 | -0.6648 | 0.0422 |
| 760 | Irf7 | -0.6646 | 0.0017 |
| 761 | Adck3 | -0.6645 | 0.0049 |
| 762 | Fis1 | -0.6644 | 0.0167 |
| 763 | Rfesd | -0.6640 | 0.0034 |
| 764 | Klhl25 | -0.6640 | 0.0000 |
| 765 | Zcchc18 | -0.6632 | 0.0195 |
| 766 | Ccdc88a | -0.6628 | 0.0000 |
| 767 | Memo1 | -0.6619 | 0.0316 |
| 768 | Slco4a1 | -0.6616 | 0.0226 |
| 769 | Fam57b | -0.6609 | 0.0117 |
| 770 | Ppp4r1l-ps | -0.6607 | 0.0007 |
| 771 | Dusp19 | -0.6607 | 0.0037 |
| 772 | Pcmt1 | -0.6604 | 0.0080 |
| 773 | Coro2a | -0.6603 | 0.0000 |
| 774 | Loxl3 | -0.6601 | 0.0059 |
| 775 | Cacna1d | -0.6598 | 0.0202 |
| 776 | Vgll4 | -0.6594 | 0.0004 |
| 777 | Proser2 | -0.6593 | 0.0011 |
| 778 | Bcl2l1 | -0.6591 | 0.0129 |
| 779 | Atrn | -0.6588 | 0.0093 |
| 780 | Tmem256 | -0.6586 | 0.0403 |
| 781 | Hmgb1 | -0.6575 | 0.0095 |
| 782 | Haao | -0.6563 | 0.0209 |
| 783 | Vdac1 | -0.6563 | 0.0011 |
| 784 | Nmrk2 | -0.6552 | 0.0321 |
| 785 | Rcan2 | -0.6552 | 0.0321 |
| 786 | Ypel5 | -0.6549 | 0.0077 |
| 787 | Oaz2 | -0.6549 | 0.0094 |
| 788 | Cysltr2 | -0.6548 | 0.0092 |
| 789 | Plekha2 | -0.6547 | 0.0002 |
| 790 | Mycbp | -0.6545 | 0.0139 |
| 791 | Bambi | -0.6545 | 0.0139 |
| 792 | 9430020K01Rik | -0.6544 | 0.0328 |
| 793 | Kcna4 | -0.6543 | 0.0215 |
| 794 | Ninj1 | -0.6538 | 0.0010 |
| 795 | Ppp2r2c | -0.6536 | 0.0322 |
| 796 | Cdh23 | -0.6534 | 0.0351 |
| 797 | Armcx3 | -0.6528 | 0.0010 |
| 798 | Dusp23 | -0.6513 | 0.0357 |
| 799 | Tns1 | -0.6503 | 0.0037 |
| 800 | Msh6 | -0.6498 | 0.0001 |
| 801 | Med12l | -0.6484 | 0.0005 |
| 802 | Otud7b | -0.6482 | 0.0037 |
| 803 | Zfp219 | -0.6470 | 0.0002 |
| 804 | Mir24-2 | -0.6468 | 0.0256 |
| 805 | Gas5 | -0.6467 | 0.0008 |
| 806 | Cttn | -0.6466 | 0.0017 |
| 807 | Tgif1 | -0.6465 | 0.0017 |
| 808 | Sh3glb1 | -0.6465 | 0.0294 |
| 809 | Lrrc24 | -0.6460 | 0.0480 |
| 810 | Nkrf | -0.6460 | 0.0020 |
| 811 | Erf | -0.6455 | 0.0000 |
| 812 | Nrp2 | -0.6448 | 0.0301 |
| 813 | Tmem40 | -0.6447 | 0.0010 |
| 814 | Vav2 | -0.6442 | 0.0063 |
| 815 | Sc5d | -0.6441 | 0.0001 |
| 816 | Eng | -0.6437 | 0.0230 |
| 817 | Med21 | -0.6431 | 0.0109 |
| 818 | Mpc2 | -0.6426 | 0.0126 |
| 819 | Mrvi1 | -0.6424 | 0.0155 |
| 820 | Ccdc92 | -0.6421 | 0.0238 |
| 821 | Nusap1 | -0.6421 | 0.0249 |
| 822 | Rtn4ip1 | -0.6419 | 0.0242 |
| 823 | Gm5069 | -0.6418 | 0.0015 |
| 824 | Snhg7 | -0.6406 | 0.0136 |
| 825 | Ppcs | -0.6400 | 0.0117 |
| 826 | Il2ra | -0.6390 | 0.0215 |
| 827 | Cpped1 | -0.6389 | 0.0030 |
| 828 | Arhgap10 | -0.6381 | 0.0128 |
| 829 | Ldlrap1 | -0.6376 | 0.0001 |
| 830 | Gmpr | -0.6375 | 0.0147 |
| 831 | Tceb2 | -0.6370 | 0.0046 |
| 832 | Cxxc5 | -0.6370 | 0.0149 |
| 833 | Dennd5b | -0.6367 | 0.0101 |
| 834 | Max | -0.6367 | 0.0067 |
| 835 | Cr2 | -0.6366 | 0.0271 |
| 836 | Htatip2 | -0.6366 | 0.0031 |
| 837 | Pdzd4 | -0.6365 | 0.0102 |
| 838 | Nop16 | -0.6359 | 0.0012 |
| 839 | Prokr1 | -0.6357 | 0.0128 |
| 840 | Mef2d | -0.6351 | 0.0000 |
| 841 | Itgb4 | -0.6347 | 0.0481 |
| 842 | Usmg5 | -0.6346 | 0.0022 |
| 843 | Map3k19 | -0.6344 | 0.0430 |
| 844 | Pprc1 | -0.6341 | 0.0000 |
| 845 | Mzb1 | -0.6340 | 0.0001 |
| 846 | Lyl1 | -0.6339 | 0.0000 |
| 847 | Zfand3 | -0.6336 | 0.0130 |
| 848 | Gpx4 | -0.6335 | 0.0030 |
| 849 | Dip2c | -0.6329 | 0.0040 |
| 850 | Vopp1 | -0.6328 | 0.0274 |
| 851 | Dnase2a | -0.6325 | 0.0018 |
| 852 | Ubald1 | -0.6323 | 0.0000 |
| 853 | Aldoc | -0.6319 | 0.0383 |
| 854 | Cpm | -0.6317 | 0.0370 |
| 855 | Fam131a | -0.6315 | 0.0321 |
| 856 | Ccdc32 | -0.6301 | 0.0054 |
| 857 | Pacsin2 | -0.6290 | 0.0035 |
| 858 | P2ry6 | -0.6289 | 0.0244 |
| 859 | Ica1l | -0.6288 | 0.0046 |
| 860 | Lyve1 | -0.6287 | 0.0184 |
| 861 | Rassf3 | -0.6278 | 0.0000 |
| 862 | Arpc1a | -0.6276 | 0.0228 |
| 863 | Ptplad2 | -0.6276 | 0.0196 |
| 864 | Nkiras2 | -0.6275 | 0.0002 |
| 865 | Ifit3 | -0.6269 | 0.0002 |
| 866 | Abcb4 | -0.6269 | 0.0366 |
| 867 | Urod | -0.6266 | 0.0191 |
| 868 | Jup | -0.6266 | 0.0095 |
| 869 | Pou2af1 | -0.6265 | 0.0154 |
| 870 | Eif2ak2 | -0.6263 | 0.0004 |
| 871 | Sbk1 | -0.6262 | 0.0002 |
| 872 | Litaf | -0.6246 | 0.0020 |
| 873 | Apba1 | -0.6244 | 0.0318 |
| 874 | Med22 | -0.6237 | 0.0002 |
| 875 | Spdl1 | -0.6228 | 0.0317 |
| 876 | Zfp296 | -0.6227 | 0.0212 |
| 877 | Slc4a1 | -0.6227 | 0.0454 |
| 878 | 2210013O21Rik | -0.6227 | 0.0180 |
| 879 | Brdt | -0.6226 | 0.0376 |
| 880 | Mtss1 | -0.6221 | 0.0000 |
| 881 | Tpi1 | -0.6218 | 0.0064 |
| 882 | N4bp3 | -0.6216 | 0.0252 |
| 883 | 3-Mar | -0.6216 | 0.0021 |
| 884 | Aldh2 | -0.6209 | 0.0047 |
| 885 | Gm11541 | -0.6207 | 0.0382 |
| 886 | Pard3b | -0.6204 | 0.0060 |
| 887 | F11r | -0.6198 | 0.0104 |
| 888 | Slc9a3 | -0.6192 | 0.0385 |
| 889 | 5430435G22Rik | -0.6190 | 0.0154 |
| 890 | Ltbp2 | -0.6190 | 0.0265 |
| 891 | Adarb1 | -0.6189 | 0.0300 |
| 892 | Pafah1b3 | -0.6177 | 0.0370 |
| 893 | Tuba8 | -0.6174 | 0.0090 |
| 894 | Becn1 | -0.6174 | 0.0093 |
| 895 | Rgag4 | -0.6170 | 0.0013 |
| 896 | Grb7 | -0.6169 | 0.0292 |
| 897 | Tmem258 | -0.6167 | 0.0004 |
| 898 | Gp6 | -0.6157 | 0.0117 |
| 899 | Kif2a | -0.6157 | 0.0117 |
| 900 | Pttg1ip | -0.6155 | 0.0141 |
| 901 | 1500012F01Rik | -0.6154 | 0.0000 |
| 902 | Ccdc166 | -0.6153 | 0.0410 |
| 903 | Klhl20 | -0.6153 | 0.0015 |
| 904 | Alkbh6 | -0.6143 | 0.0004 |
| 905 | Angpt1 | -0.6142 | 0.0296 |
| 906 | Nav1 | -0.6142 | 0.0000 |
| 907 | Etv3 | -0.6132 | 0.0000 |
| 908 | Phlda3 | -0.6129 | 0.0203 |
| 909 | Lrig1 | -0.6126 | 0.0000 |
| 910 | Odc1 | -0.6122 | 0.0157 |
| 911 | Setmar | -0.6121 | 0.0196 |
| 912 | Fdps | -0.6114 | 0.0124 |
| 913 | Blvrb | -0.6106 | 0.0384 |
| 914 | Chmp5 | -0.6097 | 0.0062 |
| 915 | S100a13 | -0.6094 | 0.0209 |
| 916 | Rbpms2 | -0.6094 | 0.0339 |
| 917 | Lzts2 | -0.6090 | 0.0297 |
| 918 | Ly86 | -0.6088 | 0.0000 |
| 919 | Sh3pxd2a | -0.6081 | 0.0025 |
| 920 | Ehd4 | -0.6080 | 0.0003 |
| 921 | Nlgn2 | -0.6067 | 0.0411 |
| 922 | Atg4c | -0.6061 | 0.0340 |
| 923 | P2rx4 | -0.6060 | 0.0001 |
| 924 | Tssc1 | -0.6059 | 0.0100 |
| 925 | Cd79b | -0.6053 | 0.0474 |
| 926 | Tspan14 | -0.6049 | 0.0033 |
| 927 | Chek2 | -0.6043 | 0.0039 |
| 928 | Gng5 | -0.6042 | 0.0137 |
| 929 | Fam210b | -0.6038 | 0.0472 |
| 930 | Stx7 | -0.6037 | 0.0096 |
| 931 | 4831440E17Rik | -0.6037 | 0.0396 |
| 932 | Eomes | -0.6028 | 0.0077 |
| 933 | Atp2a3 | -0.6025 | 0.0058 |
| 934 | Focad | -0.6019 | 0.0361 |
| 935 | Npy | -0.6017 | 0.0217 |
| 936 | Prmt2 | -0.6010 | 0.0137 |
| 937 | Pon3 | -0.6006 | 0.0210 |
| 938 | Stard10 | -0.6005 | 0.0003 |
| 939 | Dna2 | -0.6004 | 0.0080 |
| 940 | Bid | -0.5999 | 0.0113 |
| 941 | Sgce | -0.5996 | 0.0410 |
| 942 | Mb | -0.5991 | 0.0422 |
| 943 | Egln2 | -0.5988 | 0.0000 |
| 944 | Mafk | -0.5985 | 0.0002 |
| 945 | Fbxo9 | -0.5984 | 0.0166 |
| 946 | Cox7b | -0.5980 | 0.0053 |
| 947 | Pttg1 | -0.5975 | 0.0330 |
| 948 | R74862 | -0.5973 | 0.0368 |
| 949 | D830046C22Rik | -0.5967 | 0.0199 |
| 950 | Hfe | -0.5960 | 0.0068 |
| 951 | Gga2 | -0.5954 | 0.0026 |
| 952 | Papd7 | -0.5952 | 0.0102 |
| 953 | Sqle | -0.5949 | 0.0152 |
| 954 | Tagln2 | -0.5945 | 0.0103 |
| 955 | Ctsz | -0.5944 | 0.0000 |
| 956 | Slfn14 | -0.5941 | 0.0275 |
| 957 | Rnf144b | -0.5938 | 0.0043 |
| 958 | Mapk11 | -0.5929 | 0.0095 |
| 959 | I830077J02Rik | -0.5928 | 0.0088 |
| 960 | Zranb3 | -0.5924 | 0.0259 |
| 961 | Gp5 | -0.5924 | 0.0193 |
| 962 | Zfp672 | -0.5923 | 0.0003 |
| 963 | Cmas | -0.5921 | 0.0263 |
| 964 | Aplf | -0.5920 | 0.0230 |
| 965 | Tmem97 | -0.5911 | 0.0288 |
| 966 | Fbxl2 | -0.5902 | 0.0253 |
| 967 | 4930404N11Rik | -0.5901 | 0.0251 |
| 968 | Lifr | -0.5897 | 0.0000 |
| 969 | Trappc5 | -0.5891 | 0.0000 |
| 970 | Nxpe2 | -0.5891 | 0.0390 |
| 971 | Tnfrsf10b | -0.5890 | 0.0000 |
| 972 | LOC102632430 | -0.5887 | 0.0448 |
| 973 | Pecam1 | -0.5885 | 0.0010 |
| 974 | Arhgap22 | -0.5875 | 0.0259 |
| 975 | Gm15441 | -0.5872 | 0.0486 |
| 976 | Bicd2 | -0.5871 | 0.0043 |
| 977 | Arrb1 | -0.5861 | 0.0032 |
| 978 | Mtss1l | -0.5847 | 0.0331 |
| 979 | Amz2 | -0.5847 | 0.0014 |
| 980 | Skap2 | -0.5837 | 0.0066 |
| 981 | Clcn3 | -0.5834 | 0.0109 |
| 982 | Gsn | -0.5832 | 0.0073 |
| 983 | Cd151 | -0.5832 | 0.0186 |
| 984 | Dynlt3 | -0.5822 | 0.0156 |
| 985 | Homer3 | -0.5819 | 0.0399 |
| 986 | Micu1 | -0.5814 | 0.0171 |
| 987 | Ddah2 | -0.5811 | 0.0145 |
| 988 | H2-T24 | -0.5811 | 0.0031 |
| 989 | Ryr1 | -0.5802 | 0.0298 |
| 990 | Fam214b | -0.5796 | 0.0149 |
| 991 | Myl9 | -0.5792 | 0.0150 |
| 992 | Bysl | -0.5786 | 0.0044 |
| 993 | 2810013P06Rik | -0.5782 | 0.0086 |
| 994 | Ppp2r4 | -0.5782 | 0.0116 |
| 995 | Med29 | -0.5779 | 0.0031 |
| 996 | Six4 | -0.5777 | 0.0475 |
| 997 | Sh2b1 | -0.5775 | 0.0001 |
| 998 | Insig2 | -0.5775 | 0.0186 |
| 999 | Mapre2 | -0.5774 | 0.0000 |
| 1000 | Zfp512 | -0.5768 | 0.0000 |
| 1001 | Csn2 | -0.5768 | 0.0474 |
| 1002 | Uba52 | -0.5766 | 0.0012 |
| 1003 | Vdac3 | -0.5766 | 0.0244 |
| 1004 | AI662270 | -0.5765 | 0.0174 |
| 1005 | A930005H10Rik | -0.5757 | 0.0138 |
| 1006 | Mecr | -0.5756 | 0.0111 |
| 1007 | H2afj | -0.5752 | 0.0038 |
| 1008 | Trim25 | -0.5751 | 0.0149 |
| 1009 | Itga2b | -0.5749 | 0.0287 |
| 1010 | Calm3 | -0.5745 | 0.0092 |
| 1011 | Rp2h | -0.5745 | 0.0144 |
| 1012 | Mis12 | -0.5741 | 0.0106 |
| 1013 | Plxna4 | -0.5735 | 0.0202 |
| 1014 | Ndufs4 | -0.5730 | 0.0096 |
| 1015 | Cln8 | -0.5725 | 0.0012 |
| 1016 | Ywhah | -0.5720 | 0.0421 |
| 1017 | Pygb | -0.5719 | 0.0095 |
| 1018 | Gucd1 | -0.5717 | 0.0049 |
| 1019 | Dgcr6 | -0.5715 | 0.0409 |
| 1020 | Card6 | -0.5714 | 0.0002 |
| 1021 | Foxred2 | -0.5713 | 0.0018 |
| 1022 | Heg1 | -0.5713 | 0.0043 |
| 1023 | 5430417L22Rik | -0.5705 | 0.0295 |
| 1024 | Cmtm3 | -0.5701 | 0.0016 |
| 1025 | Wdr13 | -0.5700 | 0.0115 |
| 1026 | Selenbp1 | -0.5698 | 0.0306 |
| 1027 | Ptpn22 | -0.5692 | 0.0000 |
| 1028 | Nt5m | -0.5690 | 0.0122 |
| 1029 | Rnf181 | -0.5684 | 0.0011 |
| 1030 | Iscu | -0.5681 | 0.0365 |
| 1031 | Prdx4 | -0.5678 | 0.0456 |
| 1032 | Pithd1 | -0.5676 | 0.0067 |
| 1033 | Zfp874a | -0.5674 | 0.0238 |
| 1034 | Pck2 | -0.5668 | 0.0006 |
| 1035 | Afap1l1 | -0.5668 | 0.0041 |
| 1036 | Wbp2 | -0.5665 | 0.0030 |
| 1037 | 1700056E22Rik | -0.5664 | 0.0280 |
| 1038 | Capn5 | -0.5654 | 0.0007 |
| 1039 | Hmga2 | -0.5652 | 0.0410 |
| 1040 | Fbxo2 | -0.5649 | 0.0438 |
| 1041 | Sec11c | -0.5640 | 0.0114 |
| 1042 | Gm11696 | -0.5634 | 0.0102 |
| 1043 | Tsc22d2 | -0.5633 | 0.0003 |
| 1044 | Ctnna1 | -0.5632 | 0.0098 |
| 1045 | Rnf115 | -0.5623 | 0.0316 |
| 1046 | Adam9 | -0.5622 | 0.0017 |
| 1047 | Alox12 | -0.5616 | 0.0391 |
| 1048 | Tmem251 | -0.5608 | 0.0010 |
| 1049 | Rnasek | -0.5602 | 0.0071 |
| 1050 | 1700017B05Rik | -0.5599 | 0.0000 |
| 1051 | Gas2l1 | -0.5598 | 0.0261 |
| 1052 | Myo7a | -0.5597 | 0.0085 |
| 1053 | Smdt1 | -0.5596 | 0.0113 |
| 1054 | Lgmn | -0.5589 | 0.0000 |
| 1055 | Derl3 | -0.5586 | 0.0481 |
| 1056 | Parvb | -0.5578 | 0.0178 |
| 1057 | Irf2bpl | -0.5575 | 0.0000 |
| 1058 | Smox | -0.5575 | 0.0352 |
| 1059 | BC031181 | -0.5572 | 0.0142 |
| 1060 | Adipor1 | -0.5567 | 0.0330 |
| 1061 | Stx11 | -0.5563 | 0.0357 |
| 1062 | Rfk | -0.5556 | 0.0403 |
| 1063 | Ddit4 | -0.5554 | 0.0000 |
| 1064 | Amn1 | -0.5552 | 0.0102 |
| 1065 | Wdfy4 | -0.5549 | 0.0238 |
| 1066 | Dynlt1b | -0.5548 | 0.0215 |
| 1067 | Hyls1 | -0.5547 | 0.0273 |
| 1068 | Itpripl1 | -0.5545 | 0.0084 |
| 1069 | 4930404I05Rik | -0.5541 | 0.0277 |
| 1070 | Havcr2 | -0.5537 | 0.0080 |
| 1071 | Tbc1d31 | -0.5536 | 0.0013 |
| 1072 | Pkig | -0.5532 | 0.0010 |
| 1073 | Mgme1 | -0.5531 | 0.0017 |
| 1074 | Rgs1 | -0.5527 | 0.0000 |
| 1075 | Pde4a | -0.5523 | 0.0312 |
| 1076 | Sigmar1 | -0.5521 | 0.0024 |
| 1077 | Txndc16 | -0.5520 | 0.0030 |
| 1078 | Tsga10 | -0.5514 | 0.0309 |
| 1079 | Sh2b3 | -0.5514 | 0.0000 |
| 1080 | Fam117b | -0.5512 | 0.0000 |
| 1081 | Irf5 | -0.5511 | 0.0044 |
| 1082 | Chchd10 | -0.5509 | 0.0194 |
| 1083 | Ovca2 | -0.5508 | 0.0454 |
| 1084 | Tax1bp3 | -0.5505 | 0.0353 |
| 1085 | Carhsp1 | -0.5498 | 0.0314 |
| 1086 | Psenen | -0.5497 | 0.0069 |
| 1087 | Smim3 | -0.5495 | 0.0126 |
| 1088 | Anapc13 | -0.5484 | 0.0040 |
| 1089 | Akt3 | -0.5480 | 0.0115 |
| 1090 | Inf2 | -0.5480 | 0.0043 |
| 1091 | Ifng | -0.5480 | 0.0379 |
| 1092 | Igj | -0.5468 | 0.0000 |
| 1093 | A430093F15Rik | -0.5468 | 0.0476 |
| 1094 | Mpl | -0.5466 | 0.0269 |
| 1095 | Gclm | -0.5451 | 0.0230 |
| 1096 | Kdm2b | -0.5449 | 0.0000 |
| 1097 | Tspyl2 | -0.5443 | 0.0000 |
| 1098 | Fam129b | -0.5442 | 0.0011 |
| 1099 | Emr4 | -0.5439 | 0.0074 |
| 1100 | Chac2 | -0.5437 | 0.0285 |
| 1101 | Ndrg2 | -0.5436 | 0.0202 |
| 1102 | Nudt3 | -0.5427 | 0.0313 |
| 1103 | 1700113A16Rik | -0.5416 | 0.0016 |
| 1104 | Tmem51 | -0.5412 | 0.0028 |
| 1105 | BC029722 | -0.5412 | 0.0026 |
| 1106 | Ctsh | -0.5409 | 0.0024 |
| 1107 | Tiam2 | -0.5396 | 0.0186 |
| 1108 | Gpr56 | -0.5394 | 0.0447 |
| 1109 | Atox1 | -0.5392 | 0.0062 |
| 1110 | Mcfd2 | -0.5378 | 0.0140 |
| 1111 | Syvn1 | -0.5374 | 0.0006 |
| 1112 | Panx1 | -0.5370 | 0.0179 |
| 1113 | Frmd4a | -0.5363 | 0.0018 |
| 1114 | Eif1b | -0.5357 | 0.0022 |
| 1115 | 1110028F11Rik | -0.5357 | 0.0070 |
| 1116 | Tpk1 | -0.5355 | 0.0251 |
| 1117 | Zfp36l2 | -0.5353 | 0.0000 |
| 1118 | Atg12 | -0.5352 | 0.0024 |
| 1119 | Tal1 | -0.5348 | 0.0122 |
| 1120 | Neil1 | -0.5337 | 0.0078 |
| 1121 | Mob3a | -0.5333 | 0.0009 |
| 1122 | St7 | -0.5331 | 0.0234 |
| 1123 | Ccdc102a | -0.5328 | 0.0378 |
| 1124 | BC005764 | -0.5318 | 0.0072 |
| 1125 | Dapk1 | -0.5317 | 0.0008 |
| 1126 | Dnah17 | -0.5310 | 0.0022 |
| 1127 | Abtb1 | -0.5308 | 0.0069 |
| 1128 | Cbr1 | -0.5304 | 0.0073 |
| 1129 | Ppip5k1 | -0.5303 | 0.0053 |
| 1130 | Hdac5 | -0.5301 | 0.0000 |
| 1131 | Myo1c | -0.5300 | 0.0044 |
| 1132 | Rpl41 | -0.5299 | 0.0153 |
| 1133 | Laptm4a | -0.5298 | 0.0018 |
| 1134 | P2ry1 | -0.5297 | 0.0066 |
| 1135 | Ifrd2 | -0.5290 | 0.0424 |
| 1136 | Derl1 | -0.5286 | 0.0039 |
| 1137 | Fcgr2b | -0.5285 | 0.0065 |
| 1138 | Rhobtb2 | -0.5285 | 0.0000 |
| 1139 | Cdc42ep5 | -0.5280 | 0.0408 |
| 1140 | Sod1 | -0.5267 | 0.0093 |
| 1141 | Mcur1 | -0.5265 | 0.0062 |
| 1142 | Nomo1 | -0.5259 | 0.0203 |
| 1143 | Treml1 | -0.5258 | 0.0402 |
| 1144 | Casp3 | -0.5256 | 0.0036 |
| 1145 | Plaur | -0.5250 | 0.0039 |
| 1146 | Rasgrp2 | -0.5249 | 0.0005 |
| 1147 | Gpn3 | -0.5249 | 0.0004 |
| 1148 | Sema4b | -0.5242 | 0.0099 |
| 1149 | Lpcat1 | -0.5240 | 0.0075 |
| 1150 | Slc2a3 | -0.5238 | 0.0148 |
| 1151 | Kel | -0.5230 | 0.0479 |
| 1152 | Gnas | -0.5226 | 0.0129 |
| 1153 | Zfp35 | -0.5221 | 0.0081 |
| 1154 | Fbxw10 | -0.5218 | 0.0264 |
| 1155 | Dnaja4 | -0.5215 | 0.0284 |
| 1156 | Tns3 | -0.5213 | 0.0018 |
| 1157 | Cdt1 | -0.5201 | 0.0006 |
| 1158 | Tufm | -0.5200 | 0.0479 |
| 1159 | Zfp385a | -0.5199 | 0.0113 |
| 1160 | Tmem222 | -0.5197 | 0.0002 |
| 1161 | Tbxas1 | -0.5190 | 0.0320 |
| 1162 | Klhl21 | -0.5187 | 0.0005 |
| 1163 | Fyco1 | -0.5186 | 0.0276 |
| 1164 | Capn10 | -0.5185 | 0.0176 |
| 1165 | Sh3bgrl | -0.5185 | 0.0063 |
| 1166 | Kctd1 | -0.5185 | 0.0449 |
| 1167 | Acaca | -0.5181 | 0.0177 |
| 1168 | Ccr7 | -0.5180 | 0.0028 |
| 1169 | Pon2 | -0.5179 | 0.0139 |
| 1170 | Bcr | -0.5173 | 0.0025 |
| 1171 | 1700021K19Rik | -0.5171 | 0.0013 |
| 1172 | Eif1 | -0.5163 | 0.0010 |
| 1173 | H3f3a | -0.5163 | 0.0076 |
| 1174 | Ralgps2 | -0.5163 | 0.0156 |
| 1175 | Slc7a1 | -0.5163 | 0.0000 |
| 1176 | Spon1 | -0.5156 | 0.0200 |
| 1177 | 1110034G24Rik | -0.5151 | 0.0062 |
| 1178 | Coa7 | -0.5150 | 0.0036 |
| 1179 | Sla2 | -0.5148 | 0.0021 |
| 1180 | Zfp867 | -0.5137 | 0.0486 |
| 1181 | Zfp664 | -0.5137 | 0.0447 |
| 1182 | Siah2 | -0.5132 | 0.0012 |
| 1183 | St6galnac6 | -0.5132 | 0.0144 |
| 1184 | Dnajb6 | -0.5131 | 0.0018 |
| 1185 | Scpep1 | -0.5127 | 0.0087 |
| 1186 | Ppfibp2 | -0.5127 | 0.0140 |
| 1187 | Sypl | -0.5127 | 0.0000 |
| 1188 | Rps12 | -0.5126 | 0.0001 |
| 1189 | Acot2 | -0.5126 | 0.0030 |
| 1190 | Casc4 | -0.5111 | 0.0240 |
| 1191 | Tgtp2 | -0.5109 | 0.0398 |
| 1192 | Arl3 | -0.5108 | 0.0257 |
| 1193 | Bcas3 | -0.5099 | 0.0054 |
| 1194 | Cox6c | -0.5096 | 0.0287 |
| 1195 | Birc2 | -0.5095 | 0.0069 |
| 1196 | Trim7 | -0.5089 | 0.0076 |
| 1197 | Tnfaip8l1 | -0.5085 | 0.0429 |
| 1198 | Mff | -0.5082 | 0.0411 |
| 1199 | Ube2g2 | -0.5075 | 0.0000 |
| 1200 | Treml2 | -0.5074 | 0.0355 |
| 1201 | Rhoq | -0.5070 | 0.0409 |
| 1202 | Gm14005 | -0.5068 | 0.0293 |
| 1203 | Zfp101 | -0.5067 | 0.0440 |
| 1204 | Itm2b | -0.5066 | 0.0082 |
| 1205 | Rbpms | -0.5065 | 0.0227 |
| 1206 | Scmh1 | -0.5057 | 0.0000 |
| 1207 | Arhgef12 | -0.5056 | 0.0079 |
| 1208 | Zfp81 | -0.5054 | 0.0459 |
| 1209 | Gla | -0.5052 | 0.0045 |
| 1210 | Tprgl | -0.5052 | 0.0124 |
| 1211 | Naa50 | -0.5051 | 0.0000 |
| 1212 | Dock1 | -0.5043 | 0.0141 |
| 1213 | Snx5 | -0.5041 | 0.0000 |
| 1214 | Tmem206 | -0.5032 | 0.0311 |
| 1215 | Usp15 | -0.5031 | 0.0443 |
| 1216 | Tusc2 | -0.5030 | 0.0420 |
| 1217 | Ctsb | -0.5029 | 0.0012 |
| 1218 | Cox6b1 | -0.5015 | 0.0079 |
| 1219 | Usp42 | -0.5006 | 0.0006 |
| 1220 | Rpl12 | -0.5004 | 0.0000 |
| 1221 | 2010107E04Rik | -0.5002 | 0.0084 |
| 1222 | Dnlz | -0.5001 | 0.0324 |
| 1223 | Tmem134 | -0.5000 | 0.0185 |
| 1224 | Rpl28 | -0.5000 | 0.0150 |
| 1225 | Slc36a4 | -0.4994 | 0.0065 |
| 1226 | Pou2f2 | -0.4990 | 0.0071 |
| 1227 | D17Wsu92e | -0.4985 | 0.0039 |
| 1228 | Gatc | -0.4981 | 0.0280 |
| 1229 | Dynll2 | -0.4971 | 0.0284 |
| 1230 | Sirt3 | -0.4969 | 0.0266 |
| 1231 | Phykpl | -0.4949 | 0.0238 |
| 1232 | Urm1 | -0.4946 | 0.0007 |
| 1233 | St3gal1 | -0.4933 | 0.0053 |
| 1234 | Ltbp1 | -0.4932 | 0.0323 |
| 1235 | Itprip | -0.4929 | 0.0142 |
| 1236 | Slc8a1 | -0.4925 | 0.0056 |
| 1237 | Wdyhv1 | -0.4922 | 0.0035 |
| 1238 | Coq10b | -0.4918 | 0.0000 |
| 1239 | Ldlrad4 | -0.4912 | 0.0097 |
| 1240 | Slamf7 | -0.4909 | 0.0110 |
| 1241 | Armc7 | -0.4907 | 0.0054 |
| 1242 | Adrbk2 | -0.4903 | 0.0312 |
| 1243 | Pidd1 | -0.4902 | 0.0211 |
| 1244 | Tollip | -0.4901 | 0.0088 |
| 1245 | Ttc28 | -0.4899 | 0.0024 |
| 1246 | Gpr132 | -0.4898 | 0.0020 |
| 1247 | Gemin7 | -0.4892 | 0.0009 |
| 1248 | Lgals3bp | -0.4888 | 0.0000 |
| 1249 | Spty2d1 | -0.4886 | 0.0001 |
| 1250 | Eno3 | -0.4869 | 0.0215 |
| 1251 | Chchd2 | -0.4859 | 0.0089 |
| 1252 | Fchsd2 | -0.4859 | 0.0121 |
| 1253 | Tma7 | -0.4856 | 0.0176 |
| 1254 | Peg13 | -0.4849 | 0.0002 |
| 1255 | Snx30 | -0.4842 | 0.0416 |
| 1256 | Arsk | -0.4842 | 0.0322 |
| 1257 | Slc5a3 | -0.4834 | 0.0110 |
| 1258 | Exoc4 | -0.4828 | 0.0117 |
| 1259 | Nelfe | -0.4826 | 0.0042 |
| 1260 | Akt1s1 | -0.4814 | 0.0001 |
| 1261 | Mknk2 | -0.4809 | 0.0012 |
| 1262 | Dapk2 | -0.4807 | 0.0128 |
| 1263 | Plxnb2 | -0.4803 | 0.0239 |
| 1264 | Pde4d | -0.4796 | 0.0003 |
| 1265 | Slc25a25 | -0.4787 | 0.0039 |
| 1266 | Adamdec1 | -0.4783 | 0.0408 |
| 1267 | Itga6 | -0.4778 | 0.0279 |
| 1268 | Prkce | -0.4778 | 0.0015 |
| 1269 | Sec23a | -0.4764 | 0.0138 |
| 1270 | Rtn4 | -0.4759 | 0.0055 |
| 1271 | Abcg3 | -0.4758 | 0.0018 |
| 1272 | Cdc42bpb | -0.4755 | 0.0000 |
| 1273 | 1810037I17Rik | -0.4744 | 0.0045 |
| 1274 | Snx10 | -0.4736 | 0.0359 |
| 1275 | Lpar6 | -0.4721 | 0.0034 |
| 1276 | G3bp2 | -0.4715 | 0.0057 |
| 1277 | Atic | -0.4709 | 0.0442 |
| 1278 | Map1lc3b | -0.4708 | 0.0144 |
| 1279 | Ptpn11 | -0.4705 | 0.0418 |
| 1280 | Zfp467 | -0.4697 | 0.0499 |
| 1281 | Xkrx | -0.4696 | 0.0473 |
| 1282 | Mrm1 | -0.4696 | 0.0165 |
| 1283 | 5430416N02Rik | -0.4695 | 0.0033 |
| 1284 | Tmod3 | -0.4694 | 0.0094 |
| 1285 | Mpv17l2 | -0.4692 | 0.0237 |
| 1286 | Slc22a23 | -0.4690 | 0.0342 |
| 1287 | Tsc22d3 | -0.4687 | 0.0000 |
| 1288 | Fntb | -0.4686 | 0.0318 |
| 1289 | Gt(ROSA)26Sor | -0.4684 | 0.0074 |
| 1290 | Gmppb | -0.4677 | 0.0407 |
| 1291 | Tspo | -0.4674 | 0.0479 |
| 1292 | Ubc | -0.4671 | 0.0012 |
| 1293 | Atp6v0e | -0.4654 | 0.0114 |
| 1294 | Mrps18b | -0.4644 | 0.0029 |
| 1295 | Mbnl1 | -0.4640 | 0.0158 |
| 1296 | BC030336 | -0.4638 | 0.0221 |
| 1297 | Dazap2 | -0.4637 | 0.0105 |
| 1298 | Rpl36 | -0.4634 | 0.0008 |
| 1299 | Gfod1 | -0.4629 | 0.0002 |
| 1300 | Spcs1 | -0.4628 | 0.0149 |
| 1301 | Ppm1k | -0.4617 | 0.0041 |
| 1302 | Gm11974 | -0.4611 | 0.0010 |
| 1303 | Mob2 | -0.4609 | 0.0065 |
| 1304 | Limk1 | -0.4606 | 0.0067 |
| 1305 | Zfp157 | -0.4603 | 0.0394 |
| 1306 | 1500011B03Rik | -0.4598 | 0.0307 |
| 1307 | Ptpn12 | -0.4597 | 0.0193 |
| 1308 | Slc35d1 | -0.4596 | 0.0041 |
| 1309 | Vkorc1 | -0.4596 | 0.0427 |
| 1310 | Ap1s3 | -0.4586 | 0.0270 |
| 1311 | Ptprj | -0.4586 | 0.0000 |
| 1312 | Fanci | -0.4584 | 0.0340 |
| 1313 | Rbm15 | -0.4582 | 0.0010 |
| 1314 | Leprot | -0.4577 | 0.0141 |
| 1315 | Cd38 | -0.4575 | 0.0465 |
| 1316 | Gm3258 | -0.4574 | 0.0031 |
| 1317 | Hsd3b7 | -0.4572 | 0.0043 |
| 1318 | Ndufa7 | -0.4568 | 0.0044 |
| 1319 | Stx1a | -0.4560 | 0.0058 |
| 1320 | Ankrd10 | -0.4555 | 0.0002 |
| 1321 | St8sia4 | -0.4553 | 0.0019 |
| 1322 | Kbtbd4 | -0.4552 | 0.0028 |
| 1323 | Lrrc28 | -0.4548 | 0.0384 |
| 1324 | Setd3 | -0.4544 | 0.0060 |
| 1325 | Tssc4 | -0.4538 | 0.0098 |
| 1326 | Dpy19l1 | -0.4528 | 0.0011 |
| 1327 | Rab2b | -0.4521 | 0.0124 |
| 1328 | Rusc1 | -0.4515 | 0.0006 |
| 1329 | Stat2 | -0.4514 | 0.0121 |
| 1330 | Itk | -0.4506 | 0.0000 |
| 1331 | Plcxd2 | -0.4497 | 0.0028 |
| 1332 | Pepd | -0.4486 | 0.0047 |
| 1333 | Tgfb1 | -0.4483 | 0.0001 |
| 1334 | Dbf4 | -0.4479 | 0.0008 |
| 1335 | Rasl10a | -0.4478 | 0.0156 |
| 1336 | Tcta | -0.4476 | 0.0218 |
| 1337 | Rab3ip | -0.4472 | 0.0084 |
| 1338 | Slc20a2 | -0.4469 | 0.0285 |
| 1339 | Pef1 | -0.4464 | 0.0108 |
| 1340 | Pbx3 | -0.4463 | 0.0496 |
| 1341 | Pld2 | -0.4462 | 0.0360 |
| 1342 | Camkk2 | -0.4458 | 0.0205 |
| 1343 | Zbtb34 | -0.4448 | 0.0451 |
| 1344 | Cobll1 | -0.4444 | 0.0433 |
| 1345 | Cdkn1a | -0.4443 | 0.0026 |
| 1346 | Ywhag | -0.4443 | 0.0000 |
| 1347 | Fam126a | -0.4441 | 0.0193 |
| 1348 | Dot1l | -0.4440 | 0.0058 |
| 1349 | Rtn3 | -0.4432 | 0.0374 |
| 1350 | Arhgap18 | -0.4432 | 0.0305 |
| 1351 | Drap1 | -0.4432 | 0.0173 |
| 1352 | Pabpc1 | -0.4426 | 0.0357 |
| 1353 | Rcc1 | -0.4425 | 0.0094 |
| 1354 | Dpm3 | -0.4424 | 0.0224 |
| 1355 | Stoml1 | -0.4421 | 0.0203 |
| 1356 | Gpr114 | -0.4418 | 0.0011 |
| 1357 | Lamp1 | -0.4402 | 0.0074 |
| 1358 | Serpinb9 | -0.4402 | 0.0003 |
| 1359 | 2610002J02Rik | -0.4400 | 0.0276 |
| 1360 | Trim65 | -0.4394 | 0.0054 |
| 1361 | Hspa4 | -0.4393 | 0.0000 |
| 1362 | Usp12 | -0.4390 | 0.0001 |
| 1363 | Nedd8 | -0.4390 | 0.0405 |
| 1364 | Ubl5 | -0.4389 | 0.0123 |
| 1365 | Rap2a | -0.4387 | 0.0089 |
| 1366 | Txnl1 | -0.4376 | 0.0140 |
| 1367 | Calm2 | -0.4373 | 0.0006 |
| 1368 | Hexa | -0.4370 | 0.0140 |
| 1369 | Atp5h | -0.4368 | 0.0226 |
| 1370 | Cox8a | -0.4367 | 0.0106 |
| 1371 | Evi5 | -0.4364 | 0.0109 |
| 1372 | Trafd1 | -0.4359 | 0.0008 |
| 1373 | Htra2 | -0.4353 | 0.0026 |
| 1374 | Lrpap1 | -0.4351 | 0.0001 |
| 1375 | Pole3 | -0.4349 | 0.0035 |
| 1376 | Slc38a2 | -0.4348 | 0.0063 |
| 1377 | Impdh2 | -0.4346 | 0.0129 |
| 1378 | Ptms | -0.4345 | 0.0229 |
| 1379 | Dusp3 | -0.4343 | 0.0137 |
| 1380 | Mtmr4 | -0.4342 | 0.0122 |
| 1381 | Lax1 | -0.4339 | 0.0101 |
| 1382 | Tmem57 | -0.4333 | 0.0000 |
| 1383 | Kif3c | -0.4330 | 0.0331 |
| 1384 | Ccdc23 | -0.4330 | 0.0205 |
| 1385 | Ticam1 | -0.4321 | 0.0356 |
| 1386 | St3gal3 | -0.4320 | 0.0115 |
| 1387 | Tex30 | -0.4319 | 0.0368 |
| 1388 | Kifc3 | -0.4314 | 0.0040 |
| 1389 | Nmb | -0.4311 | 0.0303 |
| 1390 | Prr3 | -0.4307 | 0.0325 |
| 1391 | Erlin1 | -0.4305 | 0.0485 |
| 1392 | Dok1 | -0.4303 | 0.0066 |
| 1393 | Lat2 | -0.4286 | 0.0238 |
| 1394 | Ppan | -0.4282 | 0.0004 |
| 1395 | Tm2d2 | -0.4278 | 0.0004 |
| 1396 | Higd2a | -0.4270 | 0.0038 |
| 1397 | Etnk1 | -0.4269 | 0.0023 |
| 1398 | Mtm1 | -0.4265 | 0.0201 |
| 1399 | Rpl10a | -0.4261 | 0.0038 |
| 1400 | Fermt3 | -0.4258 | 0.0352 |
| 1401 | Trp53i11 | -0.4258 | 0.0349 |
| 1402 | Arhgap17 | -0.4256 | 0.0145 |
| 1403 | Uqcr11 | -0.4247 | 0.0465 |
| 1404 | Uqcc1 | -0.4246 | 0.0217 |
| 1405 | Tsr3 | -0.4239 | 0.0240 |
| 1406 | Ctu2 | -0.4238 | 0.0151 |
| 1407 | Tnik | -0.4231 | 0.0403 |
| 1408 | 1810026B05Rik | -0.4230 | 0.0066 |
| 1409 | Dusp4 | -0.4229 | 0.0331 |
| 1410 | Gch1 | -0.4224 | 0.0348 |
| 1411 | Rap1b | -0.4214 | 0.0122 |
| 1412 | Mob3b | -0.4213 | 0.0121 |
| 1413 | Gtpbp6 | -0.4196 | 0.0313 |
| 1414 | Man1a | -0.4195 | 0.0011 |
| 1415 | Smtn | -0.4194 | 0.0279 |
| 1416 | Cse1l | -0.4192 | 0.0007 |
| 1417 | Psmg2 | -0.4186 | 0.0455 |
| 1418 | Cd36 | -0.4185 | 0.0000 |
| 1419 | Abcg1 | -0.4178 | 0.0230 |
| 1420 | Yy2 | -0.4177 | 0.0482 |
| 1421 | B4galnt1 | -0.4168 | 0.0004 |
| 1422 | Acot7 | -0.4168 | 0.0046 |
| 1423 | Gna12 | -0.4164 | 0.0167 |
| 1424 | Naga | -0.4162 | 0.0348 |
| 1425 | Msi2 | -0.4162 | 0.0148 |
| 1426 | Kpnb1 | -0.4159 | 0.0000 |
| 1427 | Grap2 | -0.4155 | 0.0080 |
| 1428 | Tprn | -0.4153 | 0.0136 |
| 1429 | Camk1 | -0.4150 | 0.0277 |
| 1430 | Hnrnpa1 | -0.4146 | 0.0033 |
| 1431 | Gnptg | -0.4146 | 0.0153 |
| 1432 | Skil | -0.4142 | 0.0164 |
| 1433 | Cfl2 | -0.4138 | 0.0395 |
| 1434 | Hk1 | -0.4133 | 0.0333 |
| 1435 | Mien1 | -0.4118 | 0.0065 |
| 1436 | Phc2 | -0.4117 | 0.0000 |
| 1437 | Tubb4b | -0.4116 | 0.0145 |
| 1438 | 3110082I17Rik | -0.4113 | 0.0205 |
| 1439 | Tfb2m | -0.4108 | 0.0039 |
| 1440 | Endod1 | -0.4107 | 0.0081 |
| 1441 | Emd | -0.4106 | 0.0000 |
| 1442 | Hnrnpl | -0.4102 | 0.0000 |
| 1443 | Map1lc3a | -0.4099 | 0.0105 |
| 1444 | Tmem50b | -0.4098 | 0.0000 |
| 1445 | Aff3 | -0.4097 | 0.0001 |
| 1446 | Pld3 | -0.4096 | 0.0015 |
| 1447 | Cd7 | -0.4095 | 0.0298 |
| 1448 | Fgfr1op | -0.4090 | 0.0395 |
| 1449 | Rcc2 | -0.4088 | 0.0008 |
| 1450 | Hs6st1 | -0.4087 | 0.0155 |
| 1451 | Ppp1r14b | -0.4085 | 0.0343 |
| 1452 | Fez2 | -0.4074 | 0.0309 |
| 1453 | Snap47 | -0.4070 | 0.0414 |
| 1454 | Rbm38 | -0.4065 | 0.0074 |
| 1455 | AI504432 | -0.4056 | 0.0137 |
| 1456 | Mast4 | -0.4056 | 0.0144 |
| 1457 | Tec | -0.4054 | 0.0218 |
| 1458 | Rabac1 | -0.4048 | 0.0023 |
| 1459 | Dclk2 | -0.4045 | 0.0326 |
| 1460 | Ppp1r21 | -0.4029 | 0.0088 |
| 1461 | Prcp | -0.4020 | 0.0363 |
| 1462 | Ada | -0.4020 | 0.0172 |
| 1463 | Psmb5 | -0.4015 | 0.0322 |
| 1464 | Mrpl49 | -0.4012 | 0.0334 |
| 1465 | Abcb9 | -0.4011 | 0.0162 |
| 1466 | Pla2g6 | -0.4004 | 0.0269 |
| 1467 | Atg4a | -0.3996 | 0.0408 |
| 1468 | Vamp1 | -0.3994 | 0.0137 |
| 1469 | Taf5 | -0.3994 | 0.0160 |
| 1470 | Cyb561a3 | -0.3982 | 0.0067 |
| 1471 | Tesk1 | -0.3978 | 0.0353 |
| 1472 | Cxcr4 | -0.3978 | 0.0058 |
| 1473 | Fam13b | -0.3975 | 0.0448 |
| 1474 | Golga7 | -0.3969 | 0.0283 |
| 1475 | Twf1 | -0.3967 | 0.0291 |
| 1476 | 1810032O08Rik | -0.3957 | 0.0107 |
| 1477 | Irgq | -0.3944 | 0.0298 |
| 1478 | Rcan1 | -0.3938 | 0.0314 |
| 1479 | Xaf1 | -0.3935 | 0.0279 |
| 1480 | Elmsan1 | -0.3935 | 0.0009 |
| 1481 | Gimap1 | -0.3932 | 0.0102 |
| 1482 | Irf8 | -0.3926 | 0.0304 |
| 1483 | Dgkz | -0.3926 | 0.0009 |
| 1484 | Gimap5 | -0.3925 | 0.0054 |
| 1485 | Chst12 | -0.3924 | 0.0328 |
| 1486 | Tceb1 | -0.3912 | 0.0317 |
| 1487 | Runx3 | -0.3902 | 0.0077 |
| 1488 | Ahsa1 | -0.3902 | 0.0241 |
| 1489 | Pold4 | -0.3898 | 0.0221 |
| 1490 | Galnt6 | -0.3897 | 0.0003 |
| 1491 | Ptplad1 | -0.3891 | 0.0007 |
| 1492 | Lmna | -0.3878 | 0.0292 |
| 1493 | Nans | -0.3876 | 0.0143 |
| 1494 | Tmem128 | -0.3873 | 0.0002 |
| 1495 | Rmnd5a | -0.3871 | 0.0178 |
| 1496 | Stard3 | -0.3869 | 0.0144 |
| 1497 | Cd28 | -0.3867 | 0.0388 |
| 1498 | Slc25a38 | -0.3865 | 0.0045 |
| 1499 | Ints5 | -0.3862 | 0.0070 |
| 1500 | Zfr | -0.3861 | 0.0232 |
| 1501 | Slc22a5 | -0.3859 | 0.0098 |
| 1502 | Bbip1 | -0.3852 | 0.0328 |
| 1503 | Dhrs3 | -0.3851 | 0.0288 |
| 1504 | Taf13 | -0.3840 | 0.0323 |
| 1505 | Als2 | -0.3830 | 0.0401 |
| 1506 | Brd2 | -0.3819 | 0.0073 |
| 1507 | Tshz1 | -0.3818 | 0.0332 |
| 1508 | Psap | -0.3817 | 0.0054 |
| 1509 | Dad1 | -0.3816 | 0.0002 |
| 1510 | Ndufa2 | -0.3815 | 0.0238 |
| 1511 | Cox20 | -0.3809 | 0.0363 |
| 1512 | Ceacam1 | -0.3809 | 0.0177 |
| 1513 | Cdkn2aip | -0.3808 | 0.0043 |
| 1514 | Zbed4 | -0.3808 | 0.0402 |
| 1515 | Cldn25 | -0.3803 | 0.0035 |
| 1516 | Dxo | -0.3793 | 0.0022 |
| 1517 | Cenpa | -0.3793 | 0.0415 |
| 1518 | Anapc16 | -0.3791 | 0.0016 |
| 1519 | Trappc9 | -0.3789 | 0.0385 |
| 1520 | Srsf2 | -0.3779 | 0.0001 |
| 1521 | Akap2 | -0.3762 | 0.0470 |
| 1522 | Pgm2l1 | -0.3761 | 0.0120 |
| 1523 | Ndufa3 | -0.3749 | 0.0297 |
| 1524 | Trappc4 | -0.3745 | 0.0186 |
| 1525 | Vps37b | -0.3738 | 0.0040 |
| 1526 | Abt1 | -0.3729 | 0.0085 |
| 1527 | Otulin | -0.3726 | 0.0389 |
| 1528 | Nucb2 | -0.3721 | 0.0345 |
| 1529 | Mad2l1bp | -0.3715 | 0.0231 |
| 1530 | Adap1 | -0.3709 | 0.0288 |
| 1531 | Mrps21 | -0.3707 | 0.0056 |
| 1532 | Ccdc50 | -0.3700 | 0.0201 |
| 1533 | Ptk2 | -0.3698 | 0.0335 |
| 1534 | Stat5a | -0.3698 | 0.0111 |
| 1535 | Nfkbil1 | -0.3697 | 0.0469 |
| 1536 | Reep4 | -0.3695 | 0.0188 |
| 1537 | Agpat4 | -0.3688 | 0.0357 |
| 1538 | Fasn | -0.3681 | 0.0260 |
| 1539 | Galnt2 | -0.3680 | 0.0234 |
| 1540 | Susd3 | -0.3675 | 0.0153 |
| 1541 | Zfand5 | -0.3674 | 0.0000 |
| 1542 | Pold1 | -0.3662 | 0.0171 |
| 1543 | Ncor2 | -0.3653 | 0.0278 |
| 1544 | Usp36 | -0.3644 | 0.0000 |
| 1545 | Rcor1 | -0.3640 | 0.0044 |
| 1546 | Abhd17a | -0.3639 | 0.0044 |
| 1547 | Zc3hav1l | -0.3639 | 0.0369 |
| 1548 | Idh2 | -0.3638 | 0.0405 |
| 1549 | Mfng | -0.3630 | 0.0078 |
| 1550 | Rrp1b | -0.3627 | 0.0113 |
| 1551 | Uqcr10 | -0.3620 | 0.0074 |
| 1552 | Ipo7 | -0.3619 | 0.0000 |
| 1553 | Lst1 | -0.3619 | 0.0236 |
| 1554 | Bcl2l2 | -0.3612 | 0.0432 |
| 1555 | Mturn | -0.3609 | 0.0351 |
| 1556 | Sulf2 | -0.3609 | 0.0389 |
| 1557 | Incenp | -0.3599 | 0.0021 |
| 1558 | Atp13a2 | -0.3598 | 0.0016 |
| 1559 | Ddx54 | -0.3597 | 0.0029 |
| 1560 | Acss1 | -0.3596 | 0.0024 |
| 1561 | Sms | -0.3588 | 0.0140 |
| 1562 | Dohh | -0.3587 | 0.0205 |
| 1563 | Rps15a-ps4 | -0.3578 | 0.0228 |
| 1564 | Map3k1 | -0.3577 | 0.0061 |
| 1565 | Gcsh | -0.3575 | 0.0420 |
| 1566 | Aes | -0.3569 | 0.0371 |
| 1567 | Zyg11b | -0.3557 | 0.0000 |
| 1568 | Ccdc117 | -0.3556 | 0.0114 |
| 1569 | Stk19 | -0.3554 | 0.0198 |
| 1570 | Cish | -0.3554 | 0.0208 |
| 1571 | Cst3 | -0.3553 | 0.0007 |
| 1572 | Rpl10 | -0.3553 | 0.0024 |
| 1573 | Crcp | -0.3550 | 0.0478 |
| 1574 | Pdzk1ip1 | -0.3546 | 0.0193 |
| 1575 | Oxa1l | -0.3545 | 0.0044 |
| 1576 | Naaa | -0.3542 | 0.0340 |
| 1577 | BC005537 | -0.3541 | 0.0138 |
| 1578 | Zfp180 | -0.3541 | 0.0478 |
| 1579 | Mink1 | -0.3540 | 0.0191 |
| 1580 | Atp5k | -0.3537 | 0.0439 |
| 1581 | Dynlrb1 | -0.3527 | 0.0299 |
| 1582 | Ddr1 | -0.3525 | 0.0367 |
| 1583 | Srsf3 | -0.3518 | 0.0001 |
| 1584 | Bcat2 | -0.3514 | 0.0174 |
| 1585 | Actn4 | -0.3514 | 0.0374 |
| 1586 | 3110043O21Rik | -0.3505 | 0.0003 |
| 1587 | Itm2c | -0.3500 | 0.0013 |
| 1588 | Rsrp1 | -0.3492 | 0.0136 |
| 1589 | Ngfrap1 | -0.3488 | 0.0219 |
| 1590 | Yrdc | -0.3484 | 0.0017 |
| 1591 | Brpf3 | -0.3483 | 0.0308 |
| 1592 | Usp54 | -0.3477 | 0.0195 |
| 1593 | Rbm3 | -0.3472 | 0.0098 |
| 1594 | Tdp2 | -0.3469 | 0.0019 |
| 1595 | Calr | -0.3469 | 0.0010 |
| 1596 | Itpr1 | -0.3453 | 0.0393 |
| 1597 | Maf1 | -0.3443 | 0.0000 |
| 1598 | Usp4 | -0.3441 | 0.0265 |
| 1599 | Dscr3 | -0.3439 | 0.0376 |
| 1600 | Samm50 | -0.3435 | 0.0304 |
| 1601 | Socs7 | -0.3433 | 0.0429 |
| 1602 | Rabggtb | -0.3429 | 0.0073 |
| 1603 | Ncbp2 | -0.3428 | 0.0397 |
| 1604 | Tmem50a | -0.3423 | 0.0250 |
| 1605 | Hcfc1 | -0.3419 | 0.0006 |
| 1606 | Usp10 | -0.3415 | 0.0036 |
| 1607 | H2-D1 | -0.3415 | 0.0098 |
| 1608 | Ino80c | -0.3410 | 0.0390 |
| 1609 | Srebf2 | -0.3410 | 0.0174 |
| 1610 | Tbc1d5 | -0.3403 | 0.0277 |
| 1611 | Prmt1 | -0.3398 | 0.0042 |
| 1612 | Mat2a | -0.3391 | 0.0000 |
| 1613 | Rbm19 | -0.3388 | 0.0411 |
| 1614 | Pdcd5 | -0.3384 | 0.0402 |
| 1615 | Pi4k2b | -0.3376 | 0.0424 |
| 1616 | Btk | -0.3366 | 0.0120 |
| 1617 | 4930523C07Rik | -0.3365 | 0.0008 |
| 1618 | Pdrg1 | -0.3360 | 0.0438 |
| 1619 | Slc39a10 | -0.3359 | 0.0378 |
| 1620 | Dcaf11 | -0.3357 | 0.0139 |
| 1621 | Trim35 | -0.3350 | 0.0233 |
| 1622 | 9-Sep | -0.3349 | 0.0012 |
| 1623 | Ank | -0.3348 | 0.0046 |
| 1624 | Rpl5 | -0.3347 | 0.0169 |
| 1625 | Ube2j1 | -0.3344 | 0.0013 |
| 1626 | Ptk2b | -0.3340 | 0.0005 |
| 1627 | Mprip | -0.3333 | 0.0181 |
| 1628 | Uba7 | -0.3330 | 0.0286 |
| 1629 | Fam89b | -0.3328 | 0.0341 |
| 1630 | Uqcrh | -0.3325 | 0.0275 |
| 1631 | Tapbpl | -0.3317 | 0.0144 |
| 1632 | Edem1 | -0.3316 | 0.0409 |
| 1633 | Cnot8 | -0.3315 | 0.0044 |
| 1634 | Sash3 | -0.3314 | 0.0469 |
| 1635 | Mnt | -0.3306 | 0.0006 |
| 1636 | Slc39a9 | -0.3305 | 0.0236 |
| 1637 | Tmem115 | -0.3302 | 0.0333 |
| 1638 | Topors | -0.3300 | 0.0032 |
| 1639 | Crtc1 | -0.3300 | 0.0224 |
| 1640 | Aff4 | -0.3291 | 0.0001 |
| 1641 | Mtdh | -0.3289 | 0.0017 |
| 1642 | Laptm5 | -0.3285 | 0.0049 |
| 1643 | Rftn1 | -0.3285 | 0.0079 |
| 1644 | Zfp706 | -0.3281 | 0.0437 |
| 1645 | Myo9a | -0.3277 | 0.0195 |
| 1646 | Manf | -0.3276 | 0.0131 |
| 1647 | Sumo3 | -0.3276 | 0.0026 |
| 1648 | Trak1 | -0.3274 | 0.0000 |
| 1649 | Smg5 | -0.3272 | 0.0013 |
| 1650 | Abcc1 | -0.3268 | 0.0004 |
| 1651 | Atg101 | -0.3265 | 0.0477 |
| 1652 | Csnk1g2 | -0.3262 | 0.0006 |
| 1653 | Srsf9 | -0.3250 | 0.0465 |
| 1654 | Fubp1 | -0.3244 | 0.0009 |
| 1655 | Gm614 | -0.3237 | 0.0336 |
| 1656 | Cops3 | -0.3231 | 0.0019 |
| 1657 | Azin1 | -0.3230 | 0.0198 |
| 1658 | Rassf2 | -0.3229 | 0.0420 |
| 1659 | Ptpra | -0.3227 | 0.0133 |
| 1660 | Mesdc2 | -0.3224 | 0.0192 |
| 1661 | Necap2 | -0.3203 | 0.0378 |
| 1662 | Elovl1 | -0.3200 | 0.0151 |
| 1663 | Atp5d | -0.3191 | 0.0051 |
| 1664 | Ubl3 | -0.3190 | 0.0018 |
| 1665 | Acox1 | -0.3189 | 0.0168 |
| 1666 | Per1 | -0.3171 | 0.0320 |
| 1667 | Stx4a | -0.3168 | 0.0013 |
| 1668 | Arpc3 | -0.3166 | 0.0119 |
| 1669 | Ppm1b | -0.3162 | 0.0464 |
| 1670 | Fen1 | -0.3159 | 0.0284 |
| 1671 | Irf2bp2 | -0.3157 | 0.0217 |
| 1672 | Impact | -0.3153 | 0.0009 |
| 1673 | Ppdpf | -0.3149 | 0.0100 |
| 1674 | Mtf2 | -0.3142 | 0.0137 |
| 1675 | Grn | -0.3141 | 0.0200 |
| 1676 | Wdr18 | -0.3141 | 0.0169 |
| 1677 | Slbp | -0.3140 | 0.0128 |
| 1678 | Ilf3 | -0.3140 | 0.0004 |
| 1679 | Tinf2 | -0.3135 | 0.0019 |
| 1680 | Ubfd1 | -0.3129 | 0.0295 |
| 1681 | Cnbp | -0.3128 | 0.0062 |
| 1682 | Lyz2 | -0.3125 | 0.0042 |
| 1683 | Inip | -0.3118 | 0.0374 |
| 1684 | Nolc1 | -0.3116 | 0.0061 |
| 1685 | D8Ertd738e | -0.3115 | 0.0157 |
| 1686 | Rpl3 | -0.3114 | 0.0039 |
| 1687 | Sepw1 | -0.3107 | 0.0198 |
| 1688 | Fam20b | -0.3103 | 0.0439 |
| 1689 | Slc25a22 | -0.3098 | 0.0329 |
| 1690 | Pgap1 | -0.3090 | 0.0064 |
| 1691 | Unc119b | -0.3081 | 0.0149 |
| 1692 | Skp1a | -0.3055 | 0.0323 |
| 1693 | Zfp146 | -0.3049 | 0.0183 |
| 1694 | Sav1 | -0.3046 | 0.0243 |
| 1695 | Phf5a | -0.3046 | 0.0093 |
| 1696 | Ywhae | -0.3040 | 0.0049 |
| 1697 | Rplp2 | -0.3032 | 0.0098 |
| 1698 | Pqlc1 | -0.3031 | 0.0344 |
| 1699 | Tnfrsf4 | -0.3028 | 0.0414 |
| 1700 | Rab43 | -0.3024 | 0.0033 |
| 1701 | 5031439G07Rik | -0.3022 | 0.0318 |
| 1702 | Tbrg4 | -0.3019 | 0.0236 |
| 1703 | Cdk5rap2 | -0.3015 | 0.0252 |
| 1704 | Fbxo33 | -0.3008 | 0.0249 |
| 1705 | Crkl | -0.3007 | 0.0016 |
| 1706 | Snx3 | -0.3007 | 0.0096 |
| 1707 | Faah | -0.3003 | 0.0065 |
| 1708 | Capns1 | -0.3001 | 0.0392 |
| 1709 | Rbmx | -0.3000 | 0.0049 |
| 1710 | Ccdc127 | -0.2993 | 0.0496 |
| 1711 | Hmgcr | -0.2992 | 0.0340 |
| 1712 | Zeb2 | -0.2982 | 0.0181 |
| 1713 | Gdi2 | -0.2977 | 0.0006 |
| 1714 | Trim28 | -0.2976 | 0.0041 |
| 1715 | Gmpr2 | -0.2972 | 0.0160 |
| 1716 | Btg2 | -0.2962 | 0.0340 |
| 1717 | Rabep1 | -0.2957 | 0.0062 |
| 1718 | Fam134c | -0.2939 | 0.0100 |
| 1719 | Szrd1 | -0.2928 | 0.0016 |
| 1720 | Mid1ip1 | -0.2924 | 0.0108 |
| 1721 | Pou6f1 | -0.2922 | 0.0318 |
| 1722 | Tmem167b | -0.2911 | 0.0145 |
| 1723 | Cdk9 | -0.2909 | 0.0294 |
| 1724 | Cers4 | -0.2906 | 0.0282 |
| 1725 | Dok2 | -0.2904 | 0.0203 |
| 1726 | Nedd4 | -0.2900 | 0.0469 |
| 1727 | Actr1b | -0.2898 | 0.0050 |
| 1728 | Snhg8 | -0.2885 | 0.0470 |
| 1729 | Pomp | -0.2883 | 0.0162 |
| 1730 | Lsm3 | -0.2883 | 0.0403 |
| 1731 | Ggps1 | -0.2880 | 0.0349 |
| 1732 | Cdc42se2 | -0.2878 | 0.0004 |
| 1733 | Fam107b | -0.2876 | 0.0005 |
| 1734 | Map4k3 | -0.2875 | 0.0427 |
| 1735 | P2ry10 | -0.2868 | 0.0016 |
| 1736 | Fiz1 | -0.2863 | 0.0114 |
| 1737 | Zfp142 | -0.2856 | 0.0316 |
| 1738 | Btbd1 | -0.2852 | 0.0461 |
| 1739 | Spsb3 | -0.2850 | 0.0371 |
| 1740 | Nr1d2 | -0.2845 | 0.0148 |
| 1741 | Inpp5d | -0.2842 | 0.0081 |

Supplementary Table 5. Primers sequences for real-time PCR.

| **Primer** | **Primer sequence** | **Size (bp)** |
| --- | --- | --- |
| DUSP1 (Homo sapiens) | F: 5’- AGTACCCCACTCTACGATCAGG -3’  R: 5’- GAAGCGTGATACGCACTGC -3’ | 77 |
| RXRA (Homo sapiens) | F: 5’- ATGGACACCAAACATTTCCTGC -3’  R: 5’- GGGAGCTGATGACCGAGAAAG -3’ | 211 |
| CNR1 (Homo sapiens) | F: 5’- TTACAACAAGTCTCTCTCGTCCT -3’  R: 5’- GGCTGCCGATGAAGTGGTA -3’ | 233 |
| INSR (Homo sapiens) | F: 5’- TCTTCCACAGCGAGGAGAAC -3’  R: 5’- ACCGAGGCAAGGTCAGAATC -3’ | 228 |
| ADRB3 (Homo sapiens) | F: 5’- TACTCCCTTCCCTTCTACTC -3’  R: 5’- GCTACTCAATGGCAACTG -3’ | 197 |
| PPARG (Homo sapiens) | F: 5’- GCCGAGAAGGAGAAGCTGTTG -3’  R: 5’- CCTGGGCGGTTGATTTGTCTG -3’ | 184 |
| GAPDH (Homo sapiens) | F: 5’- CACCCACTCCTCCACCTTTG -3’  R: 5’- CCACCACCCTGTTGCTGTAG -3’ | 110 |
| Dusp1 (Mus musculus) | F: 5’- GTGCCTATCACGCTTCTC -3’  R: 5’- GTCTGCCTTGTGGTTGTC -3’ | 143 |
| Rxra (Mus musculus) | F: 5’- GATGGCACCACCAATCATC -3’  R: 5’- CATGGTTCAGCTCCAAGTC -3’ | 165 |
| Cnr1 (Mus musculus) | F: 5’- CAAGCACGCCAATAACAC -3’  R: 5’- ACCGATGAGACAACAGAC -3’ | 201 |
| Insr (Mus musculus) | F: 5’- GCTGAGCTGGAGGCTAACCTTG -3’  R: 5’- GAGCTTGCCCTGAGTGATGGTG -3’ | 225 |
| Adrb3 (Mus musculus) | F: 5’- CAGTCCCTGCCTATGTTTGTG -3’  R: 5’- GGTCCAAGATGGTGCTTAGAG -3’ | 230 |
| Pparg (Mus musculus) | F: 5’- CCGTAGAAGCCGTGCAAGAG -3’  R: 5’- TCATCAGGGAGGCCAGCATC -3’ | 138 |
| Il1b (Mus musculus) | F: 5’- CCCAAGCAATACCCAAAG -3’  R: 5’- CCTGACCACTGTTGTTTC -3’ | 163 |
| GAPDH (Mus musculus) | F: 5’- CTGCCCAGAACATCATCC -3’  R: 5’- CTCAGATGCCTGCTTCAC -3’ | 197 |

**Supplementary Table 6.**  DEGs have been reported in the study of Ray S et al. [^1^](#_ENREF_1)

| **Reference** |  | **DEGs** |
| --- | --- | --- |
| Postpartum Depression | Up-regulated | Per2 |
|  | Up-regulated | Mthfr |
| Depressive disorder | Up-regulated | Per2, Ptgs2, Adrb2, Bhlhe40, Dgkh, Arntl2, Rps6ka2, Adrb1, Cry1, Grin3a |
|  | Dow-regulated | Mthfr, Hspa1b, Cat, Gad1, Serpine1, Gpx1, Mdga1, Apoe, Nr3c2, Vegfa, Csgalnact1, Pla2g4a, Aldh2, Npy, Hdac5, Ptk2b, Per1, Zeb2 |

Reference: 1. Ray S, Tzeng R-Y, DiCarlo LM, et al. An examination of dynamic gene expression changes in the mouse brain during pregnancy and the postpartum period*. G3: Genes, Genomes, Genetics*. 2016;6(1):221-233.
